# Improving Movement Behavior in People after Stroke with the RISE Intervention: A Randomized Multiple Baseline Study

**DOI:** 10.3390/jcm13154341

**Published:** 2024-07-25

**Authors:** Wendy Hendrickx, Roderick Wondergem, Cindy Veenhof, Coralie English, Johanna M. A. Visser-Meily, Martijn F. Pisters

**Affiliations:** 1 Research Group Empowering Healthy Behavior, Department of Health Innovations and Technology, Fontys University of Applied Sciences, 5600 AH Eindhoven, The Netherlands; w.hendrickx@umcutrecht.nl; 2 Department of Rehabilitation, Physiotherapy Science & Sport, UMC Utrecht Brain Center, Utrecht University, 3584 CX Utrecht, The Netherlands; 3 Center for Physical Therapy Research and Innovation in Primary Care, Julius Health Care Centers, 3454 PV De Meern, The Netherlands; 4 School of Sport Studies, Fontys University of Applied Sciences, 5644 HZ Eindhoven, The Netherlands; 5 Research Group Innovation of Human Movement Care, University of Applied Sciences Utrecht, 3584 CS Utrecht, The Netherlands; 6 School of Health Sciences, University of Newcastle, Callaghan, NSW 2308, Australia; 7 Heart and Stroke Program, Hunter Medical Research Institute, Newcastle, NSW 2305, Australia; 8 Centre of Research Excellence to Accelerate Stroke Trial Innovation and Translation, University of Sydney, Sydney, NSW 2010, Australia; 9 Center of Excellence for Rehabilitation Medicine, UMC Utrecht Brain Center and De Hoogstraat Rehabilitation, 3583 TM Utrecht, The Netherlands

**Keywords:** cardiovascular diseases, stroke, sedentary behavior, sedentary time, sitting time, physical activity, movement behaviors, behavior change

## Abstract

**Objective:** High amounts of sedentary behavior increase the risk of cardiovascular disease. This study aimed to determine the preliminary effectiveness and feasibility of the RISE intervention to support community-dwelling people with stroke, who are highly sedentary, to reduce and interrupt sedentary time. Additionally, the added value of including participatory support was determined. **Methods:** A randomized, multiple-baseline study was conducted including 14 participants. All received the RISE intervention, a 15-week blended behavioral intervention in which a primary care physiotherapist provided personalized coaching in the home setting by using behavior-change techniques and the RISE eCoaching system, including an activity monitor and app to provide real time feedback. Half of the participants (randomly allocated) received participatory support from someone from their social network (e.g., partner or close friend) who joined them in the intervention. Preliminary effectiveness was determined with significant changes in total sedentary time and fragmentation (interruption) of sedentary time using a randomization test. Feasibility was assessed by adherence with the intervention protocol, safety, and satisfaction with the intervention. **Results:** Participants significantly reduced total sedentary time (*p* = 0.01) by 1.3 h on average and increased their fragmentation (*p* < 0.01). Subgroup analyses showed significant improvements in both outcomes only in the group with participatory support. Thirteen (92.9%) participants completed the intervention, no related adverse events occurred, and the reported participant satisfaction was sufficient. **Conclusions:** The RISE intervention appears promising to support people with stroke who are highly sedentary to reduce and interrupt their sedentary time. Participatory support appears to contribute to greater results. **Trial registration**: ISRCTN international trial registry, 10694741.

## 1. Introduction

Each year, about 43,000 people have a stroke in the Netherlands [1]. Despite significant improvements in acute care, the risk of recurrent stroke is high [2,3,4]. Consequently, secondary prevention is important for people with a stroke. Several risk factors for cardiovascular disease and stroke are known. Relevant risk factors include elevated systolic blood pressure, high body mass index, high fasting glucose and lifestyle factors including physical inactivity and sedentary behavior [5,6,7,8,9,10,11]. Sedentary behavior is defined as “any waking behavior characterized by an energy expenditure ≤ 1.5 metabolic equivalent of task while in a sitting, reclining, or lying posture” [12,13]. Large observational studies report that higher levels of total physical activity at any intensity and less time spent sedentary are associated with a substantially reduced risk for recurrent cardiovascular events and/or mortality [6]. Furthermore, the risk from sedentary behavior increases when sedentary time is accumulated in prolonged bouts [14,15,16,17]. The Breaking Up Sitting Time after Stroke study found that when sedentary time is interrupted by short bouts of standing exercises or walking, systolic blood pressure reduced in people with stroke, even when participants were taking anti-hypertensive medication [18]. High (systolic) blood pressure is the greatest modifiable risk factor contributing to first and recurrent stroke [19].

A previous study measured the movement behaviors of 190 people after stroke who had returned home. The results showed that 79% of the population was highly sedentary (over 9.5 h with 13.5 h of activity monitor-wear time) and spent minimal time engaged in moderate to vigorous physical activity (MVPA) [20]. Of these participants, 31% accumulated their sedentary time in prolonged bouts [20]. These results indicate that over three-quarters of the people with stroke have a movement-behavior pattern that may increase their risk of recurrent stroke and other cardiovascular events.

No effective interventions are known to support people living in the community who have had a stroke to reduce sedentary behavior [21]. Intervention development to reduce sedentary behavior should target supporting behavior change and self-management [21]. Qualitative and quantitative studies indicate a need to focus on people’s awareness of their movement behavior and health consequences and to support people to consciously regulate their movement behavior [20,22,23,24,25,26,27]. Furthermore, factors related to the social and physical environment that influence movement behavior (such as commonly sitting while entertaining visitors and chores around the house) and other individual factors like stroke sequelae and self-efficacy need consideration [20,22,23,24,25,26,27]. Since these factors can vary across people with stroke, the ability to tailor the intervention to a person’s individual needs is required [20,22,23,24,25,26,27].

We developed the RISE intervention (Reduce and Interrupt Sedentary behavior using a blended behavior intervention to Empower people at risk towards sustainable movement-behavior change) to support highly sedentary people with stroke [28]. The RISE intervention aims to reduce and interrupt sedentary behavior by replacing it with physical activity. Sustainable movement-behavior change can be challenging [21], so within the co-design process to develop a personalized intervention, we considered all identified influencing factors from the behavior domains (capabilities, opportunities, and motivation) and accounted for the different phases of change [27,28]. Social support and the social environment were identified as key elements for an effective intervention to reduce sedentary time in the literature [25,27,28]. Therefore, participatory support, where a member of the participant’s immediate social environment participates as a participatory support person in the intervention, may contribute to adherence and improved movement behavior [29,30]. The added value of participatory support and the feasibility of integrating this within movement-behavioral change interventions is currently unknown.

Therefore the objective of this study was to determine the preliminary effectiveness and feasibility of the RISE intervention to support community-dwelling people with stroke, who are highly sedentary, to reduce total sedentary time and interrupt sedentary time. Additionally, the added value of including participatory support within the RISE intervention was determined.

## 2. Method

### 2.1. Design

A randomized, multiple baseline design was used [31,32,33,34,35,36]. This study was conducted according to the Consolidated Standard of Reporting Trials (CONSORT) 2010 statement, extended with reporting N-of-1 trials (CENT) [37]. Within multiple-baseline designs, for each participant, the movement-behavior outcome variables were measured repeatedly in each of the phases (baseline phase, intervention phase, and follow-up phase). The duration of the baseline measures was randomized for each participant. By applying multiple baselines of varying length, the observed effects of the treatment could be distinguished from effects due to chance [31,32,33,34,35,36,38]. This method was conducted for two groups: one receiving the RISE intervention alone (15 weeks) and a second group that had additional participatory support. Participants were randomly allocated by an independent researcher using a computer-generated random sequence table. The study was approved by the ethics review board of the University Utrecht, number ABR NL73036.041.20, METC 20/250. The trial protocol was registered at ISRCTN international trial registry (10694741). The funders played no role in the design, conduct, or reporting of this study.

### 2.2. Participants

Participants were recruited via the stroke units of four hospitals in the Netherlands (regions of Utrecht and Eindhoven), between September 2020 and December 2021. Informed consent was obtained from each participant who was willing to participate and eligible. The eligibility criteria were as follows:

#### 2.2.1. Inclusion Criteria

1. Aged > 18 years;
2. Stroke diagnosed in hospital in previous six months and discharged to home setting;
3. Able to walk independently (functional ambulation category score ≥ 3) [39];
4. Sedentary movement-behavior pattern, i.e., ≥9.5 h of sedentary time per day and meeting at least one of the following criteria: >50% of the sedentary time is spent in bouts > 30 min and/or not reaching the physical activity guideline (150 min MVPA during the week) [20]. This was determined by wearing the activ8, a thigh-worn activity monitor that determines movement behavior during waking hours for one week;
5. Independent in activities of daily living pre-stroke (Barthel Index score > 18 [40]);
6. Have someone who could participate as a participatory support person in the RISE intervention with participatory support.

#### 2.2.2. Exclusion Criteria

1. Insufficient knowledge of the Dutch language to understand the intervention content;
2. Score < 4 on the Utrecht Communication Assessment, which assesses speech and conversation abilities on a 5-point scale, with a score of 5 being independent in conversations regarding multiple topics;
3. Severe comorbidities that prevent that person from safely reducing and interrupting their sedentary time (e.g., severe pulmonary diseases, heart failure, or malignities), determined with the Physical Activity Readiness Questionnaire [41];
4. Receiving physiotherapy in any other setting than primary care.

The participatory support person of the participant with stroke had to be part of the participant’s immediate social environment (e.g., partner or close friend), meaning someone who has regular social interactions with the participant, i.e., at least two contact moments a week. They had to meet inclusion criteria 1 and 3 and were excluded based on exclusion criteria 1 and 3.

### 2.3. Sample Size

The sample size was based on the incorporation of a randomization test to assess the preliminary effectiveness regarding sedentary behavior with sufficient power fitting for a multiple-baseline design [31,32,33,36]. Randomization was conducted based on the concealed allocation principle using the Wamplod and Worsham method, in which power is determined by the relationship between the number of possible intervention starting points for each participant and the number of participants [31,32,33,36]. Participants were randomized to a baseline measurement duration of either 4, 6, 8, 10, 12, or 14 days. Based on these six randomization options, a sufficient number of permutations was achieved to enable the analyses (minimal *p*-value < 0.01) to determine significant changes [31,32,33,36]; thus, six participants per group would be sufficient. To ensure that dropout (for reasons not related to the intervention) after recruitment was finished did not affect the rigor of the data analyses, one additional participant per group was included, so fourteen participants in total were included.

### 2.4. Intervention

**Figure 1:**
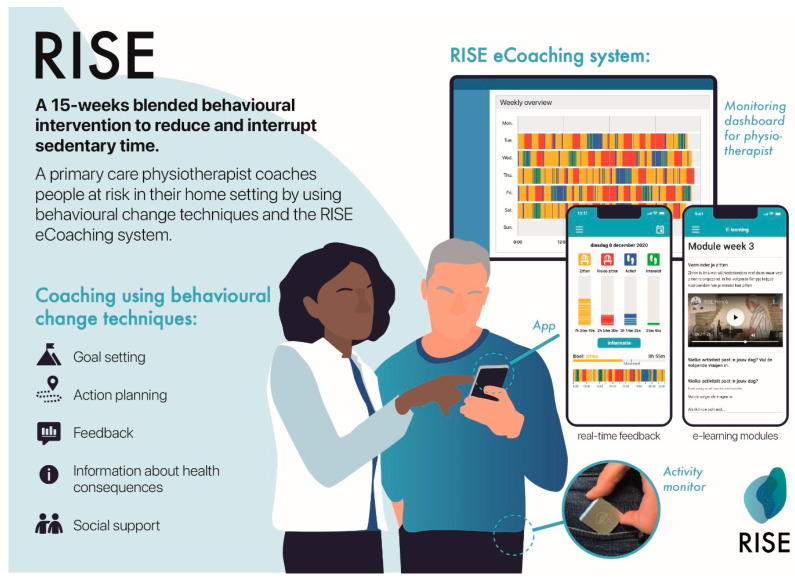
RISE intervention.

Participants received the RISE intervention (see Figure 1), a 15-week blended behavioral intervention in which a primary care physiotherapist coached participants to reduce and interrupt their sedentary time. Physiotherapists provided personalized coaching to people with a first-ever stroke in their home setting by using behavior-change techniques and the RISE eCoaching system. The RISE eCoaching system consists of (1) an activity monitor, (2) a smartphone application that provides real-time feedback and contains e-learning modules, and (3) a monitoring dashboard for the physiotherapist. Participants received participatory support from someone from their social network (e.g., partner or close friend) who joined them in the intervention.

The coaching sessions included (among other aspects) discussion of movement behaviors and activities and identifying possibilities for change. Goals were set, and action plans were made. In between the coaching sessions, real-time feedback on movement behavior was provided by using the RISE eCoaching system, and eLearning modules were available for the participant to undertake. These eLearning modules included information on subjects such as stroke, healthy movement behavior, and behavior change. Supplementary File S1—“RISE intervention details” provides detailed information about the weekly intervention schedule.

The following behavior-change techniques were at the core of this blended intervention: goal-setting (on behavior and outcome), action planning, social support, self-monitoring on behavior, feedback on behavior, the discrepancy between current behavior and goal, information about health consequences, problem solving, restructuring the social environment, prompts and cues, habits formation, and instructions how to perform the behavior. The RISE eCoaching system used the activ8 activity monitor, a reliable and valid tool to determine movement behavior [42,43]. The intervention was delivered in the participants’ home by four primary care physiotherapists who all received training to provide the RISE intervention. The training included subjects such as healthy movement behavior, behavior change, and coaching on the job.

The content of the RISE intervention was identical for participants with and without participatory support. The only difference was that those with participatory support had their participatory support person present at the face-to-face sessions. Participatory support persons also received the RISE monitor with the app to gain insight and received information regarding healthy movement behavior and how to provide meaningful support.

### 2.5. Outcome Measures

Demographic and stroke-related data were obtained from the medical file and a baseline questionnaire. The preliminary effectiveness of the intervention on sedentary behavior and the added value of participatory support were assessed using the total amount of sedentary time (in hours) and the sedentary time interruption, using the fragmentation index. Sedentary behavior was measured with the ActivPAL activity monitor. This monitor (PAL Technologies Ltd., Glasgow, UK) is reliable (intraclass correlation coefficient 0.79–0.99) and valid (98–100% accuracy) for measuring movement behavior during daily life in people with stroke [44,45,46]. Participants were asked to keep a diary to keep track of the time they got up out of bed in the morning and time they went to sleep at night, and this information was used to determine waking hours.

Feasibility was assessed by measures of adherence with the intervention protocol, safety, and satisfaction. Adherence and safety were determined by measuring (1) the number of people that completed the intervention; (2) the number of participants that missed one or more of the face-to-face sessions and the reasons for missing sessions; (3) the number of adverse events. Participants’ satisfaction with the RISE system was determined using the System Usability Scale (SUS) questionnaire. The SUS is a valid and reliable instrument to measure participants’ perceived satisfaction [47]. Scores range from 0–100, with a score of 70–80 representing medium satisfaction and a score over >80 high satisfaction [47].

Secondary outcomes included the amount of light physical activity (LPA) (hours) and moderate to vigorous physical activity (MVPA) (minutes) per day, measured with the ActivPAL activity monitor, and was used to determine what sedentary behavior was replaced with if a reduction occurred. Other physical activity outcomes, additional sedentary outcomes such as the percentage of waking hours spent sedentary, and sleep time are presented in Supplementary File S2, “Data visualization”.

### 2.6. Data Analyses

All analyses were conducted with R statistical software, version 3.6.1. The ActivPAL data were downloaded from the device using the manufacturer’s provided software. A Knitter program was used to combine the repeated measurements into one dataset for each participant [48]. The ProcessingPAL software V1.3 was used to determine waking hours (in combination with diaries) and extract the relevant outcome variables [49,50]. All available data for any participant that dropped out were included in the analyses following the intention-to-treat principles.

To determine the intervention’s preliminary effectiveness, both group and individual participant analyses were conducted. The Wampold and Worsham randomization test [31,32,33,36,51,52], was used to determine if there was a statistically significant change at group level. The null hypothesis was that there was no effect of the intervention, i.e., no difference between the baseline and post-intervention period in sedentary time or fragmentation. We used a two-tailed alternative hypothesis, with a significance level set at α = 0.05.

The data per participant were graphed and visually assessed to yield an indication of any differences in the level, trend, variability, overlap, or consistency of the data over time [53,54,55]. The addition of a mean and 2-SD band (standard deviation) was used to support the visual analyses [31,32,33]. The non-parametric effect size was then determined using the percentage exceeding the median (PEM), which is the best fit when there is larger variability in baseline data [53,54,56,57]. The PEM represents the percentage of days during and after the intervention in which there was an improvement compared to the baseline median. For the PEM, >90% is considered a high effect, 70–90% moderate, 60–70% mild, 50–60% questionable, and <50% no effect [57].

To determine if there was any added value of participatory support, the above analyses were conducted separately for the group with and without added participatory support. The visual and PEM analyses were also used in the secondary analyses to indicate what type of physical activity participants used to replace their sedentary time.

Feasibility outcome variables are reported as total numbers and/or percentages. The reasons for drop out or any missing appointments are listed. The SUS score was calculated per participant; then, the mean score and standard deviation were calculated.

## 3. Results

**Figure 2:**
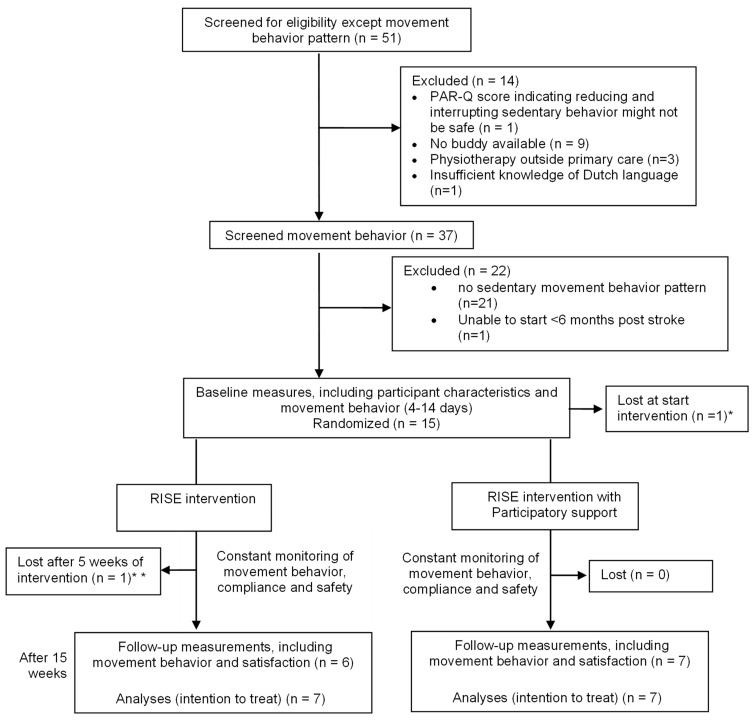
Flow of participants through the trial, including measurements conducted. Note: * Participant did not like measuring device; ** reason for drop-out: “no longer wanted to feel like a patient”.

Fifty-one potential participants were screened for participation in the study, and fourteen participants were included (see Figure 2). Four participants were female (28.6%), and the median age was 66.5 (49–78). Other participant characteristics can be found in Table 1. At baseline, the average time spent sedentary during waking hours was 11.4 (SD 1.1, range 10.0–13.7) hours. One participant from the group without participatory support dropped out after 5 weeks.

### 3.1. Preliminary Effectiveness

**Figure 3:**
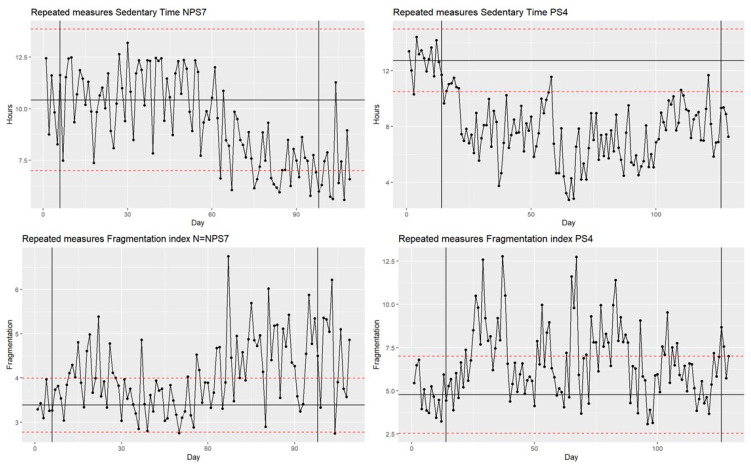
Examples of visualization of movement behavior. Note: PS: participatory support; NPS: group without participatory support. The black vertical lines indicate the start and stop of the intervention. The black horizontal line represents the mean from the baseline measurements. The red dashed horizontal lines indicate the two standard deviation bands from the baseline measurement. In total sedentary time, a downward trend indicates a reduction in sedentary time. An upwards trend in fragmentation indicates an increase in the interruption of sedentary time.

The randomization test showed a significant change in both total sedentary time during waking hours (*p* = 0.01) and in the fragmentation of sedentary time (*p* < 0.01) for the overall group (all 14 participants). In individual-level analyses, the PEM showed (score > 60%, Table 2) that 12 participants (85.7%) improved on at least one outcome of sedentary behavior, and 7 (50.0%) improved on both outcomes. Examples of the visualization of the data can be found in Figure 3, and all visuals are included in Supplementary File S2.

Eleven out of fourteen participants showed a change in total sedentary time; the reduction ranged from 0.1 to 5.2 h, with an average of 1.3 h (SD 1.4). Eight (72.7%) of these eleven participants had a reduction of sedentary time that exceeded 30 min, and seven (63.6%) reduced sedentary time by more than 60 min. The fragmentation index score increases (8 out of 14 participants) ranged from 0.6 to 2.0 with an average change of 1.1 (SD 0.5).

When we looked at the groups with and without participatory support separately, there was a significant change in total sedentary time (*p* = 0.03) with the addition of participatory support. In this group, there was also a significant change in the fragmentation of sedentary time (*p* = 0.03). In the group without participatory support, only the change in the fragmentation was significant (*p* = 0.04); the change in total sedentary time was not (*p* = 0.11).

The PEM indicated that all participants in the group with participatory support improved (PEM score > 60%, see Table 2) on at least one sedentary behavior outcome, and five (71.4%) of the participants improved on both. By comparison, only five (71.4%) and two (28.6%) participants in the group without participatory support improved on these metrics, respectively.

### 3.2. Feasibility

Thirteen participants (92.9%) completed the intervention, including all face-to-face sessions, and used the RISE eCoaching system. One participant dropped out after 5 weeks citing “not wanting to be monitored”. Three adverse events were registered during the intervention period (a hospitalization due to an abdominal infections and two TIAs), though none were related to the intervention.

Participants’ SUS questionnaire scores ranged from 60 to 92.5, with an average score of 73 points (SD 13.8), indicating a medium level of satisfaction with the e-health component of the intervention.

### 3.3. Secondary Outcomes

The PEM scores for physical activities were calculated (see Supplementary File S2, Table PEM Physical activity). Forty-five percent of the participants replaced their sedentary behavior with both light and moderate to vigorous physical activity. A further 45.4% only increased their time spent in light physical activity, and 9.1% only increased their time spent in moderate to vigorous activity.

## 4. Discussion

This study provides initial proof of concept that the RISE intervention may be effective in supporting people with stroke who are highly sedentary to reduce and interrupt their sedentary time. Our preliminary effectiveness analyses showed significant positive effects on total sedentary time and the fragmentation of sedentary time. The intervention also appears feasible, with 13 (92.9%) participants completing the intervention and no intervention-related adverse events. Participants reported sufficient satisfaction with the intervention. Furthermore, the results of this study suggests the potential added value of integrating participatory support in the RISE intervention.

The changes seen in total sedentary time in our study (average 1.3 h) may be clinically meaningful. There is no current consensus about the magnitude of reduction in sedentary time needed for a clinically meaningful effect; however, several studies [21,58] have reported a dose–response relationship between higher amounts of sedentary time and greater health risks [5,6,58,59,60,61]. Increases of 30–60 min in sedentary time have been shown to be associated with all-cause mortality including stroke (hazard ratio [HR] 0.20–0.46 and HR 0.47–0.85 for 30 and 60 min increases in daily sedentary time, respectively) [6]. Similarly, others have reported a significant risk increase in cardiovascular mortality of 1.04 (95% CI 1.03, 1.04, *p* < 0.001) for each additional sedentary hour in individuals who were sedentary for >6 h per day [60].

Several studies have demonstrated an association between interrupting sedentary time and a reduction in health risk factors in both laboratory-based and free-living studies, although no clear dose–response relationship has been identified [14,15,16,17,18,59]. These studies indicate that the average increased interruption observed in our study, 1.1 points on the fragmentation index (about 10 additional interruptions for an average of 10 h sitting), may be clinically relevant when it comes to cardiovascular disease risk [14,15,16,17,18,59].

It is also important to consider the magnitude of change we found with the minimal detectable change (MDC) for the measures used. The MDC for total sedentary time was determined at around 30 min for older adults and about 60 min for office workers based on an average 16 h wake time measured using the ActivPAL [62]. With an average change of 1.3 h in our study, there appears to be a real change. The MDC for the fragmentation index was 1.2 for both older adults and office workers [62].

Studies of interventions aiming to reduce sedentary behavior in other cardiovascular disease populations have reported small and non-significant effects. A recent randomized controlled study in a cardiac rehabilitation population showed a decrease in sedentary behavior in the intervention group, though there was no significant difference between intervention and control group [63]. Another recent pilot study in a cardiac population targeting moving more and sitting less only found a significant increase in daily steps, as the time spent sedentary did not change [64]. This is consistent with recent reviews in the area of cardiovascular disease that conclude the need for further research due to the lack of evidence [65,66]. Similar conclusions can be found in a Cochrane review in community-dwelling older adults [67]. Our study shows promising results, which could be related to the behavioral approach and the blended nature of the intervention combining face-to-face coaching with e-health by means of the RISE system that enabled real-time feedback on movement-behavior patterns. Thereby, the intervention enabled people with stroke to actually take action for themselves to reduce their risk of recurrent cardiovascular events. This kind of empowerment was deemed highly relevant to create motivation regarding behavior change. Another novelty in our study was the inclusion of extensive social support by means of participatory support. However, a randomized controlled trial including a longer follow-up is needed to confirm the efficacy of the RISE intervention

When it comes to the addition of participatory support to the RISE intervention, our results suggest this form of social support may provide added value. In six cases, a spouse acted as the participatory support person, and in one it was a daughter; all completed the intervention period. These results match the insights from other studies that found that the direct social environment is important when it comes to influencing movement behavior [25,27,29,30,68]. Participatory support appears to influence both daily habits and routines, and it seems capable of providing meaningful support to change behavior [25,29,30,68].

Our results suggests that a blended intervention delivered by a physiotherapist and combining coaching with an e-health system and including several behavior-change techniques seems promising to change sedentary behavior. This is consistent with recent studies that show an increase in the use of applications integrated with behavior-change techniques, which are promising to improve movement behavior [65,66,68]. However, no clear recommendations could be made regarding which techniques are effective to improve sedentary behavior [65,66,68]. Our study could be a first step to fill this gap in knowledge. A randomized controlled trail including a long-term follow-up is needed to draw definitive conclusions.

### Strengths and Limitations

This study had a number of strengths. Firstly, using the multiple-baseline design enabled us to give a well-supported preliminary estimation of the effect of the RISE intervention with a minimal burden and small sample size [69,70]. Secondly, the combination of the highly reliable and valid activity monitor [44,45,46] to measure movement behavior and the use of the ProcessingPAL software together with the diaries to determine wake time contributed to the rigor of the data collection and subsequent validity of the results. Thirdly, the intervention was delivered by physiotherapists specifically trained to provide the RISE intervention. Still, the physiotherapist afterwards stated they feel they need an even higher skill level to optimally support movement-behavior change.

Our study had some limitations. We experienced some technical difficulties with the use of the RISE monitor and app, leading to the slow loading of movement-behavior data. Though this was improved during the study, this may have negatively influenced our results in terms of satisfaction with the intervention. In addition, large variability was seen in the sedentary behavior data. Although the minimal baseline measurement duration used (4 days) was in line with previous research to determine an accurate average, this may have affected the results. However, this limitation was mitigated by the use of analysis strategies that best suited datasets with large variability. Seventy percent of our sample was male, which limits generalizability. Lastly, though the randomized multiple baseline design accounts for within-person variability, our sample size was still small and therefore more sensitive to the influence of any extraordinary occurrences within the setting of the participants.

## 5. Conclusions

The RISE intervention appears promising to support people with stroke who are highly sedentary to reduce and interrupt their sedentary time. Participatory support provided by someone from their social network (e.g., partner or close friend) who joined the participant in the RISE intervention appeared to contribute to greater results. Our results show the potential of blended behavioral interventions for supporting movement-behavior change. A randomized controlled trial including a longer follow-up is needed to confirm the efficacy of the RISE intervention.

## Figures and Tables

**Table 1:** Participant characteristics.

Characteristic	Complete Sample (n = 14)	Group with PS (n = 7)	Group without PS (n = 7)
Age (years), median (range)	66.5 (49–78)	68 (49–71)	65 (55–78)
Gender, number female (%)	4 (28.5)	1 (14.3)	3 (42.9)
Education level, number (%)			
Low	3 (21.4)	2 (28.6)	1 (14.3)
Medium	6 (42.9)	3 (42.9)	3 (42.9)
High	5 (35.7)	2 (28.6)	3 (42.9)
Comorbidities, number ≥ 2 (%, range)	11 (78.6, 0–4)	5 (71.4, 0–4)	6 (85.7, 1–4)
Living with spouse, number yes (%)	11 (78.6)	6 (85.7)	5 (71.4)
Smoking number (%)			
Current	3 (21.4)	2 (28.6)	1 (14.3)
Previous	8 (57.1)	4 (57.1)	4 (57.1)
Alcohol consumption, number one or more per day (%)	6 (42.9)	2 (28.6)	4 (57.1)
Type of stroke, number infarct (%)	14 (100)	7 (100)	7 (100)
Side of stroke, number right side (%)	3 (21.4) *	2 (28.6)	1 (14.3) *
Stroke severity (NIHSS at time of hospital admission), number ≤ 4 (%, range)	12 (92, 0–8) **	6 (100, 0–4) **	6 (86.7, 0–8)
Stroke impact (SIS physical), median (range)	88 (62–99)	86 (62–89)	89 (65–99)
Recovered (VAS)	79 (55–100)	78 (56–100)	80 (55–99)
Walking speed, number full community walkers (>0.93 m/s) vs. limited community walkers (0.40–0.93 m/s) (%) ***	9 (64.3)	6 (85.7)	3 (42.9)
General disability (mRS), number ≤ 1 (%, range)	11 (78.6, 0–2)	5 (71.4, 0–2)	6 (86.7, 0–2)
Cognitive impaired (MoCA < 26), number (%)	7 (50.0)	4 (57.1)	3 (42.9)

SD: standard deviation; NIHSS: National Institutes of Health Stroke Scale; SIS: Stroke Impact Scale; ADL: activities of daily living; mRS: modified Rankin Scale; MoCA: Montreal Cognitive Assessment, Management Scale. * One participant had a centrally located stroke. ** NIHSS was not in the medical record of one participant. *** All participants were able to walk independently within the community.

**Table 2:** PEM and mean scores (SD).

**With Participatory Support**	**Sedentary Time (h)**			Fragmentation Index *		
Participant	Percentage Exceeding Baseline Median (PEM, %)	Mean [SD] Phase A Phase B+A′	Difference Phase A Phase B+A′	Percentage Exceeding Baseline Median (PEM, %)	Mean [SD] Phase A Phase B+A′	Difference Phase A Phase B+A′
**1**	**68.6%**	10.3 (1.6) 10.1 (1.3)	**0.2**	**64.7%**	3.9 (1.1) 4.9 (1.9)	**1**
**2**	56.8%	11.8 (0.6) 11.7 (1.0)	0.1	**76.3%**	2.8 (0.4) 3.4 (0.7)	**0.6**
**3**	**60.8%**	10.1 (0.9) 10.0 (1.0)	**0.1**	58.8%	7.2 (1.6) 7.5 (1.8)	0.3
**4**	**100.0%**	12.7 (1.1) 7.5 (2.0)	**5.2**	**87.0%**	4.8 (1.1) 6.8 (2.1)	**2**
**5**	**73.1%**	11.4 (0.9) 10.0 (2.0)	**1.4**	**69.7%**	2.6 (0.7) 3.5 (1.9)	**0.9**
**6**	**70.8%**	11.2 (1.9) 9.8 (2.1)	**1.4**	**89.6%**	2.8 (1.3) 4.7 (1.8)	**1.9**
**7**	**87.0%**	12.4 (0.7) 11.1 (1.3)	**1.3**	**87.0%**	3.5 (1.1) 4.2 (0.8)	**0.7**
**Without participatory support**	**Sedentary time (h)**			Fragmentation Index *		
Participant	Percentage Exceeding baseline Median (PEM, %)	mean [SD] phase A phase B+A′	Difference phase A phase B+A′	Percentage Exceeding baseline Median (PEM, %)	mean [SD] phase A phase B+A′	Difference phase A phase B+A′
**1**	**87.0%**	13.7 (0.9) 12.0 (1.2)	**1.7**	46.0%	4.1 (0.7) 4.1 (1.1)	0
**2**	**83.2%**	12.0 (2.3) 10.6 (1.6)	**1.4**	**71.4%**	2.3 (0.5) 3.3 (1.1)	**1.0**
**3**	46.5%	11.0 (1.2) 11.0 (1.7)	0	52.5%	4.3 (1.0) 4.4 (1.5)	0.1
**4**	**64.8%**	11.0 (1.1) 10.9 (1.0)	**0.1**	23.8%	4.0 (1.0) 3.3 (0.7)	−0.7
**5**	**67.6%**	10.0 (1.7) 9.2 (2.2)	**0.8**	54.3%	5.6 (0.9) 5.9 (2.2)	0.3
6 **	22.2%	10.9 (1.4) 11.0 (0.9)	−0.1	58.3%	6.0 (1.8) 5.7 (1.2)	−0.3
**7**	**69.9%**	10.4 (1.7) 9.2 (2.2)	**1.2**	**83.5%**	3.4 (0.3) 4.1 (0.8)	**0.7**

PEM: percentage exceeding the median; represents the percentage of days during and after the intervention in which there was an improvement compared to the baseline median. h: hours; Phase A: baseline phase; Phase B: intervention phase; Phase A′: post-intervention phase; SD: standard deviation; Bold: effect of either high (>90), moderate (70–90), or mild (60–70); Non-bold: questionable effect (50–60) or no effect (<50). * A higher fragmentation index means more interruption of sedentary behavior; ** Pt who dropped out early.

## Data Availability

The data presented in this study are available on request from the corresponding author due to the fact that the datasets are still being used for secondary analyses.

## Supplementary Materials

The following supporting information can be downloaded at: https://www.mdpi.com/article/10.3390/jcm13154341/s1, File S1: RISE intervention details; File S2: Data visualization and Table PEM Physical activity.

## Trial Registration

ISRCTN international trial registry, 10694741.
