# Supplementary material for: Improving Movement Behavior in People after Stroke with the RISE Intervention: A Randomized Multiple Baseline Study
Source: J Clin Med. 2024 Jul 25;13(15):4341. doi: 10.3390/jcm13154341 (PMC11313465; doi:10.3390/jcm13154341)
Supplement: Supplementary file 1 [file jcm-13-04341-s001.zip › Supplementary Files 1 RISE intervention details_masked.pdf]

Supplemental Digital Content 1 RISE intervention details

Title: *Improving movement behavior after stroke with RISE – a randomised multiple baseline study*

RISE intervention details

Weeks	Topic	Phase	Intervention function	Behavior change techniques
**1. Face-to-face:**<br><br>**Introduction of the intervention**	• Intake<br>• Information about the intervention<br>• Start self-monitoring	Motivating stage (preintender)	Education	
**1b. MHealth application:**<br><br>**The added value of reducing sedentary behavior and improving physical activity**	• Introduction to health consequences of lifestyle and movement behavior<br>o What is a stroke<br>o What is prevention<br>o What is exercise behavior	Motivating stage (preintender)<br><br>Risk perception	Education<br>Persuasion	• Monitoring of behavior by others without feedback<br>• Information about health consequences<br>• Salience of consequences<br>• Information about emotional consequences<br>• Pros and cons<br>• Feedback on behavior
**2. Face-to-face:**<br><br>**Movement Behavioral diagnosis**	• Motivational interviewing to identify barriers and facilitators<br>• Discuss movement behavior	Motivating stage (preintender)<br><br>Task self-efficacy	Education<br>Persuasion<br>Incentivization	• Feedback on behavior<br>• Goal setting behavior<br>• Discrepancy between current behavior and goal<br>• Graded task<br>• Non-specific reward<br>• Problem-solving
**2b. MHealth application:**<br><br>**Added-value of sitting less and moving more**	• Self-monitoring: an introduction<br>• What is movement behavior part II	Motivating stage (preintender)<br><br>Risk perception	Education<br>Persuasion<br>Training<br>Incentivization	• Feedback on behavior<br>• Information about health consequences<br>• Information about emotional consequences<br>• Verbal persuasion about capability<br>• Credible sources<br>• Graded task

				• Goal setting behavior<br>• Instruction on how to perform the behavior<br>• Non-specific reward<br>• Discrepancy between goal and current behavior
**3. Face-to-face:**<br><br>**Evaluate movement behavior**	• Discuss movement behavior<br>• Discuss self-beliefs about reducing sedentary behavior<br>• What are possible barriers<br>• Enable cues	Motivating stage (preintender)<br><br>Outcome expectancies<br>Task self-efficacy	Incentivization<br>Training	• Feedback on behavior<br>• Social reward<br>• Behavior substitution<br>• Habit reversal<br>• Graded task<br>• Goal setting behavior<br>• valued self-identify<br>• Non-specific reward<br>• Verbal persuasion about capability<br>• Problem-solving<br>• Review behavioral goals
**3a. MHealth application:**<br><br>**Your daily activities**	• Self monitoring<br>• Insight in behavior<br>• Which activities to implement	Intention<br><br>Environmental barriers	Training<br>Enablement<br>Education	• Feedback on behavior<br>• Verbal persuasion about capability<br>• Instruction on how to perform a behavior<br>• demonstration of the behavior<br>• Graded task<br>• Goal setting behavior<br>• Non-specific reward<br>• Problem-solving<br>• Discrepancy between goal and current behavior
**4. Face-to-face:**<br><br>**Action planning and goal setting**	• Goal setting<br>• Self-monitoring<br>• Strategies to sit less	Volitional stage (intender)<br><br>Intention and planning	Environmental restructuring<br>Training<br>Education<br>Incentivization	• Feedback on behavior<br>• Verbal persuasion about capability<br>• Graded task<br>• Action planning<br>• Demonstration of the behavior<br>• Instruction on how to perform a behavior<br>• Prompts and cues<br>• Habit formation

				• Habit reversal<br>• Goal setting behavior<br>• Non-specific reward<br>• Problem-solving<br>• Review behavioral goals
**4a. MHealth application:**<br><br>**Trying prompts and cues**	• Self-monitoring<br>• Social support	Intention and planning<br><br>Environmental barriers and resources	Training<br>Enablement<br>Education	• Feedback on behavior<br>• Prompts and cues<br>• Graded task<br>• Action planning<br>• Problem-solving<br>• Non-specific reward<br>• Discrepancy between goal and current behavior
**5. Face-to-face:**<br>**Social support / social environment**	• Discuss movement behavior<br>• Action planning<br>• Discuss the opportunity for social support	Volitional stage (intender/actor)<br><br>Action<br>Task self-efficacy	Enablement<br>Training<br>Incentivization<br>Persuasion	• Feedback on behavior<br>• Focus on past success<br>• Social support (unspecified)<br>• Social support practical<br>• Review behavioral goals<br>• Problem-solving<br>• Habit reversal<br>• Habit formation<br>• Graded task<br>• Goal setting behavior<br>• Restructuring the physical environment<br>• Non-specific reward
**5a. MHealth application:**<br><br>**What are habits?**	• Self-monitoring<br>• What are habits<br>• Celebrating success	Maintenance task self-efficacy<br>Action	Education<br>Persuasion	• Feedback on behavior<br>• Prompts and cues<br>• Graded task<br>• Habit reversal<br>• Generalization of the target behavior<br>• Non-specific reward<br>• Problem-solving

				• Discrepancy between goal and current behavior
**6. Face-to-face:**<br><br>**Self-efficacy to interrupt sedentary behavior**	• Review current behavior<br>• Provide positive feedback<br>• Celebrate success<br>• Discuss action planning<br>• Discuss social support / social environment<br>• Decrease cues	Volitional stage (intender)<br><br>Maintenance task self-efficacy<br>Action	Persuasion<br>Education<br>Incentivization	• Feedback on behavior<br>• Feedback on outcome<br>• Verbal persuasion about capability<br>• Focusing on past success<br>• Review behavioral goals<br>• Graded task<br>• Goal setting behavior<br>• Feedback on outcome<br>• Social support (unspecified)<br>• Non-specific reward<br>• Problem-solving
**6a. MHealth application:**<br><br>**New possibilities**<br><br>**7a. MHealth application: adjust physical environment**	• Discuss new ideas/possibilities to interrupt sedentary behavior<br><br>• Discuss physical environment and possible changes<br>• Discuss set-backs	Volitional stage (intender/actor)<br>Maintenance self-efficacy<br>Action	Education<br>Persuasion<br>Training<br>Environmental restructuring<br>Enablement	• Feedback on behavior<br>• Graded task<br>• Goal setting behavior<br>• Behavior substitution<br>• Discrepancy between goal and current behavior<br>• Restructuring the physical environment<br>• Adding objects to the environment<br>• Graded task<br>• Goal setting behavior<br>• Problem-solving<br>• Non-specific reward
**8. Face-to-face:**<br><br>**Introduction set-backs**	• Discuss progress<br>• Celebrate success<br>• Discuss action plan<br>• Discuss an action plan to change the physical environment	Volitional stage (intender/actor)<br><br>Maintenance self-efficacy<br>Action<br>Recovery self-efficacy	Persuasion<br>Education<br>Environmental restructuring	• Feedback on behavior<br>• Pros and cons<br>• Graded task<br>• Focusing on past success<br>• Review behavioral goals<br>• Habit formation<br>• Goal setting behavior<br>• Social support (unspecified)<br>• Restructuring the physical environment

				• Problem-solving<br>• Non-specific reward
**8a. MHealth application:**<br><br>**Sedentary habits**<br><br>**9a MHealth application: Belief in own abilities**	• Monitoring movement behavior<br>• Created habits<br><br><br>• Self-belief	Maintenance self-efficacy<br><br>Action<br>Recovery self-efficacy	Training<br>Education	• Feedback on behavior<br>• Graded task<br>• Goal setting behavior<br>• demonstration of the behavior<br>• habit formation<br>• Non-specific reward<br>• Problem-solving<br>• Discrepancy between goal and current behavior
**10. Face-to-face:**<br><br>**Habit formation and setbacks**	• Discuss progress<br>• Feedback on behavior<br>• Reflecting on action plans and adjusting or making a new one<br>• Habits and setbacks<br>• Set performance goal	Volitional stage (actor)<br><br>Maintenance self-efficacy<br>Action<br>Recovery self-efficacy	Training<br>Education<br>Enablement<br>Persuasion	• Feedback on behavior<br>• Review behavioral goals<br>• Habit reversal<br>• Behavior substitution<br>• Graded task<br>• Goal setting behavior<br>• Social support (unspecified)<br>• demonstration of the behavior<br>• verbal persuasion about capability<br>• habit formation<br>• Non-specific reward<br>• Problem-solving
**10a. MHealth application: Celebrate success**<br><br>**11a. MHealth application: Self-monitoring**	• Celebrate success<br><br><br>• Discuss self-monitoring tool or app for the future	Maintenance self-efficacy<br>Action	Education<br>Training<br>Enablement	• Feedback on behavior<br>• Graded task<br>• Goal setting behavior<br>• Instruction on how to perform a behavior<br>• Demonstration of the behavior<br>• Action planning<br>• Problem-solving<br>• Discrepancy between goal and current behavior<br>• Non-specific reward
**12. of 13. Face-to-face:**	• Discuss progress<br>• Feedback on behavior	Volitional stage (actor)	Training<br>Enablement	• Feedback on behavior<br>• Review behavioral goals

**Self-monitoring**	• Reflecting on action plans and adjusting or making a new one<br>• Discuss the pros and cons of sitting<br>• Discuss self-monitoring tool future + action plan to implement	Action<br>Recovery self-efficacy	Incentivisation<br>Persuasion	• Graded task<br>• Goal setting behavior<br>• Social support unspecified<br>• Habit formation<br>• Action planning<br>• Non-specific reward<br>• Problem-solving
**13a. MHealth application:**<br><br>**Are you future proof**	• Questions to prevent setbacks and recover behavior	Maintenance self-efficacy<br>Action<br>Recovery self-efficacy	Persuasion<br>Training	• Feedback on behavior<br>• Graded task<br>• Feedback on behavior<br>• Goal setting behavior<br>• Valued self-identity<br>• Focus on success<br>• Action planning<br>• Problem-solving<br>• Discrepancy between goal and current behavior
**14a. MHealth application:**<br><br>**What did it bring you**	• What did it get you<br>• Questions to PT	Maintenance self-efficacy<br>Action<br>Recovery self-efficacy	Persuasion<br>Incentivization<br>Enablement	• Feedback on behavior<br>• Graded Task<br>• Review behavior goals<br>• Goal setting behavior<br>• Discrepantcy between current behavior and goal<br>• Non-specific reward<br>• Focus on past success
**15. Face-to-face:**<br><br>**Sustainable movement behavior**	• Discuss behavior<br>• Questions?<br>• Future proof action plan to prevent setbacks or recover behavior<br>• Closing	Maintenance self-efficacy<br>Action<br>Recovery self-efficacy	Persuasion<br>Incentivization<br>Enablement	• Feedback on behavior<br>• Feedback on outcome<br>• Review behavioral goals<br>• Habit reversal<br>• Behavior substitution<br>• Graded task<br>• Goal setting behavior<br>• Social support (unspecified)<br>• pros and cons<br>• Commitment<br>• Non-specific reward
