# Supplementary material for: Improving Movement Behavior in People after Stroke with the RISE Intervention: A Randomized Multiple Baseline Study
Source: J Clin Med. 2024 Jul 25;13(15):4341. doi: 10.3390/jcm13154341 (PMC11313465; doi:10.3390/jcm13154341)
Supplement: Supplementary file 1 [file jcm-13-04341-s001.zip › Supplementary Files 2 Data visualisation and Table PEM Physical activity_masked.pdf]

Supplemental Digital Content 2 Data visualisation and Table PEM Physical activity

Title: *Improving movement behavior after stroke with RISE – a randomised multiple baseline study.*

Images repeated measurement data

PS: group with Participatory Support

NPS: group without Participatory Support

The black vertical lines indicate the start and stop of the intervention

The black horizontal line represents the mean from the baseline measurements

The red dashed horizontal lines indicate the 2 standard deviation bands from the baseline measurement.

An upwards trend in fragmentation indicates an increase in the interruption of sedentary time.

NPS6 was the participant that dropped out prior to the end of the intervention protocol.

<u>Total amount of time spend Sedentary</u>

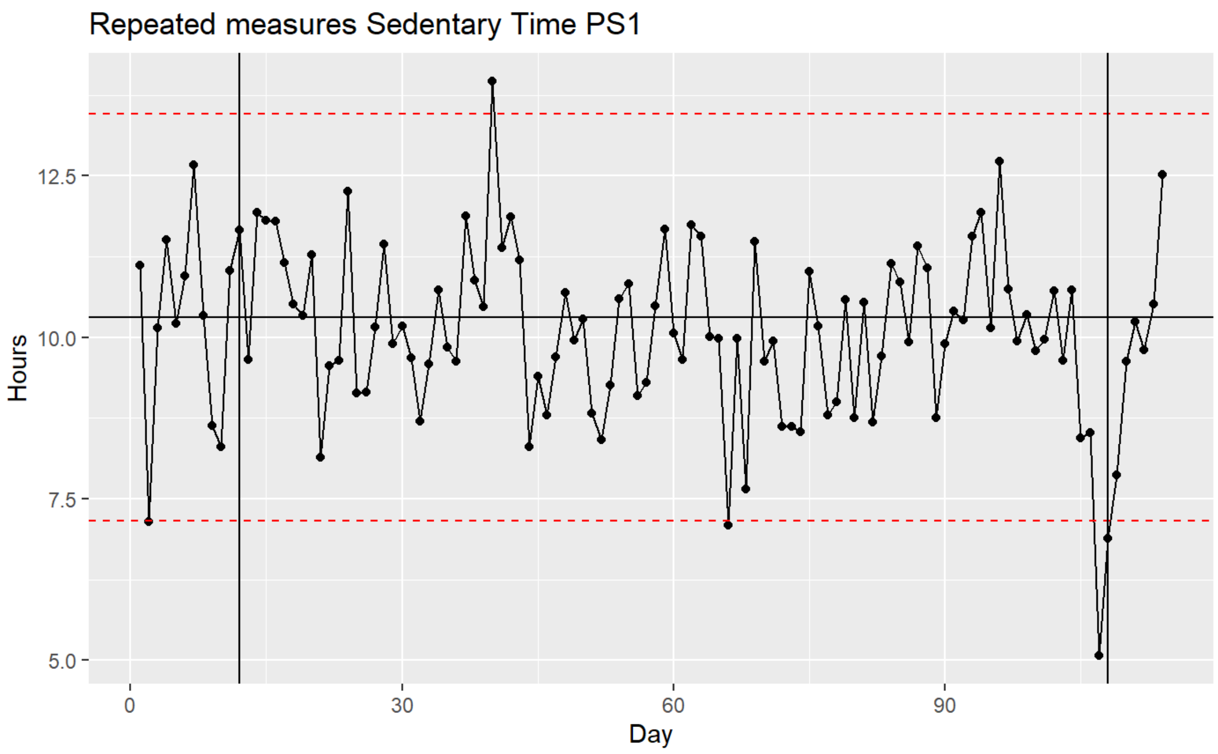

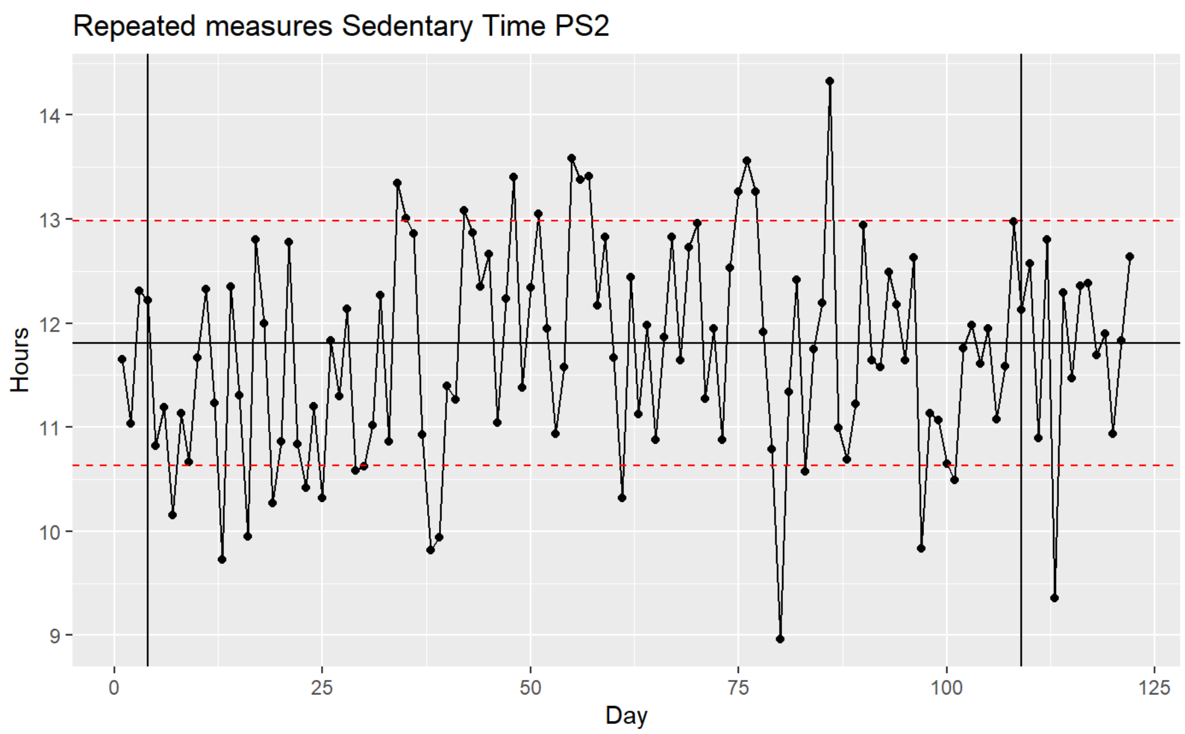

Repeated measures Sedentary Time PS2
Hours
Day
14
13
12
11
10
9
0
25
50
75
100
125

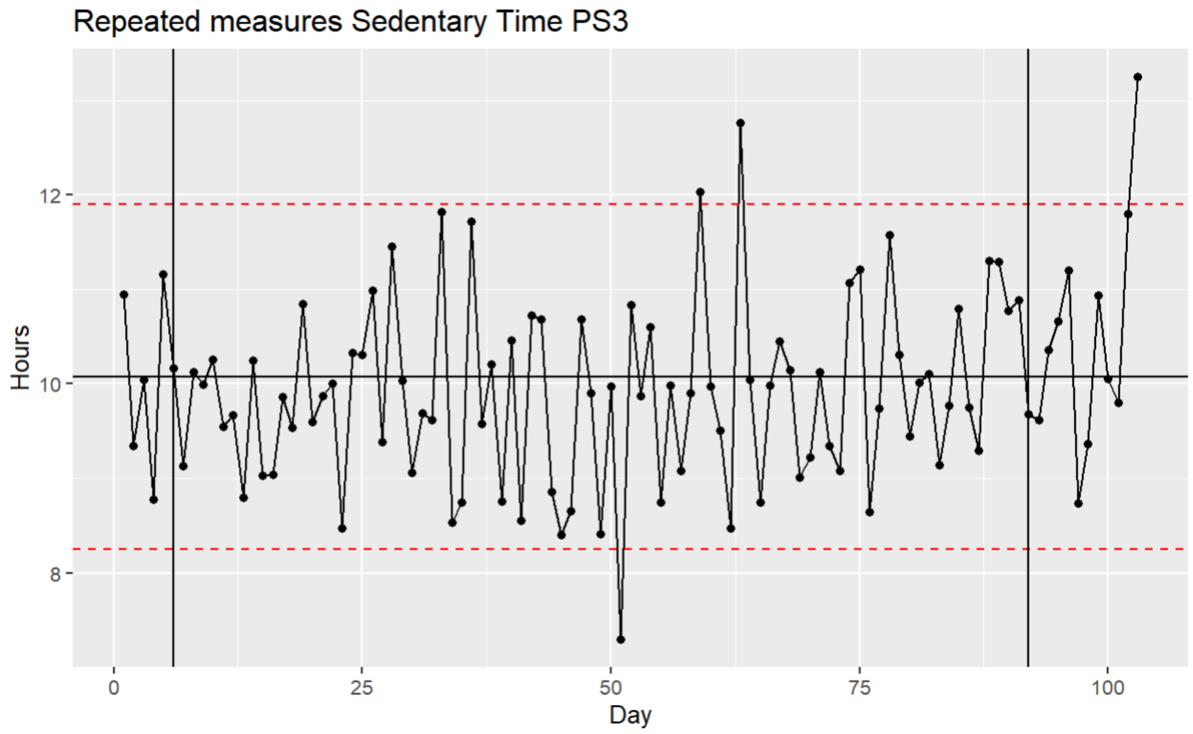

Repeated measures Sedentary Time PS3
Hours
Day
12
10
8
0
25
50
75
100

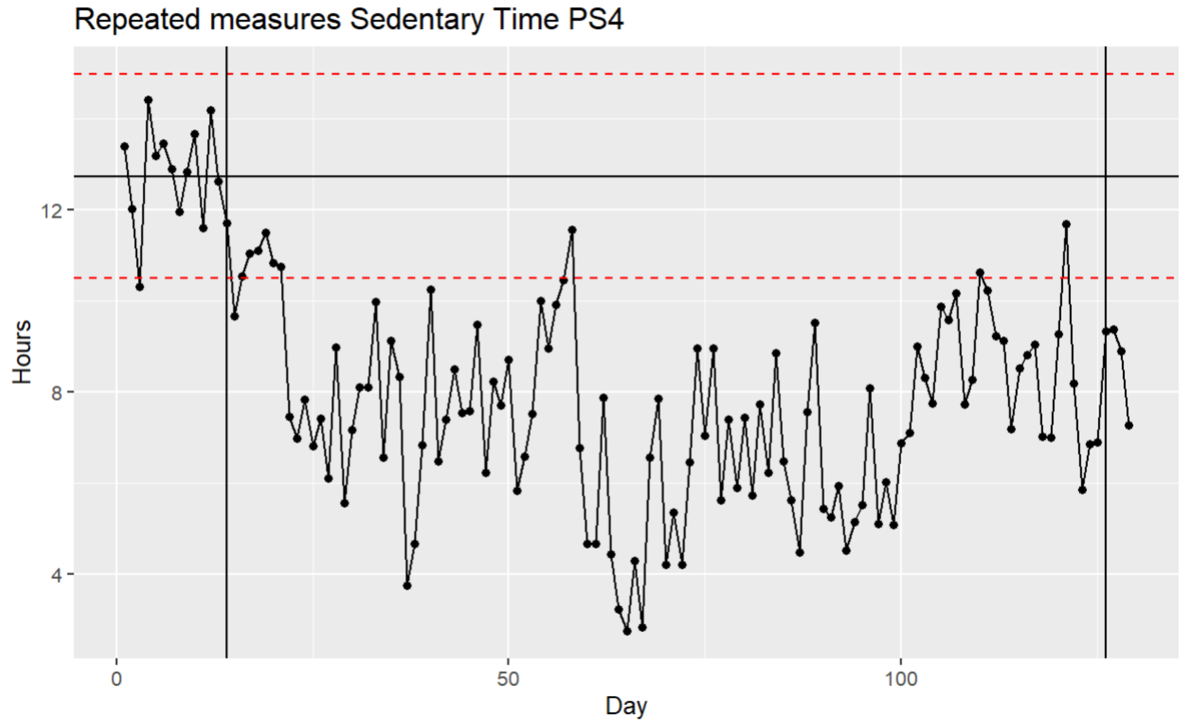
Repeated measures Sedentary Time PS4
Hours
12
8
4
0
50
100
Day

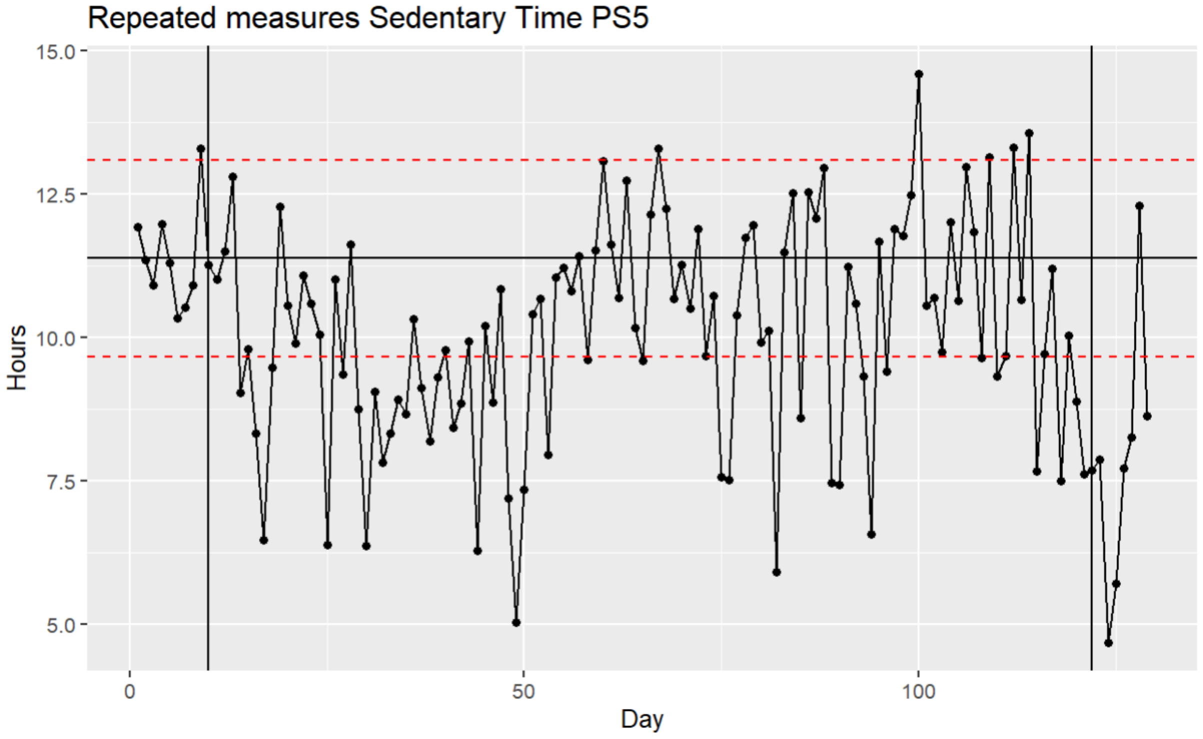
Repeated measures Sedentary Time PS5
Hours
15.0
12.5
10.0
7.5
5.0
0
50
100
Day

Repeated measures Sedentary Time PS6

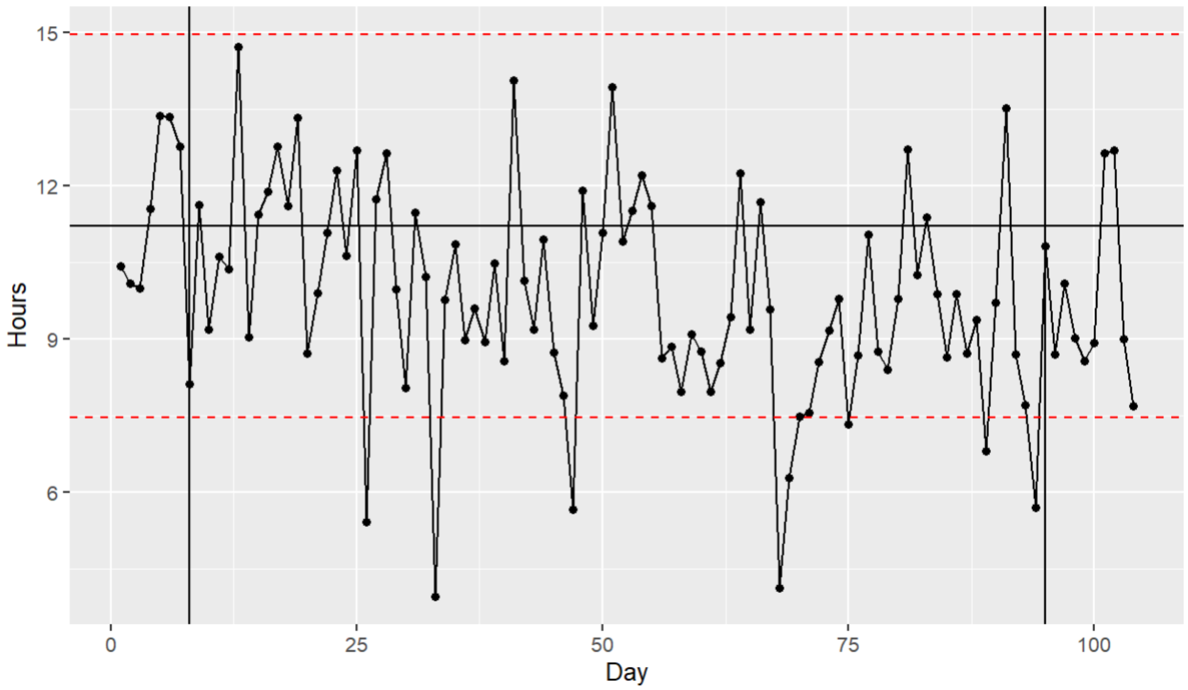

Repeated measures Sedentary Time PS7

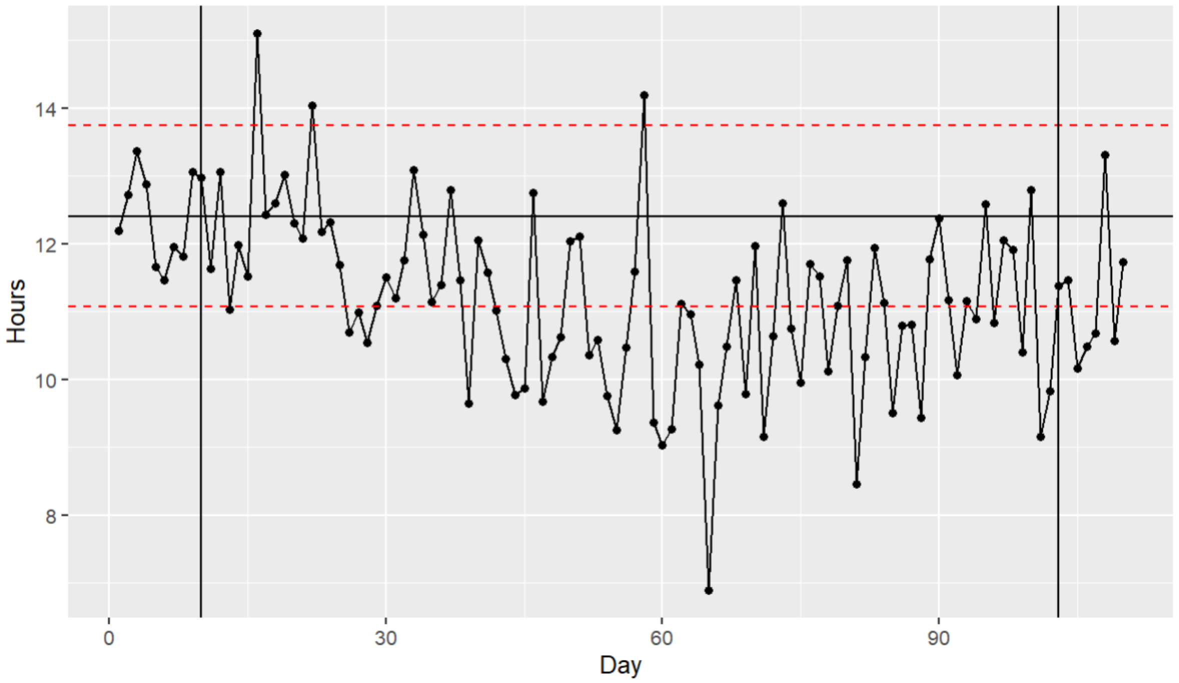

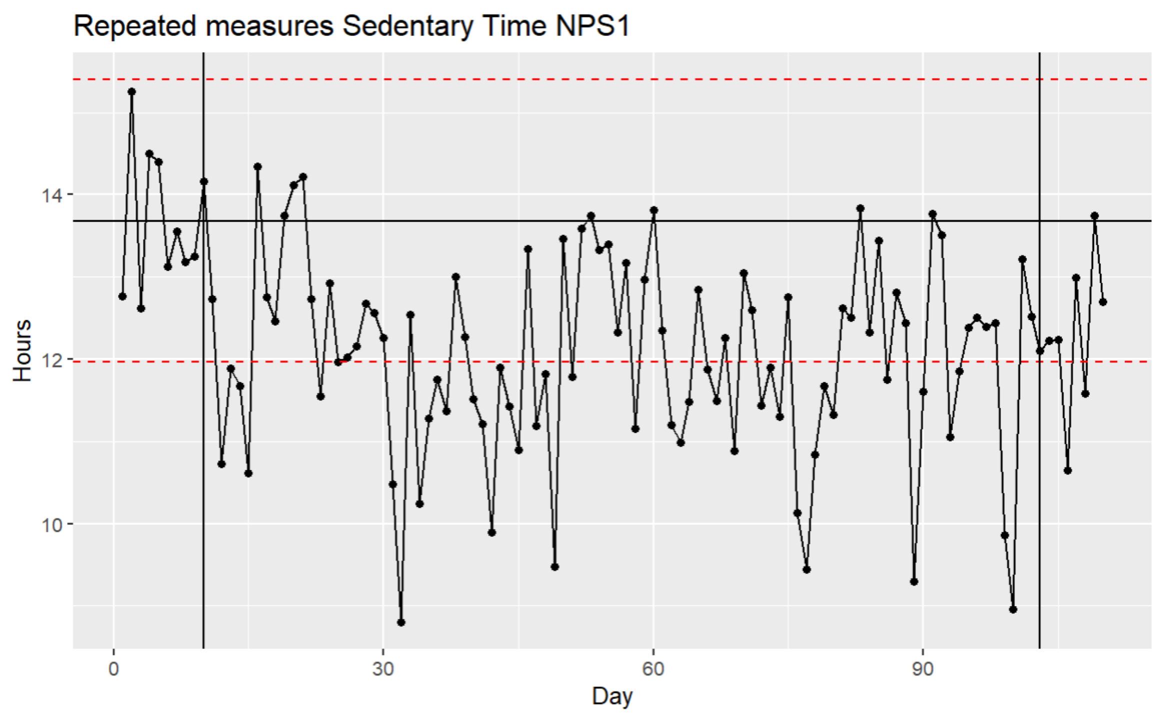
Repeated measures Sedentary Time NPS1
Hours
14
12
10
0
30
60
90
Day

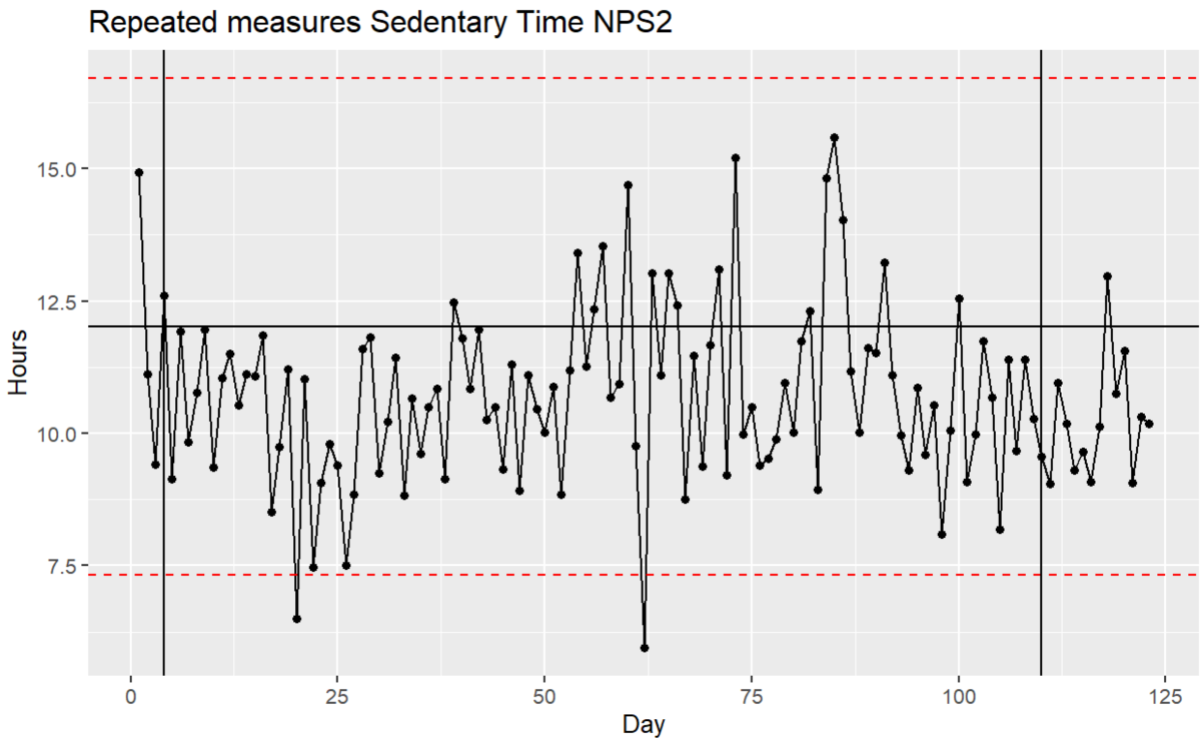
Repeated measures Sedentary Time NPS2
Hours
15.0
12.5
10.0
7.5
0
25
50
75
100
125
Day

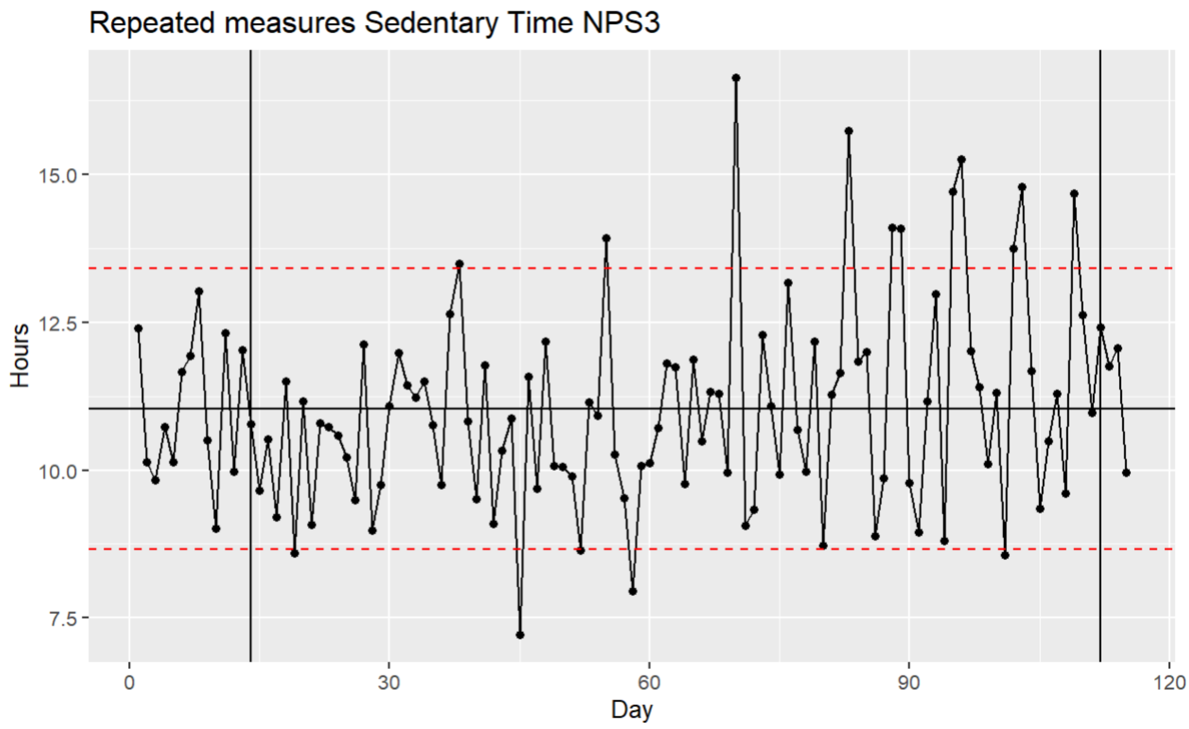

Repeated measures Sedentary Time NPS3
Hours
Day
15.0
12.5
10.0
7.5
0
30
60
90
120

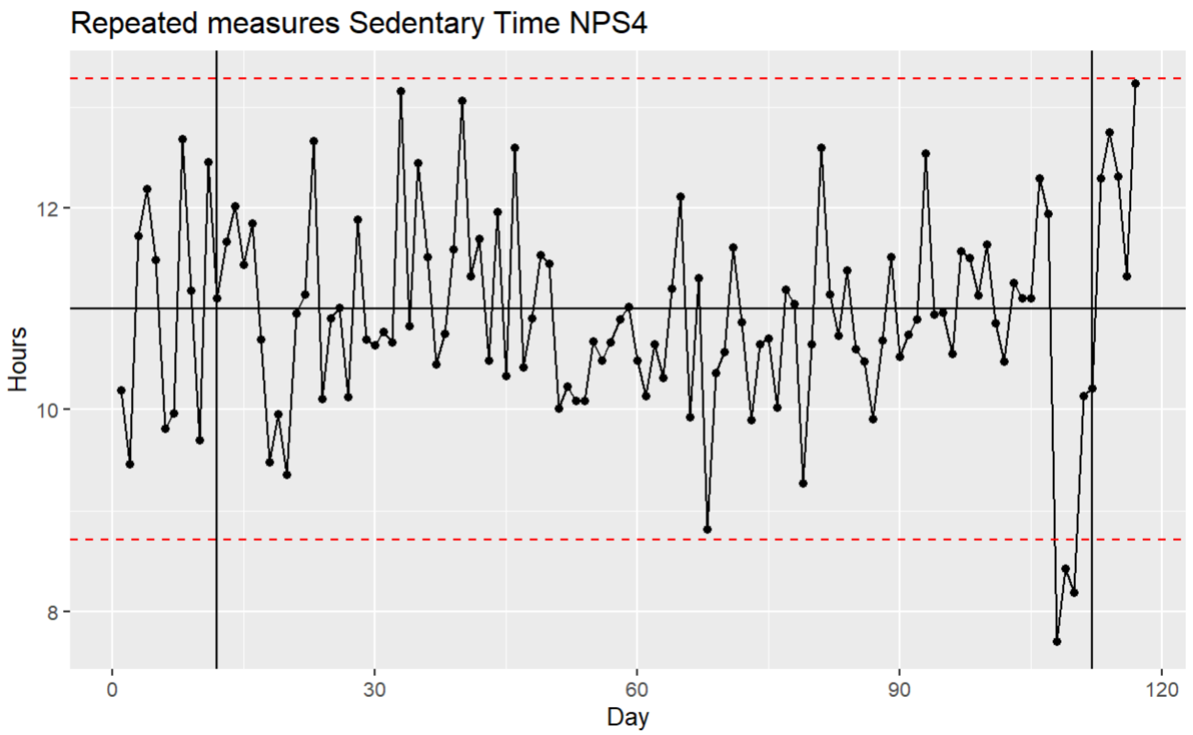

Repeated measures Sedentary Time NPS4
Hours
Day
12
10
8
0
30
60
90
120

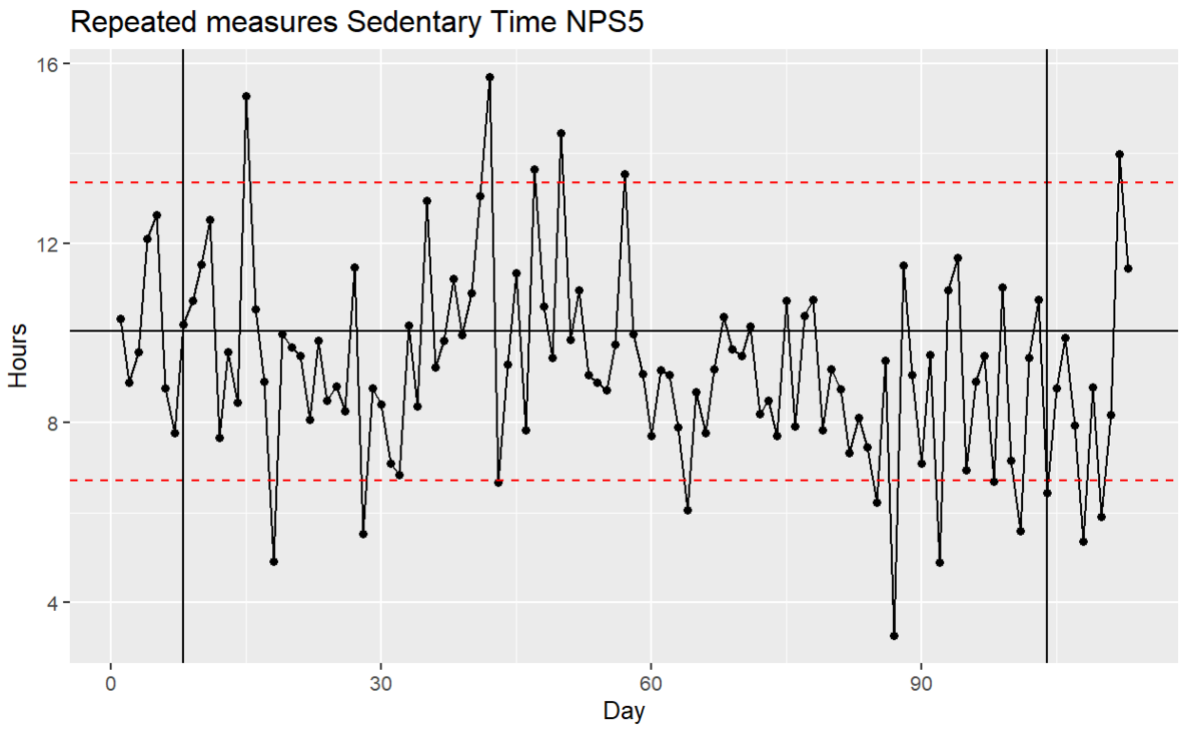

Repeated measures Sedentary Time NPS5
Hours
Day
16
12
8
4
0
30
60
90

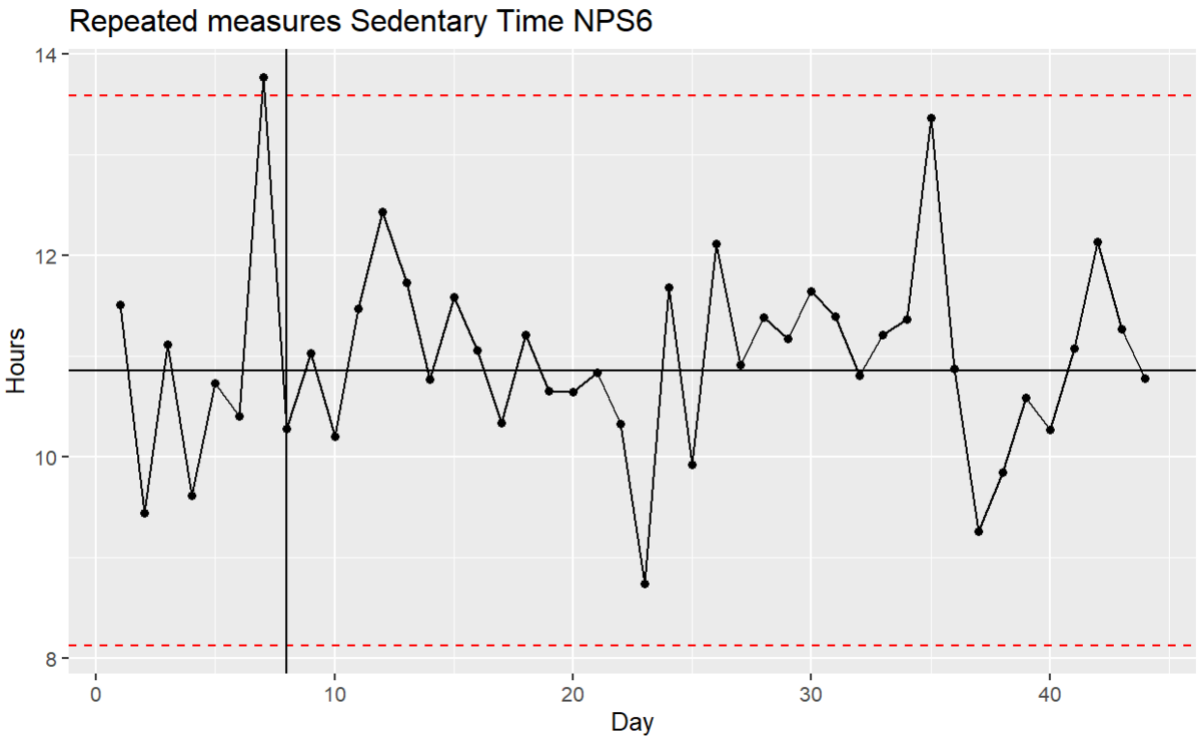

Repeated measures Sedentary Time NPS6
Hours
Day
14
12
10
8
0
10
20
30
40

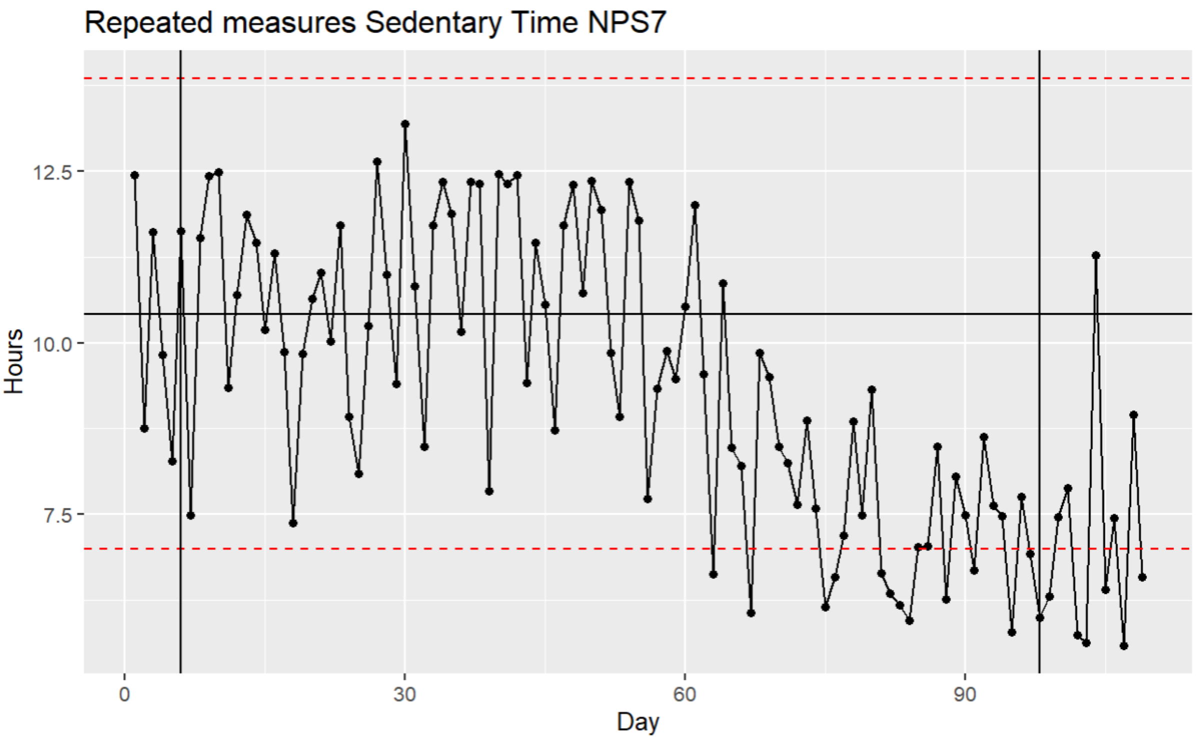

Repeated measures Sedentary Time NPS7
12.5
10.0
7.5
Hours
0
30
60
90
Day

Fragmentation of sedentary time

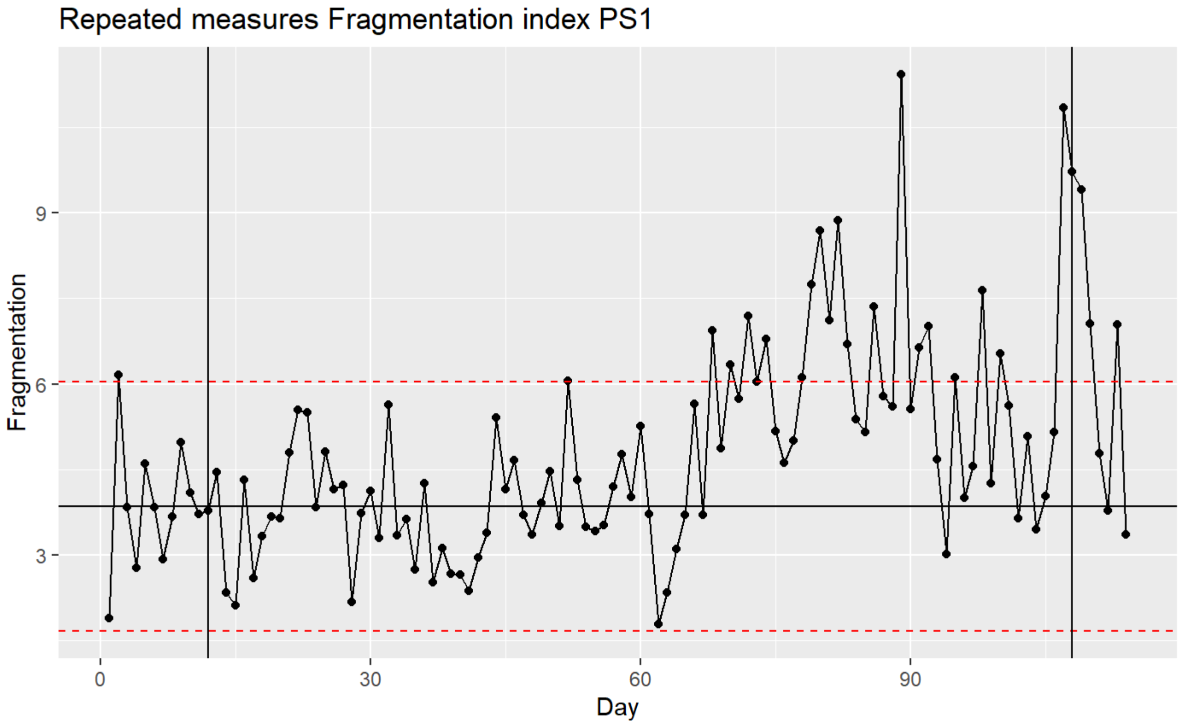

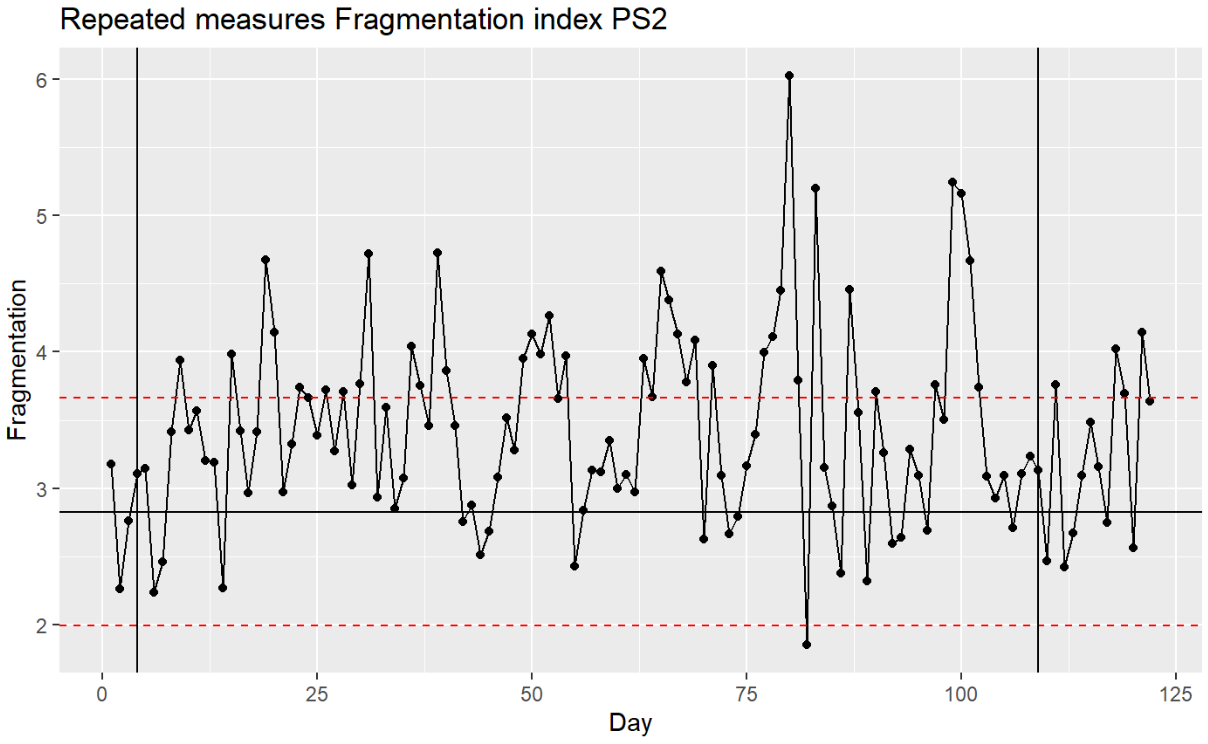

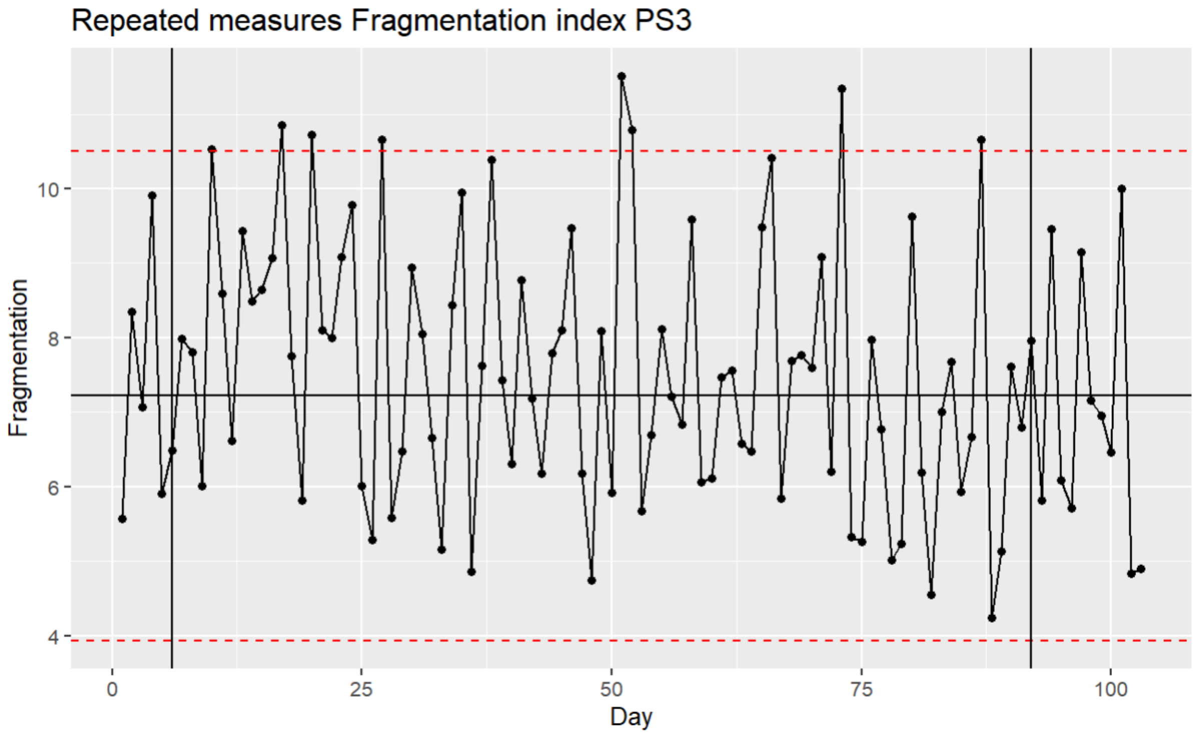

Repeated measures Fragmentation index PS3
Fragmentation
10
8
6
4
0
25
50
75
100
Day

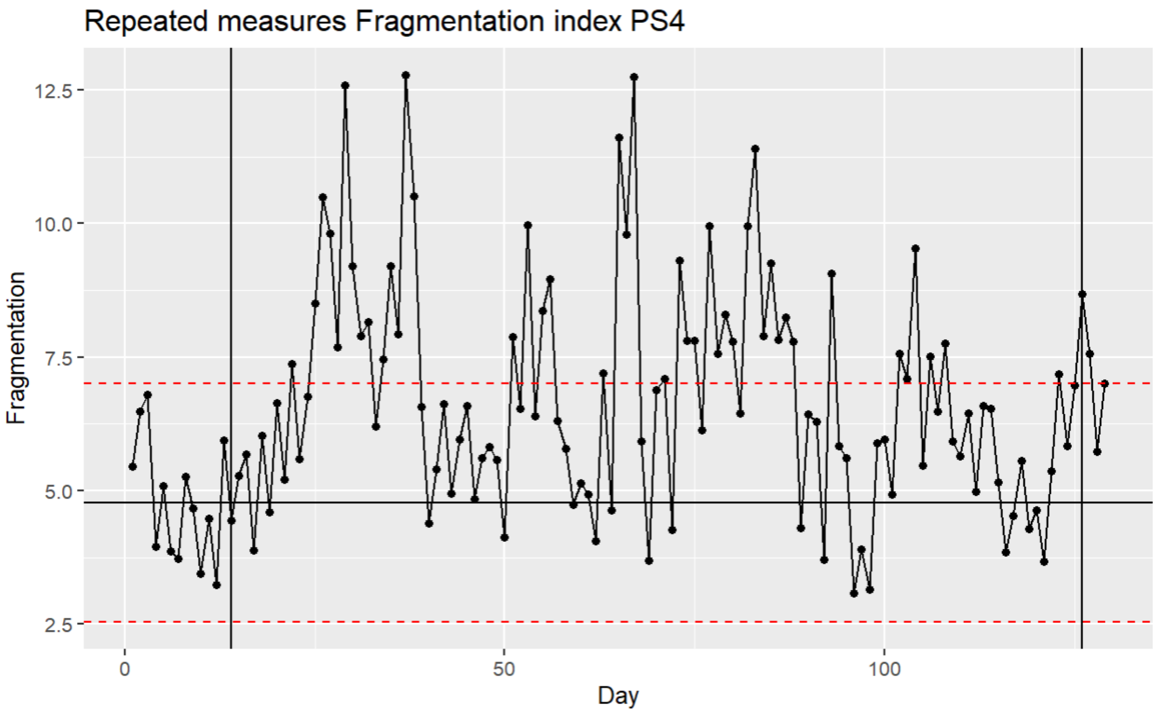

Repeated measures Fragmentation index PS4
Fragmentation
12.5
10.0
7.5
5.0
2.5
0
50
100
Day

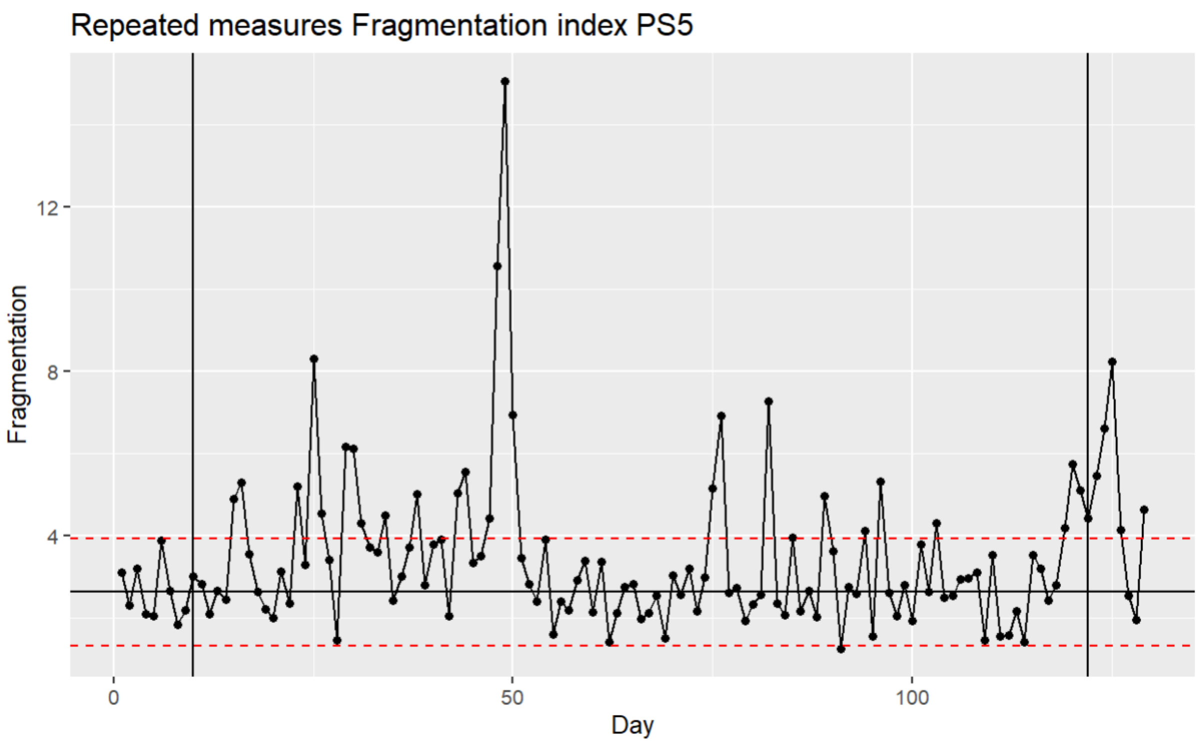
Repeated measures Fragmentation index PS5
Fragmentation
Day
12
8
4
0
50
100

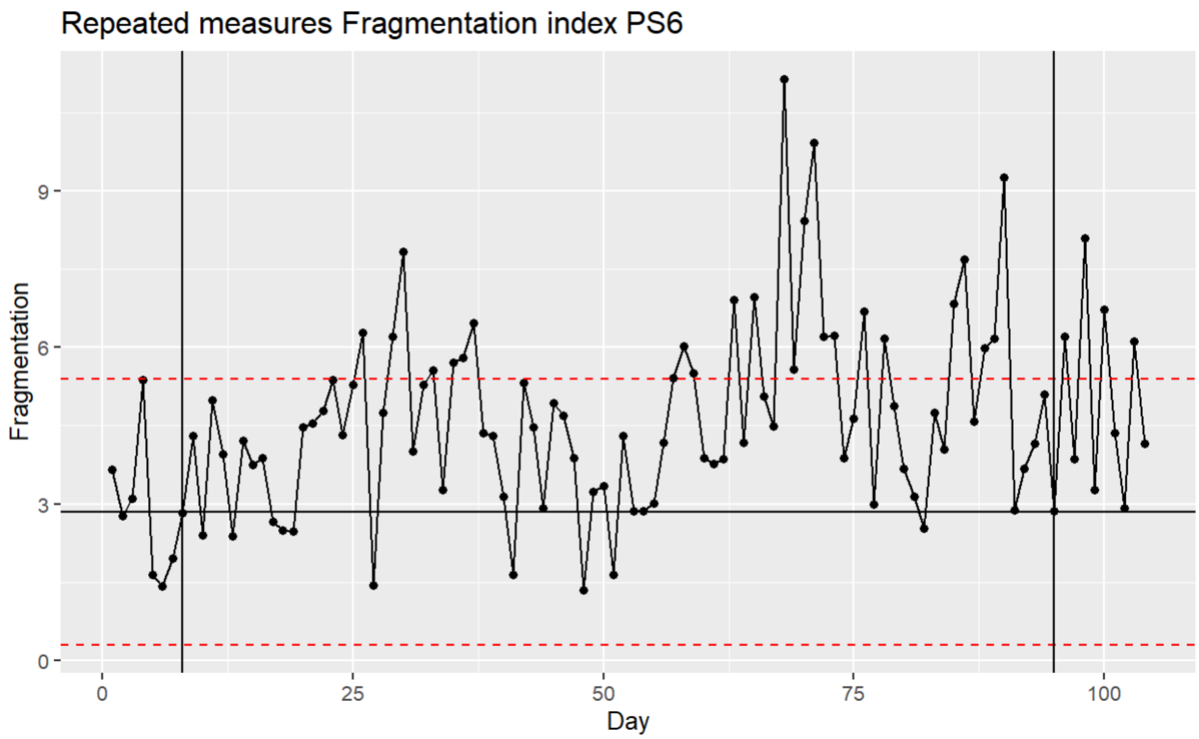
Repeated measures Fragmentation index PS6
Fragmentation
Day
9
6
3
0
0
25
50
75
100

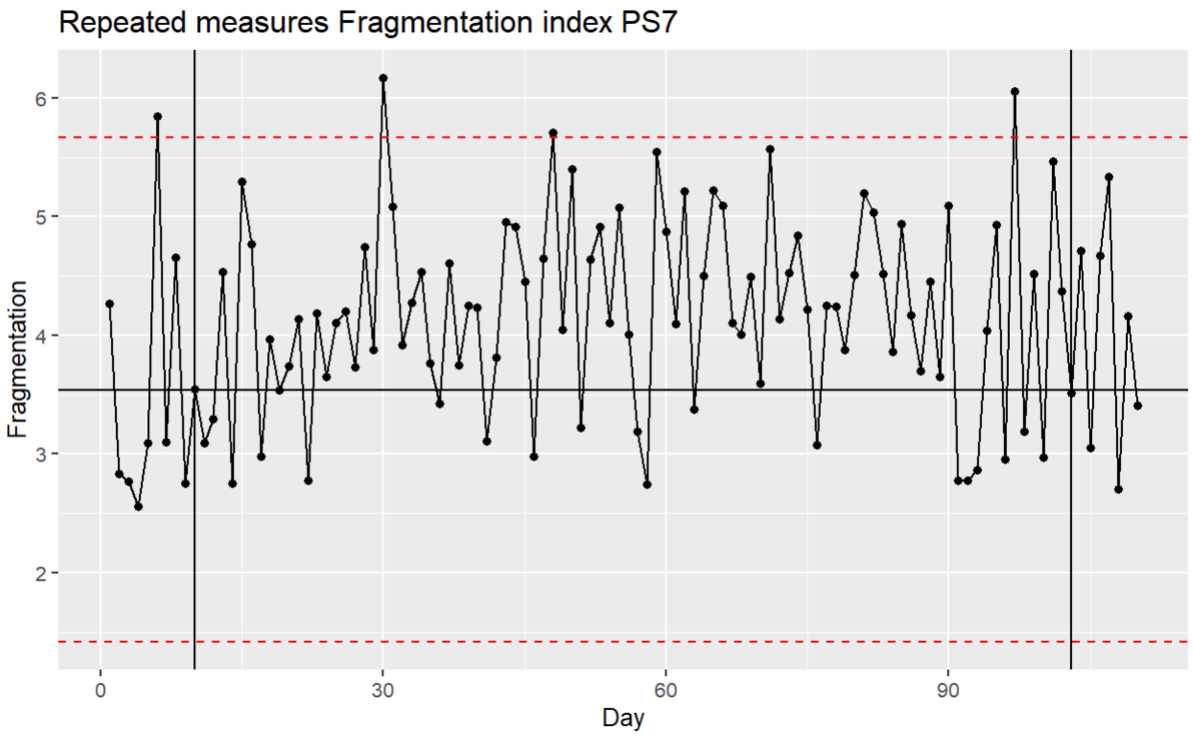
Repeated measures Fragmentation index PS7
Fragmentation
Day
6
5
4
3
2
0
30
60
90

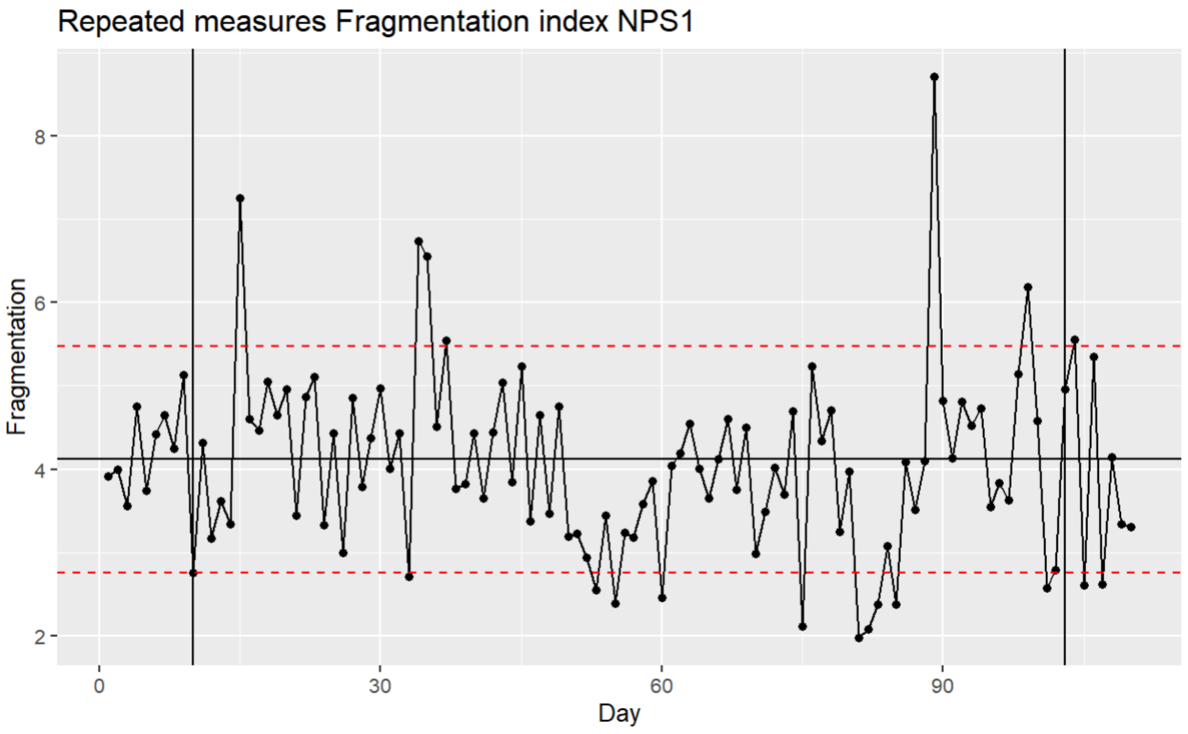

Repeated measures Fragmentation index NPS1
Fragmentation
Day
2
4
6
8
0
30
60
90

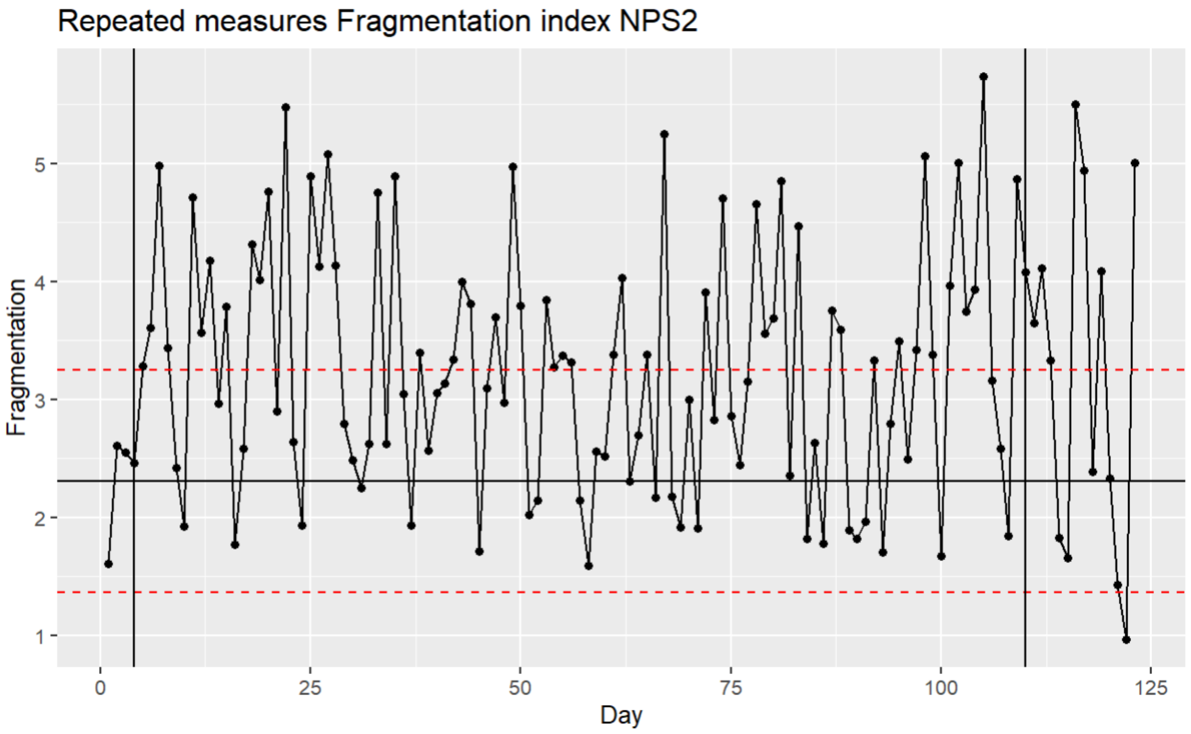

Repeated measures Fragmentation index NPS2
Fragmentation
Day
1
2
3
4
5
0
25
50
75
100
125

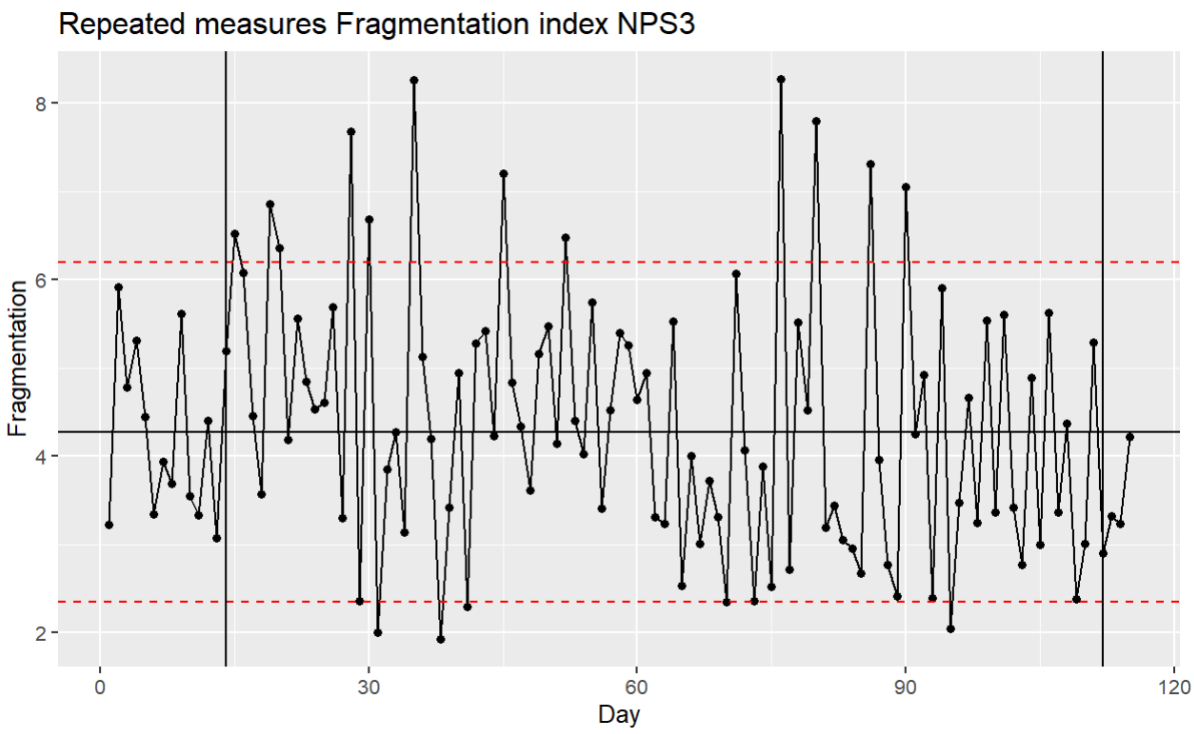
Repeated measures Fragmentation index NPS3
Fragmentation
Day
2
4
6
8
0
30
60
90
120

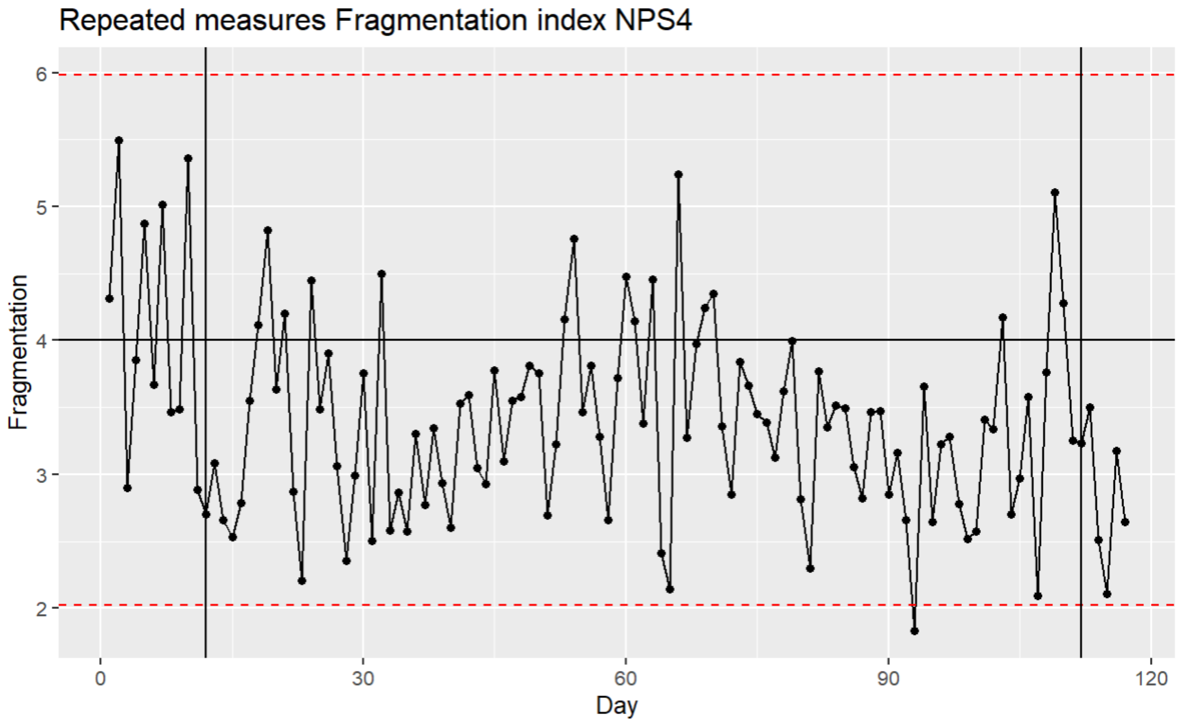
Repeated measures Fragmentation index NPS4
Fragmentation
Day
2
3
4
5
6
0
30
60
90
120

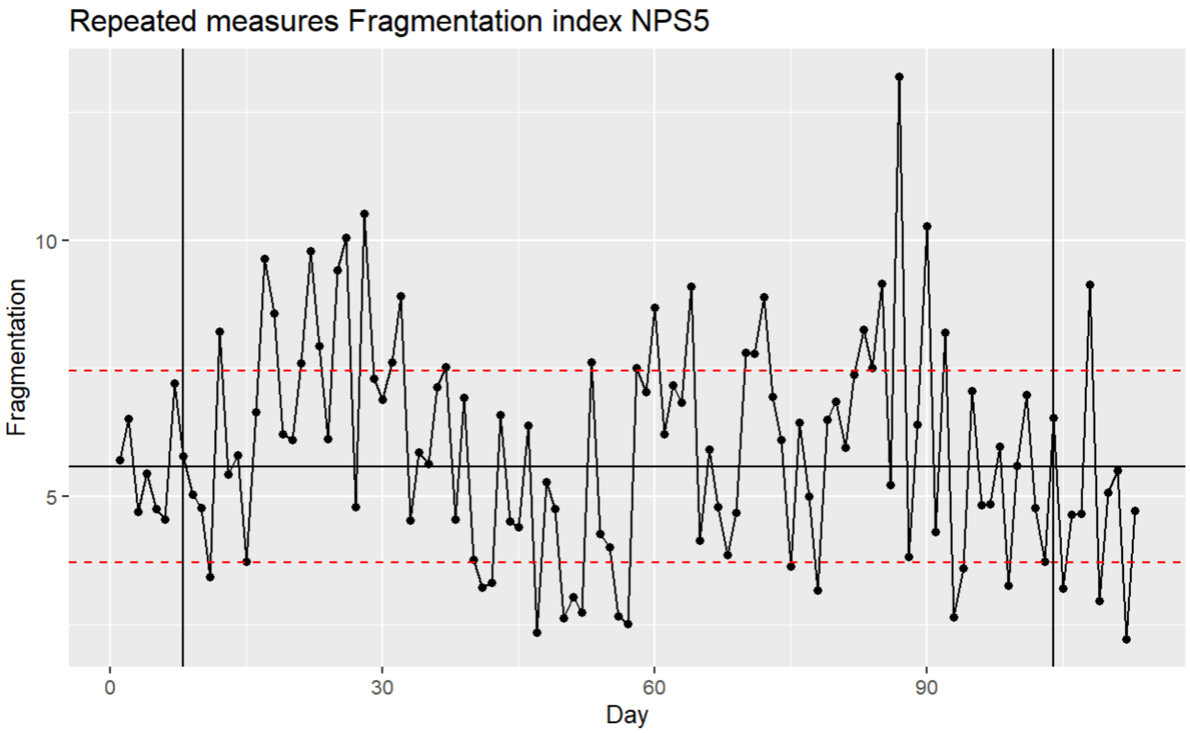

Repeated measures Fragmentation index NPS5
Fragmentation
10
5
0
30
60
90
Day

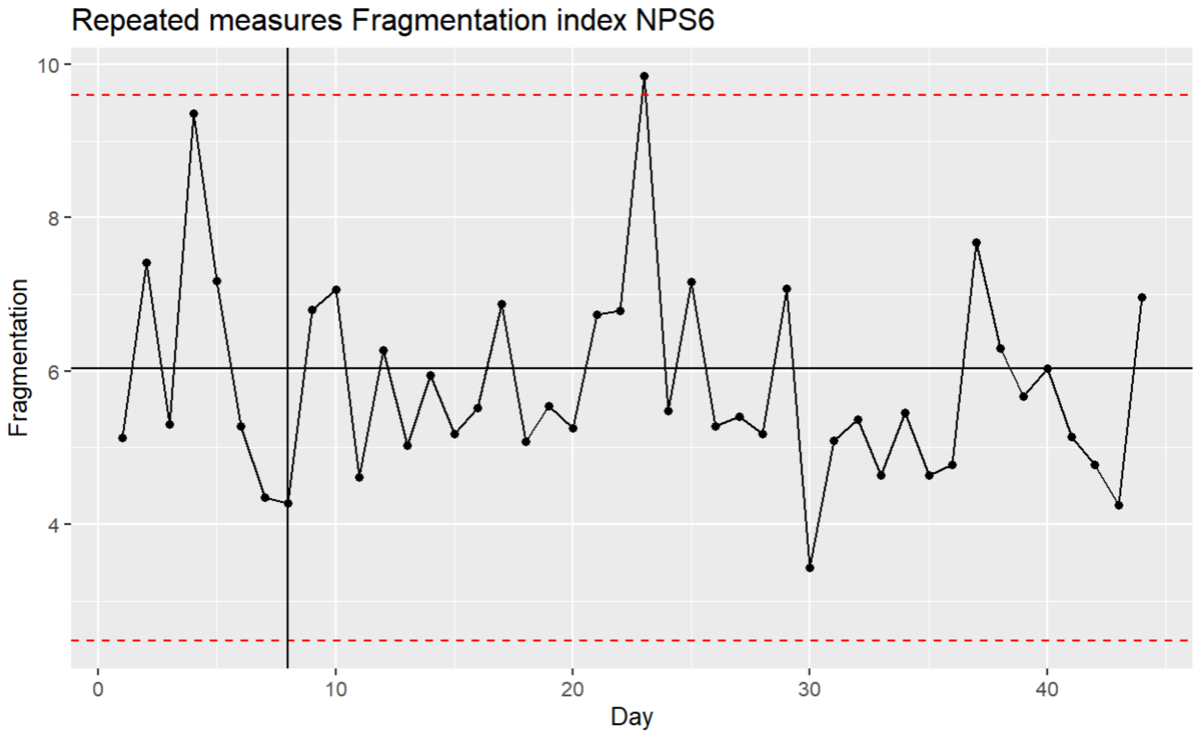

Repeated measures Fragmentation index NPS6
Fragmentation
10
8
6
4
0
10
20
30
40
Day

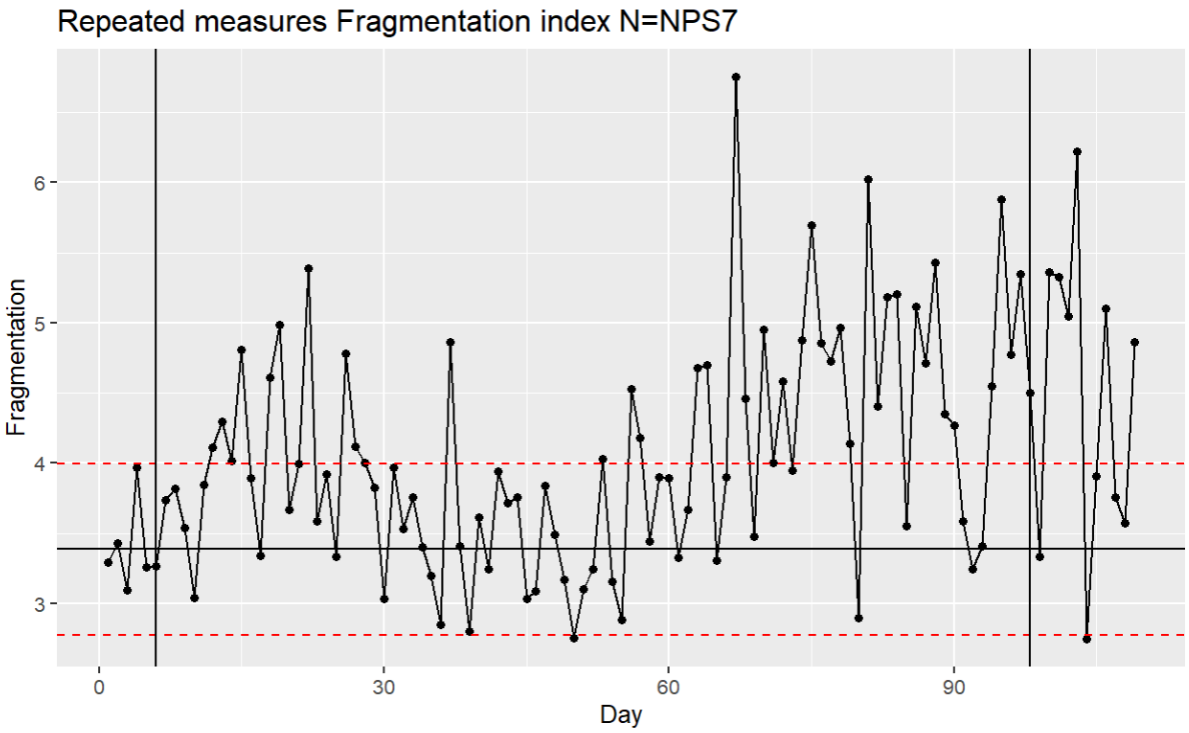

Repeated measures Fragmentation index N=NPS7
Fragmentation
6
5
4
3
0
30
60
90
Day

Percentage of waking hours spend sedentary

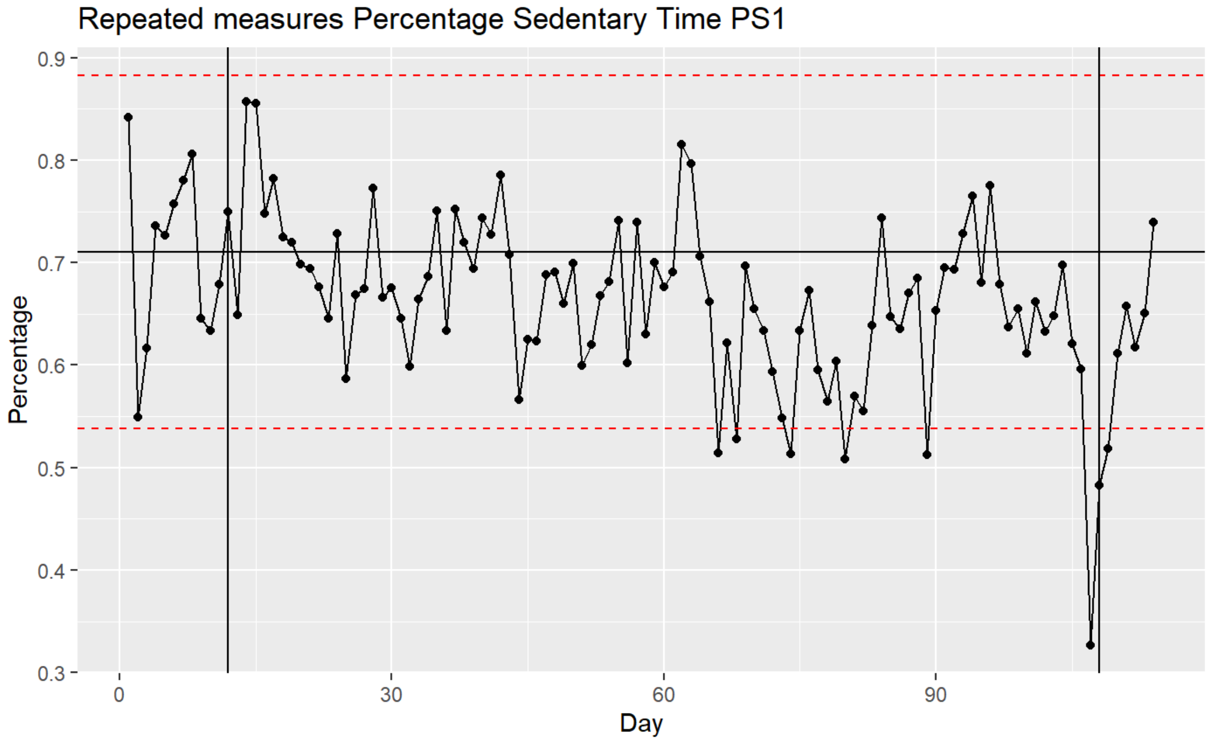

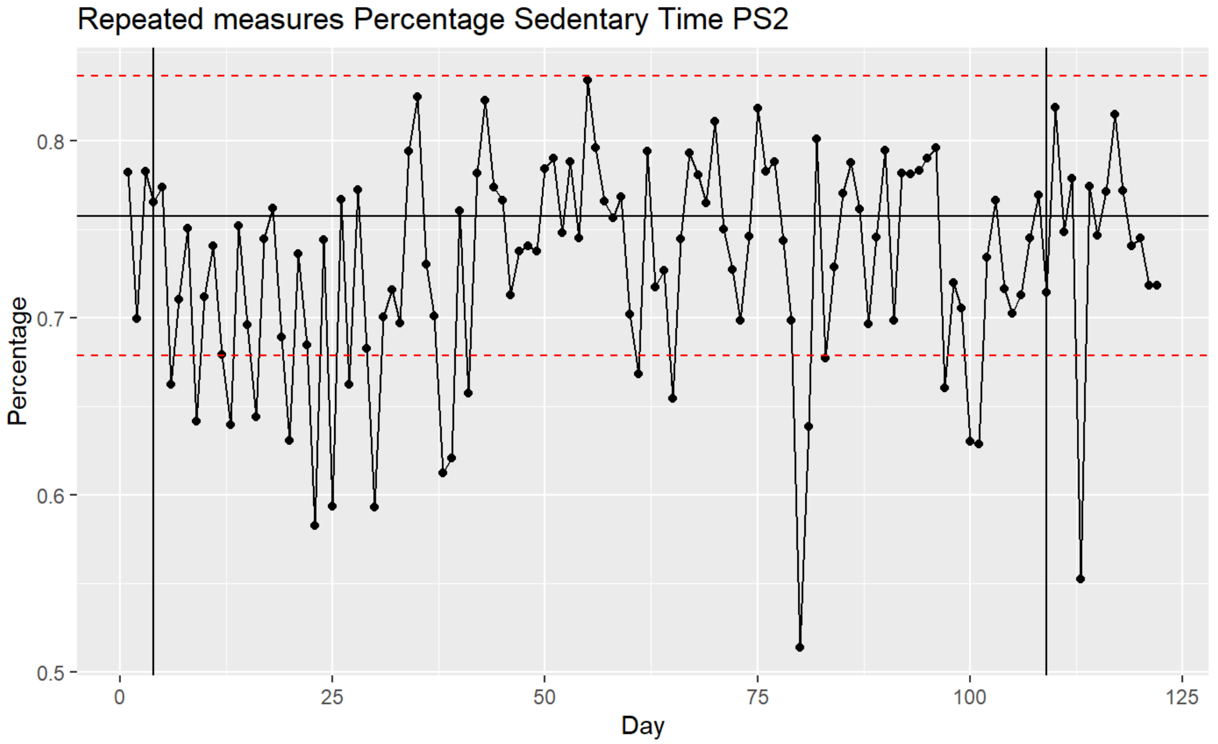

Repeated measures Percentage Sedentary Time PS3

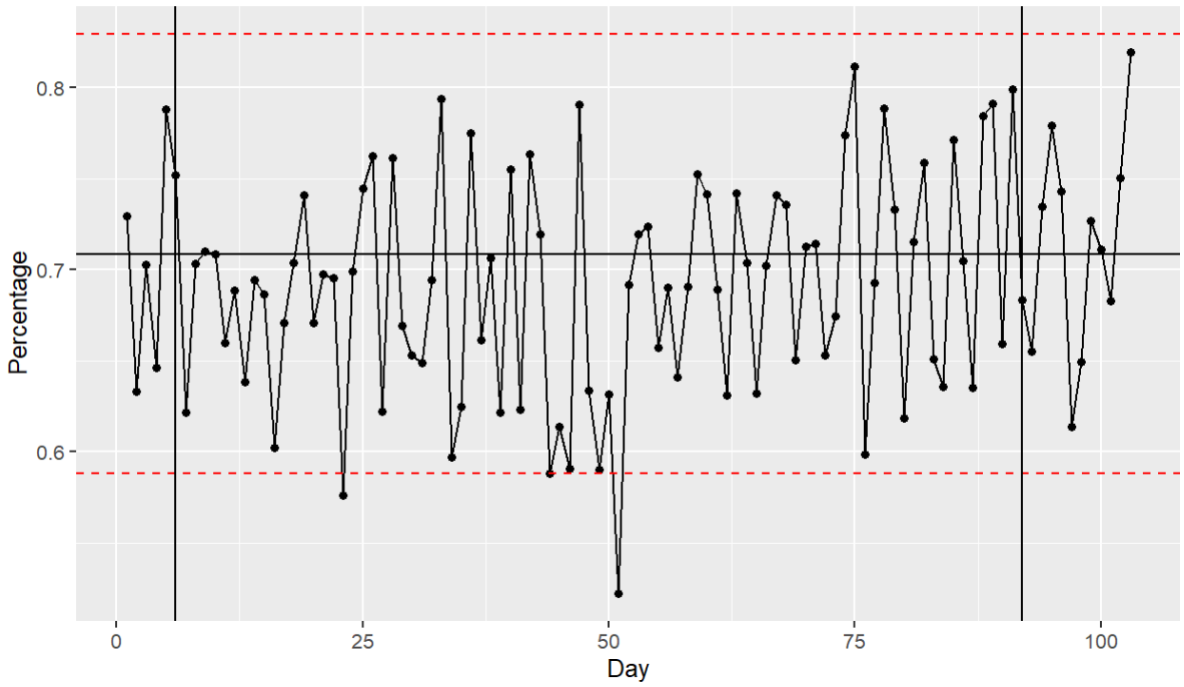

Repeated measures Percentage Sedentary Time PS4

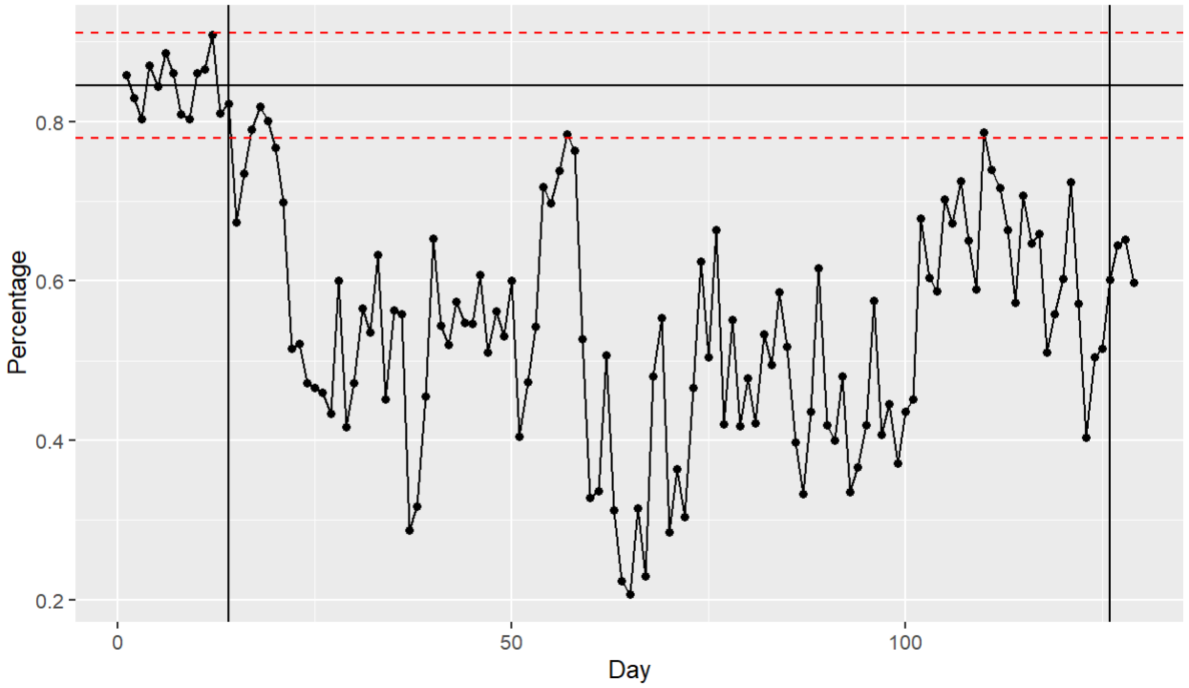

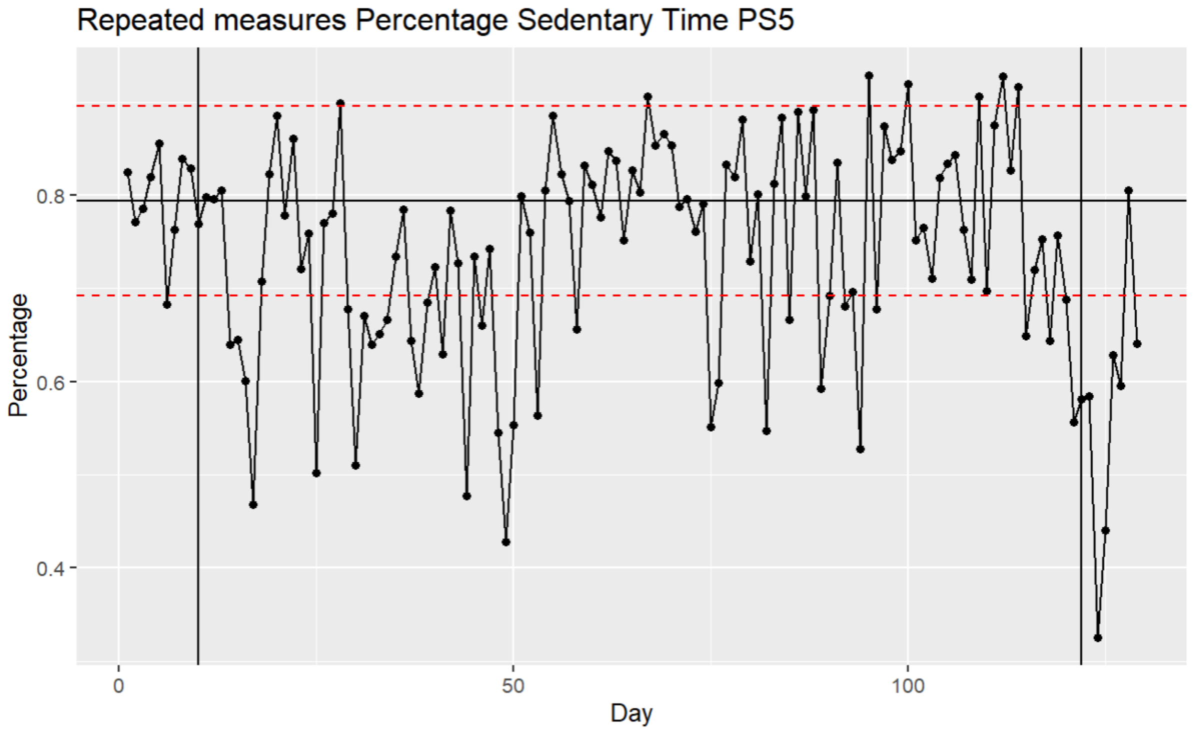

Repeated measures Percentage Sedentary Time PS5
Percentage
0.8
0.6
0.4
0
50
100
Day

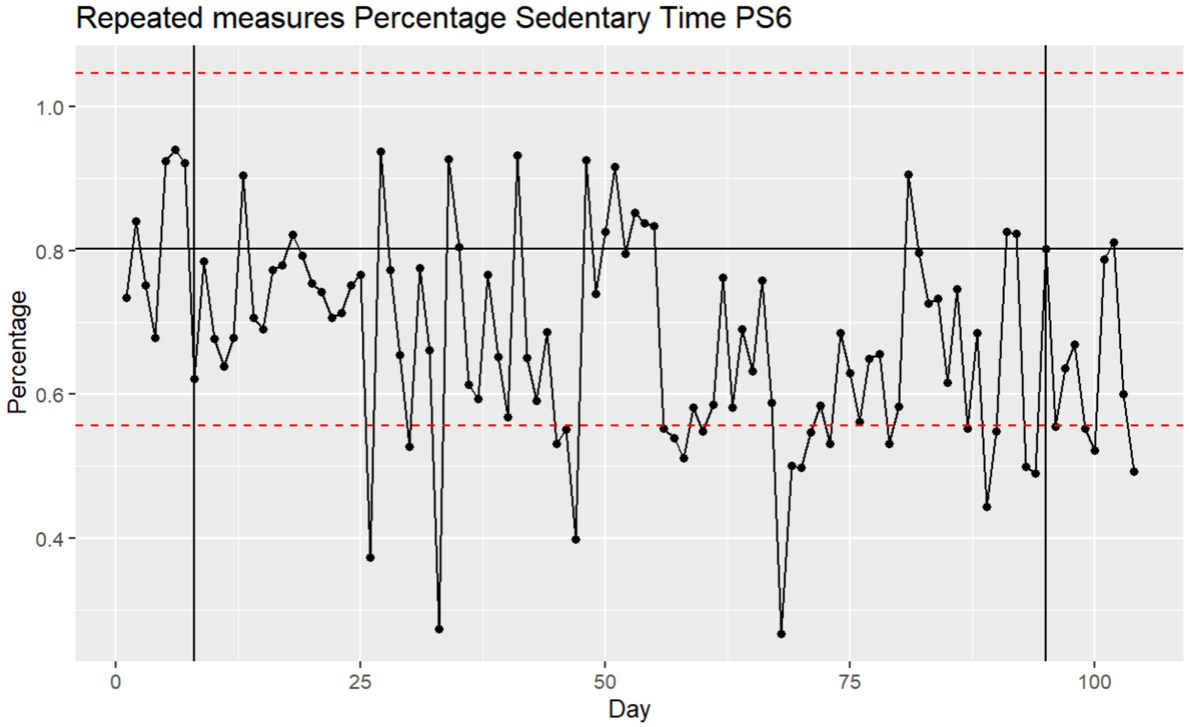

Repeated measures Percentage Sedentary Time PS6
Percentage
1.0
0.8
0.6
0.4
0
25
50
75
100
Day

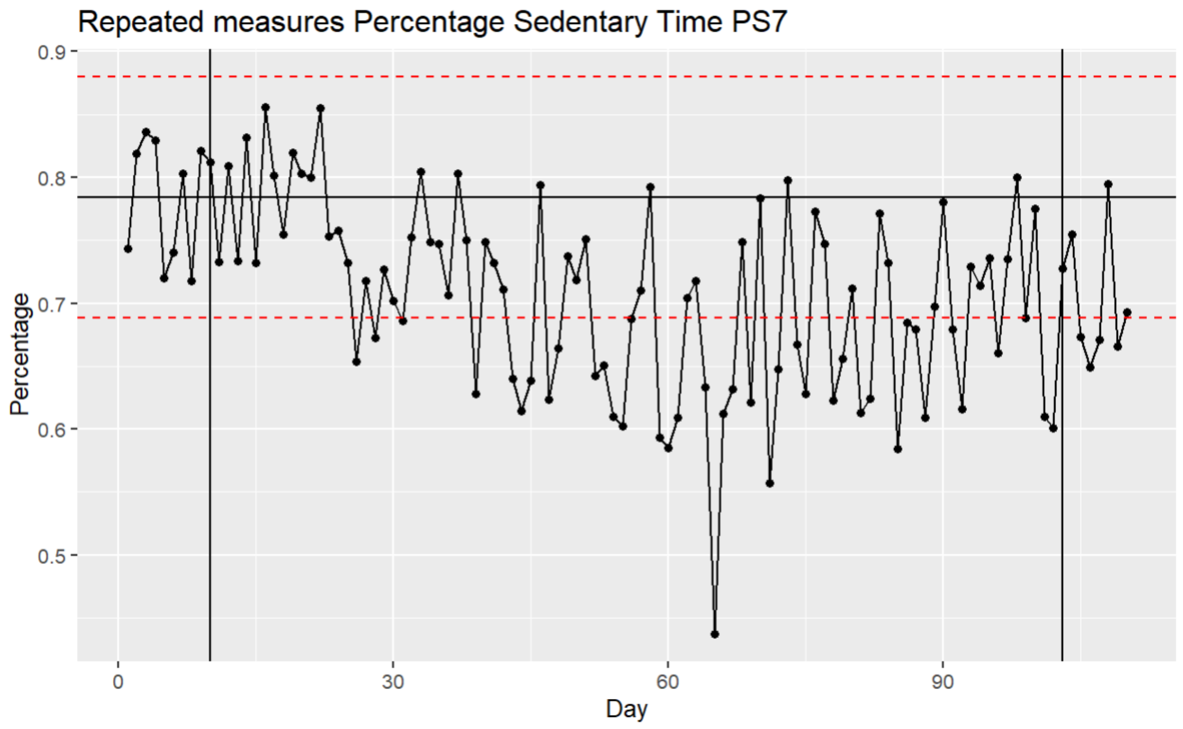

Repeated measures Percentage Sedentary Time PS7
Percentage
0.9
0.8
0.7
0.6
0.5
0
30
60
90
Day

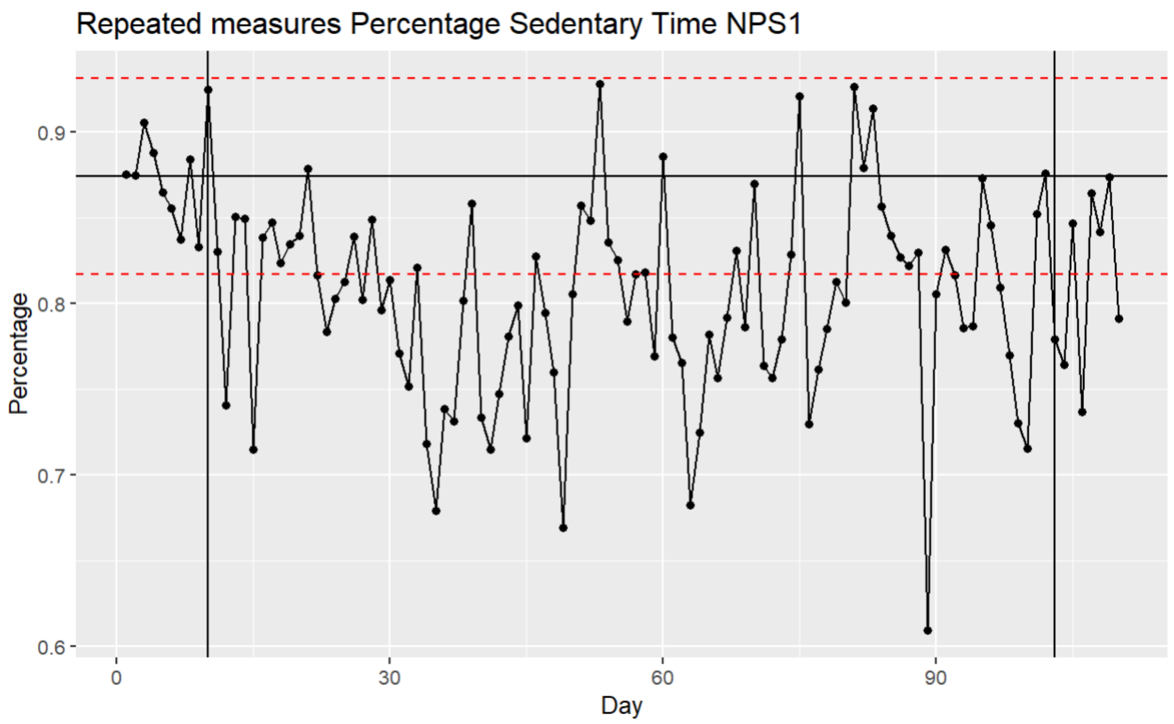

Repeated measures Percentage Sedentary Time NPS1
0.9
0.8
0.7
0.6
Percentage
0
30
60
90
Day

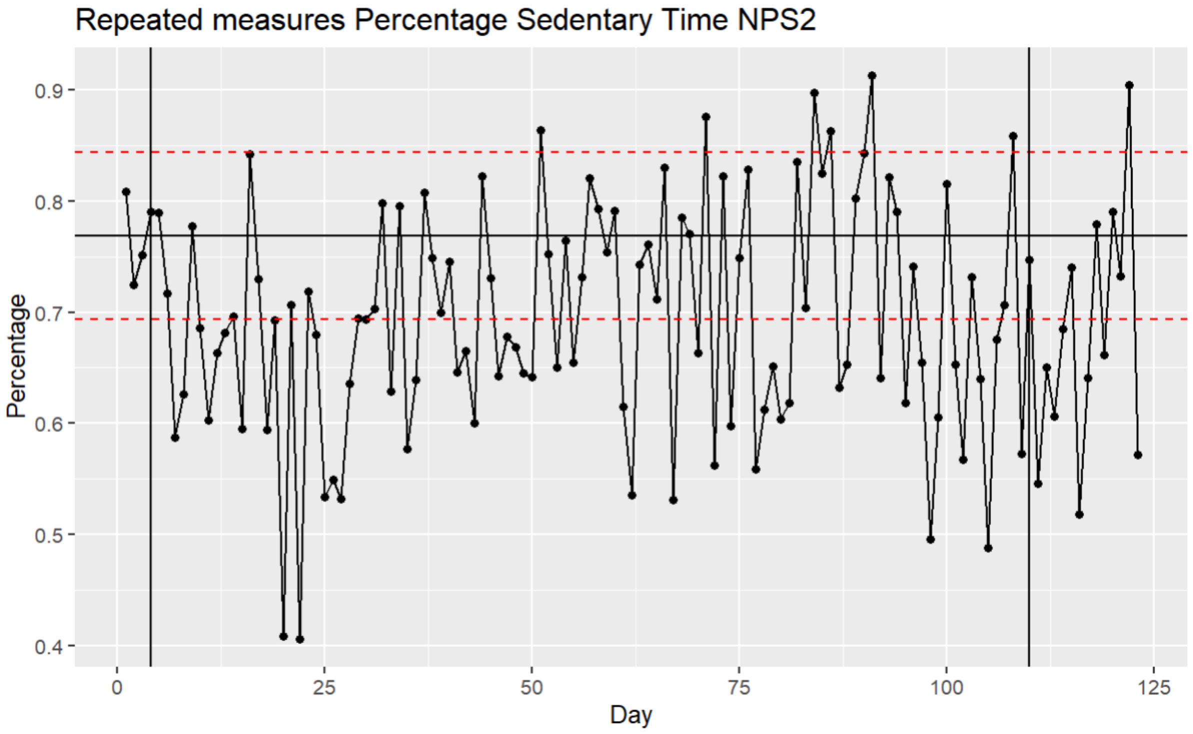

Repeated measures Percentage Sedentary Time NPS2
0.9
0.8
0.7
0.6
0.5
0.4
Percentage
0
25
50
75
100
125
Day

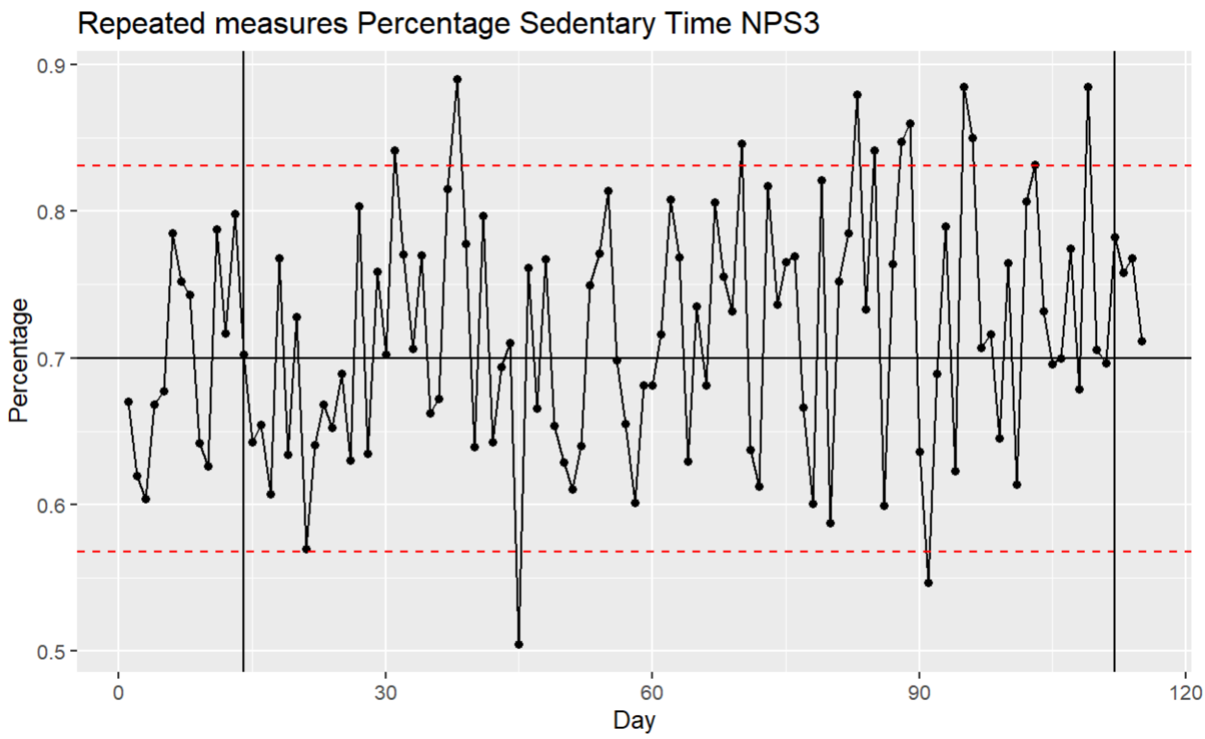
Repeated measures Percentage Sedentary Time NPS3
Percentage
0.9
0.8
0.7
0.6
0.5
0
30
60
90
120
Day

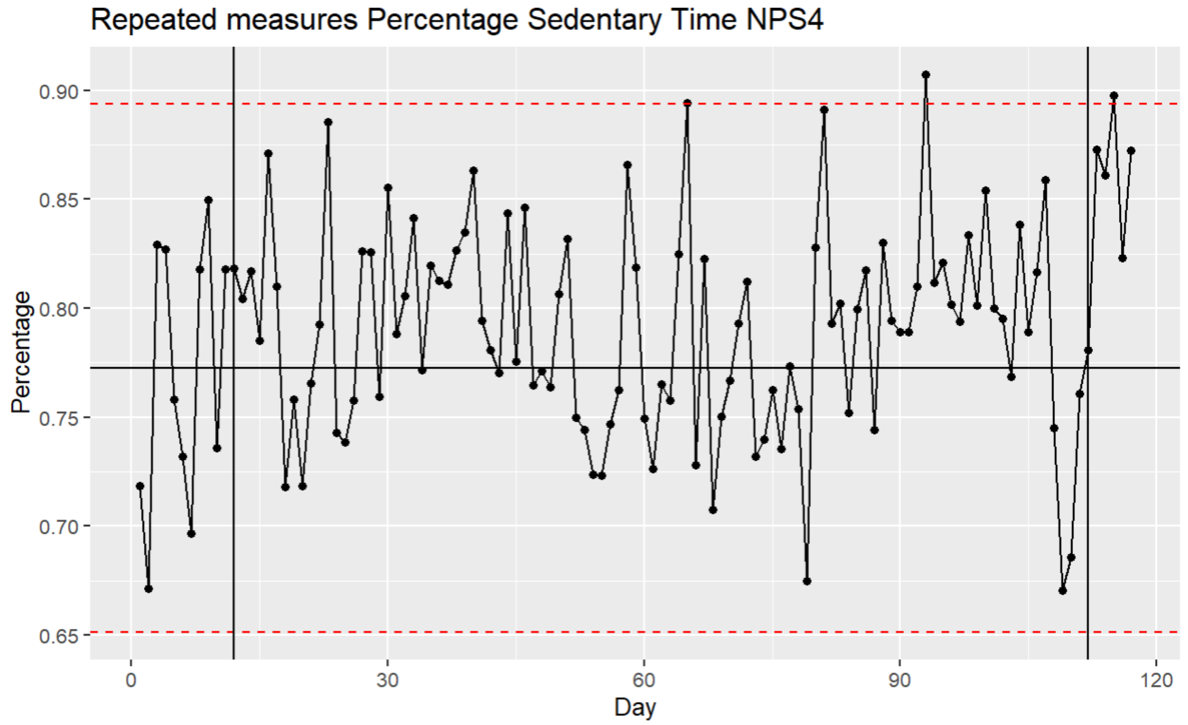
Repeated measures Percentage Sedentary Time NPS4
Percentage
0.90
0.85
0.80
0.75
0.70
0.65
0
30
60
90
120
Day

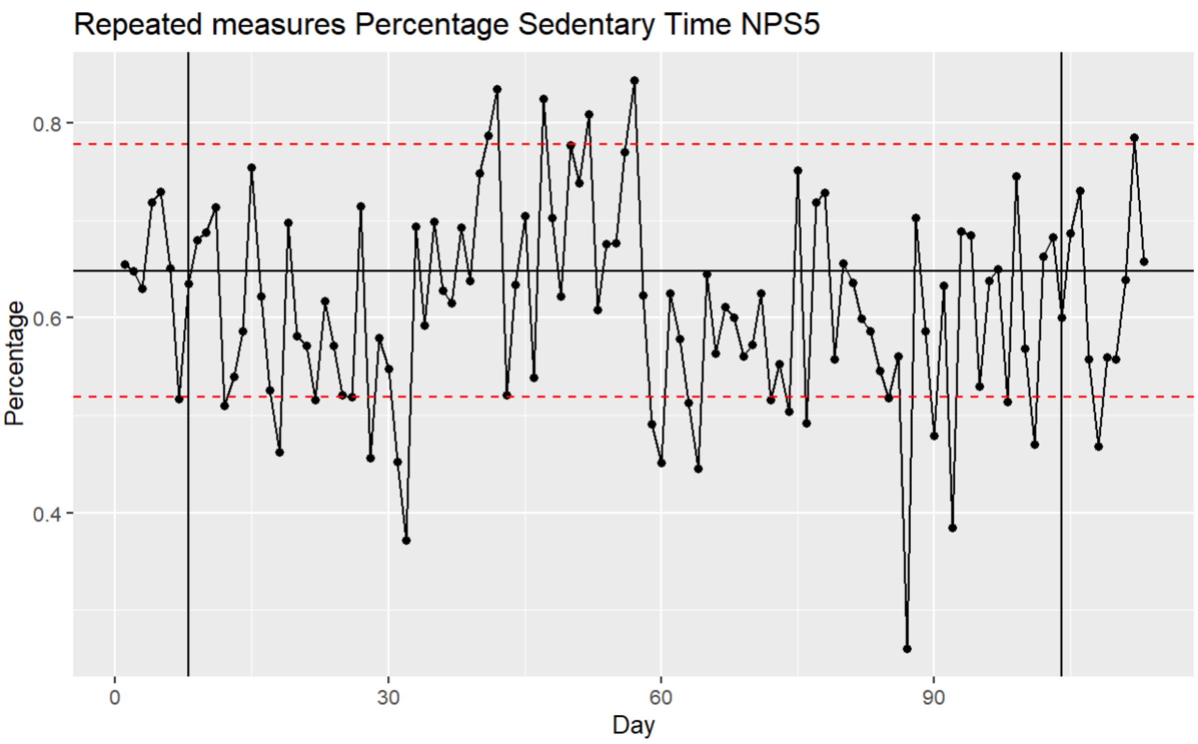
Repeated measures Percentage Sedentary Time NPS5
Percentage
0.8
0.6
0.4
0
30
60
90
Day

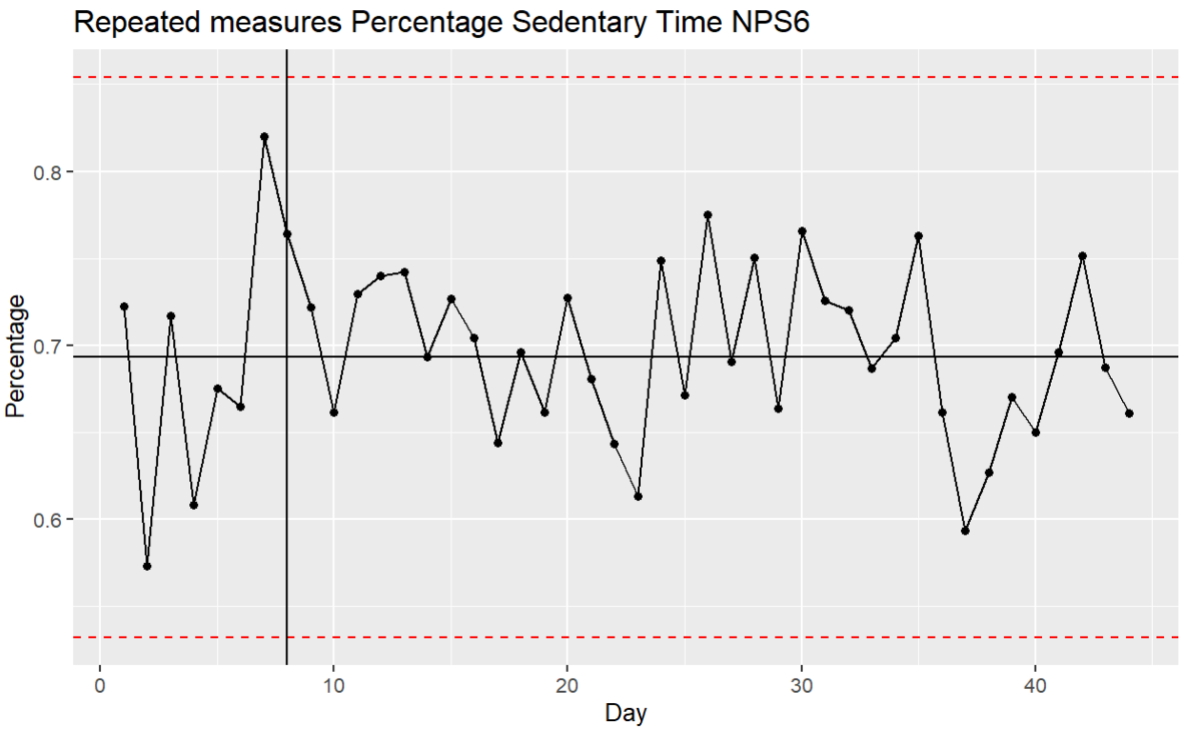
Repeated measures Percentage Sedentary Time NPS6
Percentage
0.8
0.7
0.6
0
10
20
30
40
Day

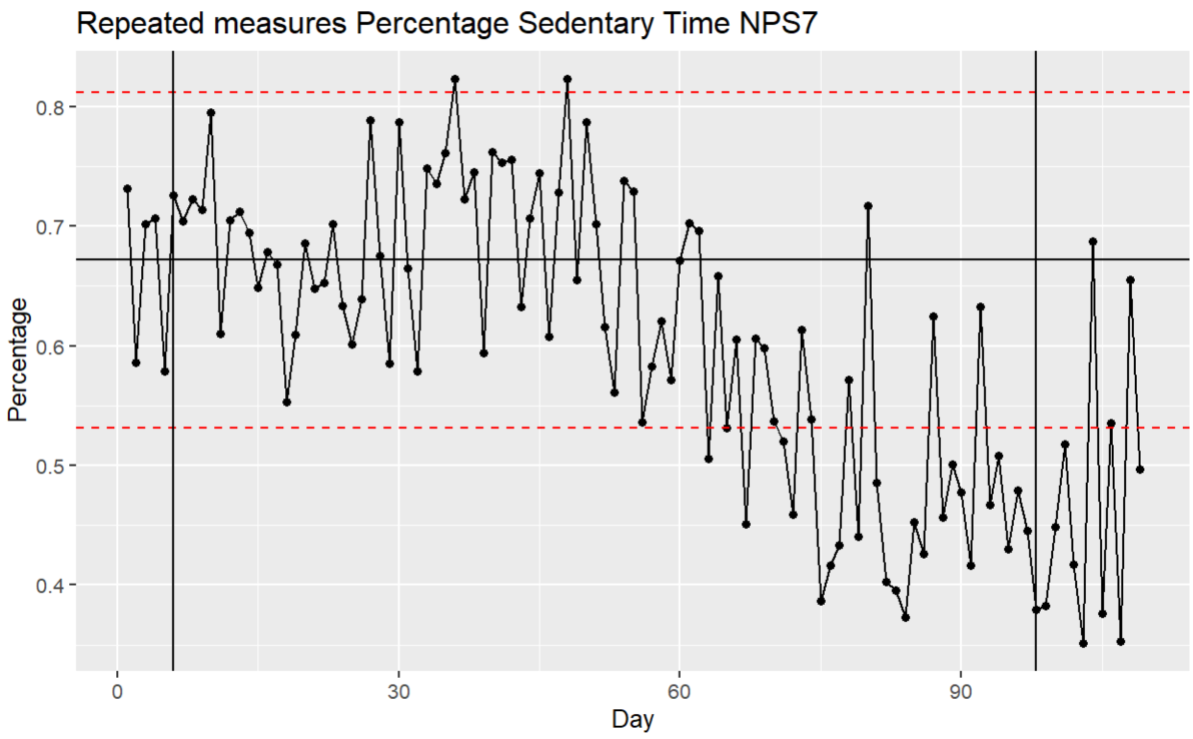
Repeated measures Percentage Sedentary Time NPS7
Percentage
0.8
0.7
0.6
0.5
0.4
0
30
60
90
Day

Average sedentary bout duration

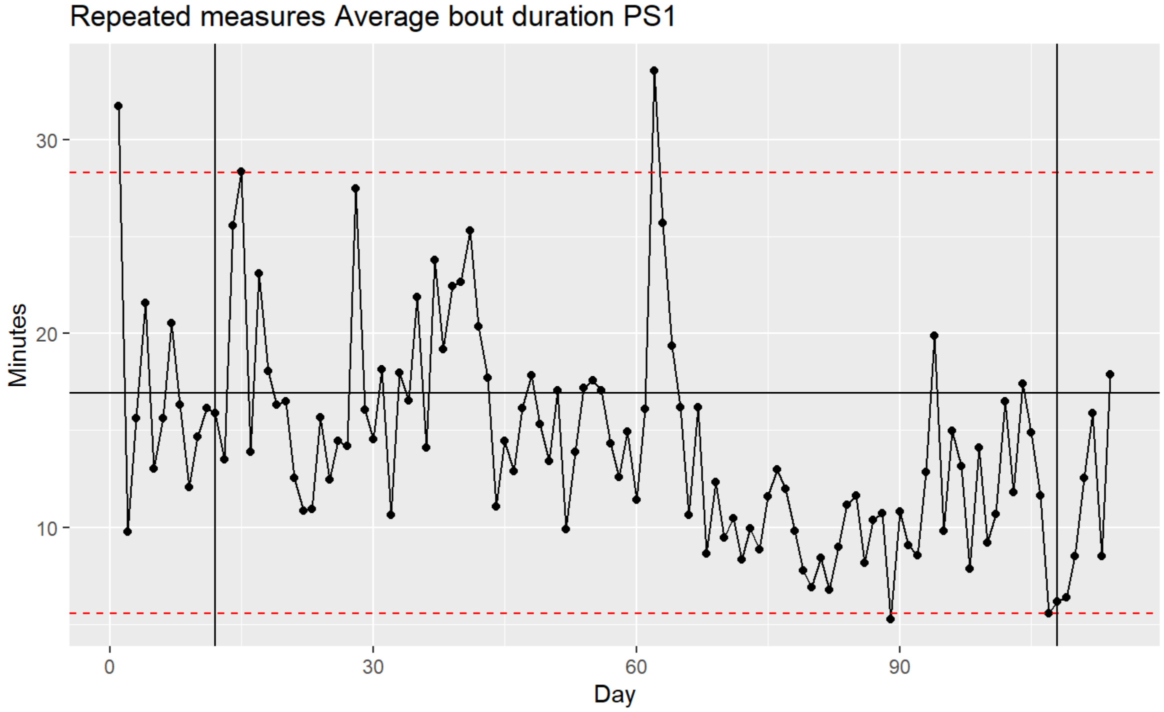

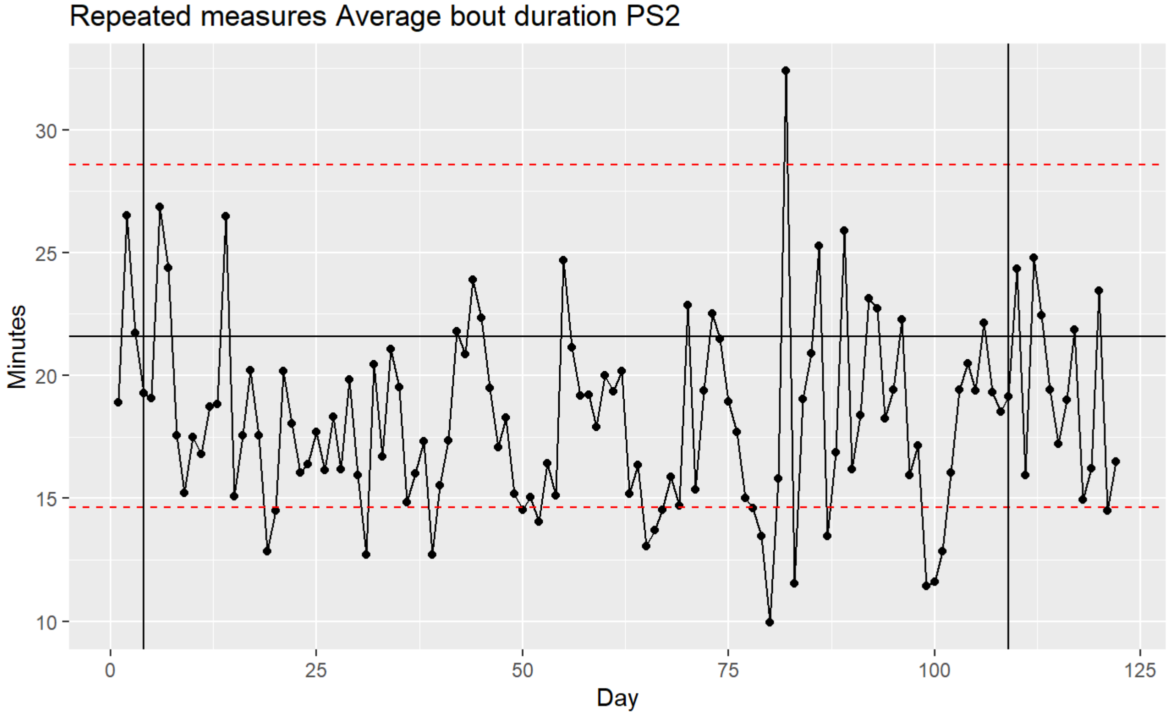

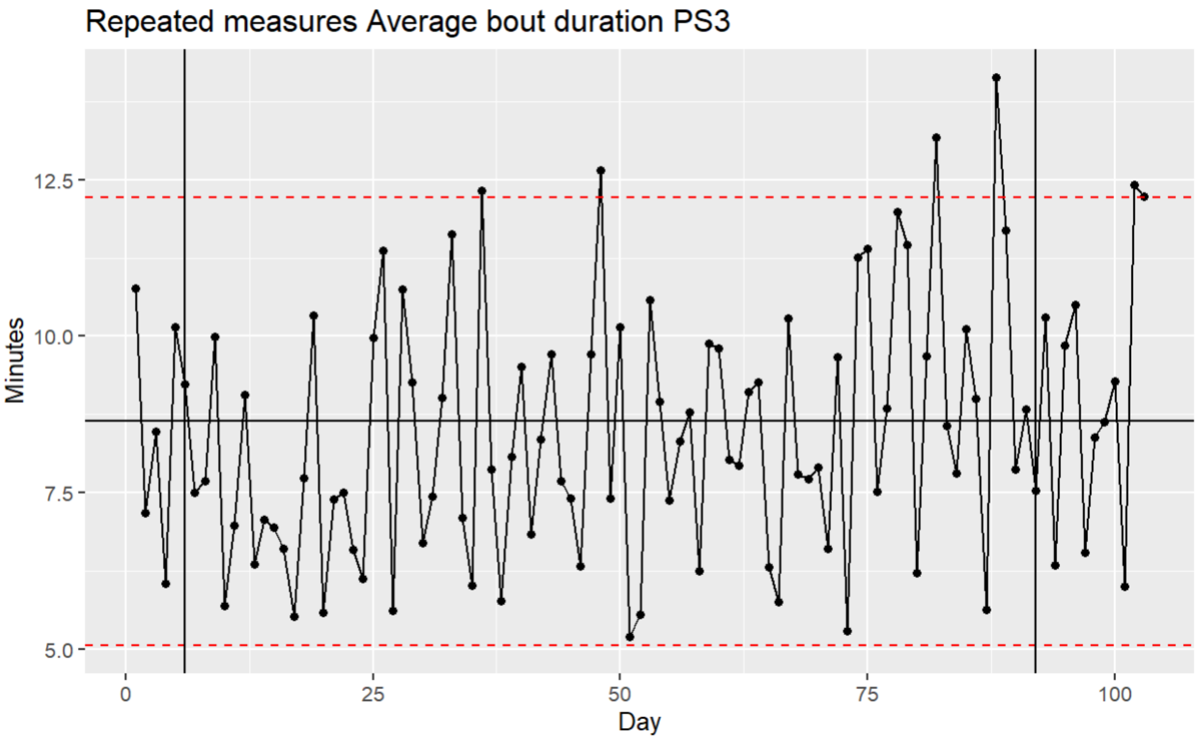
Repeated measures Average bout duration PS3
Minutes
12.5
10.0
7.5
5.0
0
25
50
75
100
Day

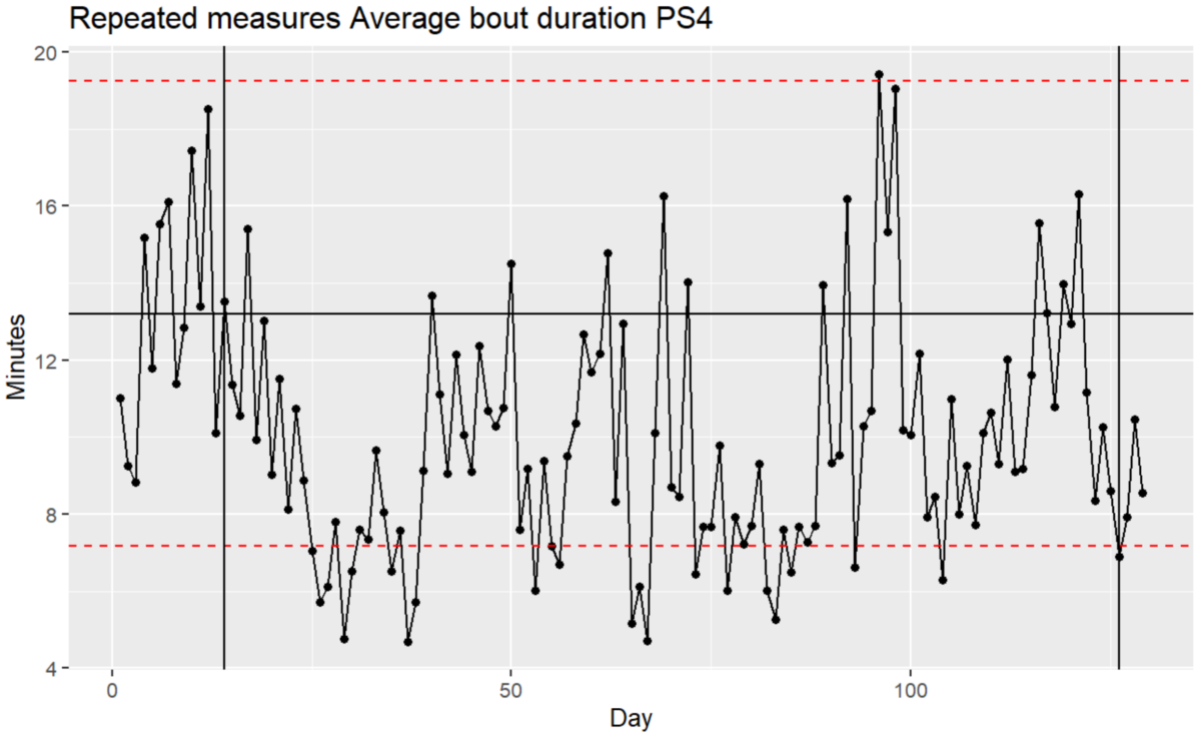
Repeated measures Average bout duration PS4
Minutes
20
16
12
8
4
0
50
100
Day

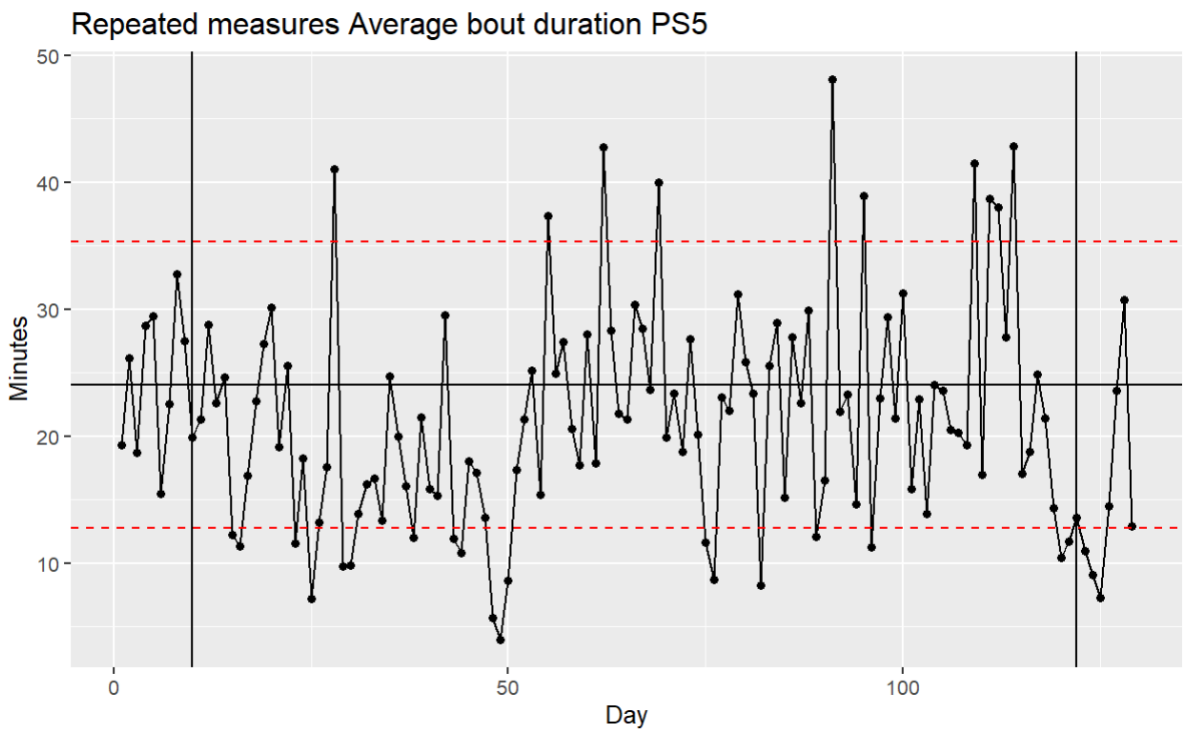

Repeated measures Average bout duration PS5
Minutes
Day
0
50
100
10
20
30
40
50

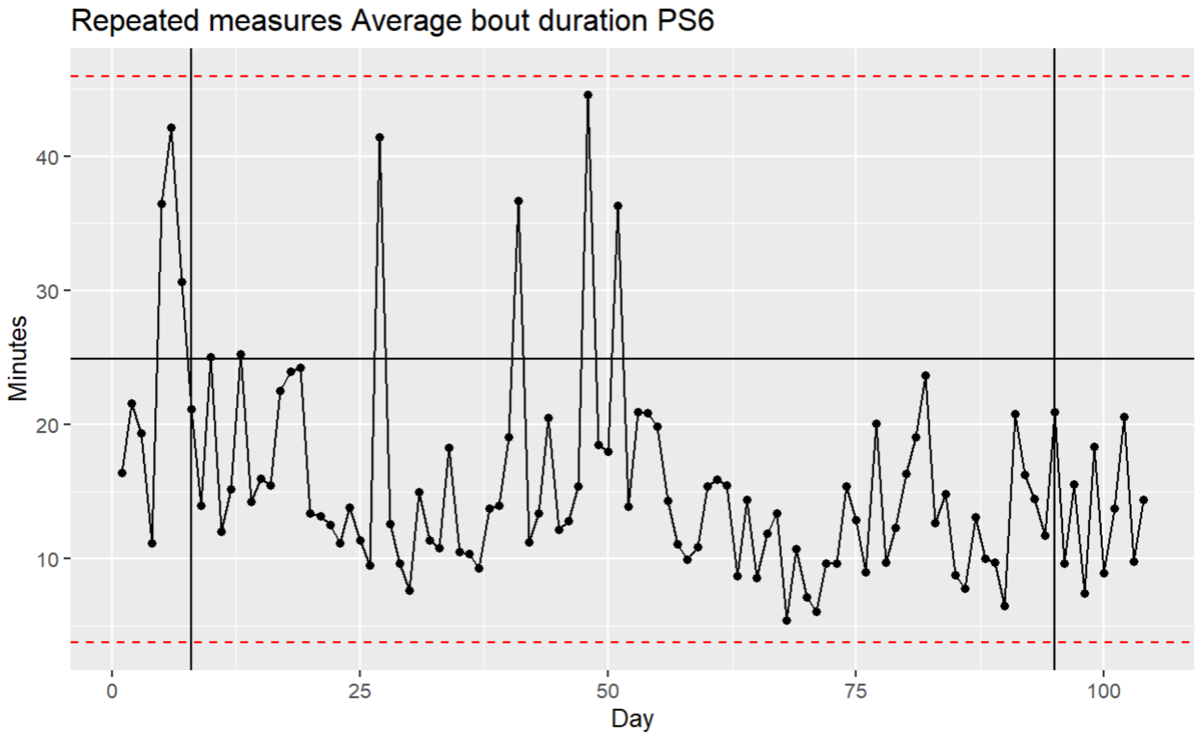

Repeated measures Average bout duration PS6
Minutes
Day
0
25
50
75
100
10
20
30
40

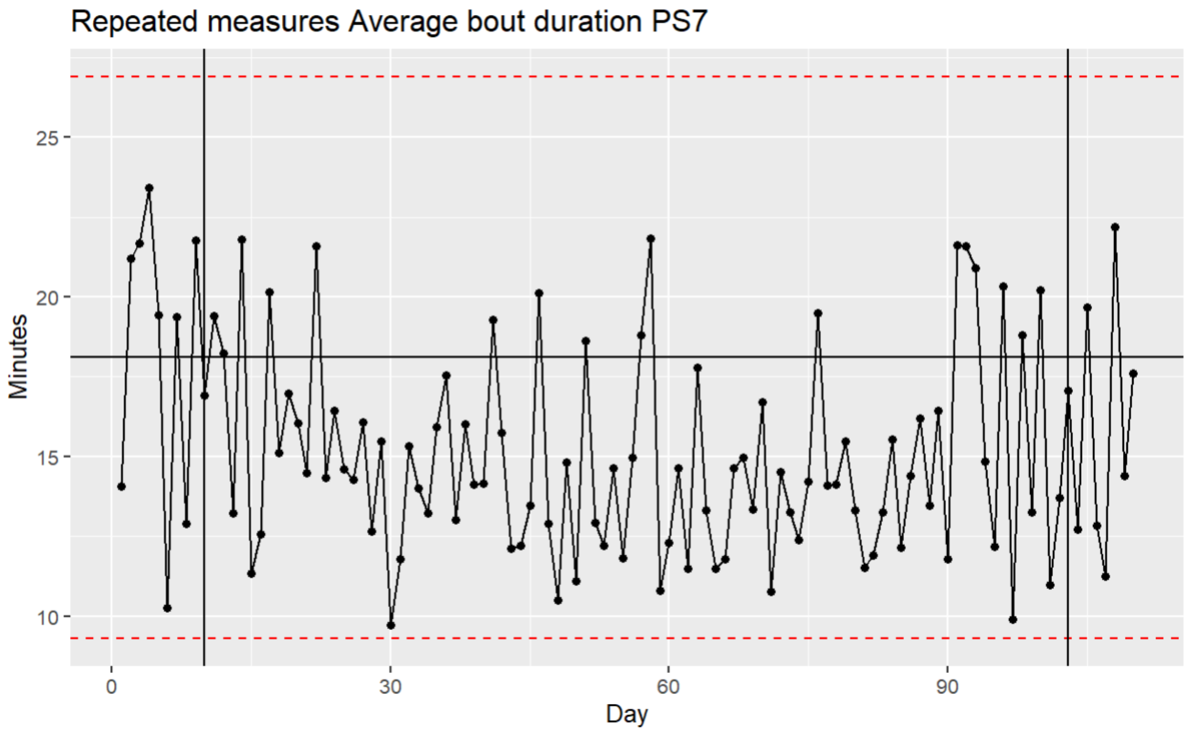

Repeated measures Average bout duration PS7
Minutes
25
20
15
10
0
30
60
90
Day

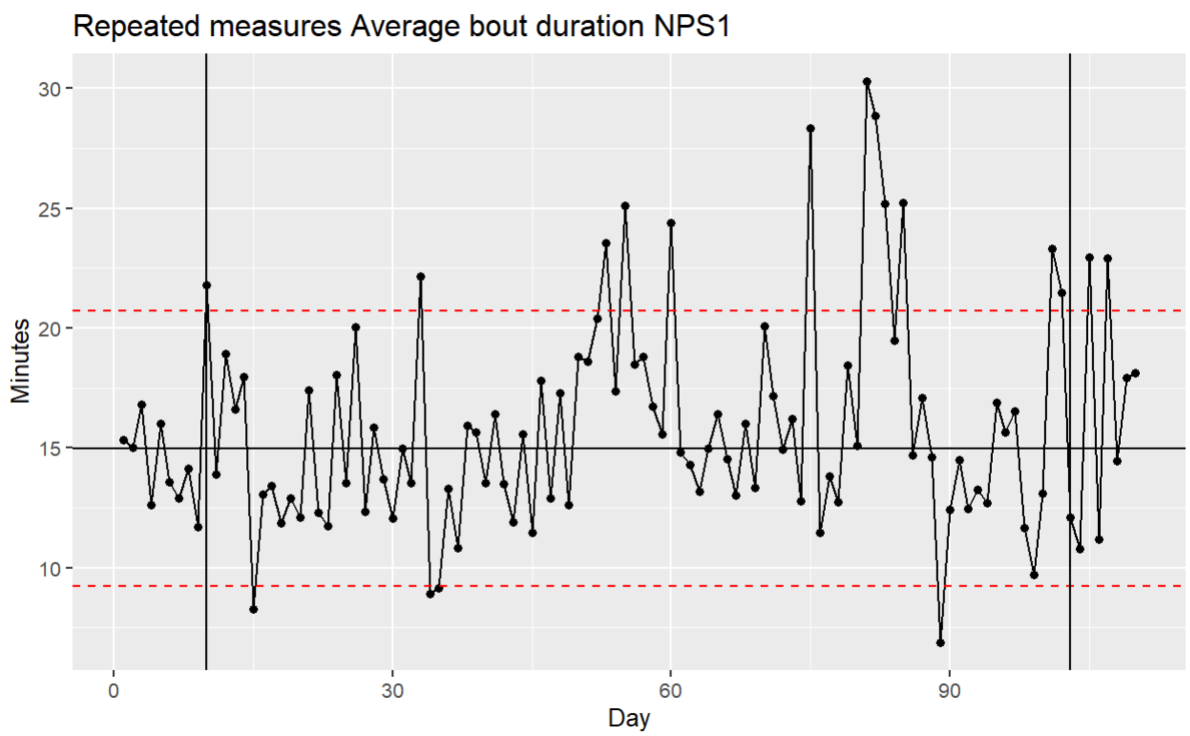
Repeated measures Average bout duration NPS1
Minutes
30
25
20
15
10
0
30
60
90
Day

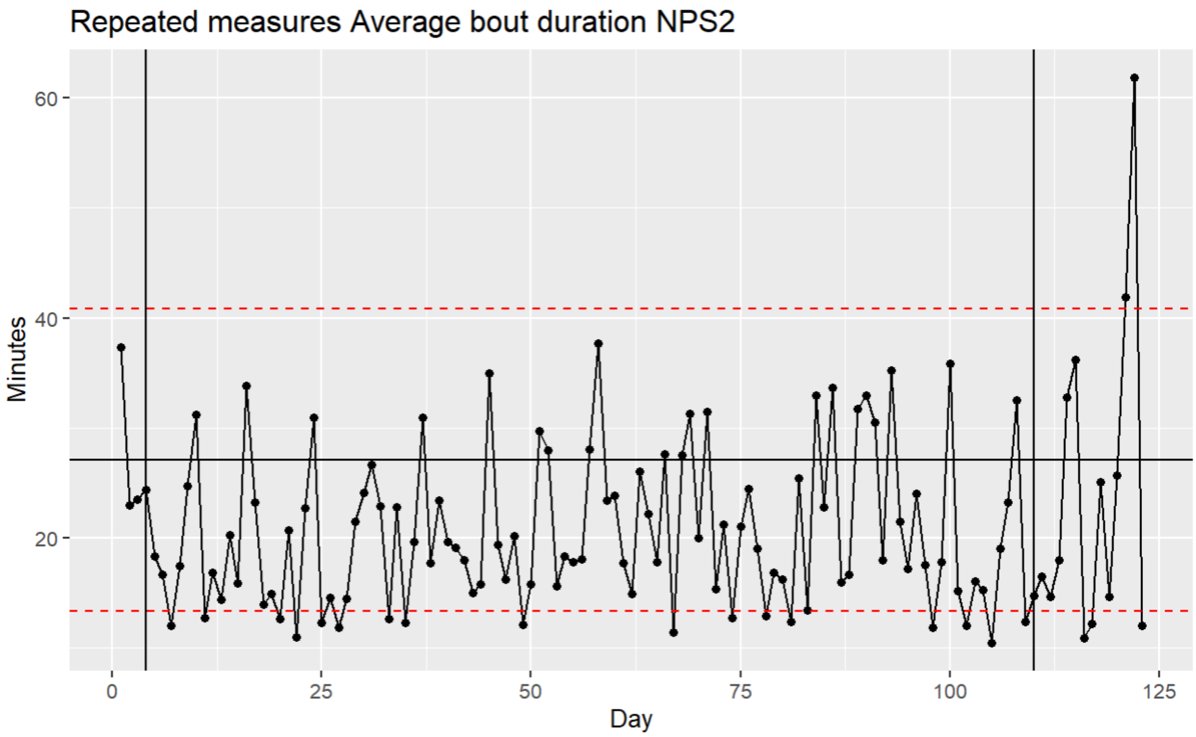
Repeated measures Average bout duration NPS2
Minutes
60
40
20
0
25
50
75
100
125
Day

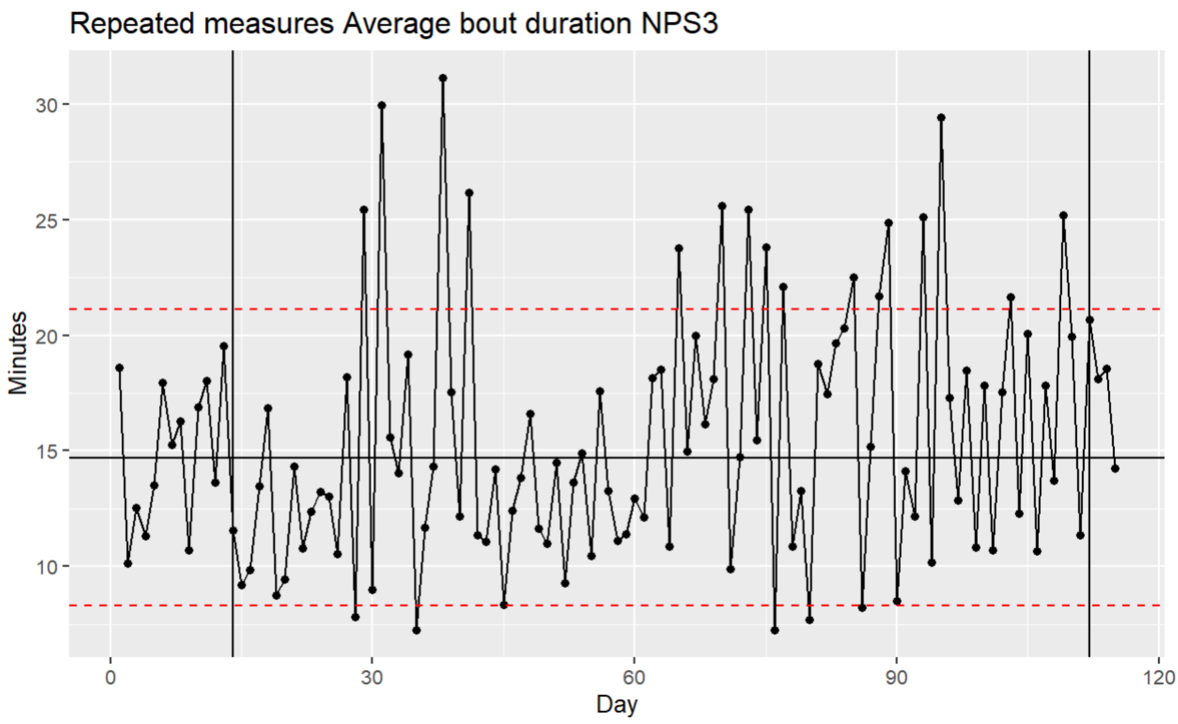
Repeated measures Average bout duration NPS3
Minutes
Day
30
25
20
15
10
0
30
60
90
120

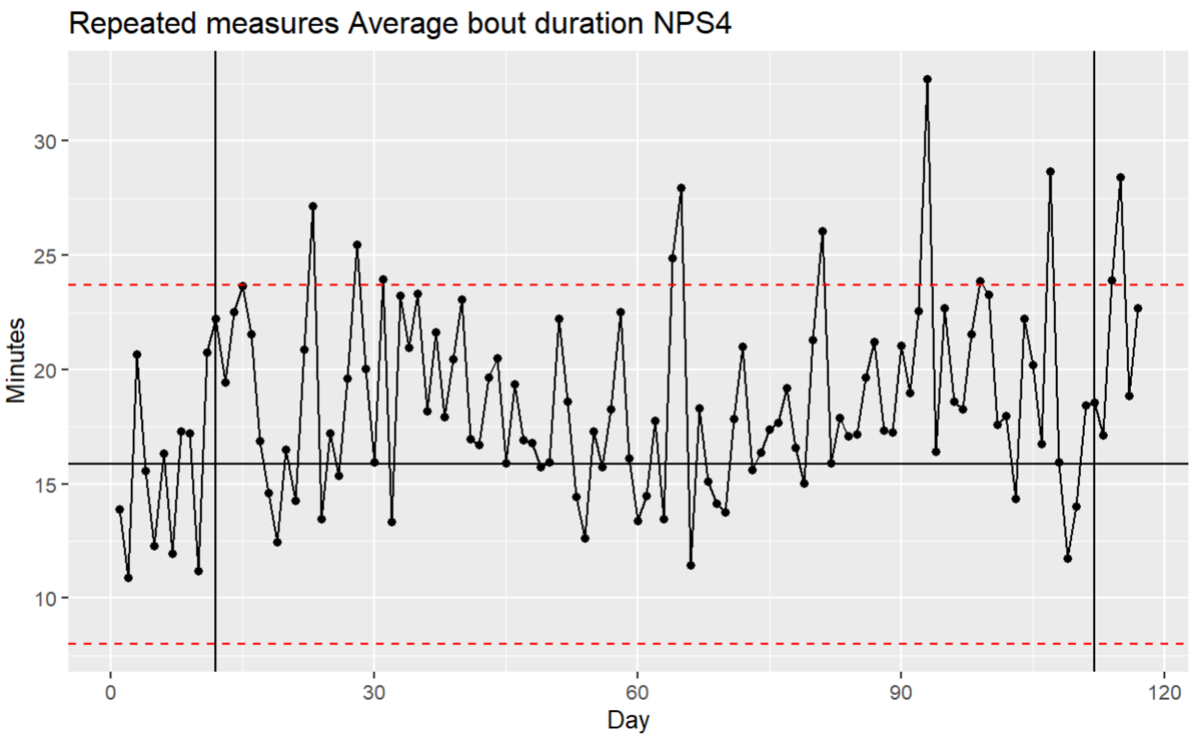
Repeated measures Average bout duration NPS4
Minutes
Day
30
25
20
15
10
0
30
60
90
120

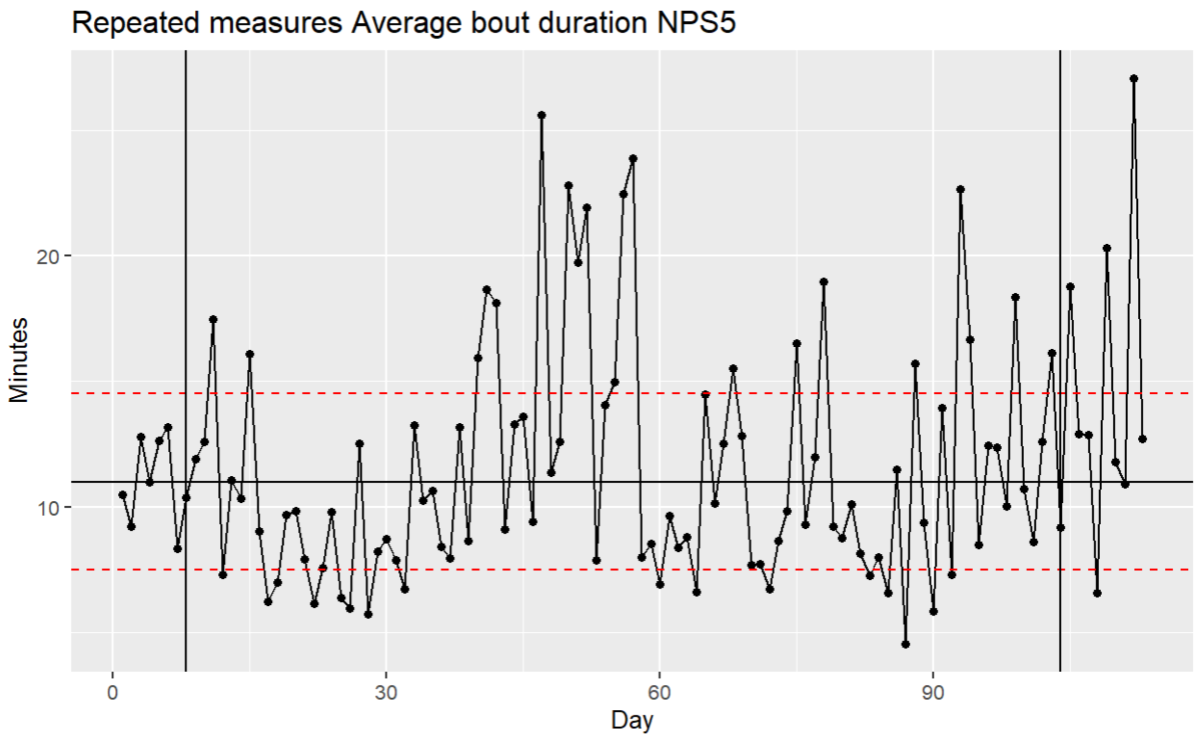
Repeated measures Average bout duration NPS5
Minutes
20
10
0
30
60
90
Day

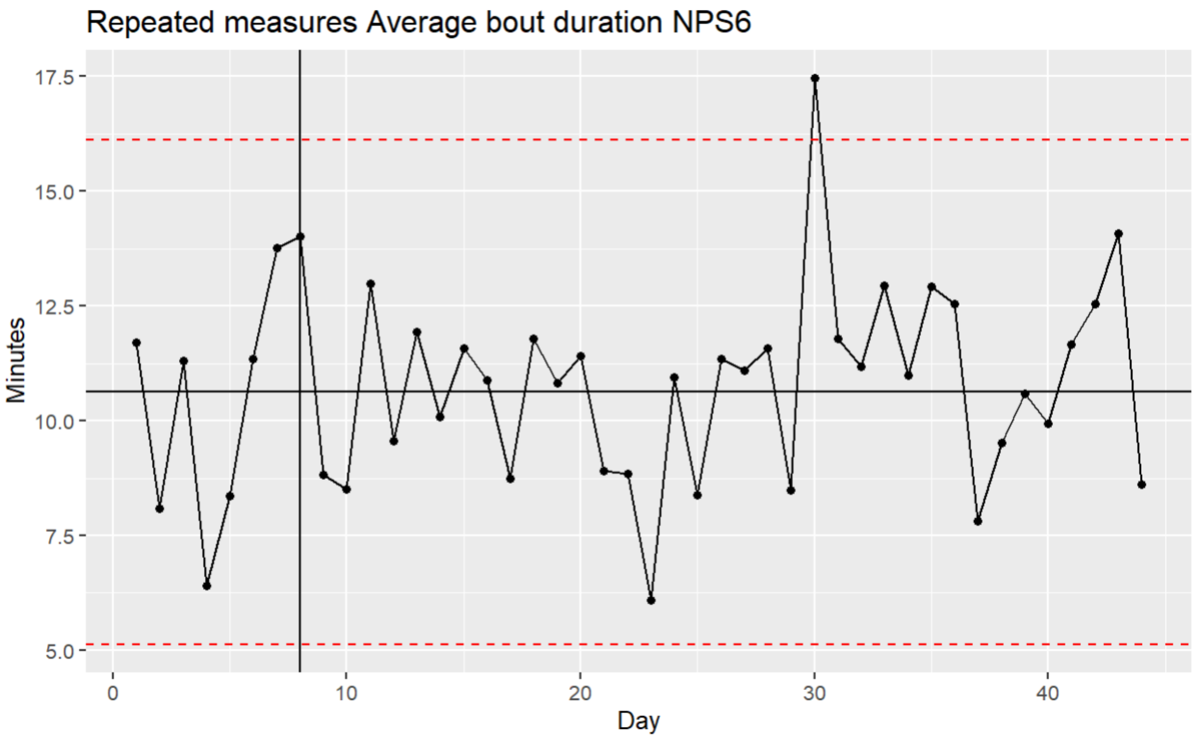
Repeated measures Average bout duration NPS6
Minutes
17.5
15.0
12.5
10.0
7.5
5.0
0
10
20
30
40
Day

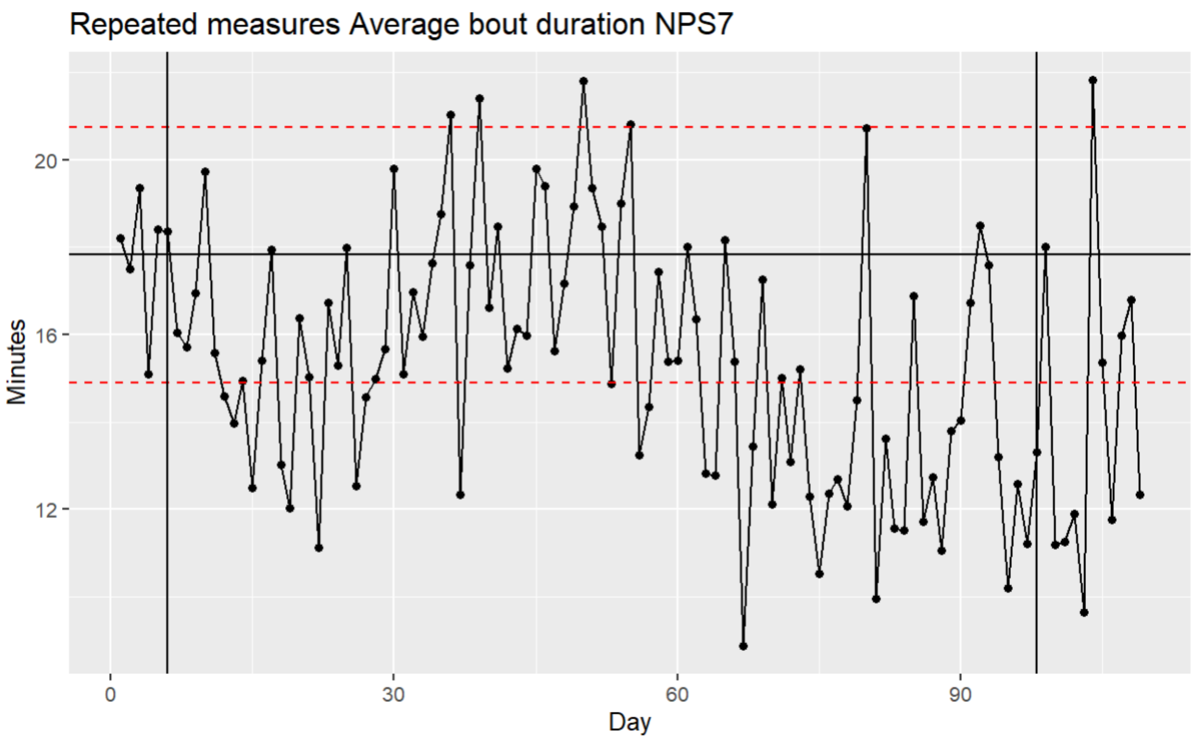
Repeated measures Average bout duration NPS7
Minutes
20
16
12
0
30
60
90
Day

__**Time spend in Light Physical Activity (LPA)**__

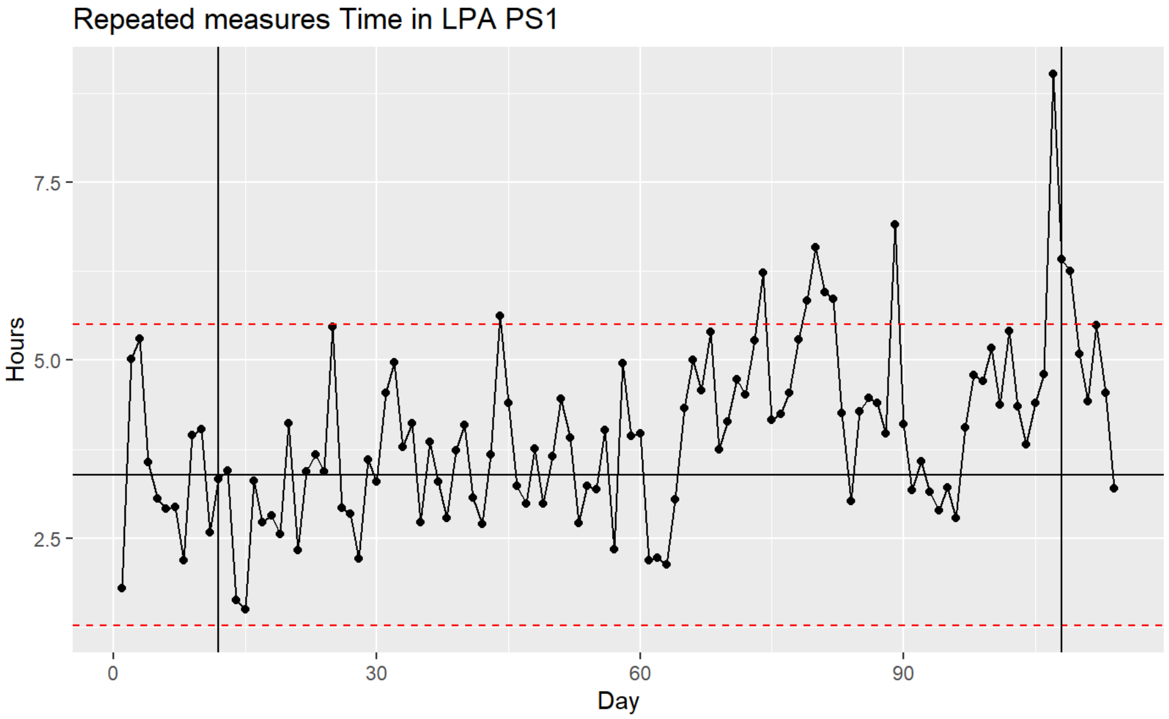

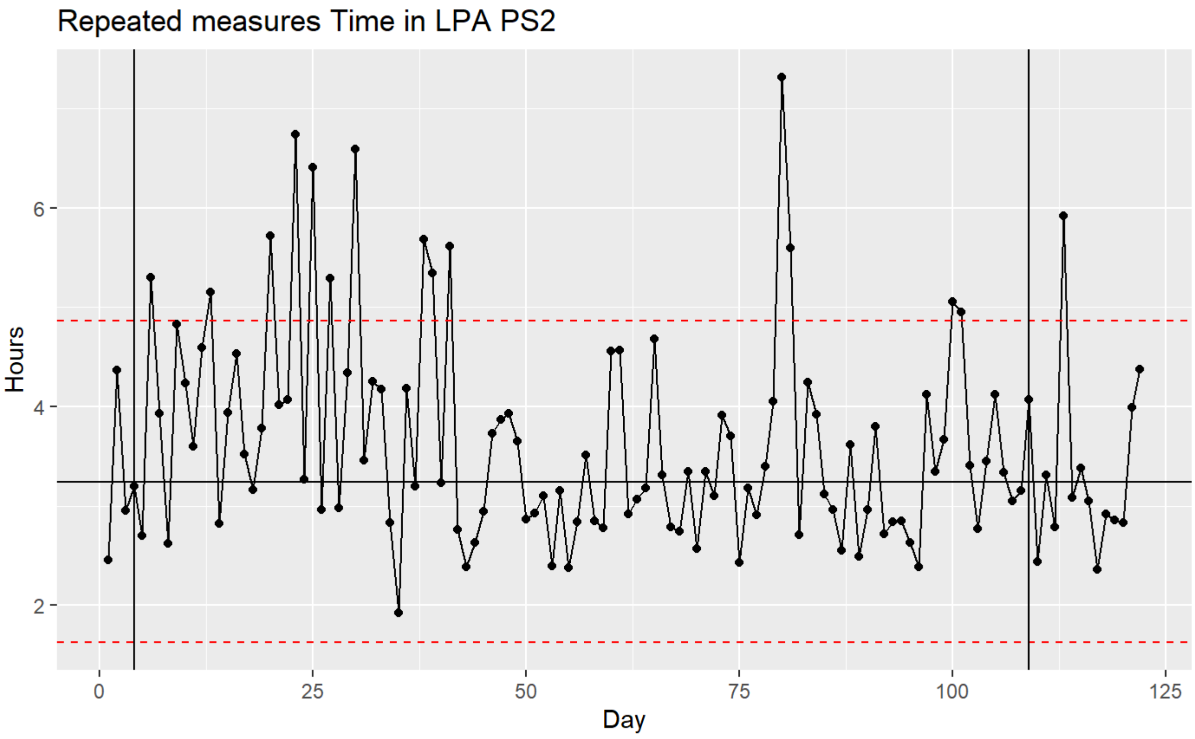

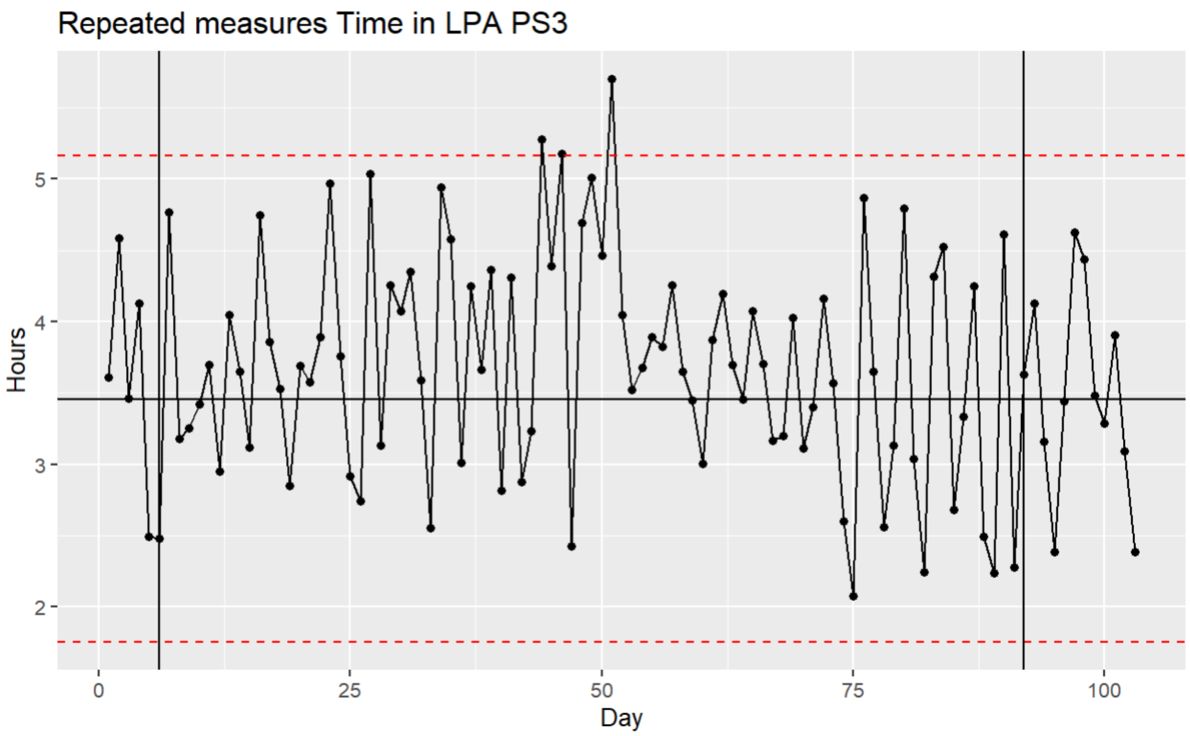
Repeated measures Time in LPA PS3
Hours
Day
2
3
4
5
0
25
50
75
100

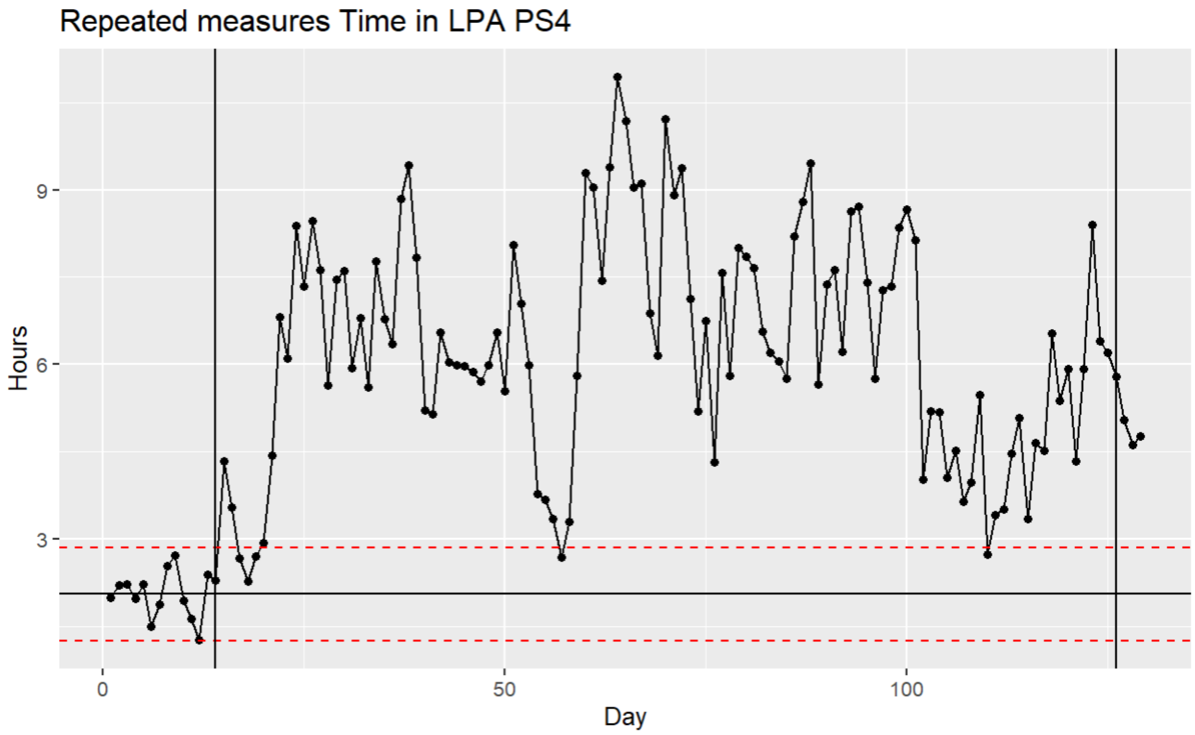
Repeated measures Time in LPA PS4
Hours
Day
3
6
9
0
50
100

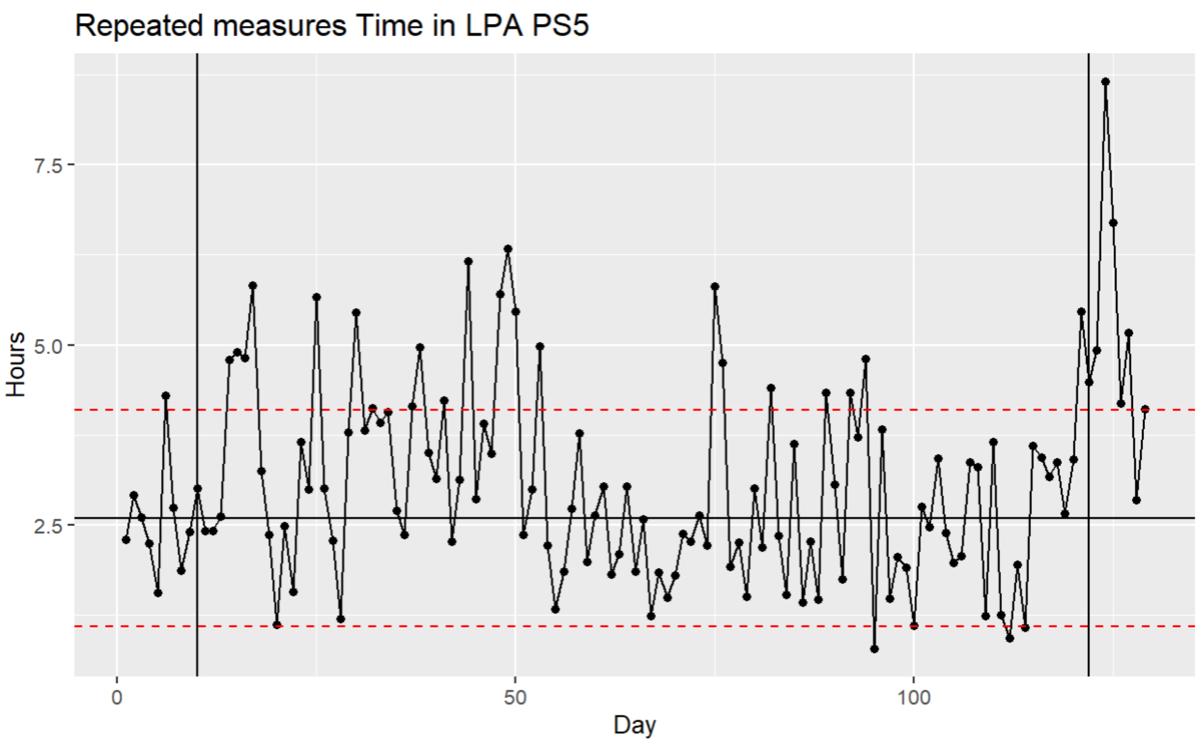
Repeated measures Time in LPA PS5
Hours
7.5
5.0
2.5
0
50
100
Day

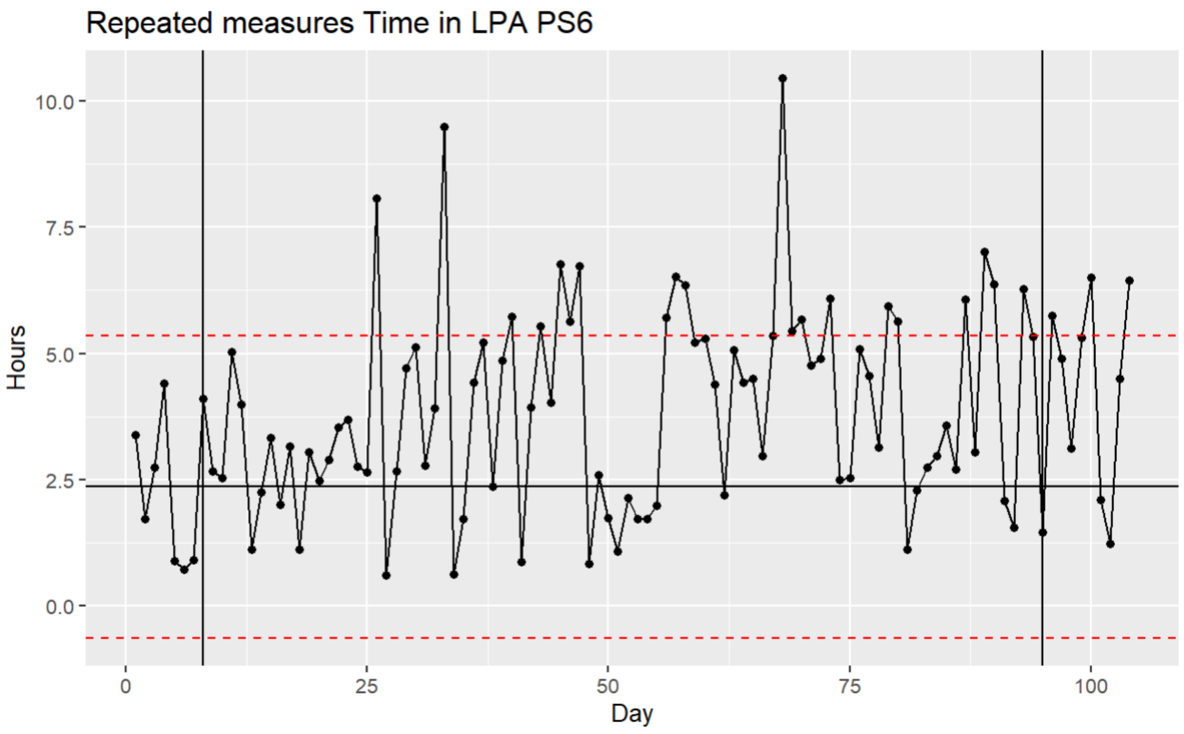
Repeated measures Time in LPA PS6
Hours
10.0
7.5
5.0
2.5
0.0
0
25
50
75
100
Day

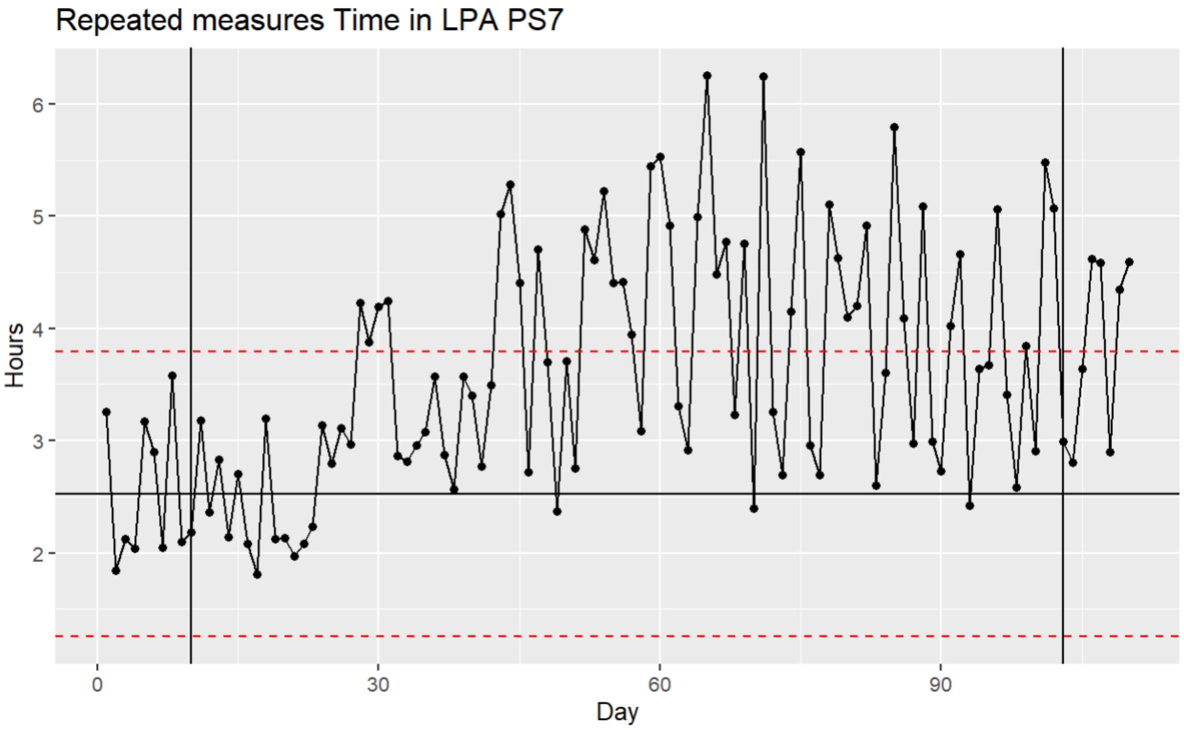
Repeated measures Time in LPA PS7
Hours
Day
6
5
4
3
2
0
30
60
90

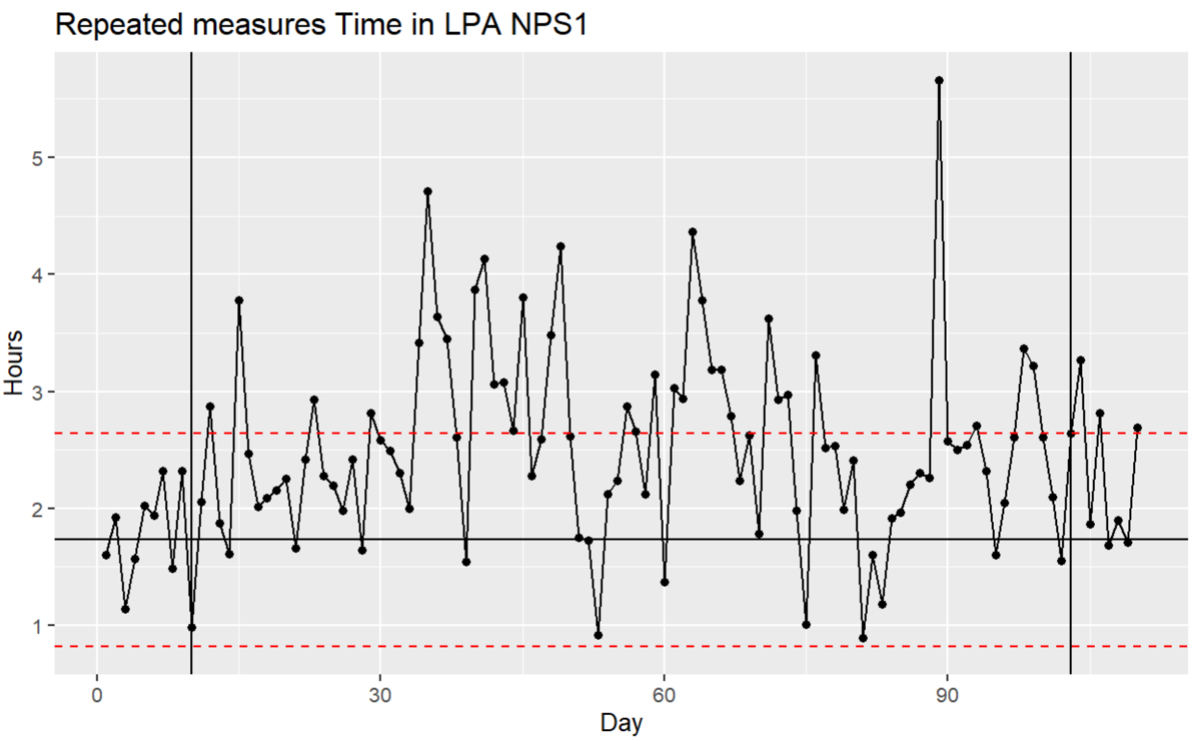
Repeated measures Time in LPA NPS1
Hours
Day
5
4
3
2
1
0
30
60
90

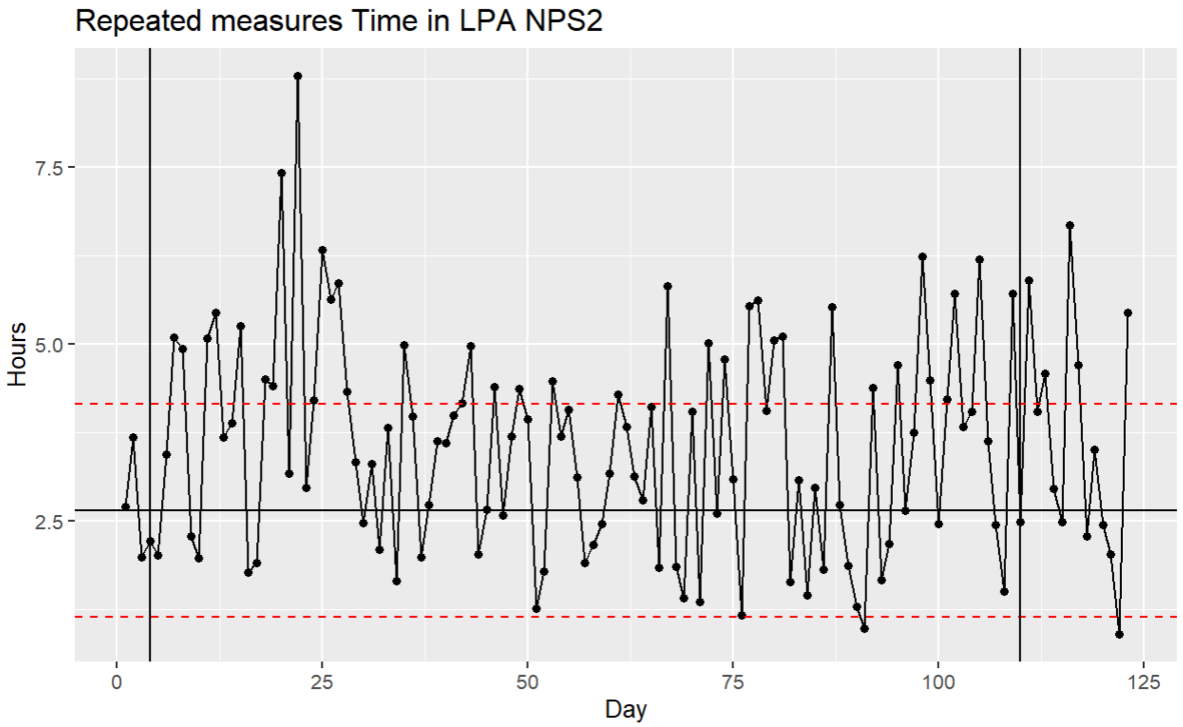
Repeated measures Time in LPA NPS2
Hours
Day
7.5
5.0
2.5
0
25
50
75
100
125

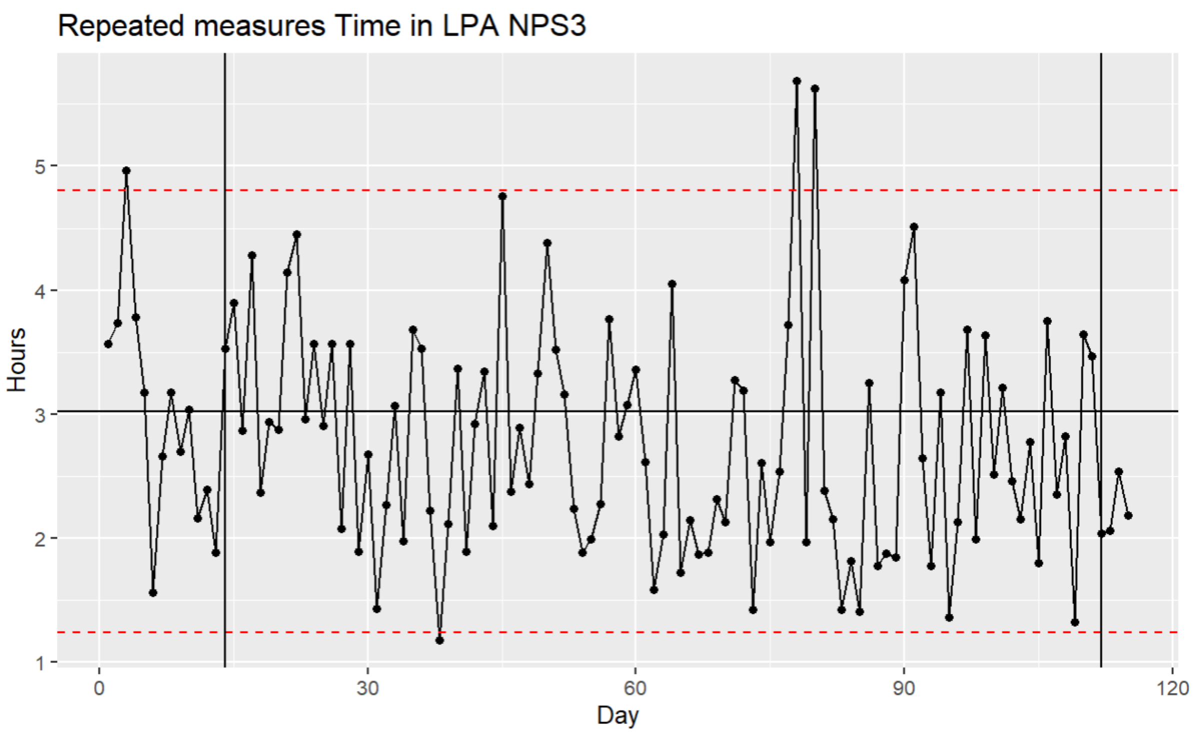

Repeated measures Time in LPA NPS3
Hours
Day
1
2
3
4
5
0
30
60
90
120

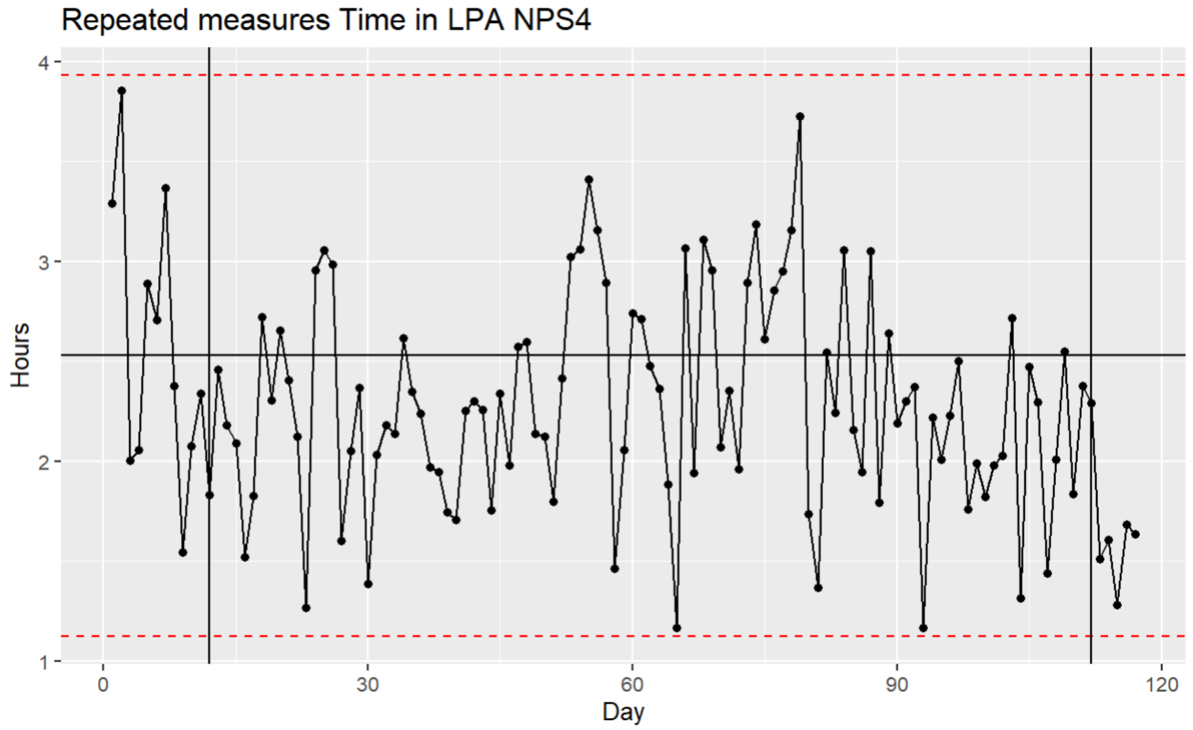

Repeated measures Time in LPA NPS4
Hours
Day
1
2
3
4
0
30
60
90
120

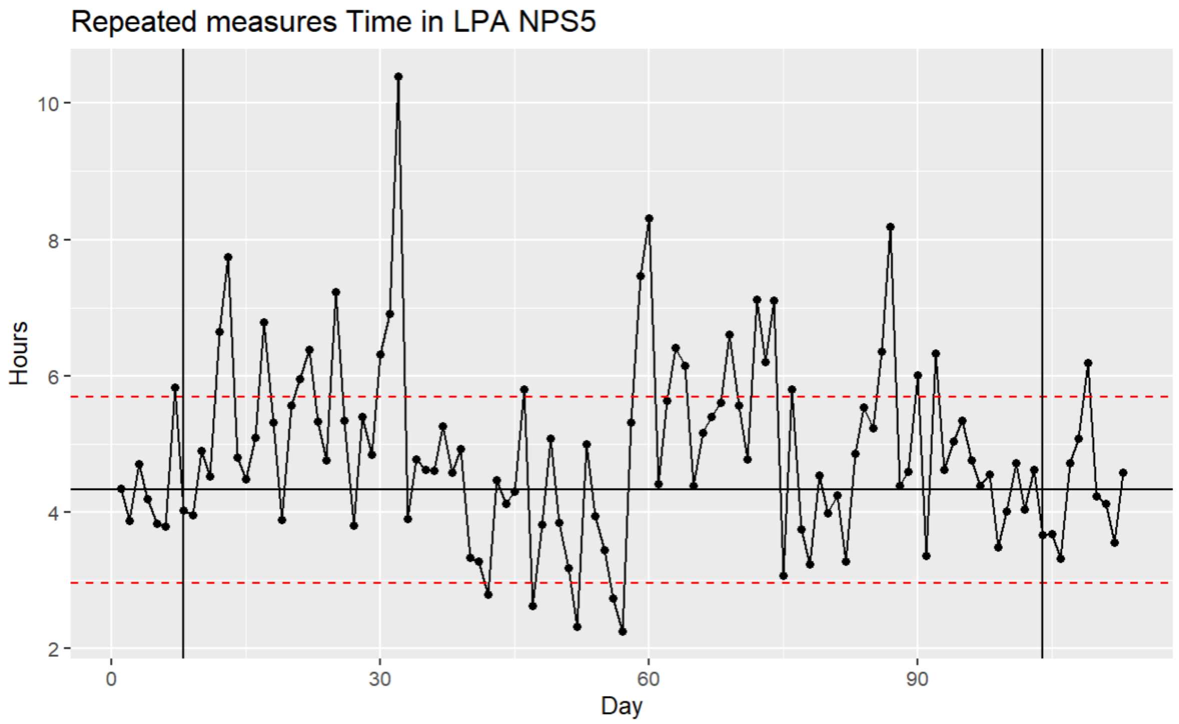
Repeated measures Time in LPA NPS5
Hours
Day
10
8
6
4
2
0
30
60
90

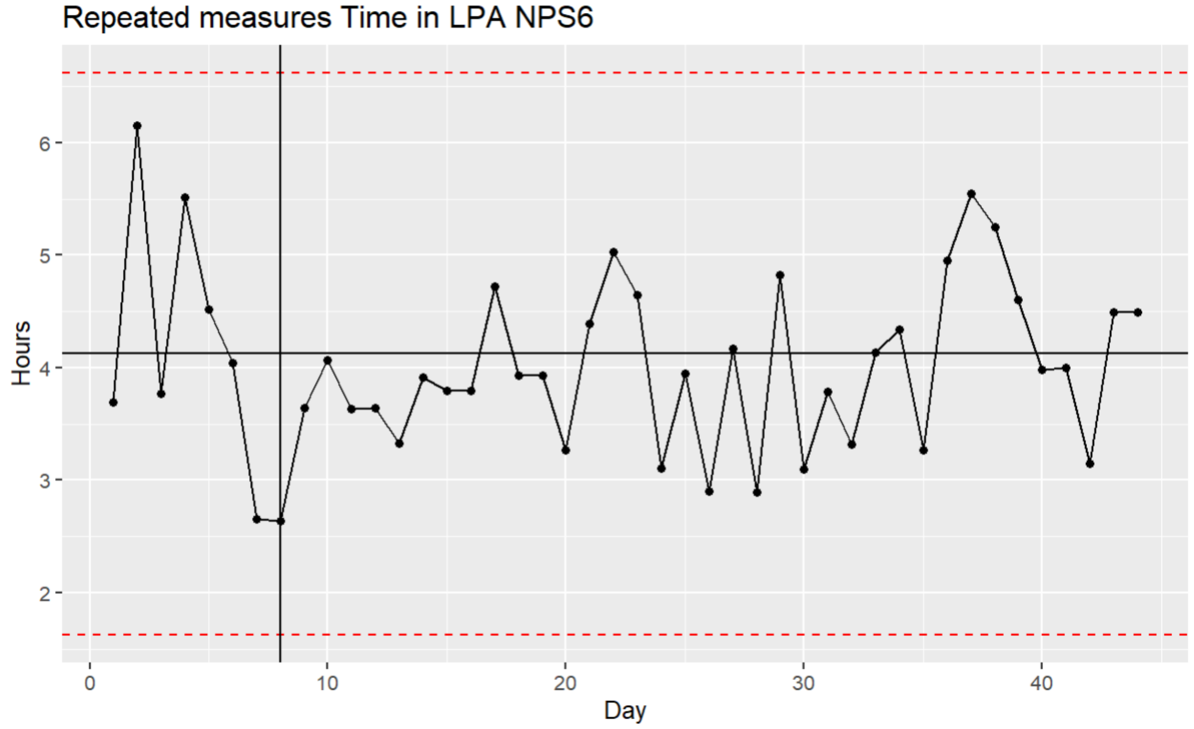
Repeated measures Time in LPA NPS6
Hours
Day
6
5
4
3
2
0
10
20
30
40

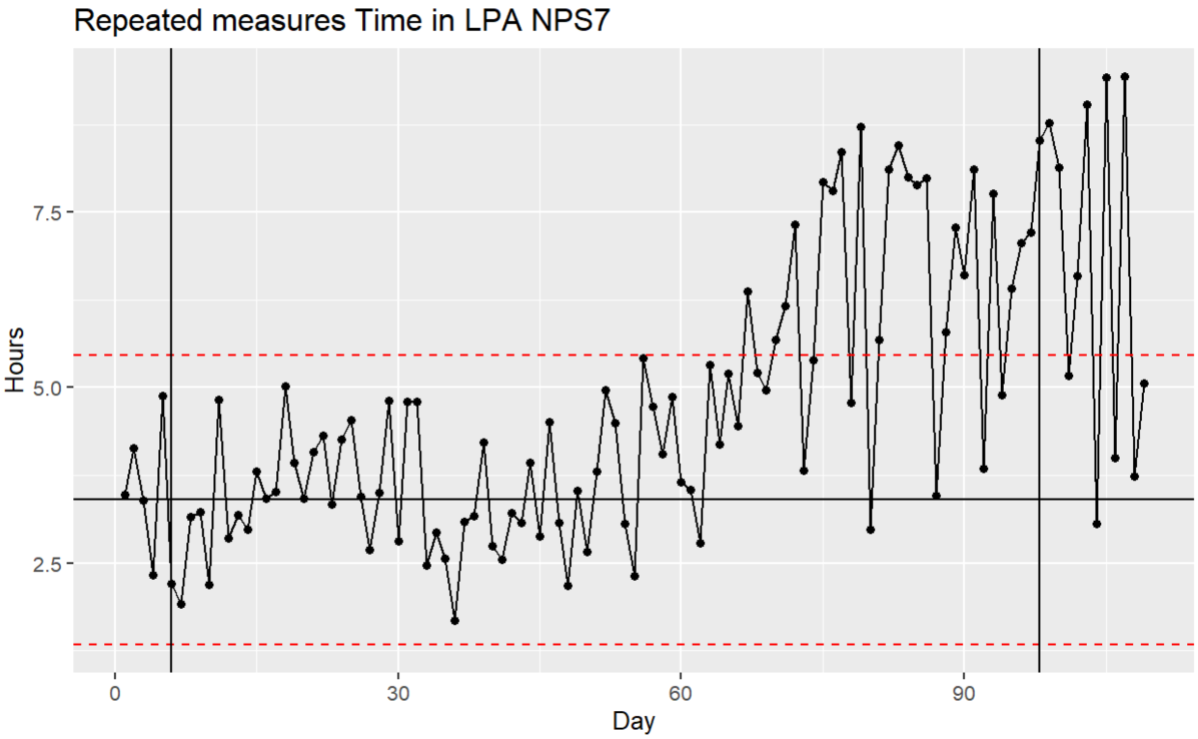

Repeated measures Time in LPA NPS7
Hours
7.5
5.0
2.5
0
30
60
90
Day

Time spend in Moderate to Vigorous Physical Activity (MVPA)

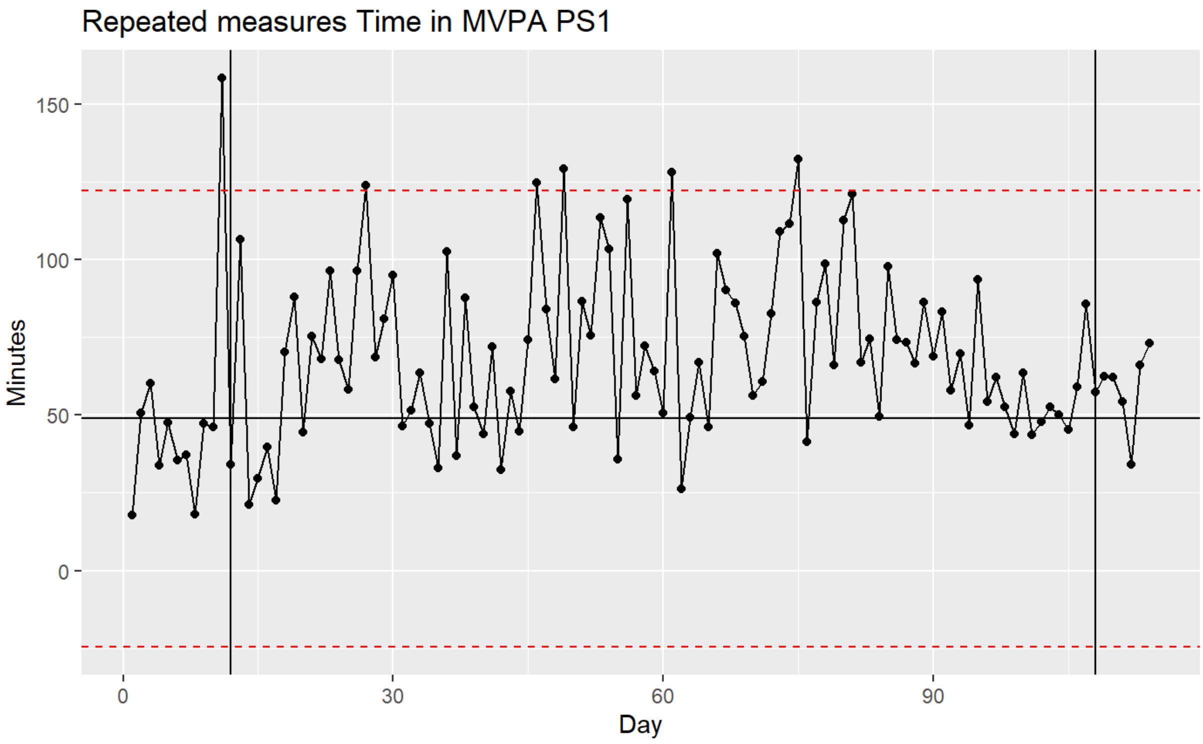

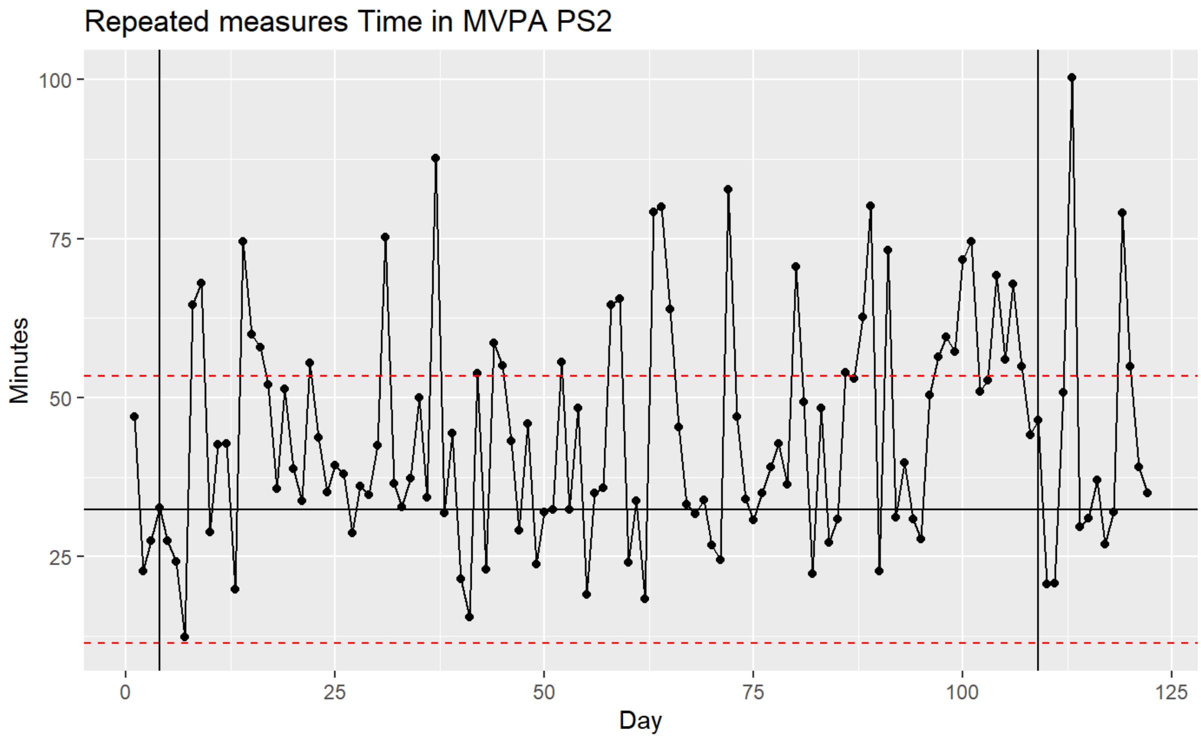

Repeated measures Time in MVPA PS3

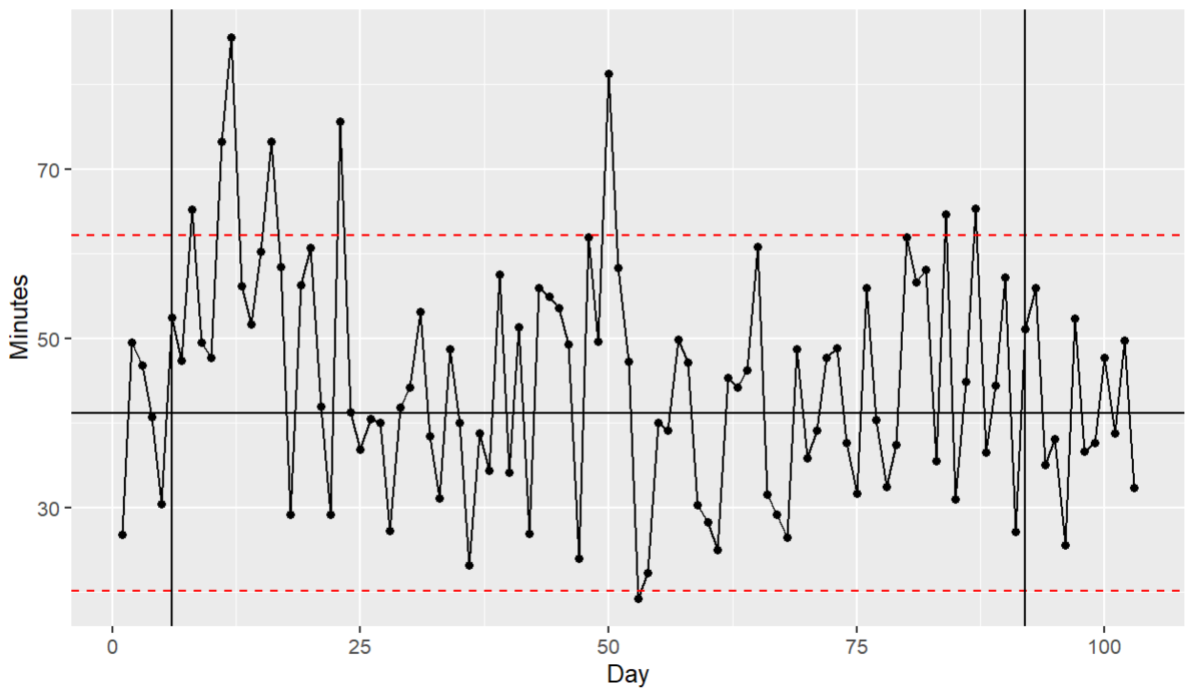

Repeated measures Time in MVPA PS4

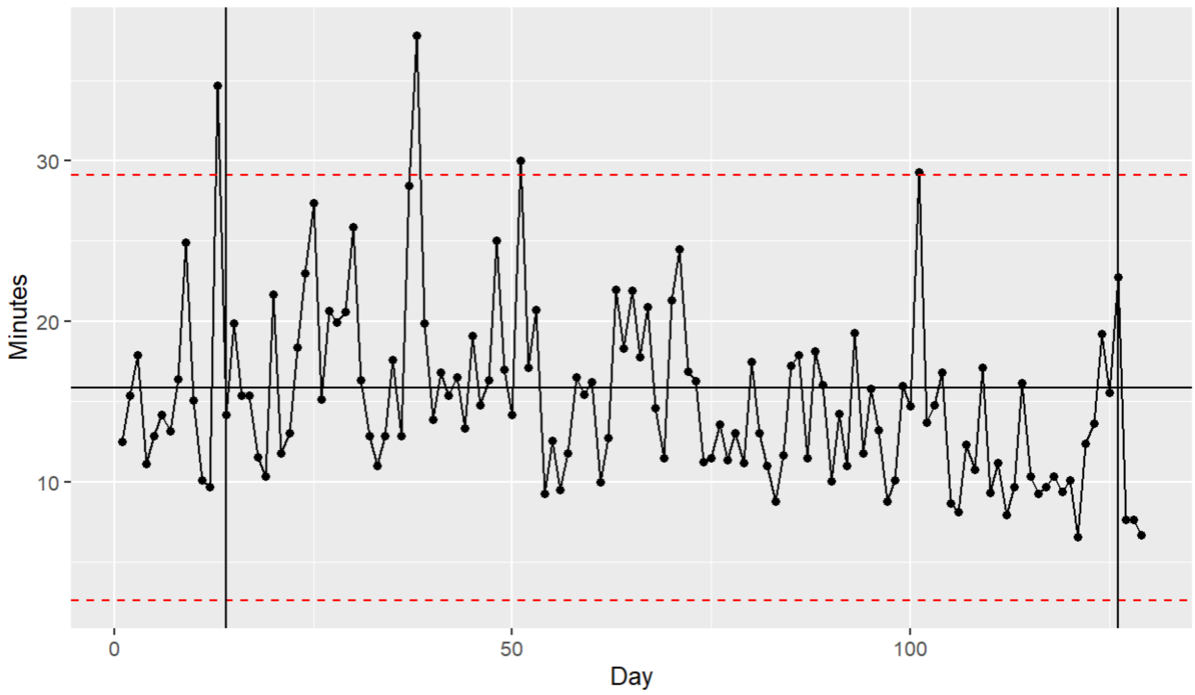

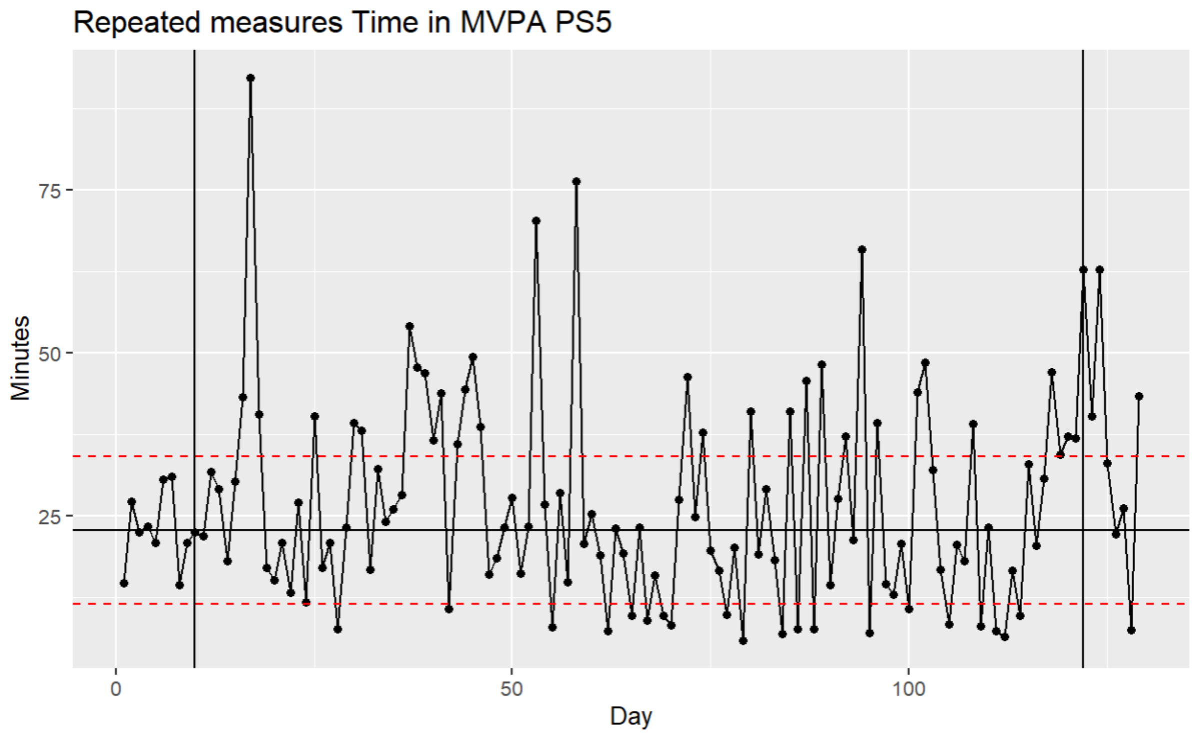
Repeated measures Time in MVPA PS5
Minutes
75
50
25
0
50
100
Day

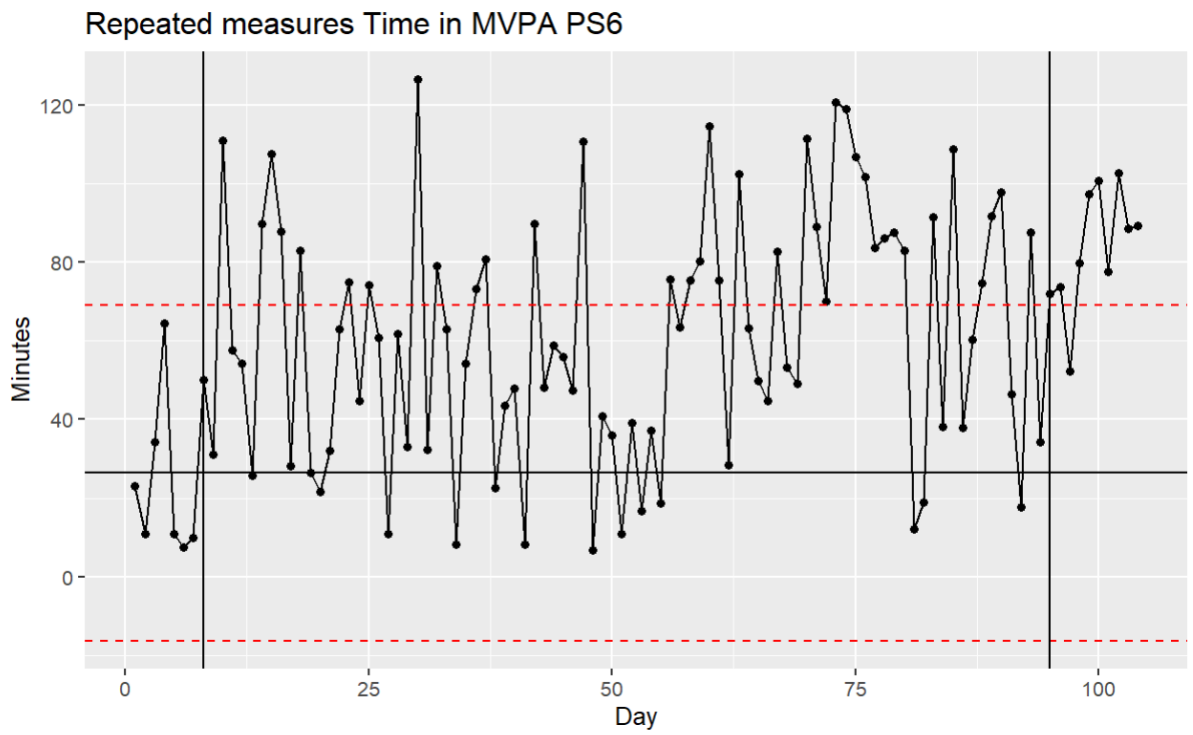
Repeated measures Time in MVPA PS6
Minutes
120
80
40
0
0
25
50
75
100
Day

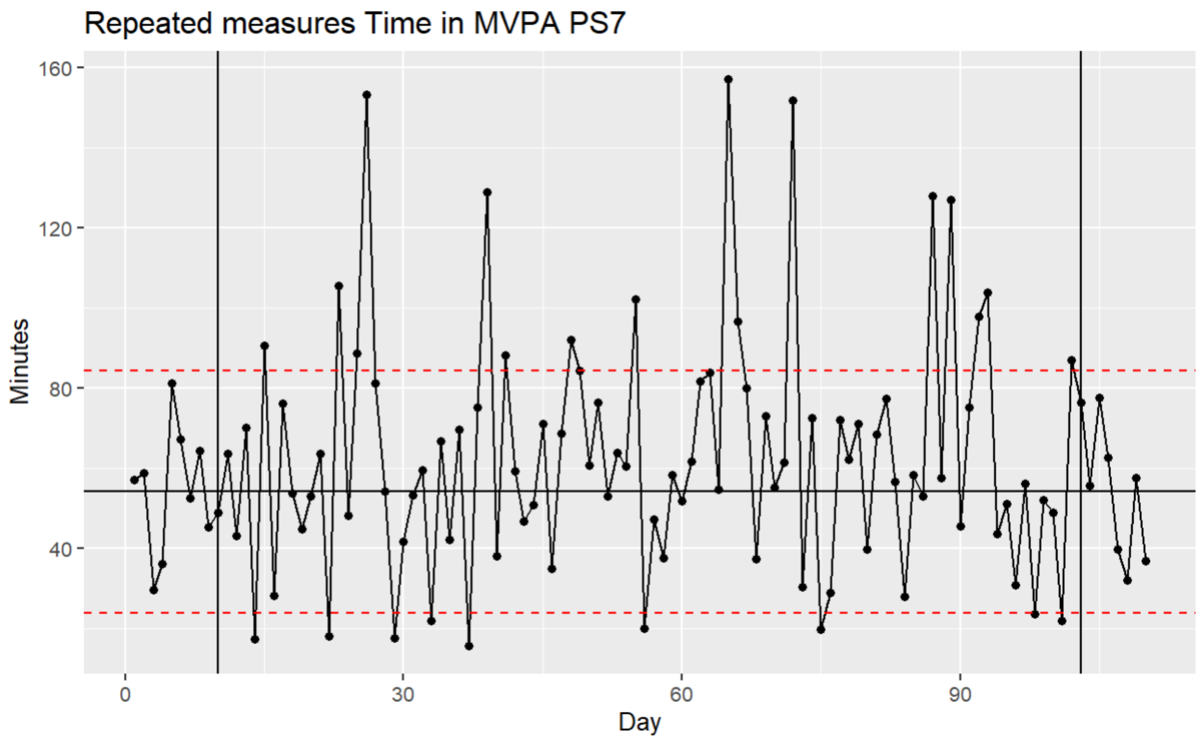

Repeated measures Time in MVPA PS7
Minutes
160
120
80
40
0
30
60
90
Day

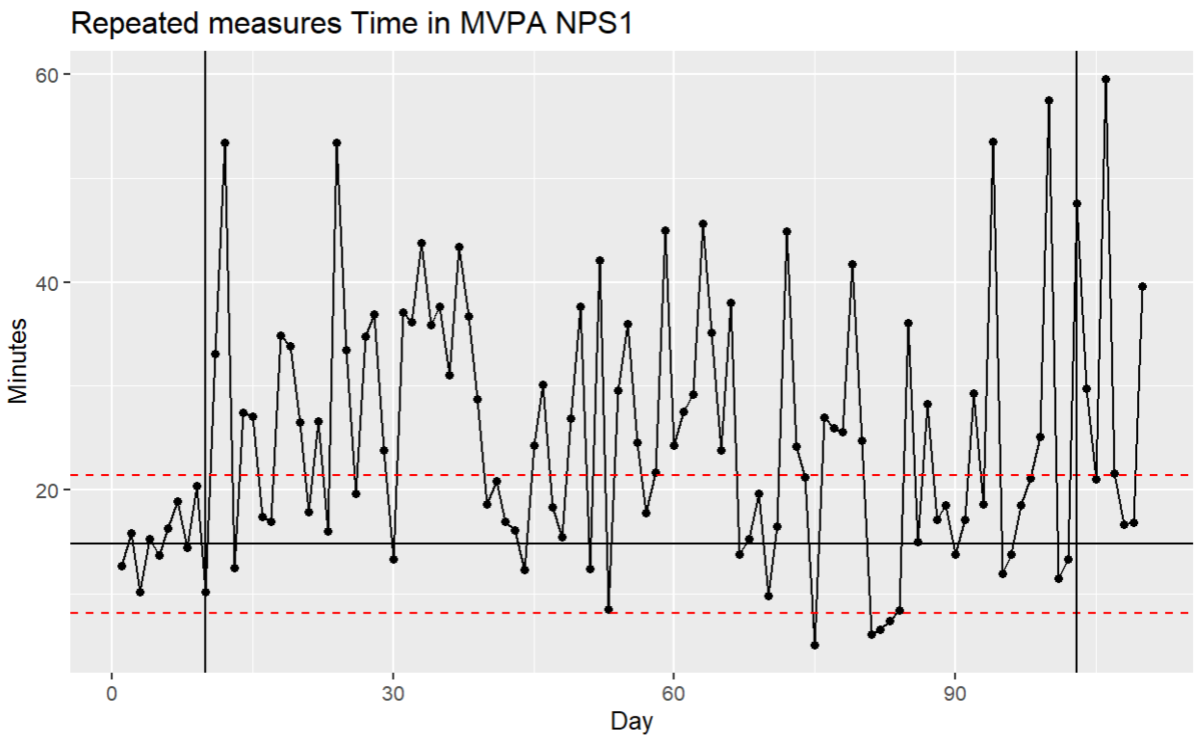

Repeated measures Time in MVPA NPS1
Minutes
Day
60
40
20
0
30
60
90

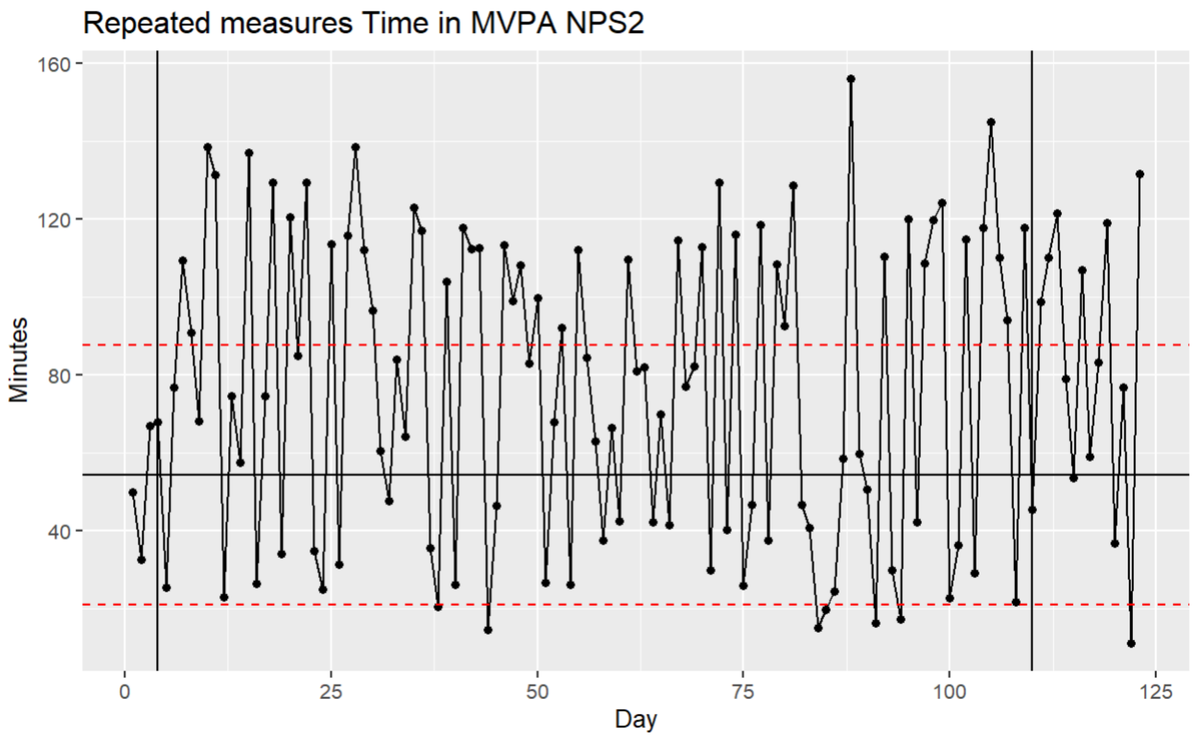

Repeated measures Time in MVPA NPS2
Minutes
Day
160
120
80
40
0
25
50
75
100
125

Repeated measures Time in MVPA NPS3

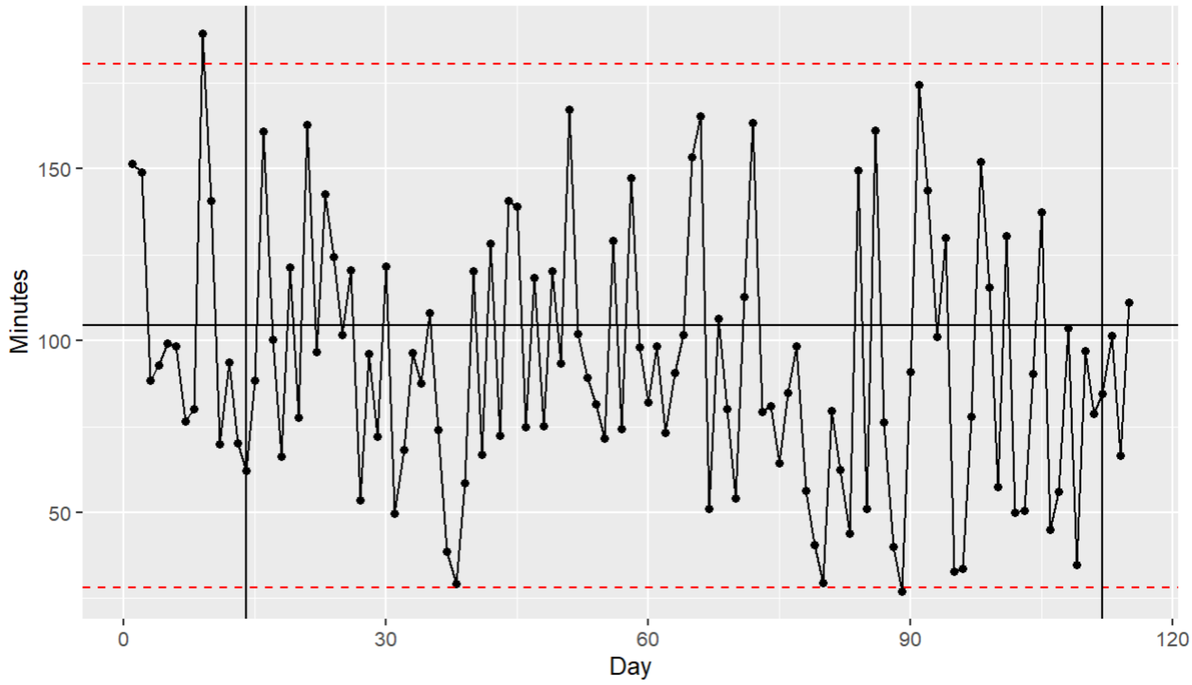

Repeated measures Time in MVPA NPS4

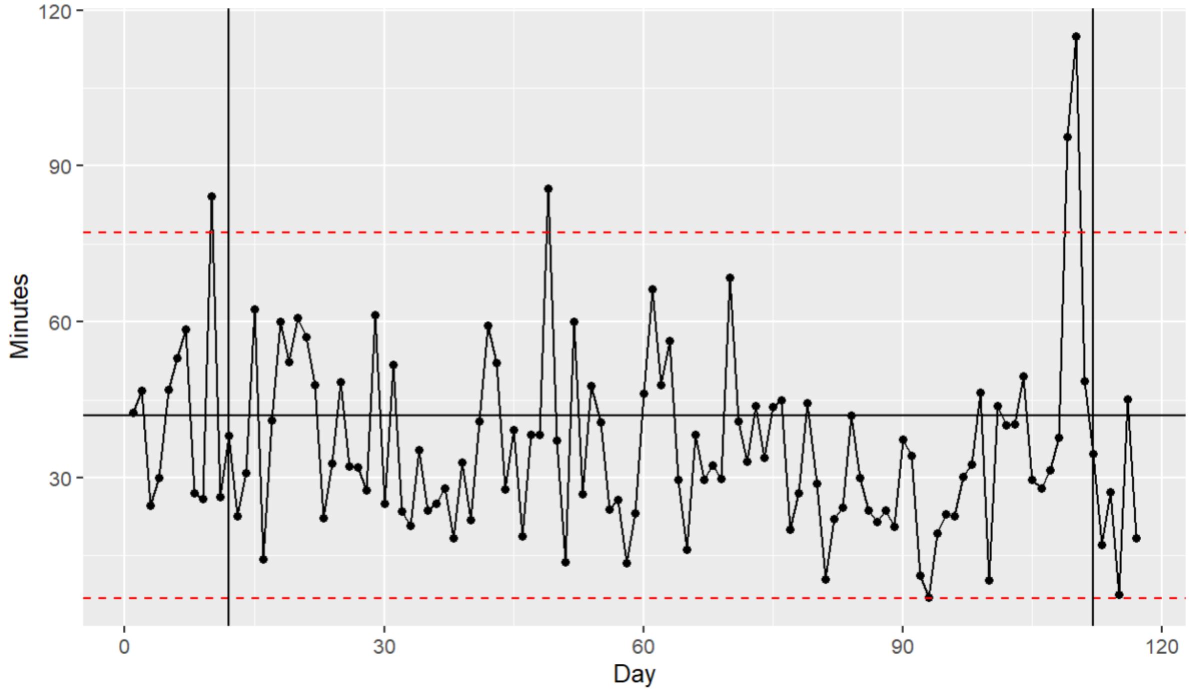

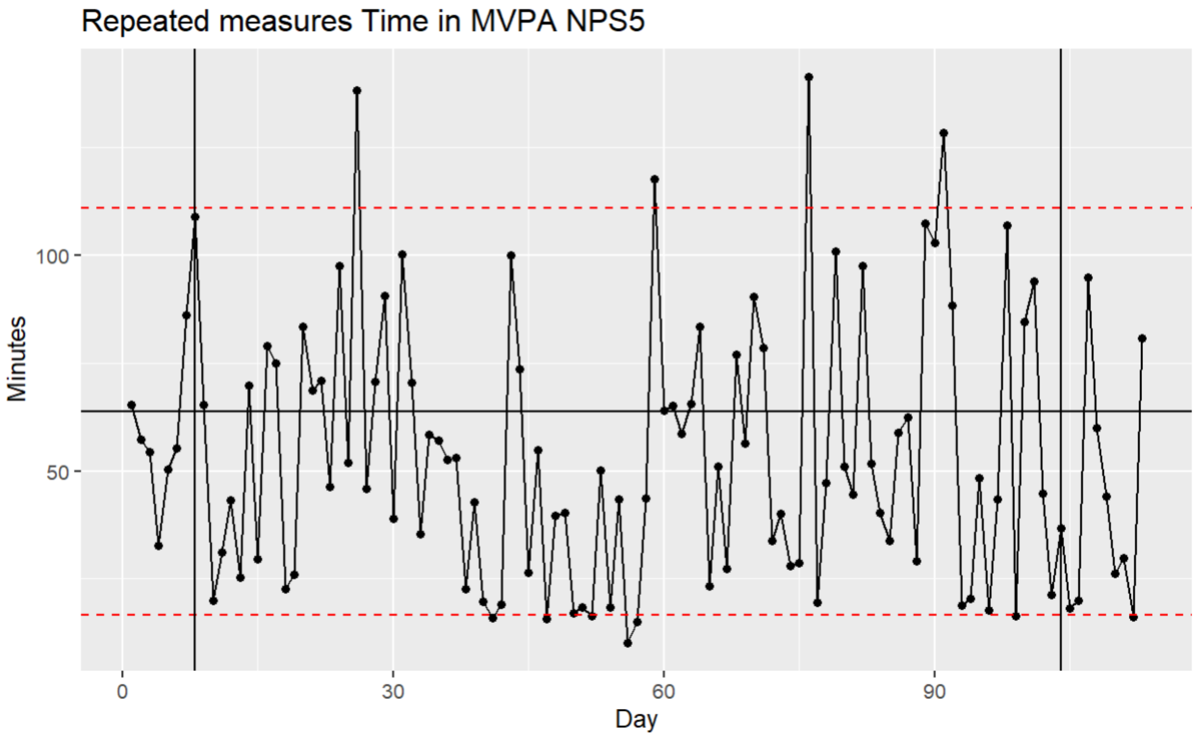

Repeated measures Time in MVPA NPS5
Minutes
100
50
0
30
60
90
Day

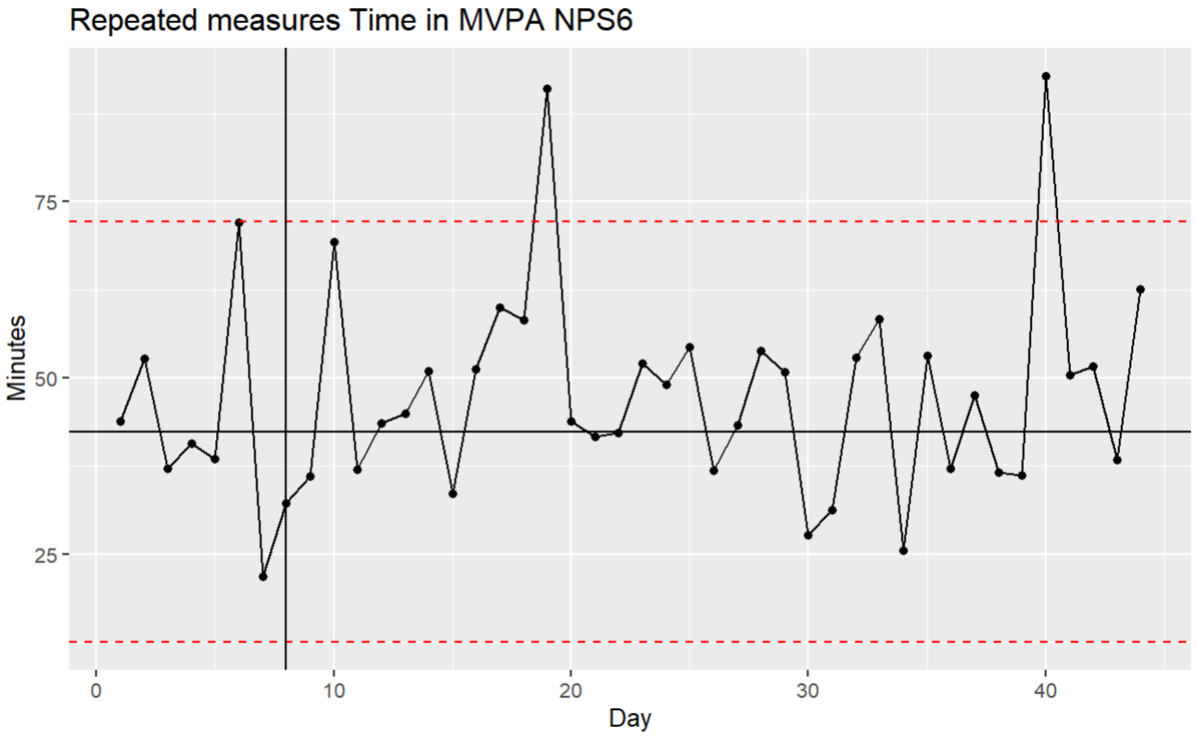

Repeated measures Time in MVPA NPS6
Minutes
75
50
25
0
10
20
30
40
Day

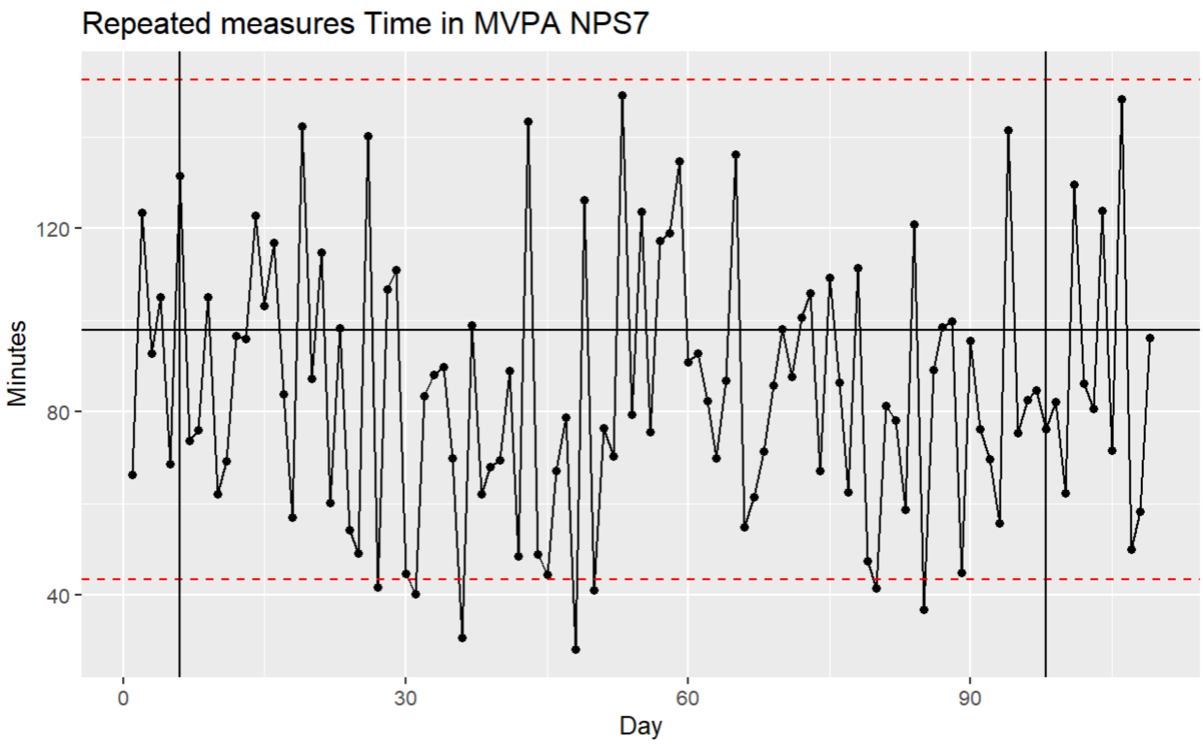
Repeated measures Time in MVPA NPS7
Minutes
120
80
40
0
30
60
90
Day

Time spend standing

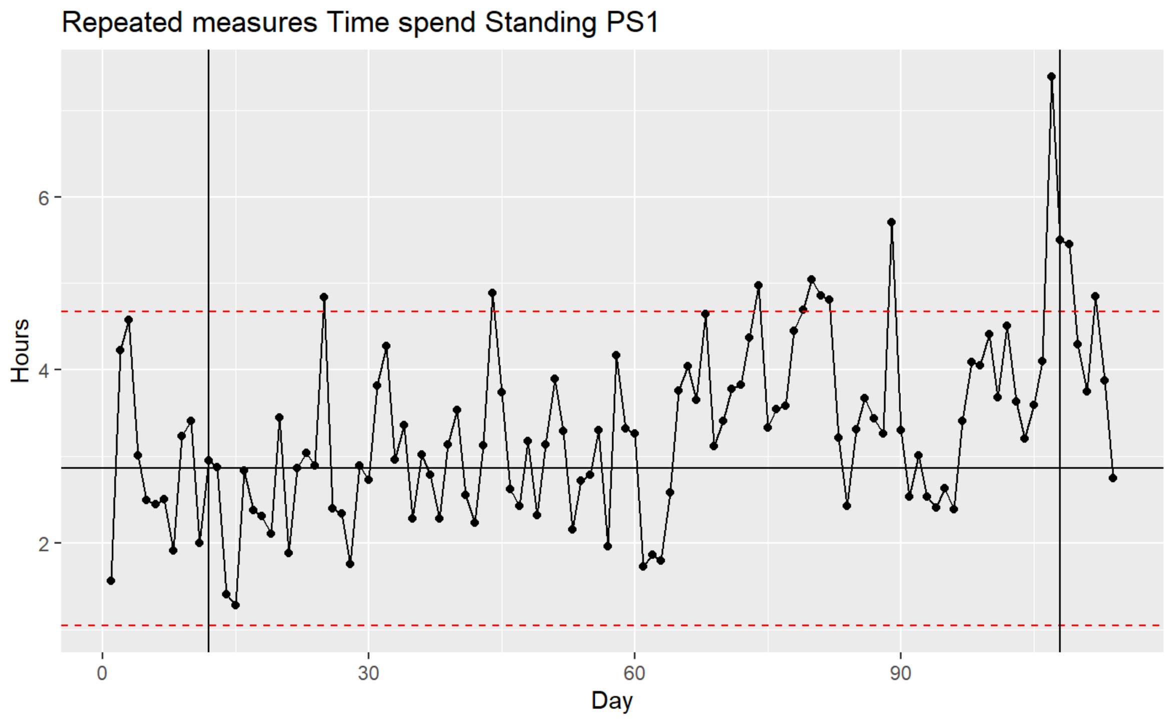

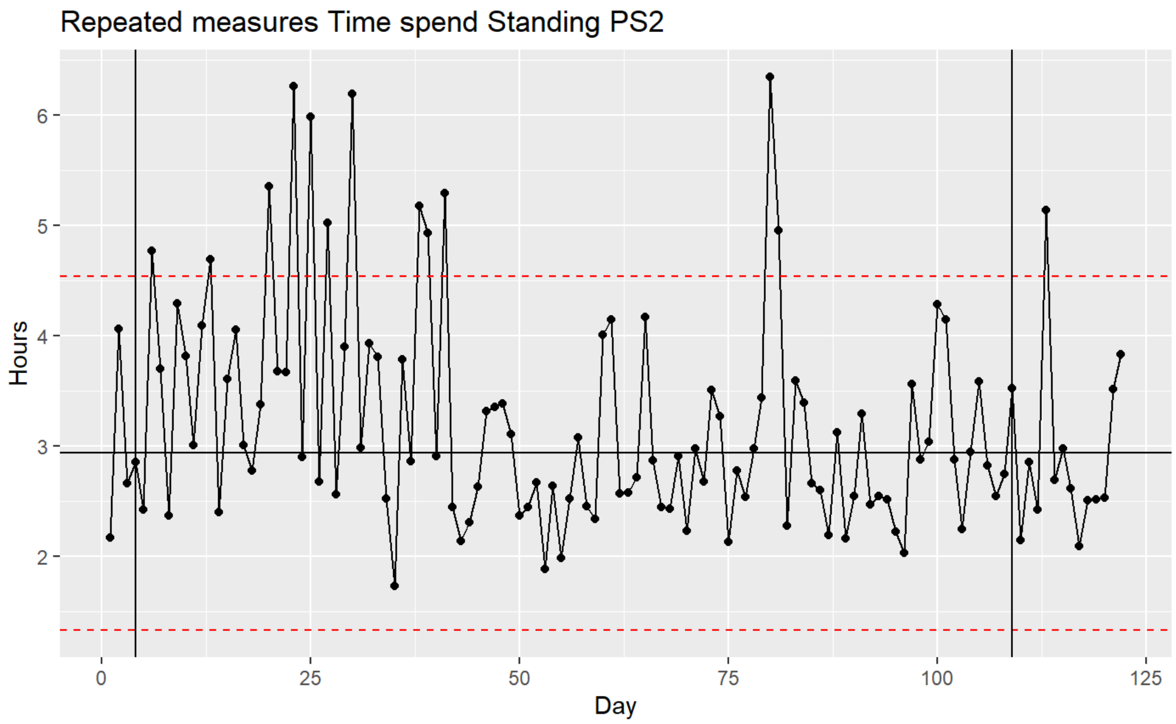

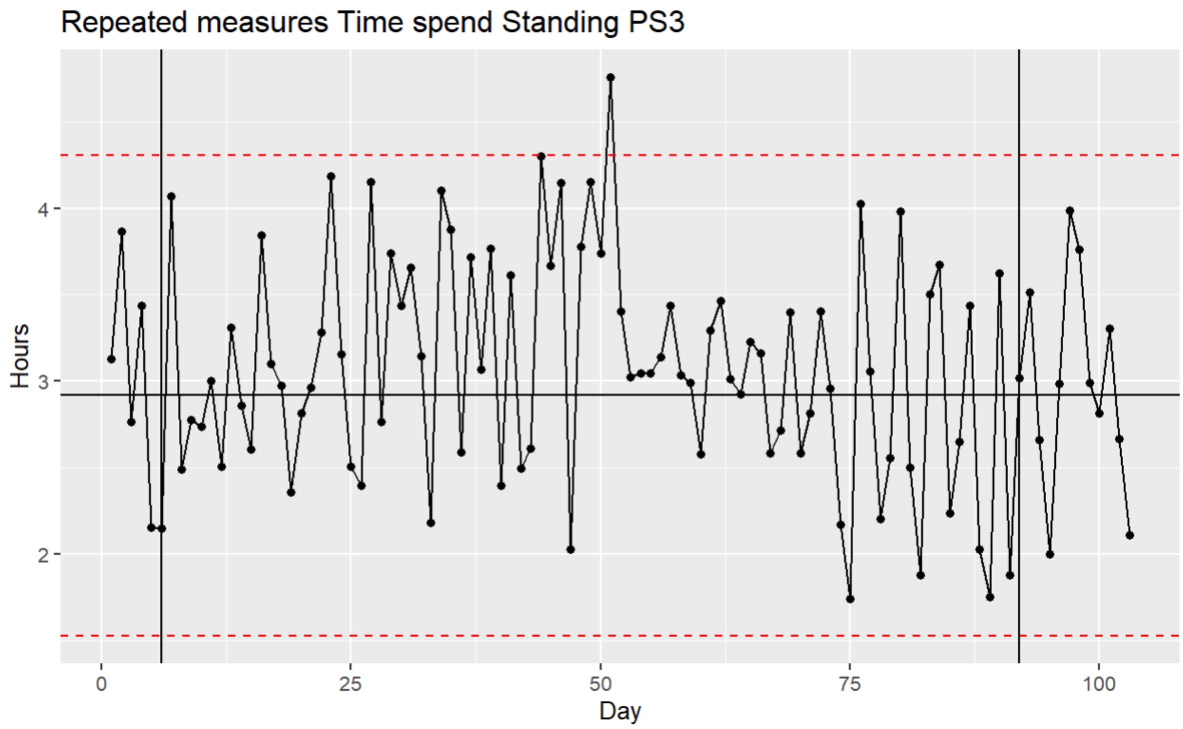
Repeated measures Time spend Standing PS3
Hours
Day
0
25
50
75
100
2
3
4

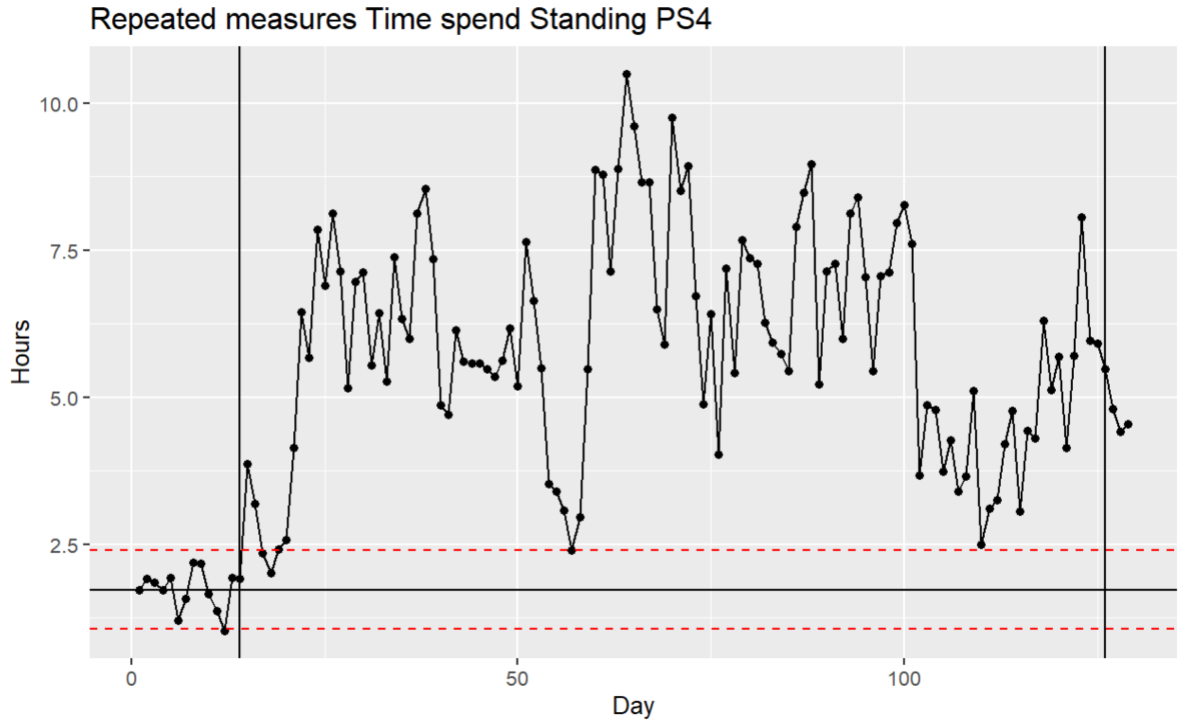
Repeated measures Time spend Standing PS4
Hours
Day
0
50
100
2.5
5.0
7.5
10.0

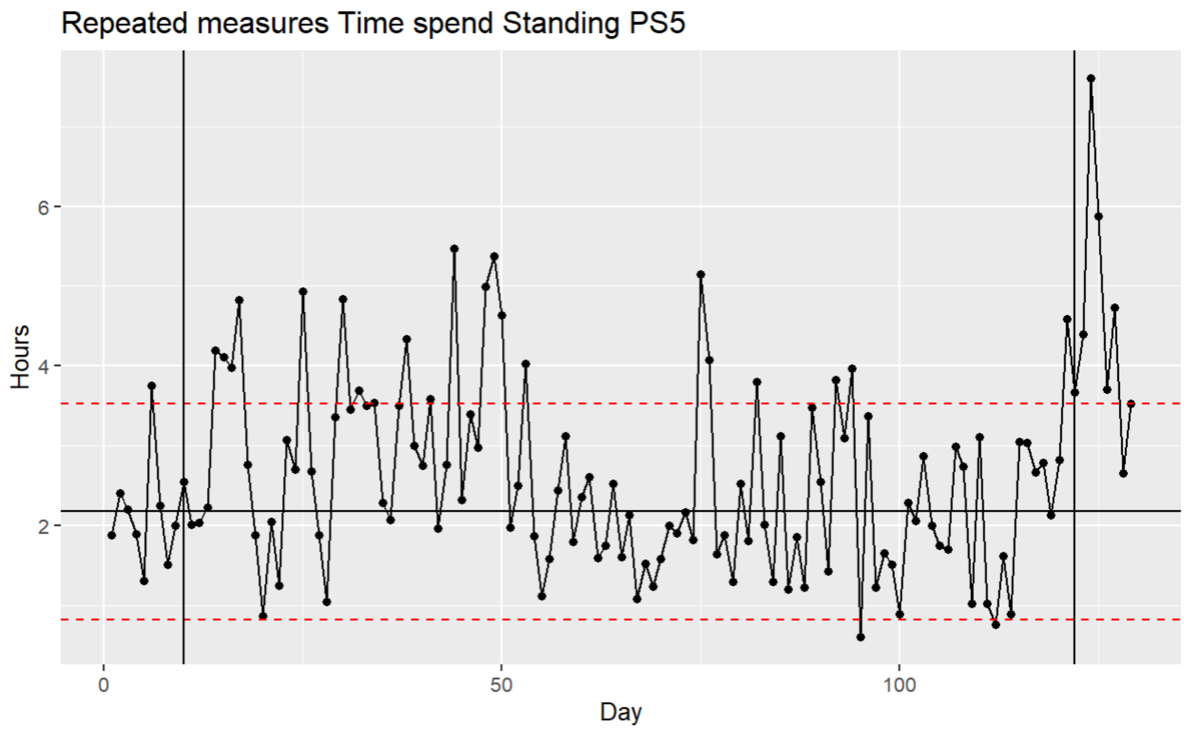
Repeated measures Time spend Standing PS5
Hours
Day
2
4
6
0
50
100

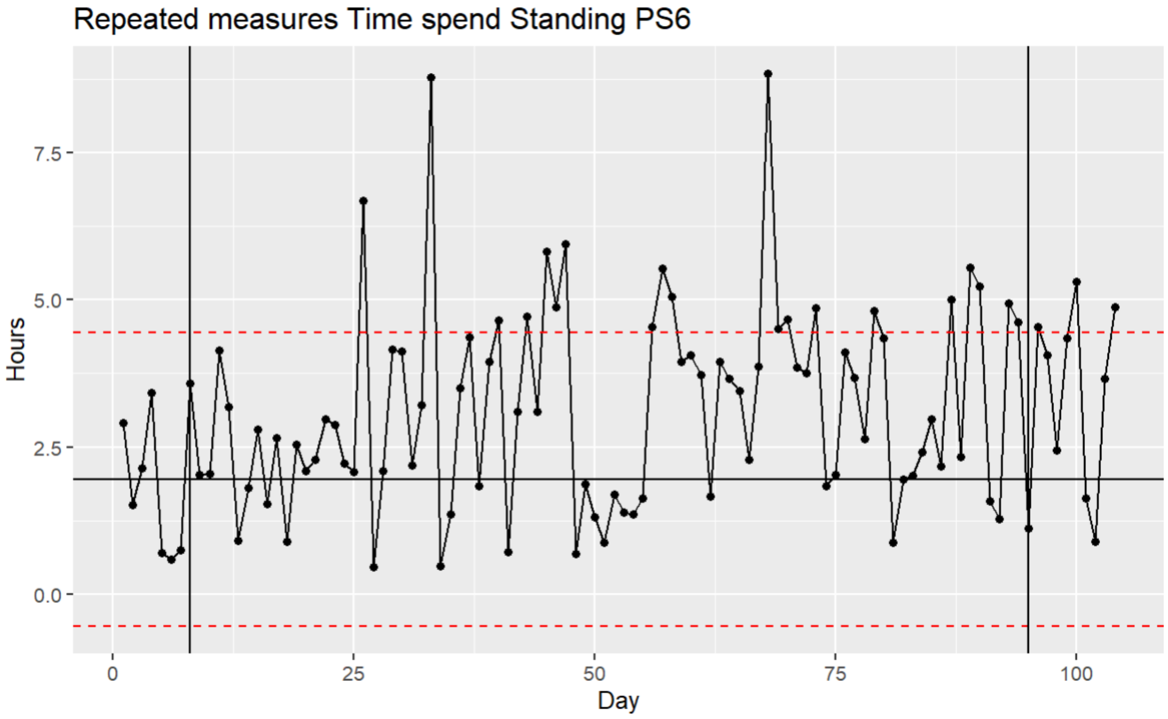
Repeated measures Time spend Standing PS6
Hours
Day
0.0
2.5
5.0
7.5
0
25
50
75
100

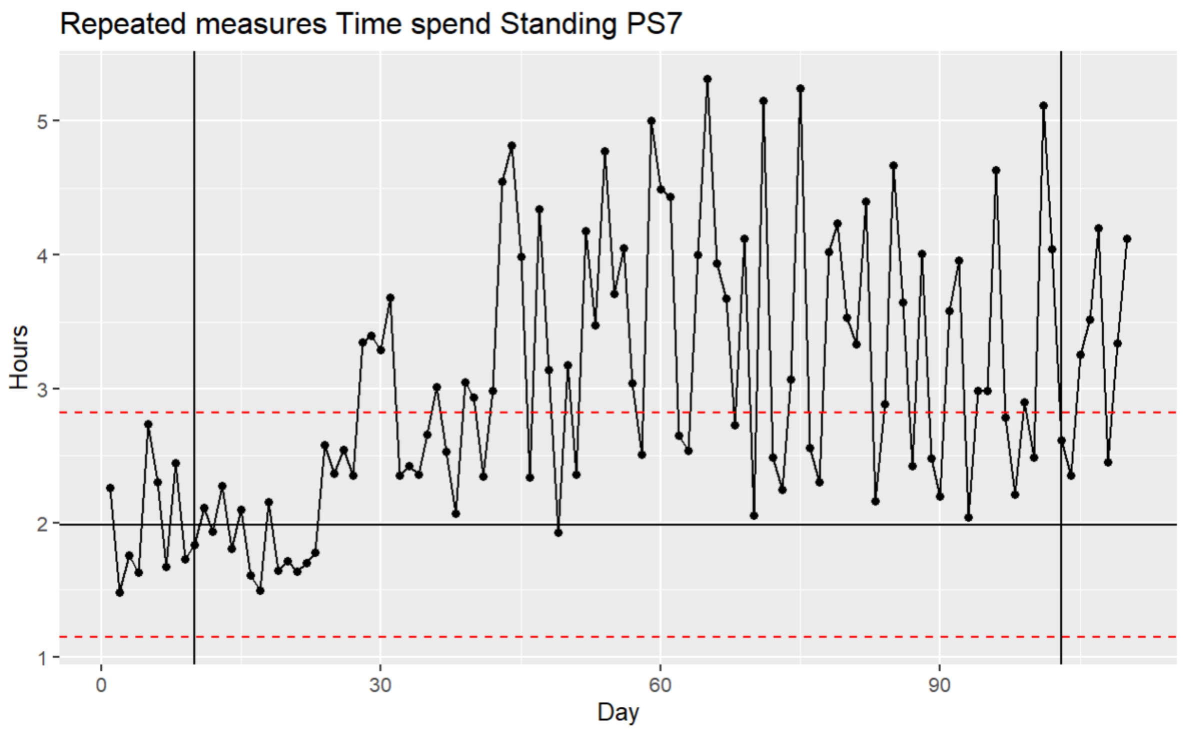
Repeated measures Time spend Standing PS7
Hours
Day
5
4
3
2
1
0
30
60
90

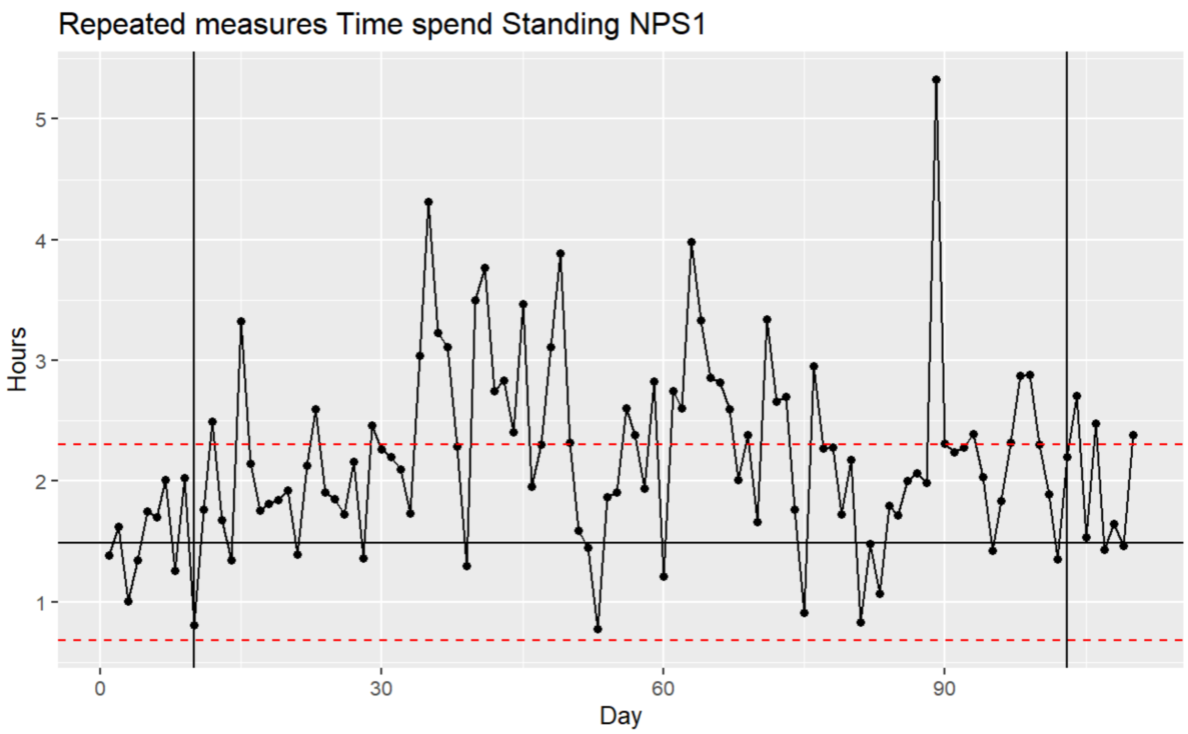
Repeated measures Time spend Standing NPS1
Hours
Day
1
2
3
4
5
0
30
60
90

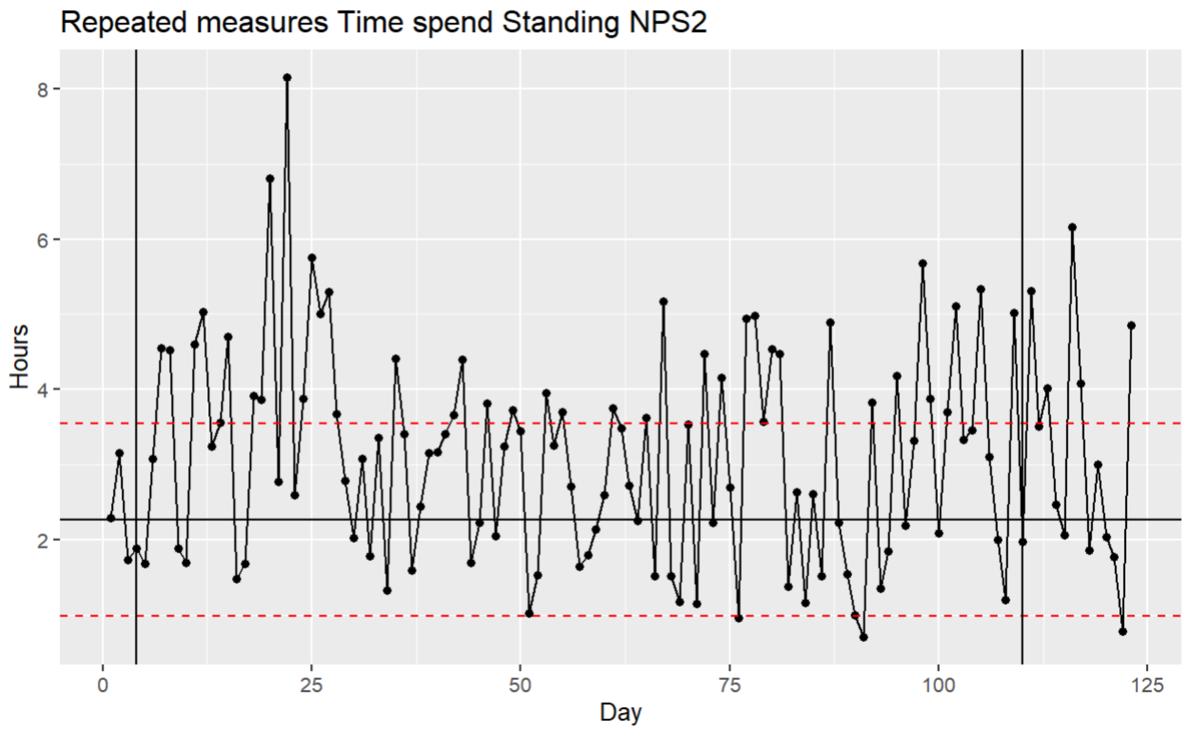
Repeated measures Time spend Standing NPS2
Hours
Day
2
4
6
8
0
25
50
75
100
125

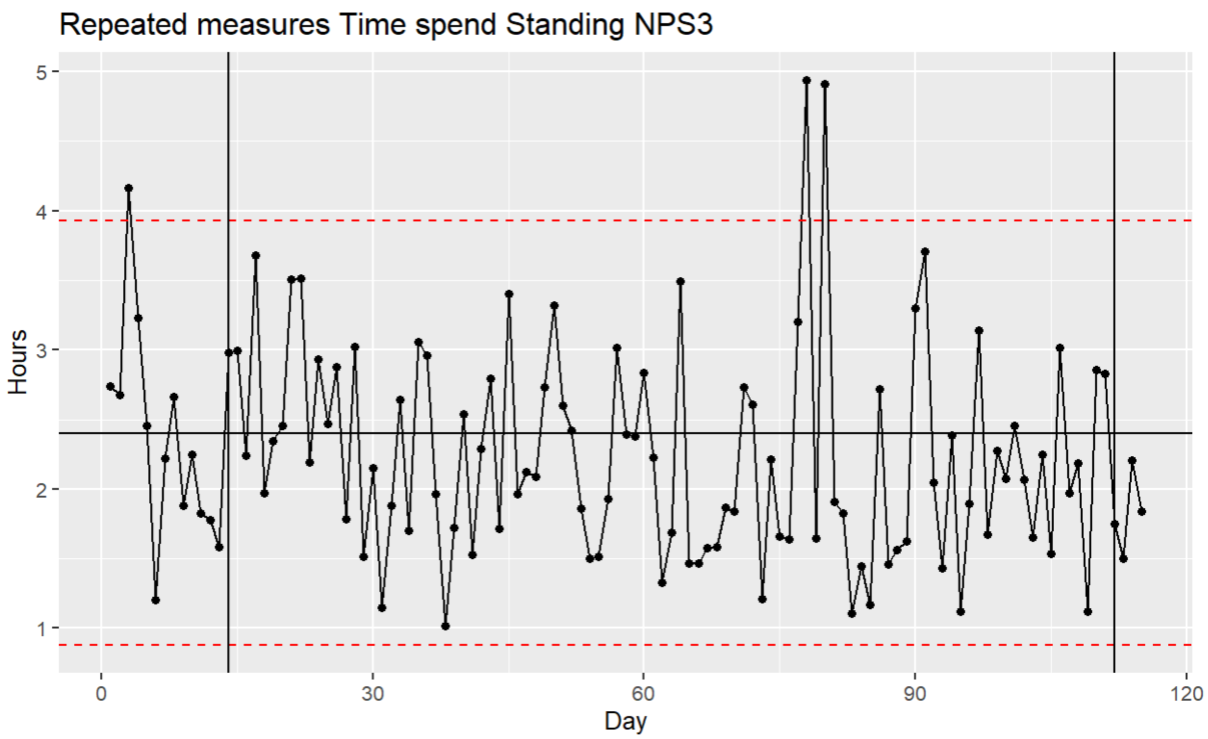

Repeated measures Time spend Standing NPS3
Hours
5
4
3
2
1
0
30
60
90
120
Day

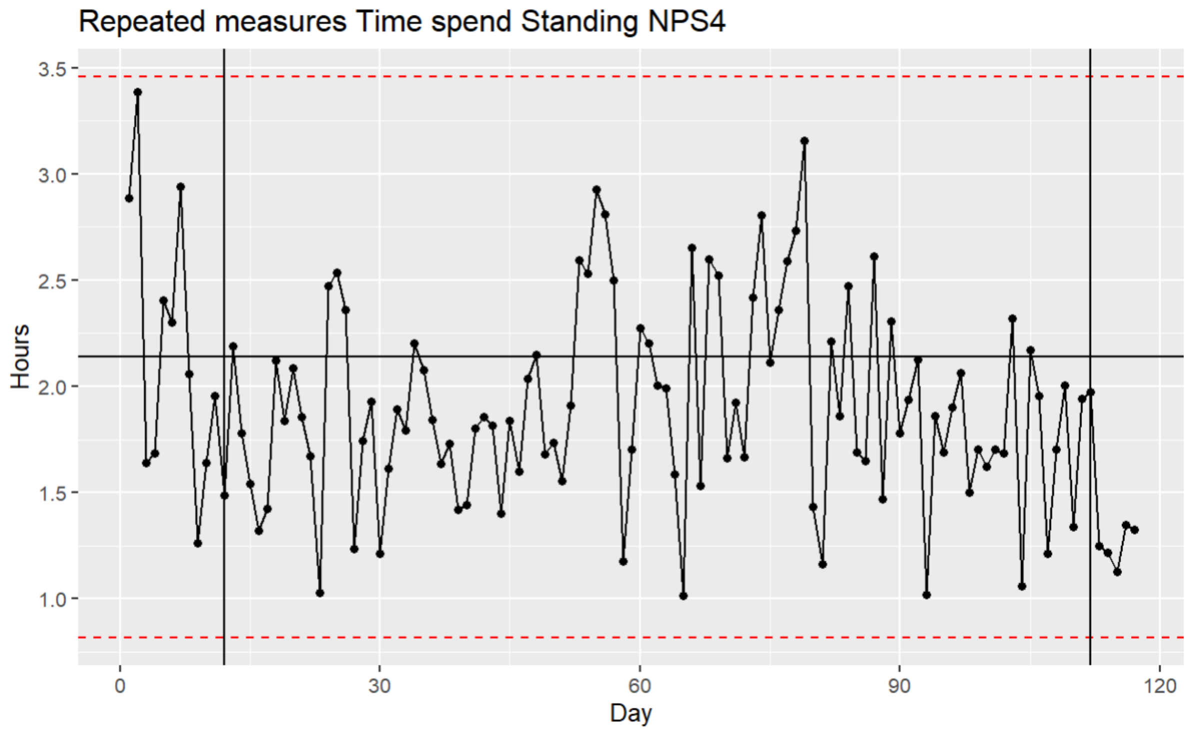

Repeated measures Time spend Standing NPS4
Hours
3.5
3.0
2.5
2.0
1.5
1.0
0
30
60
90
120
Day

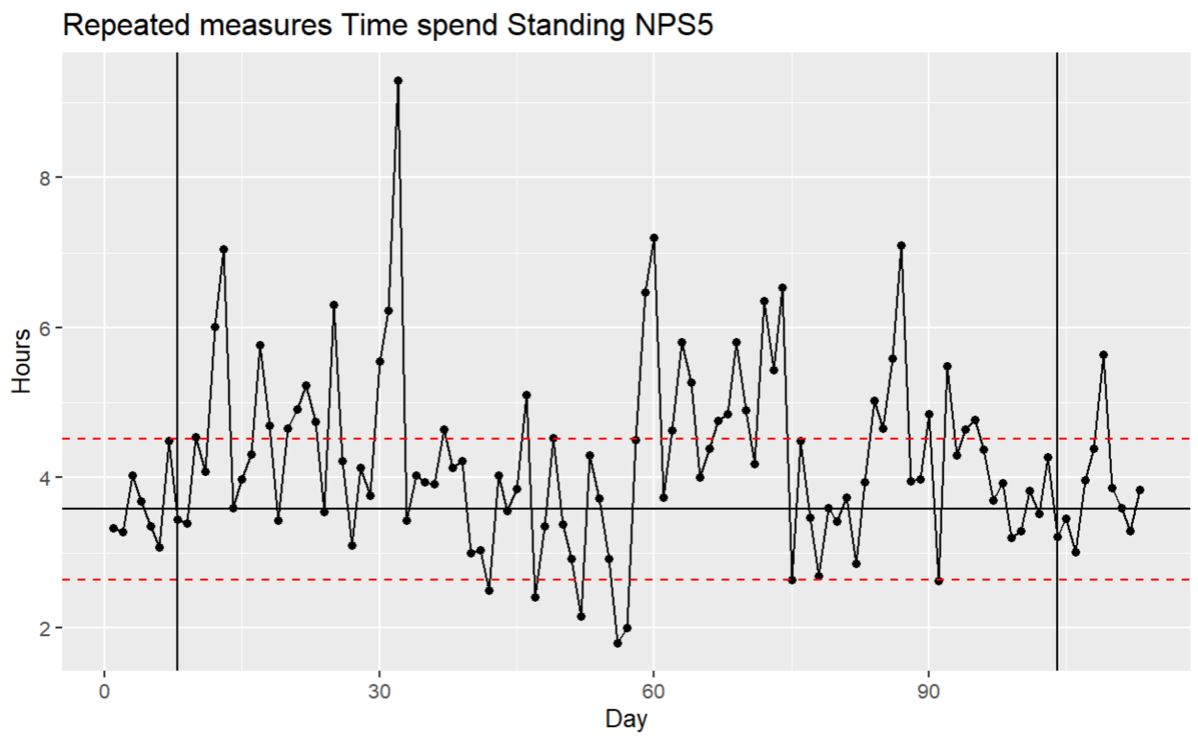
Repeated measures Time spend Standing NPS5
Hours
8
6
4
2
0
30
60
90
Day

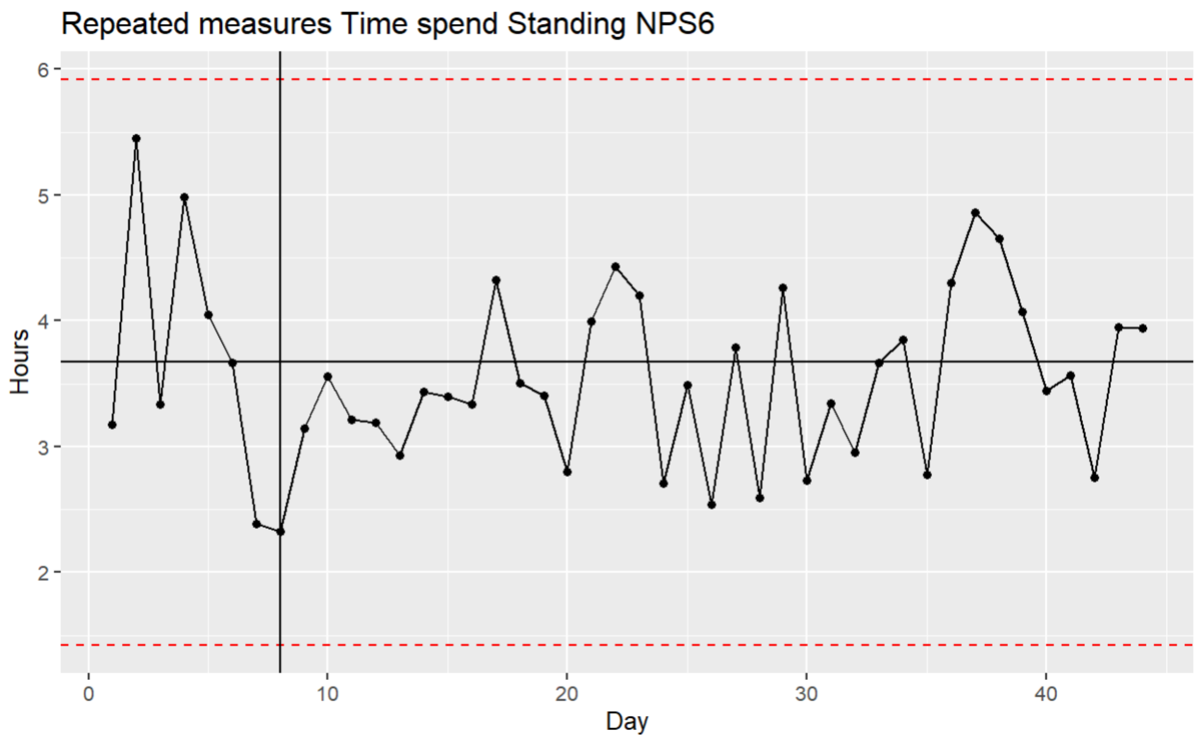
Repeated measures Time spend Standing NPS6
Hours
6
5
4
3
2
0
10
20
30
40
Day

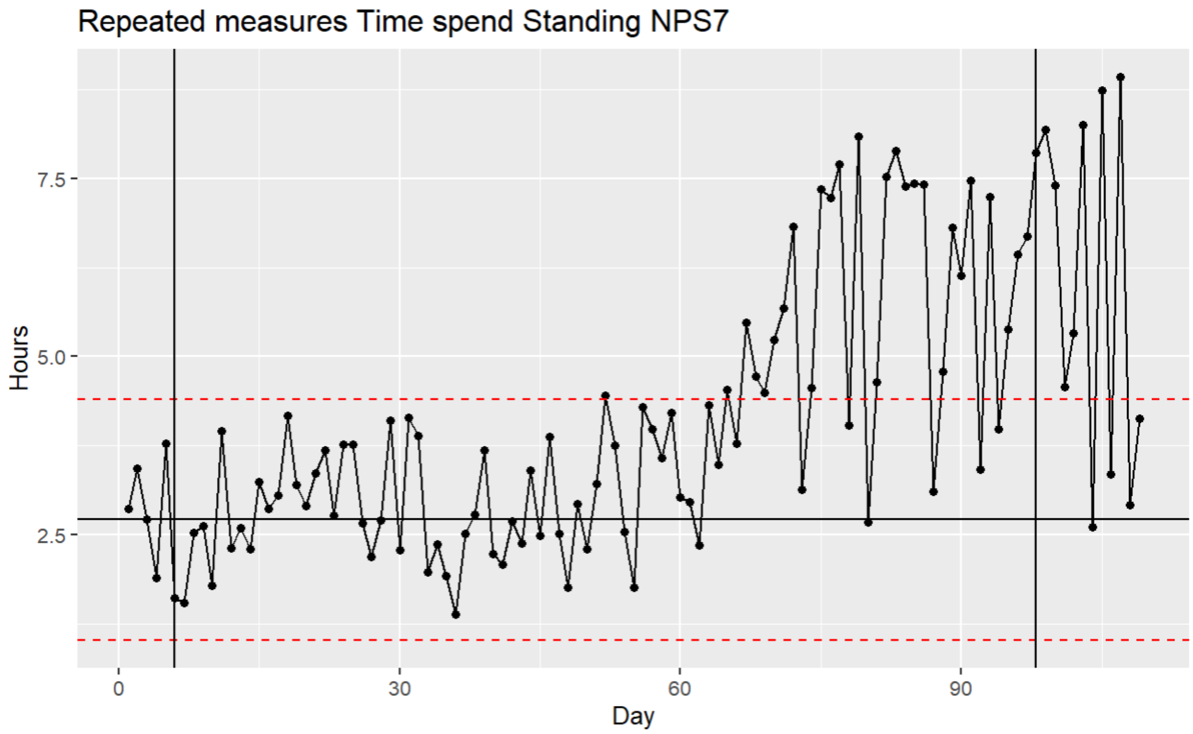

Repeated measures Time spend Standing NPS7
Hours
7.5
5.0
2.5
0
30
60
90
Day

Time spend walking

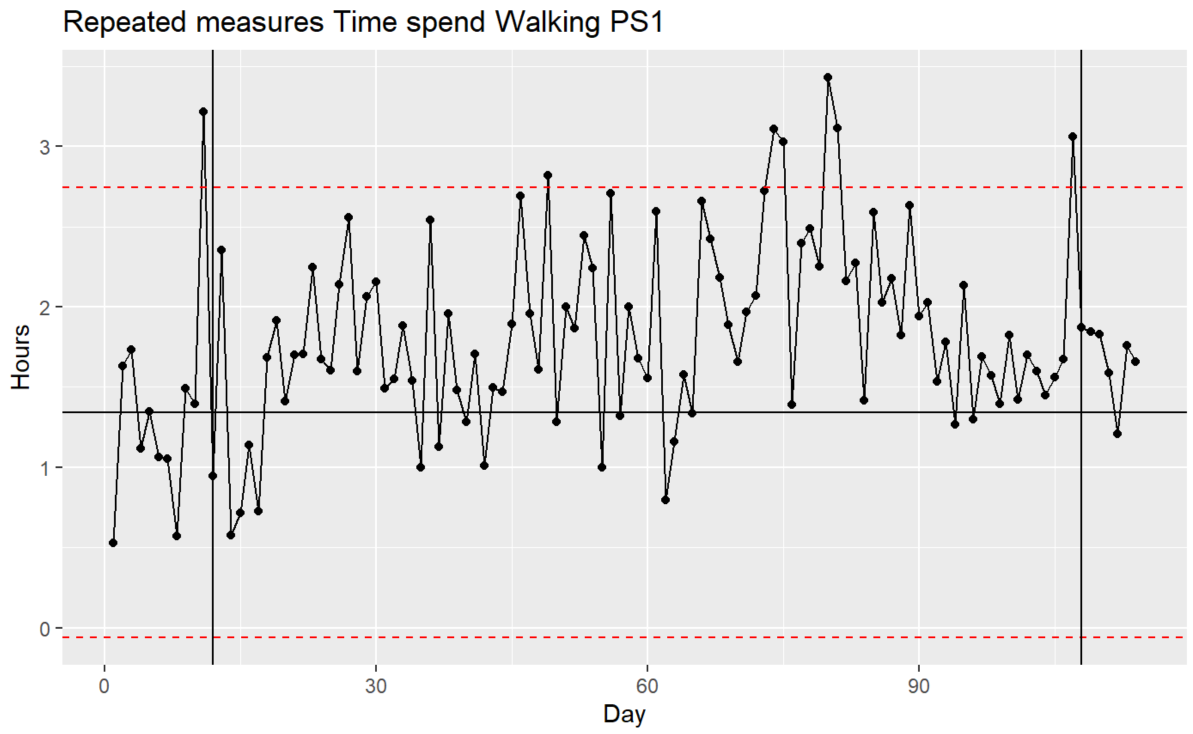

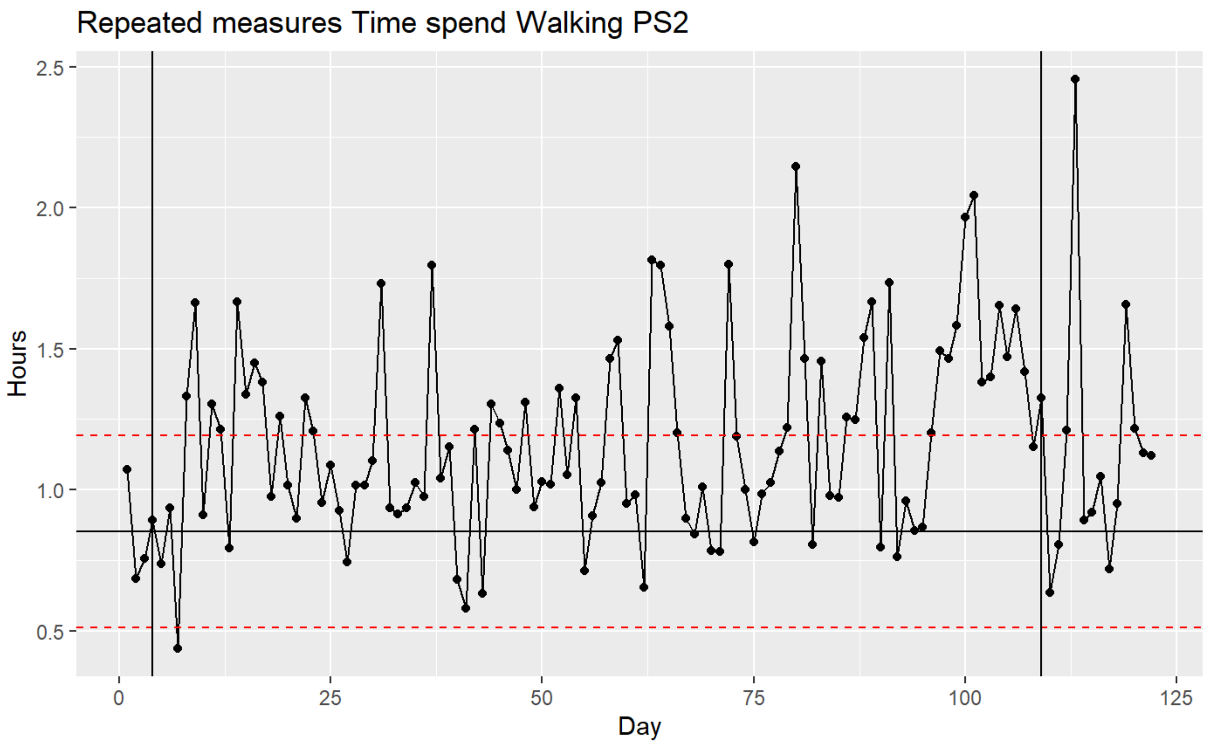

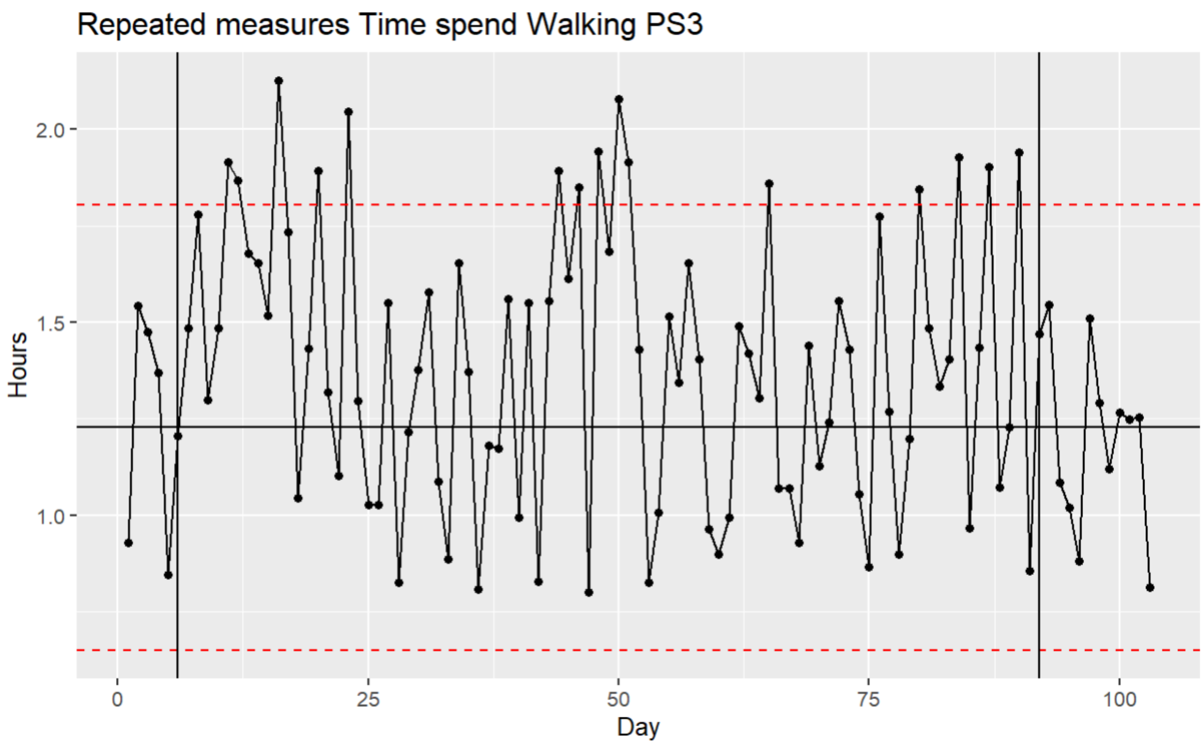
Repeated measures Time spend Walking PS3
Hours
2.0
1.5
1.0
0
25
50
75
100
Day

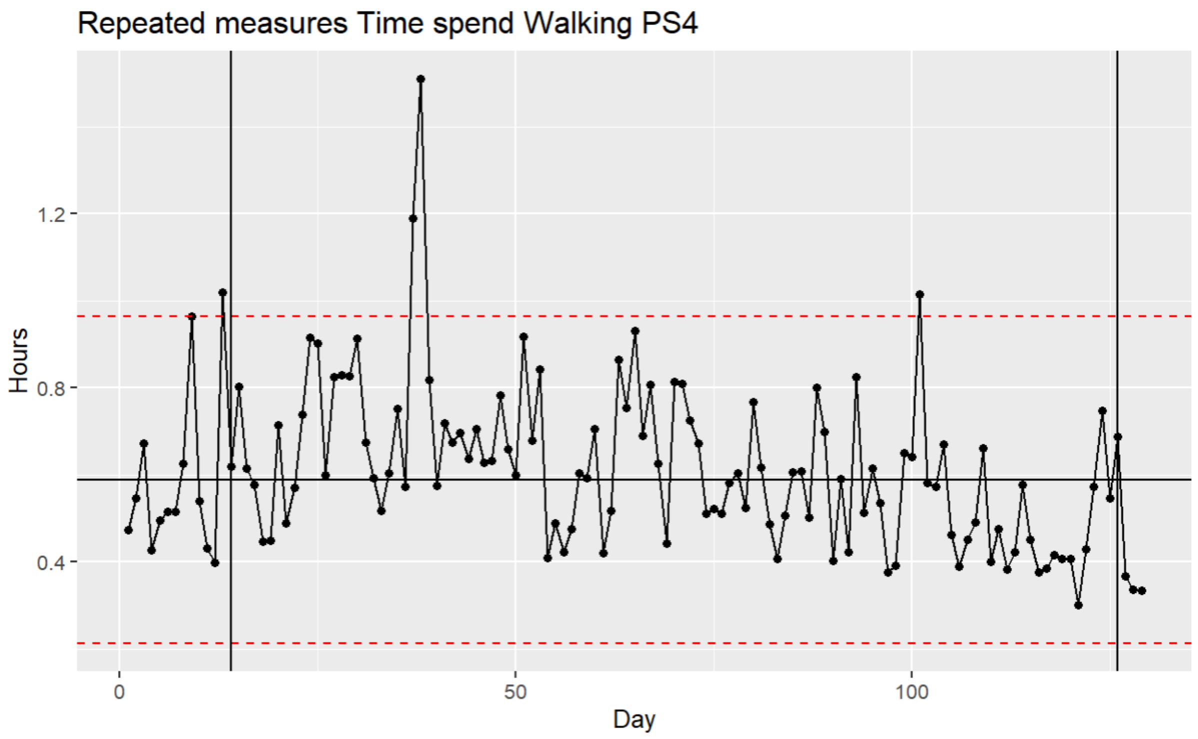
Repeated measures Time spend Walking PS4
Hours
1.2
0.8
0.4
0
50
100
Day

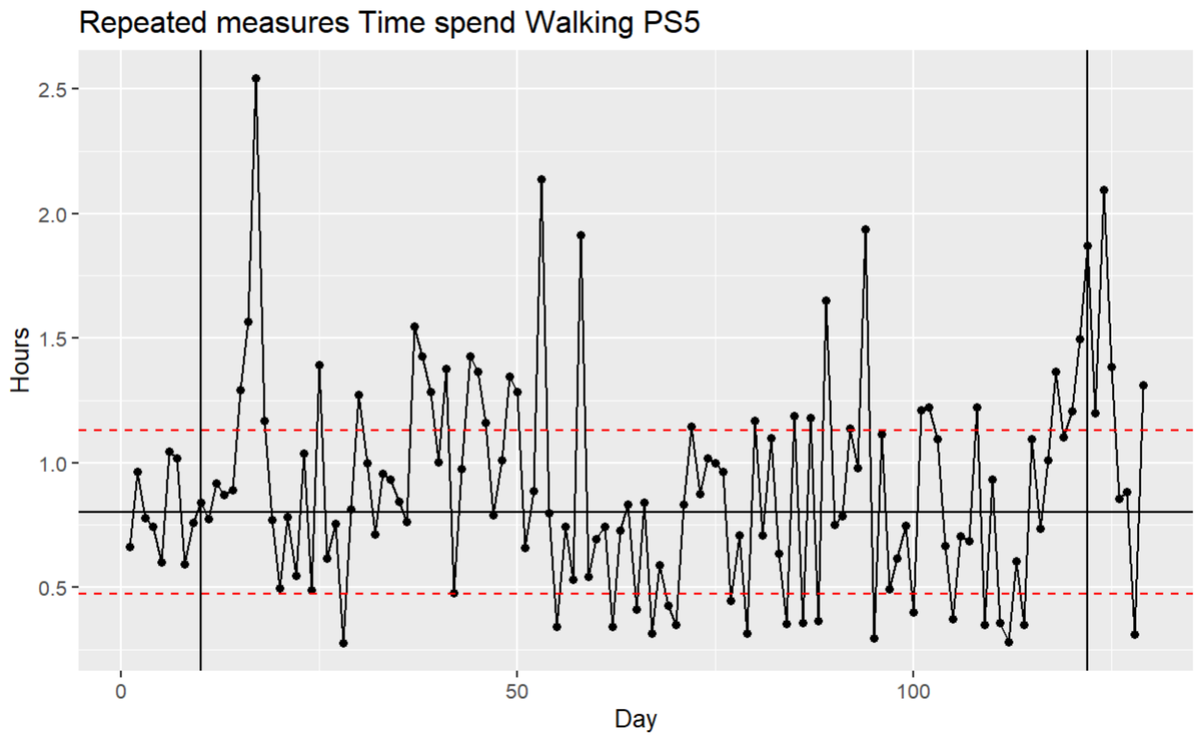

Repeated measures Time spend Walking PS5
Hours
Day
2.5
2.0
1.5
1.0
0.5
0
50
100

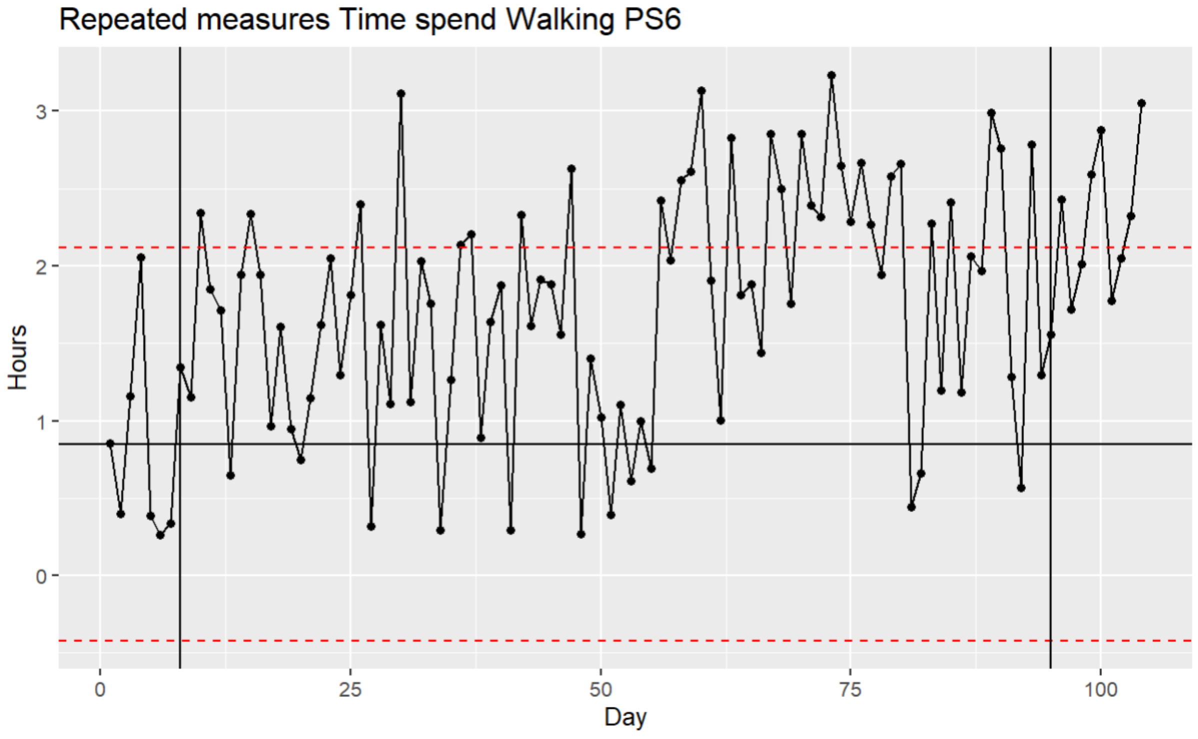

Repeated measures Time spend Walking PS6
Hours
Day
3
2
1
0
0
25
50
75
100

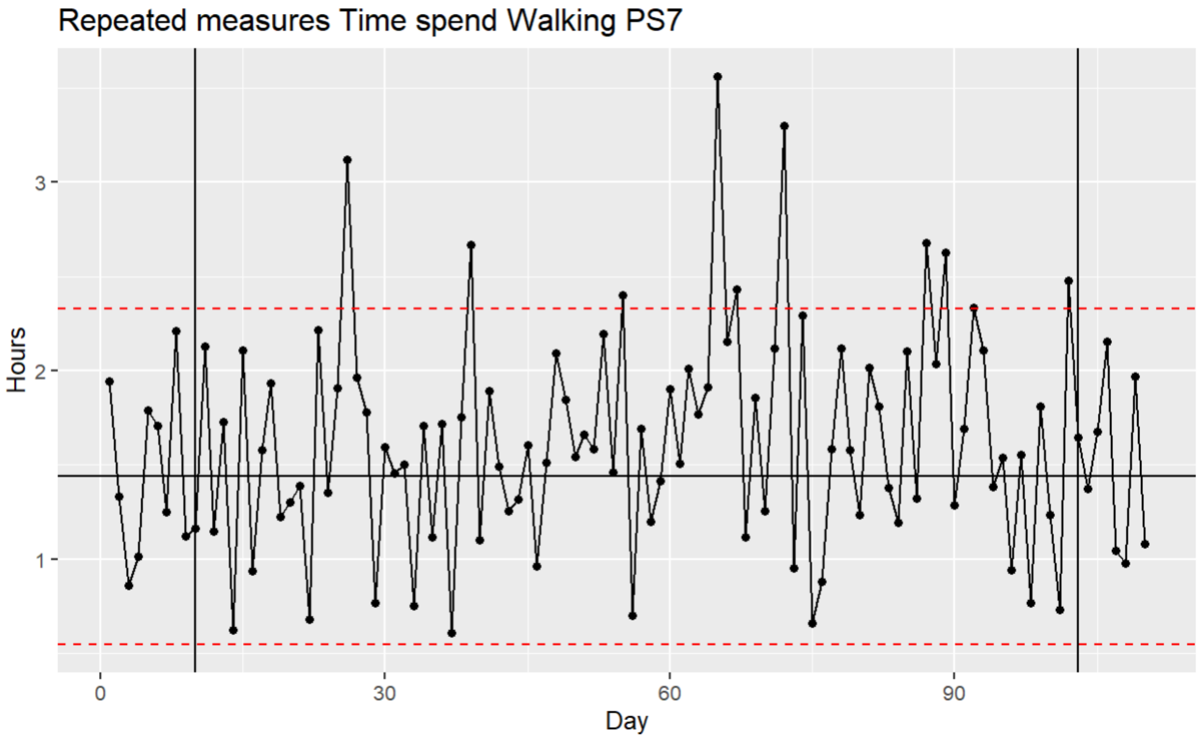

Repeated measures Time spend Walking PS7
Hours
Day
0
30
60
90
1
2
3

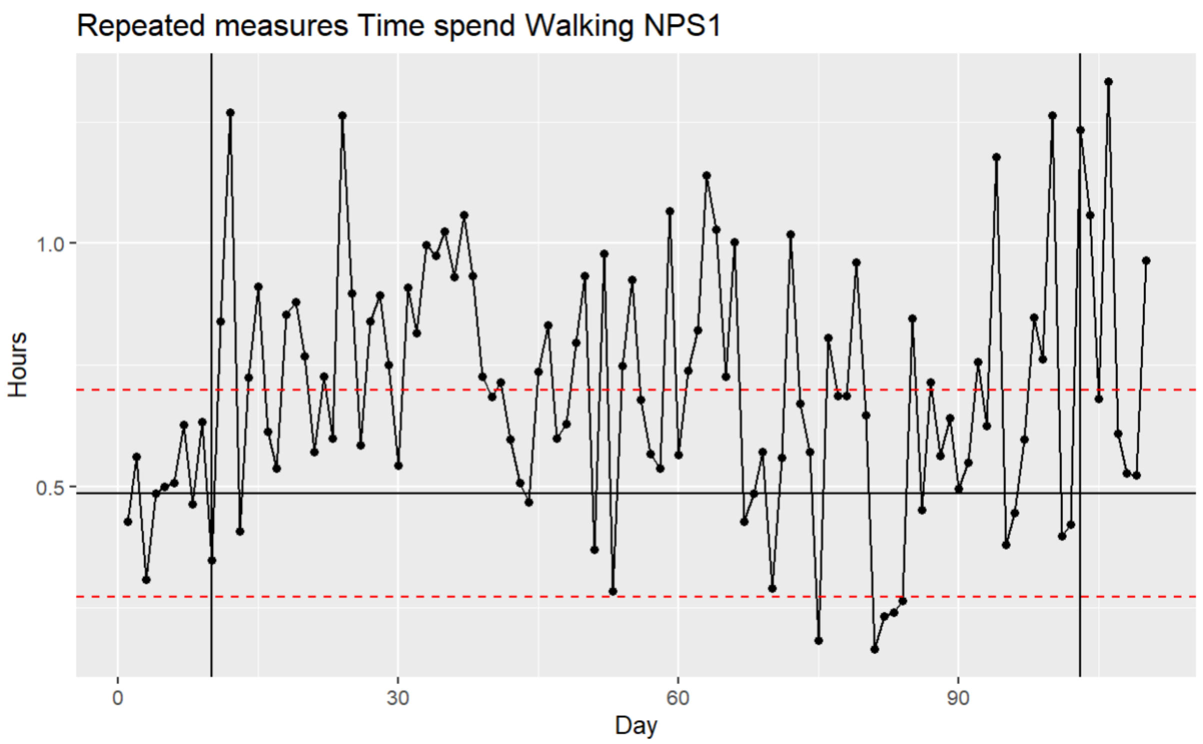
Repeated measures Time spend Walking NPS1
Hours
1.0
0.5
0
30
60
90
Day

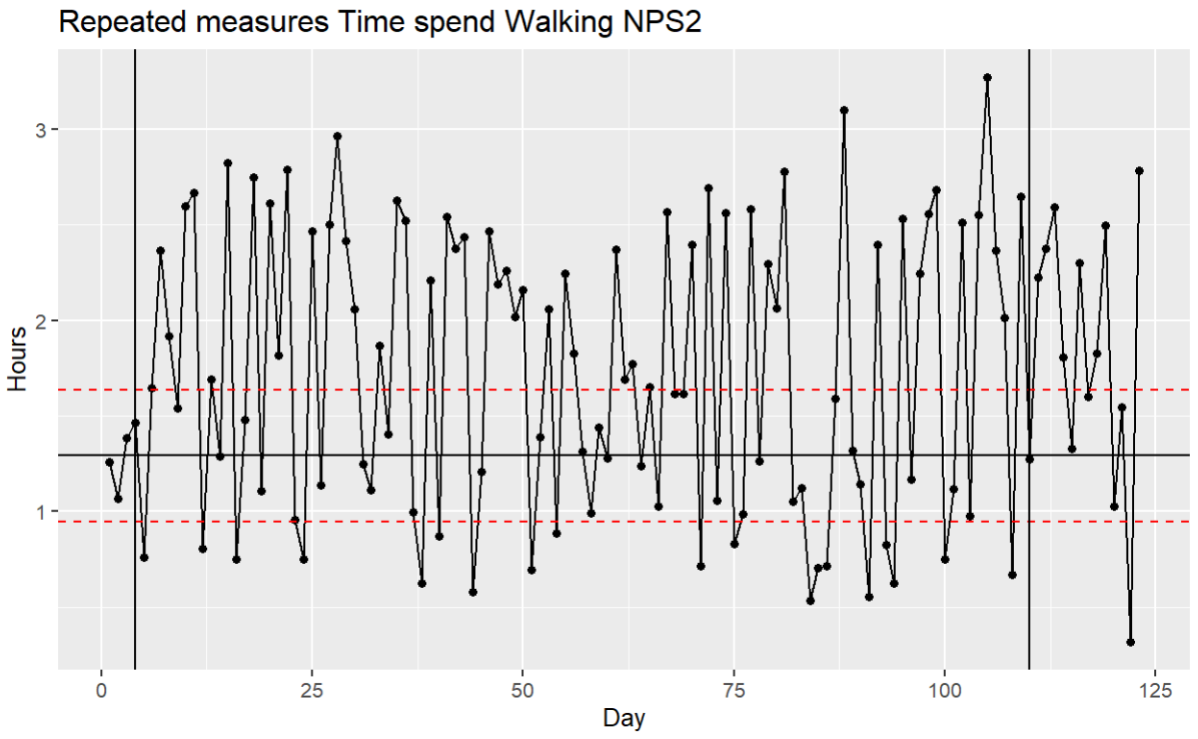
Repeated measures Time spend Walking NPS2
Hours
3
2
1
0
25
50
75
100
125
Day

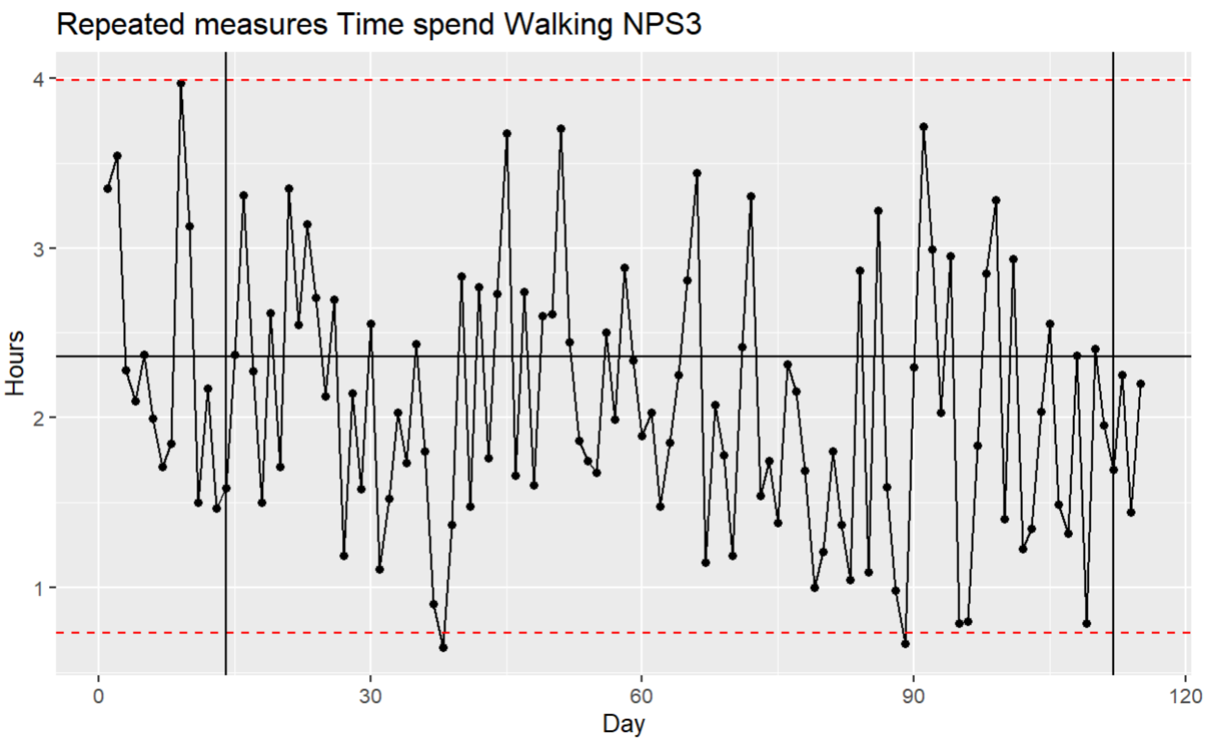
Repeated measures Time spend Walking NPS3
Hours
Day
0
30
60
90
120
1
2
3
4

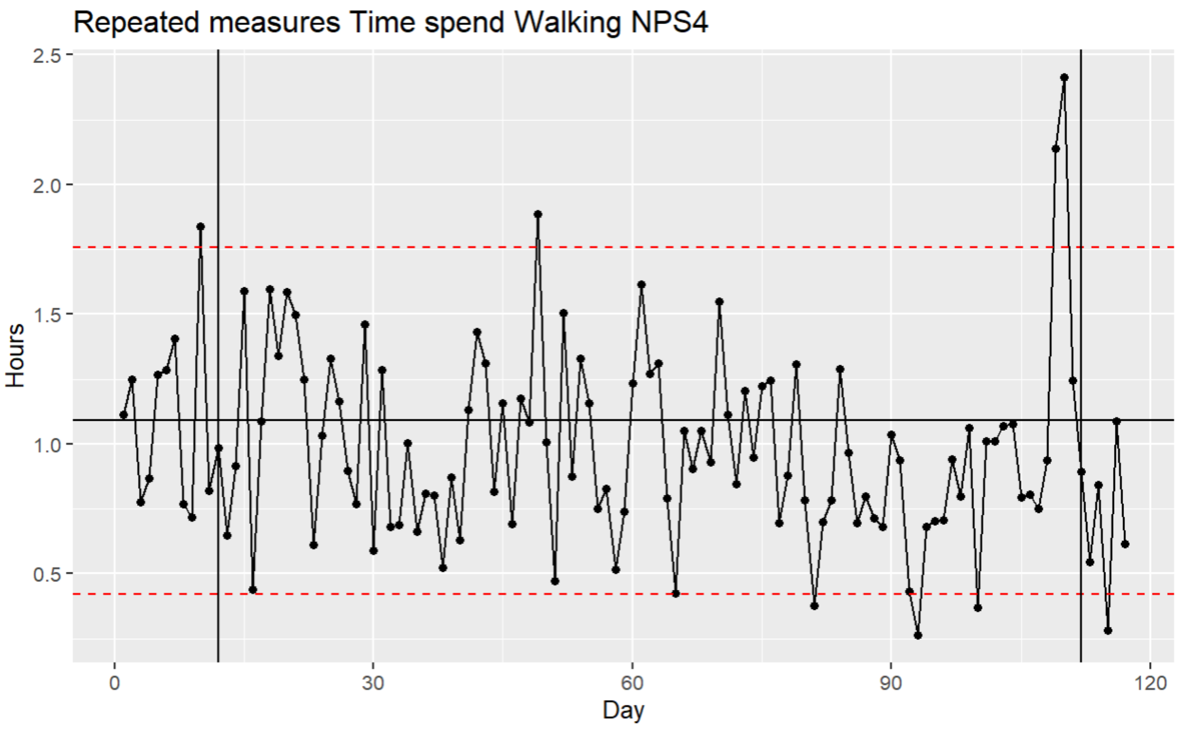
Repeated measures Time spend Walking NPS4
Hours
Day
0
30
60
90
120
0.5
1.0
1.5
2.0
2.5

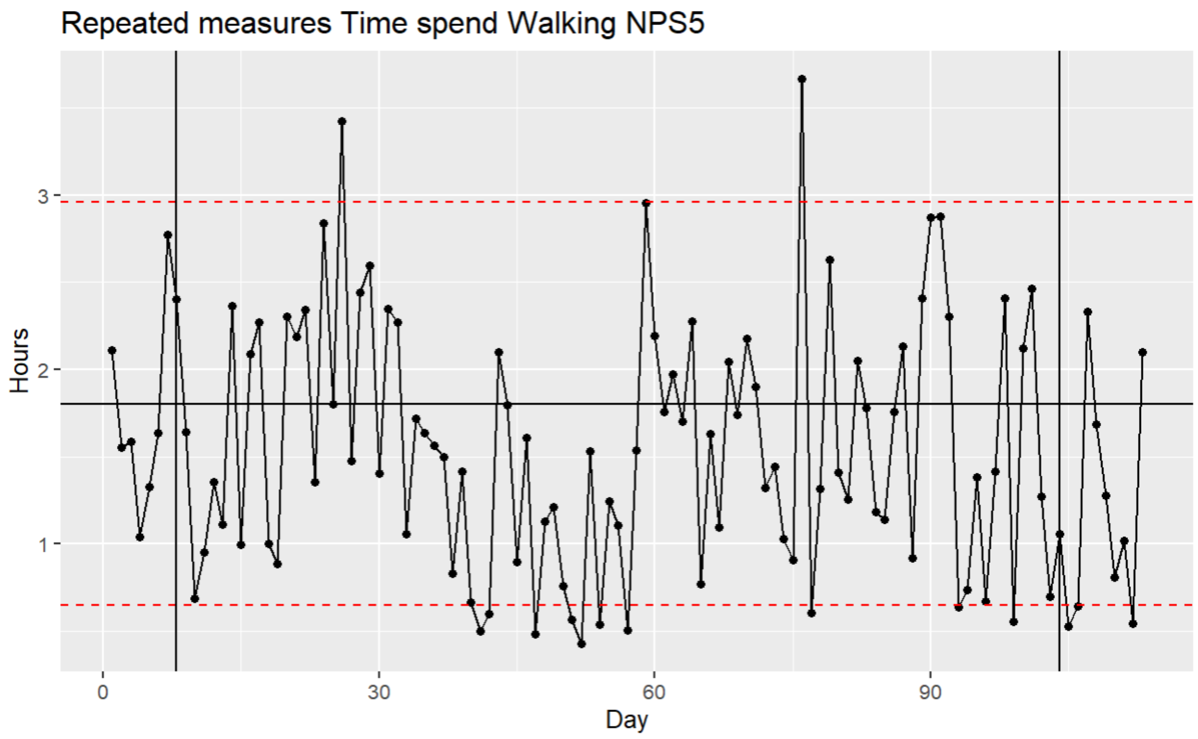

Repeated measures Time spend Walking NPS5
Hours
3
2
1
0
30
60
90
Day

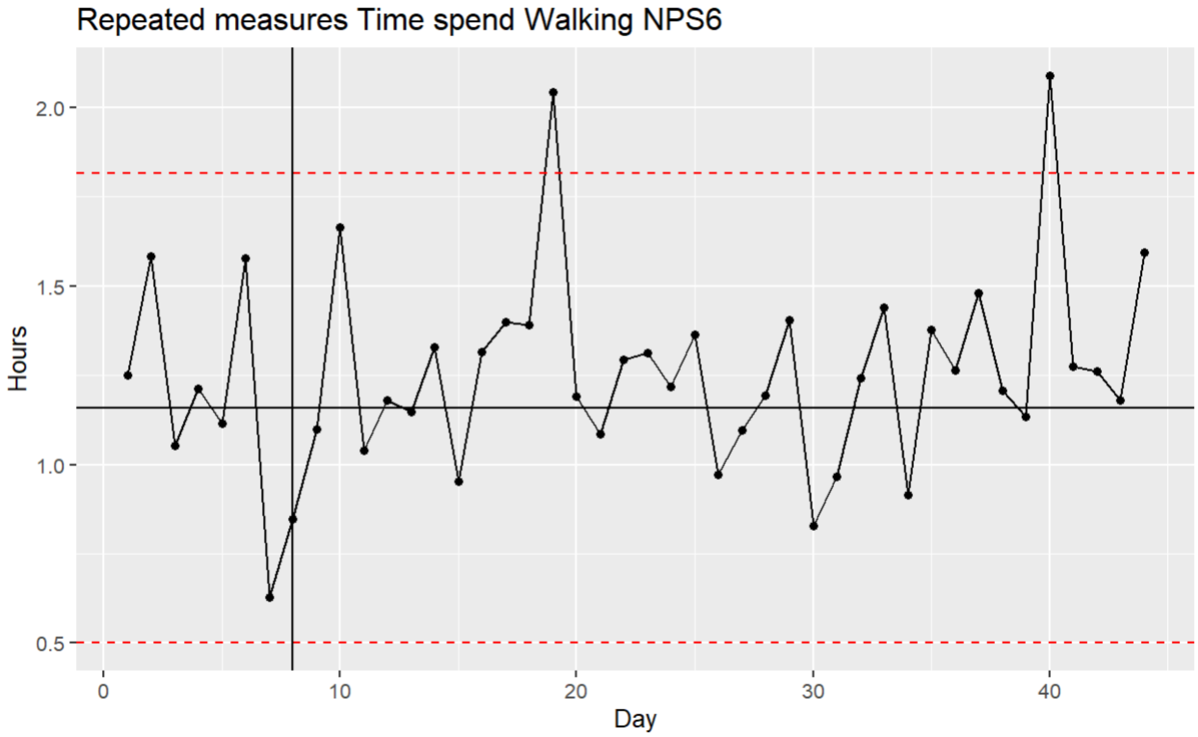

Repeated measures Time spend Walking NPS6
Hours
2.0
1.5
1.0
0.5
0
10
20
30
40
Day

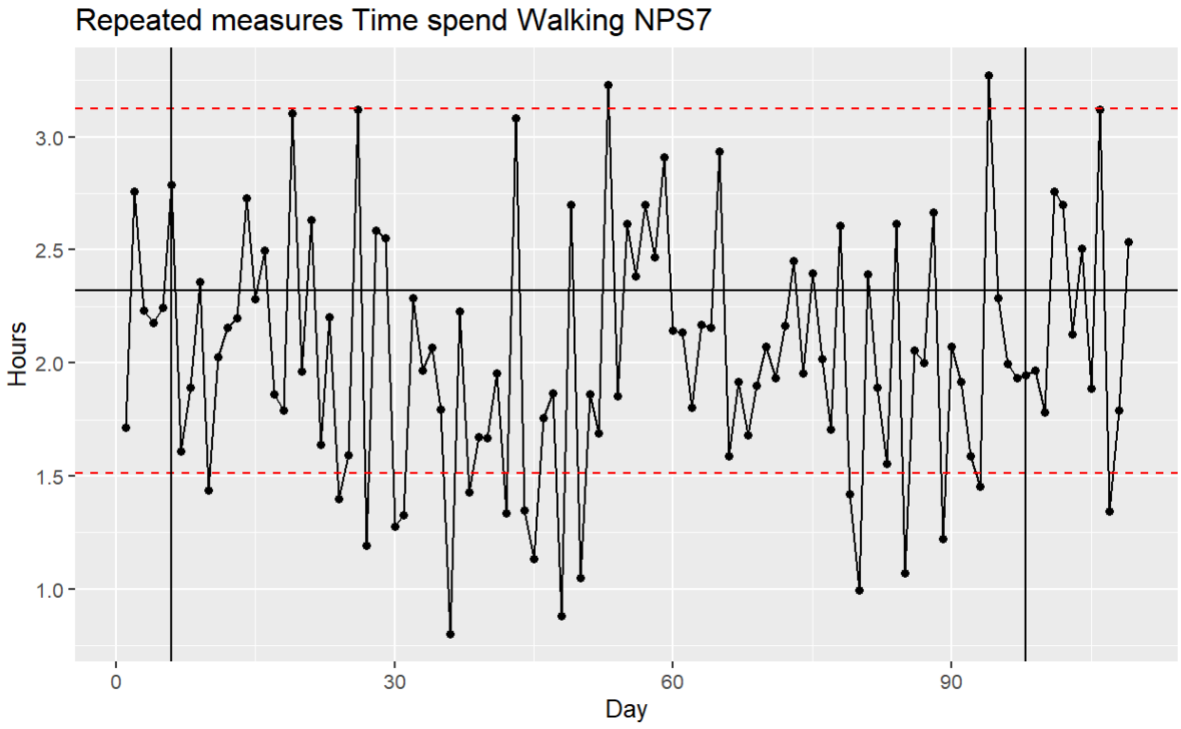

Repeated measures Time spend Walking NPS7
Hours
3.0
2.5
2.0
1.5
1.0
0
30
60
90
Day

Step count

Repeated measures Step count PS1

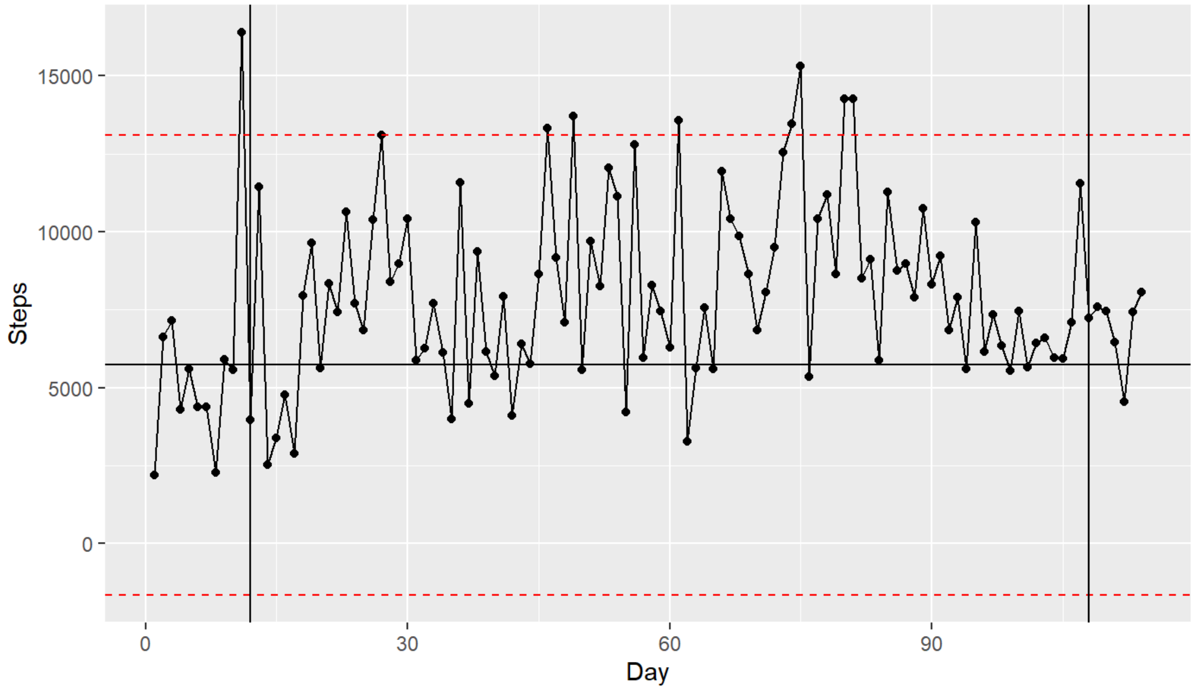

Repeated measures Step count PS2

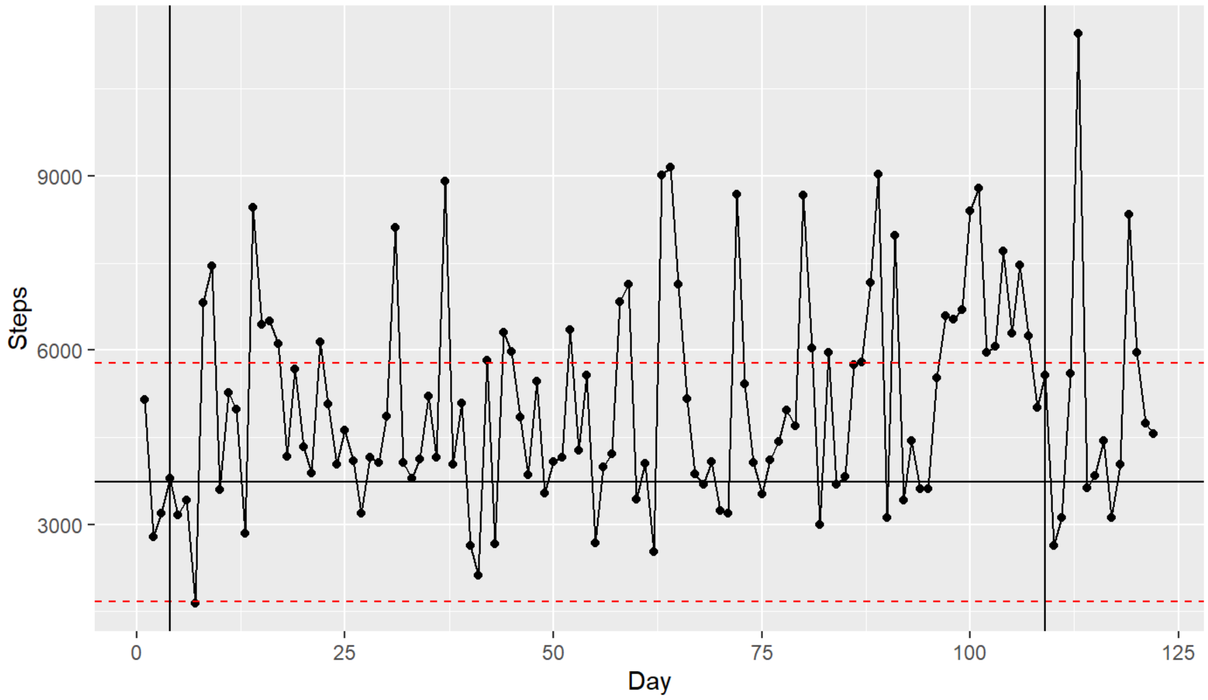

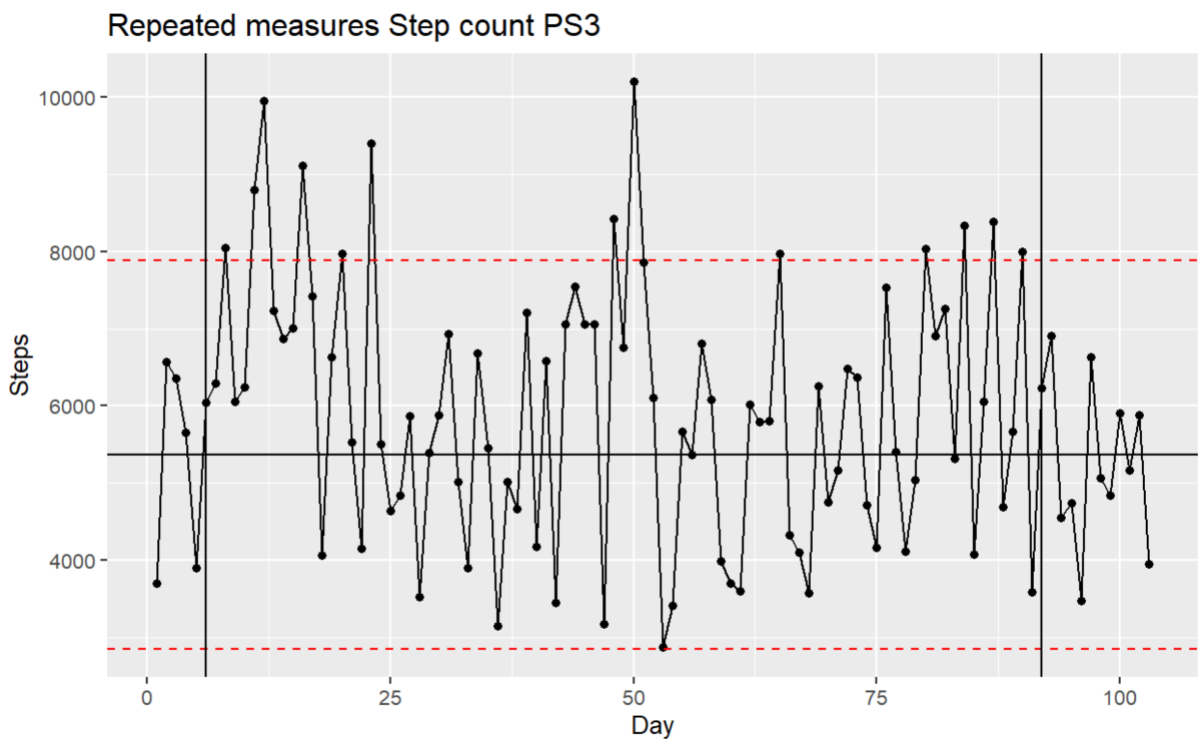

Repeated measures Step count PS3
Steps
10000
8000
6000
4000
0
25
50
75
100
Day

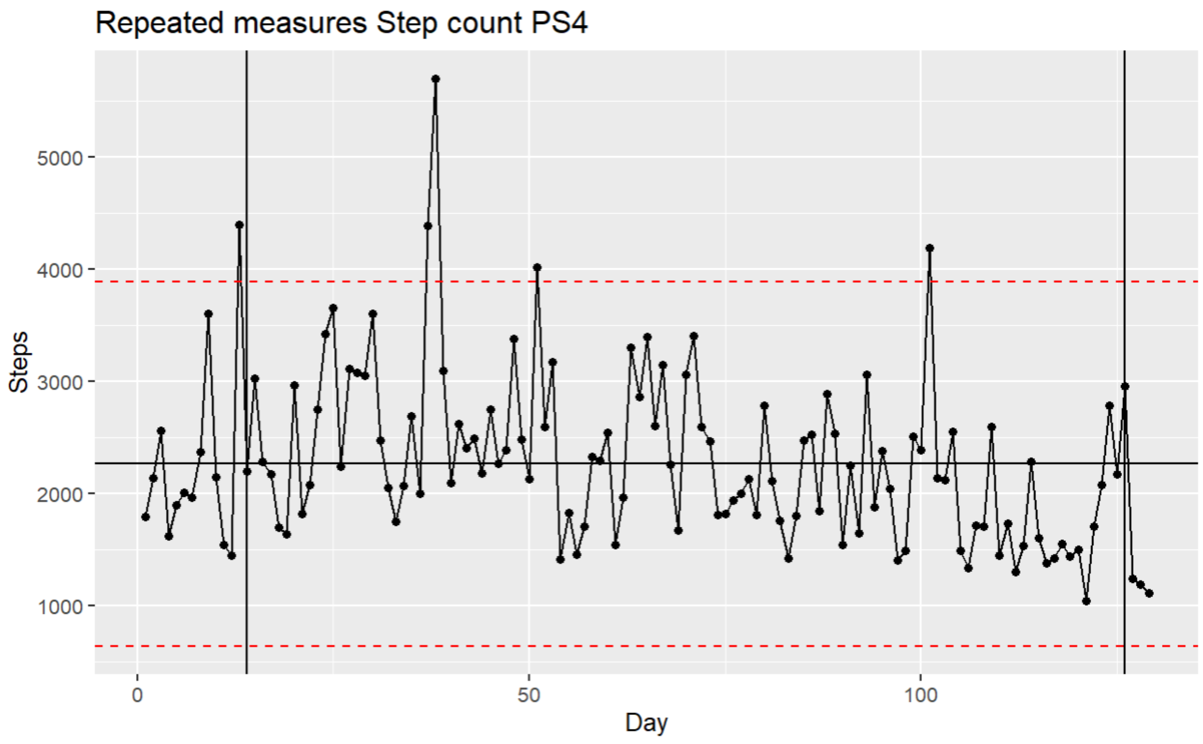

Repeated measures Step count PS4
Steps
5000
4000
3000
2000
1000
0
50
100
Day

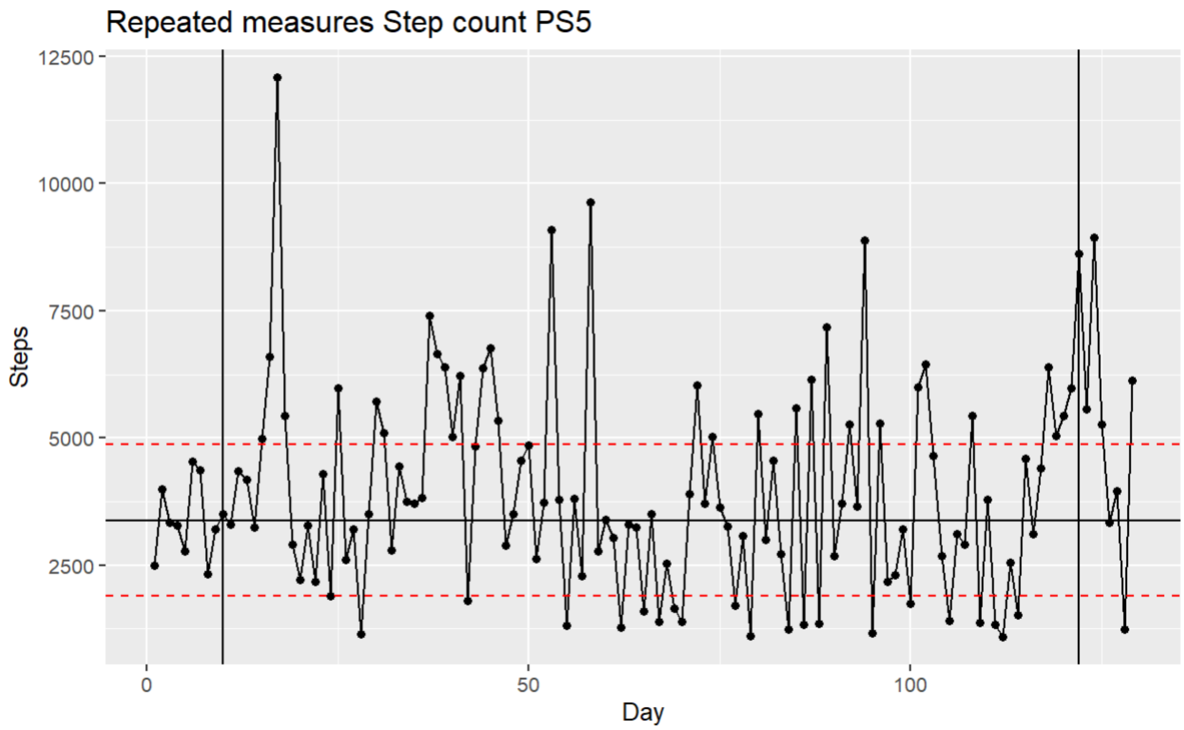
Repeated measures Step count PS5
Steps
12500
10000
7500
5000
2500
0
50
100
Day

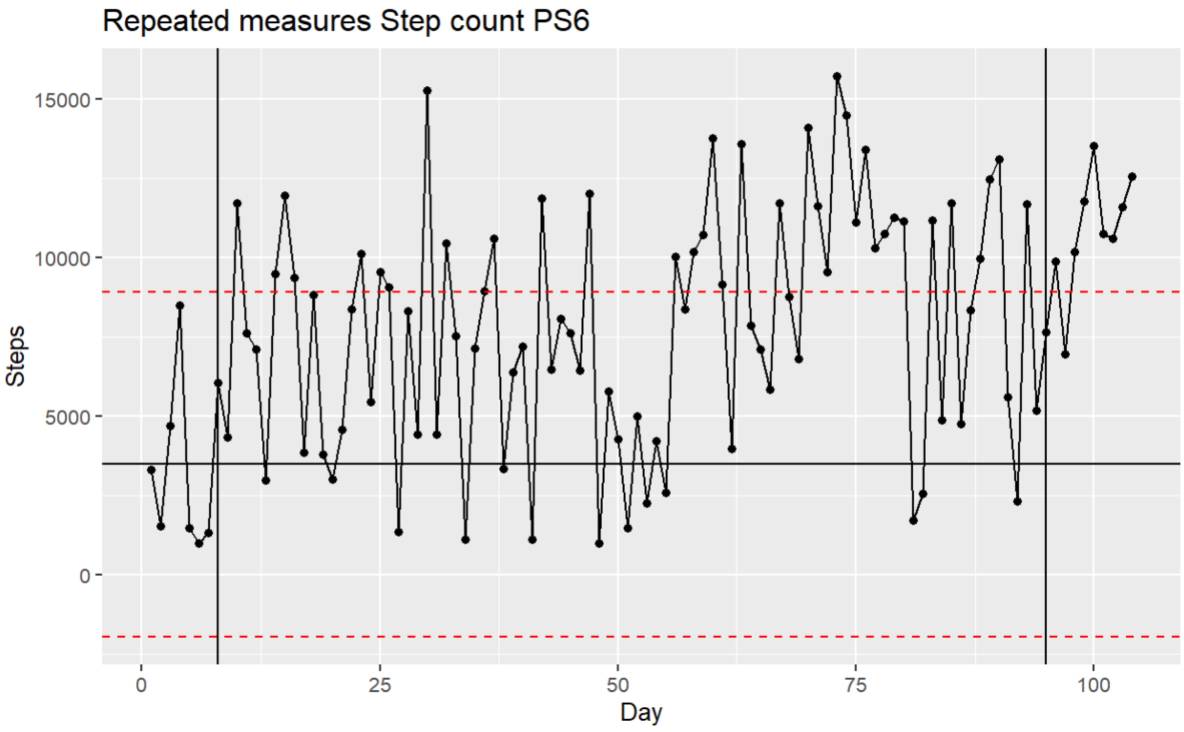
Repeated measures Step count PS6
Steps
15000
10000
5000
0
0
25
50
75
100
Day

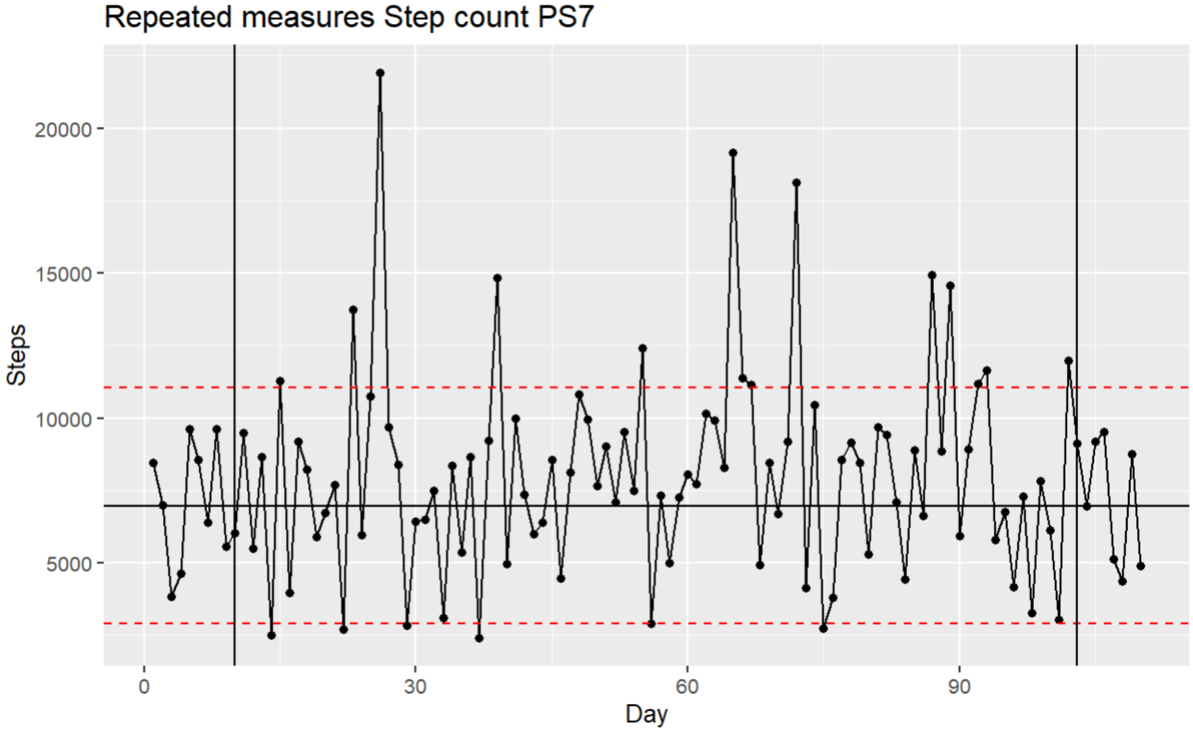

Repeated measures Step count PS7
Steps
20000
15000
10000
5000
0
30
60
90
Day

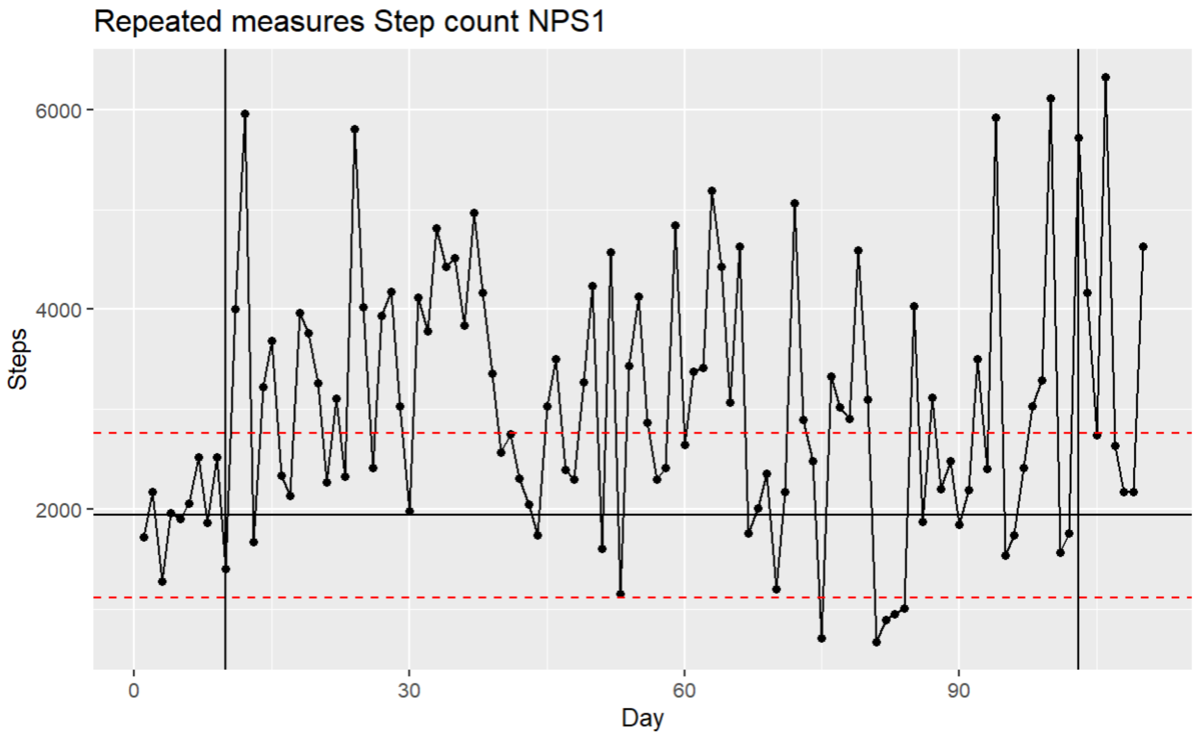

Repeated measures Step count NPS1
Steps
6000
4000
2000
0
30
60
90
Day

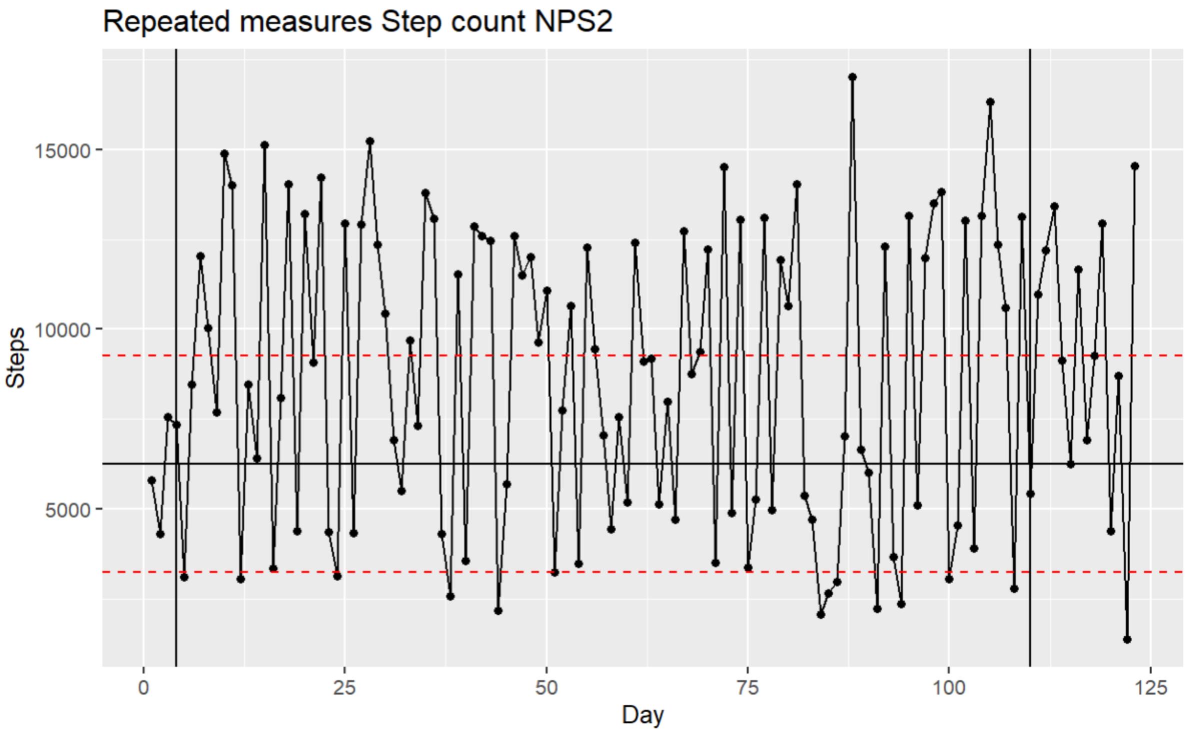

Repeated measures Step count NPS2
Steps
15000
10000
5000
0
25
50
75
100
125
Day

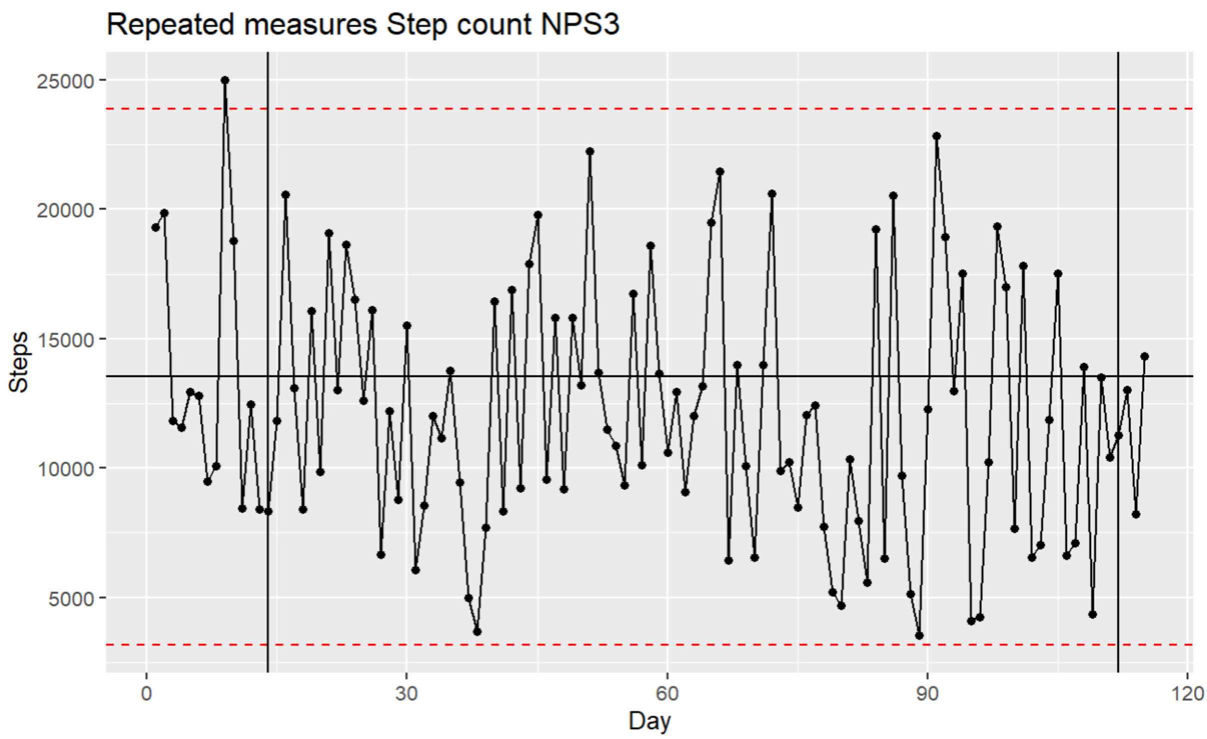

Repeated measures Step count NPS3
Steps
Day
25000
20000
15000
10000
5000
0
30
60
90
120

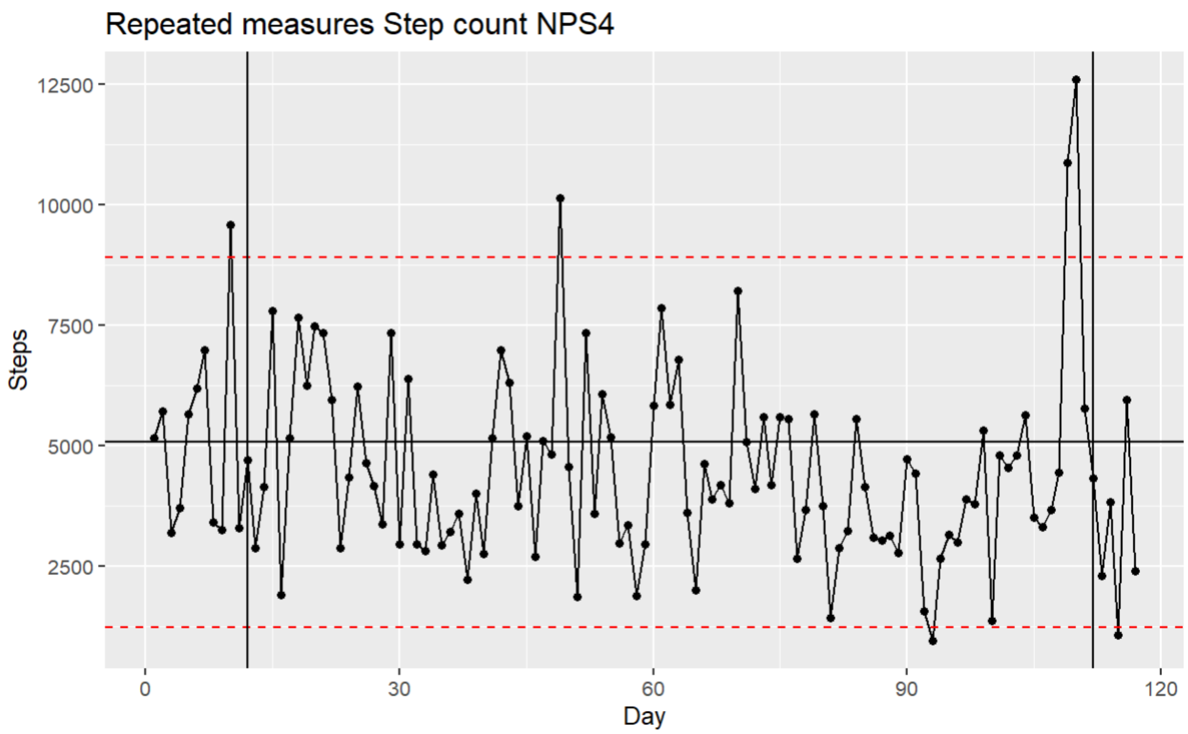

Repeated measures Step count NPS4
Steps
Day
12500
10000
7500
5000
2500
0
30
60
90
120

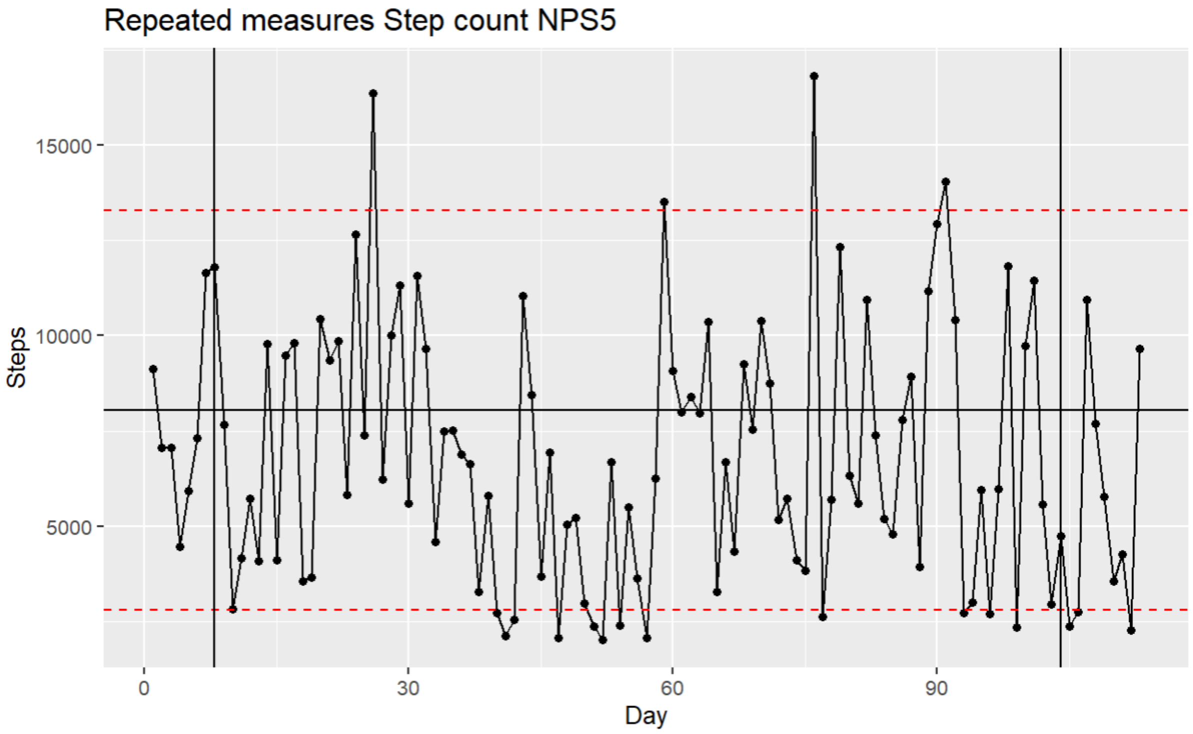
Repeated measures Step count NPS5
Steps
15000
10000
5000
0
30
60
90
Day

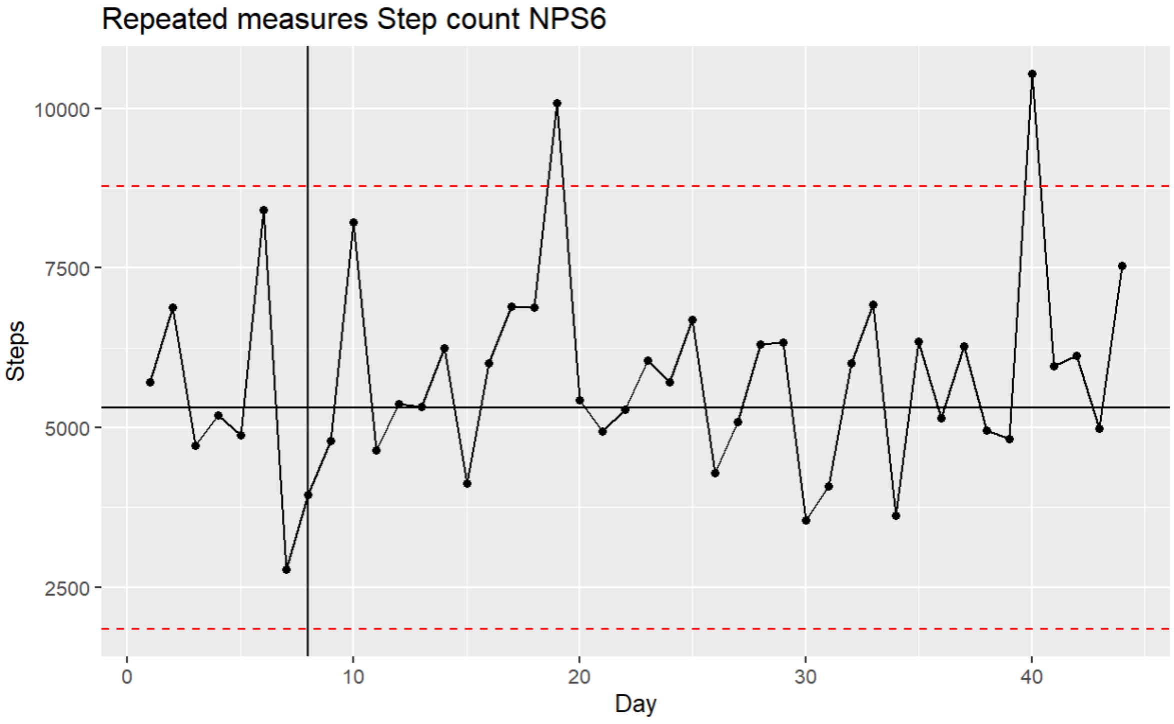
Repeated measures Step count NPS6
Steps
10000
7500
5000
2500
0
10
20
30
40
Day

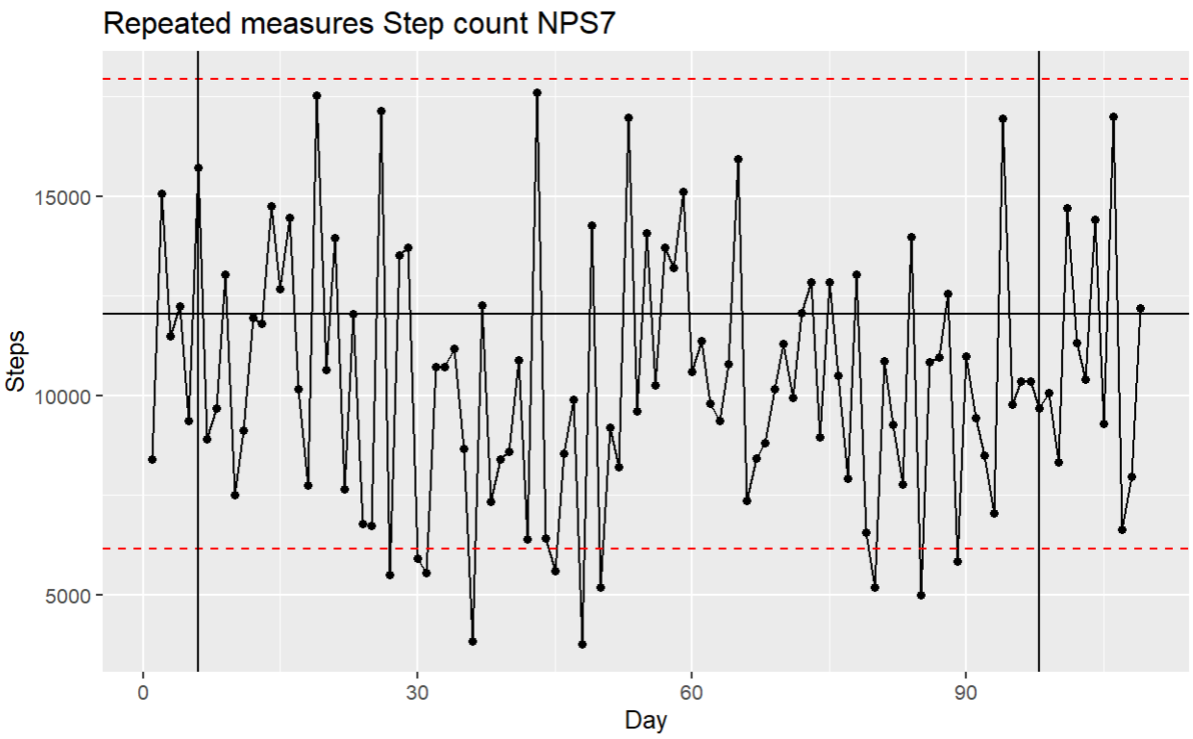

Repeated measures Step count NPS7
Steps
Day
15000
10000
5000
0
30
60
90

Time spend Sleeping

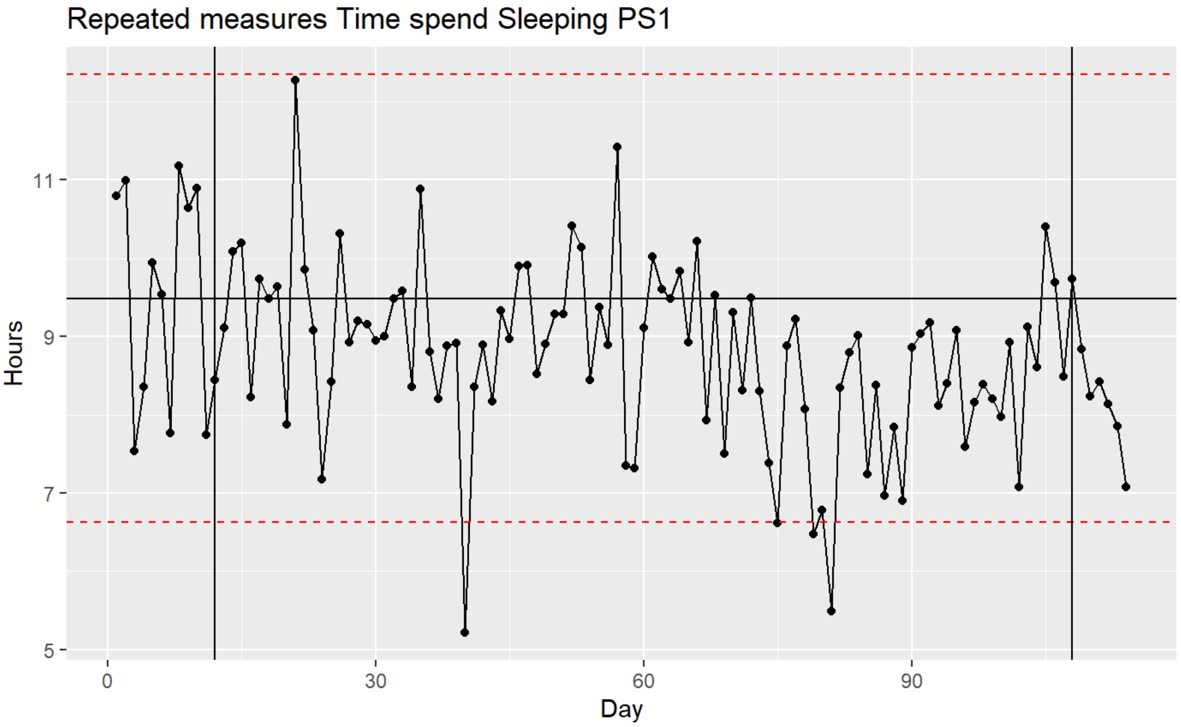

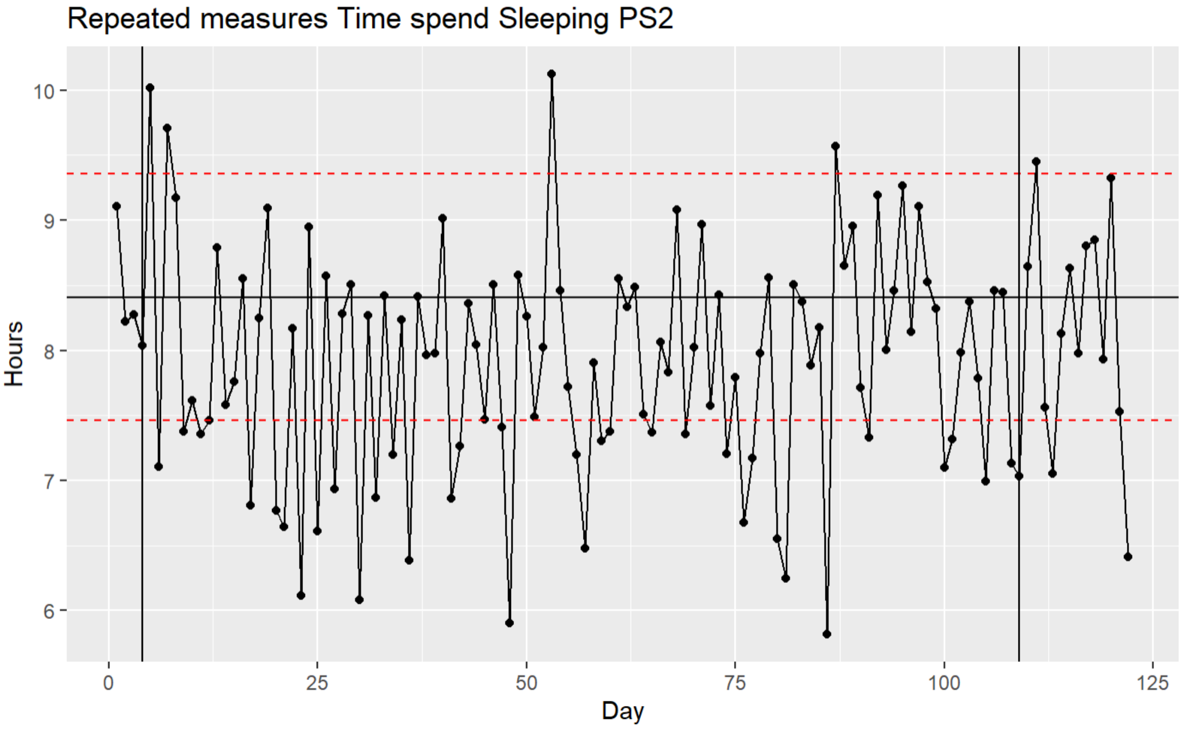

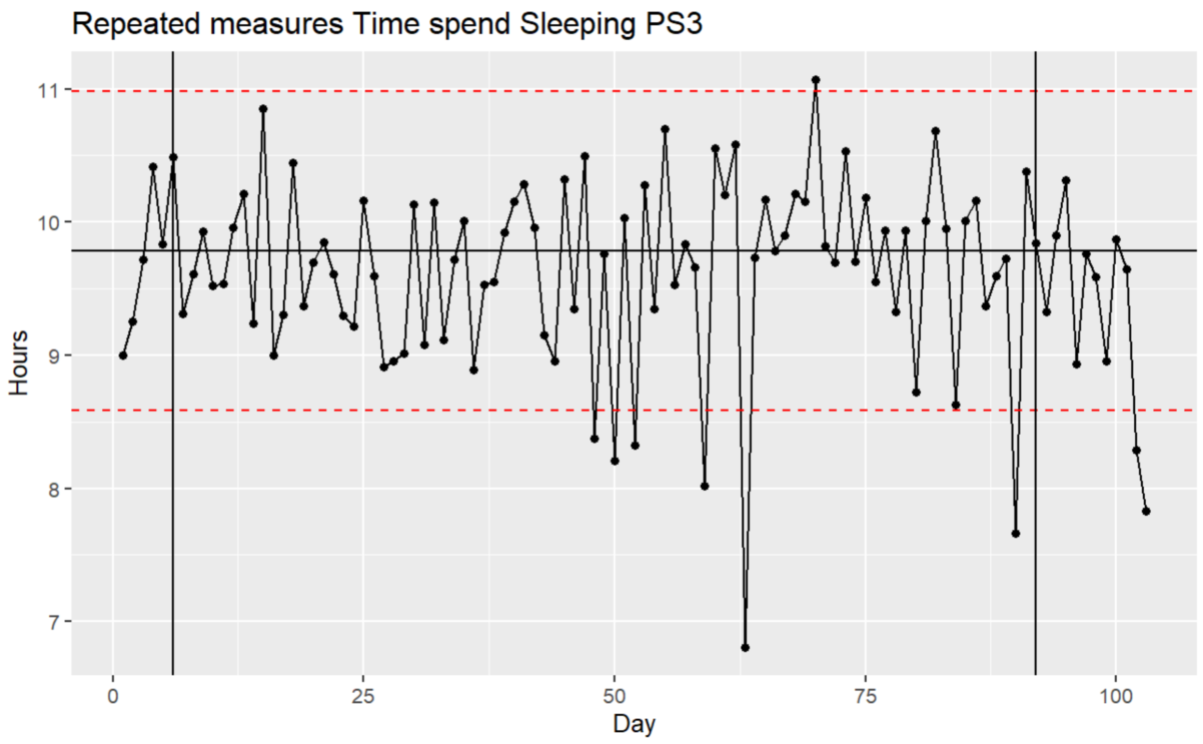

Repeated measures Time spend Sleeping PS3
Hours
11
10
9
8
7
0
25
50
75
100
Day

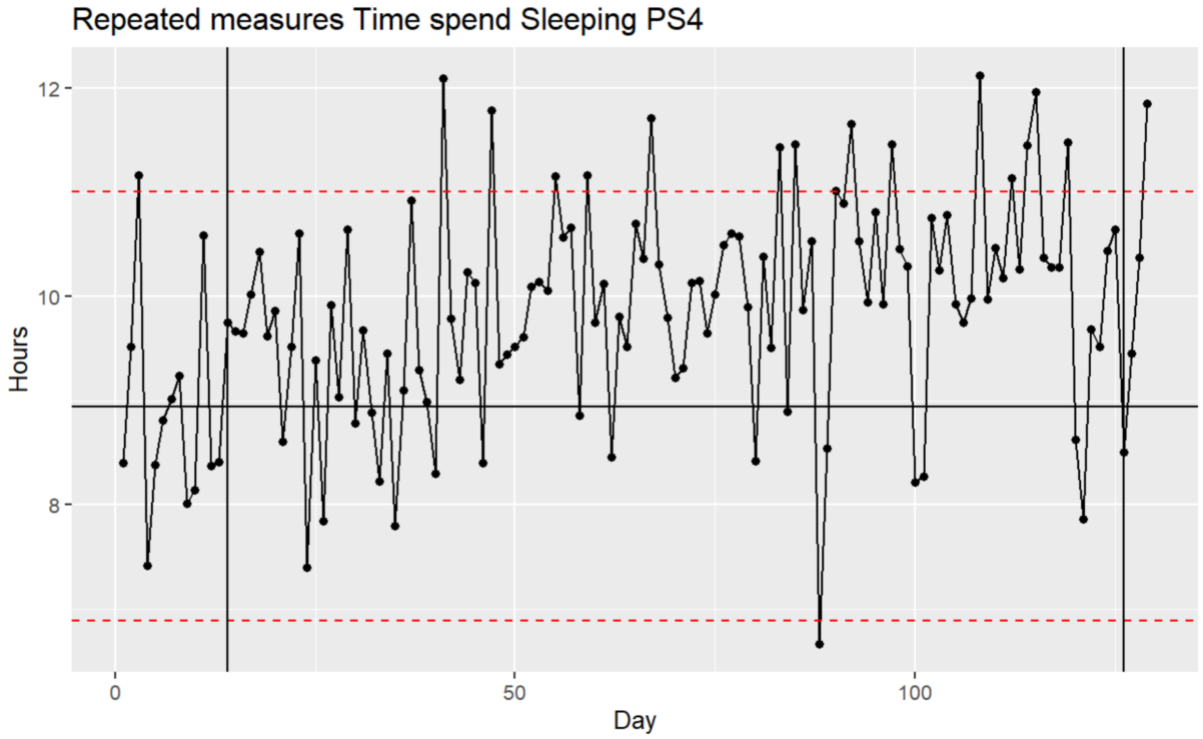

Repeated measures Time spend Sleeping PS4
Hours
12
10
8
0
50
100
Day

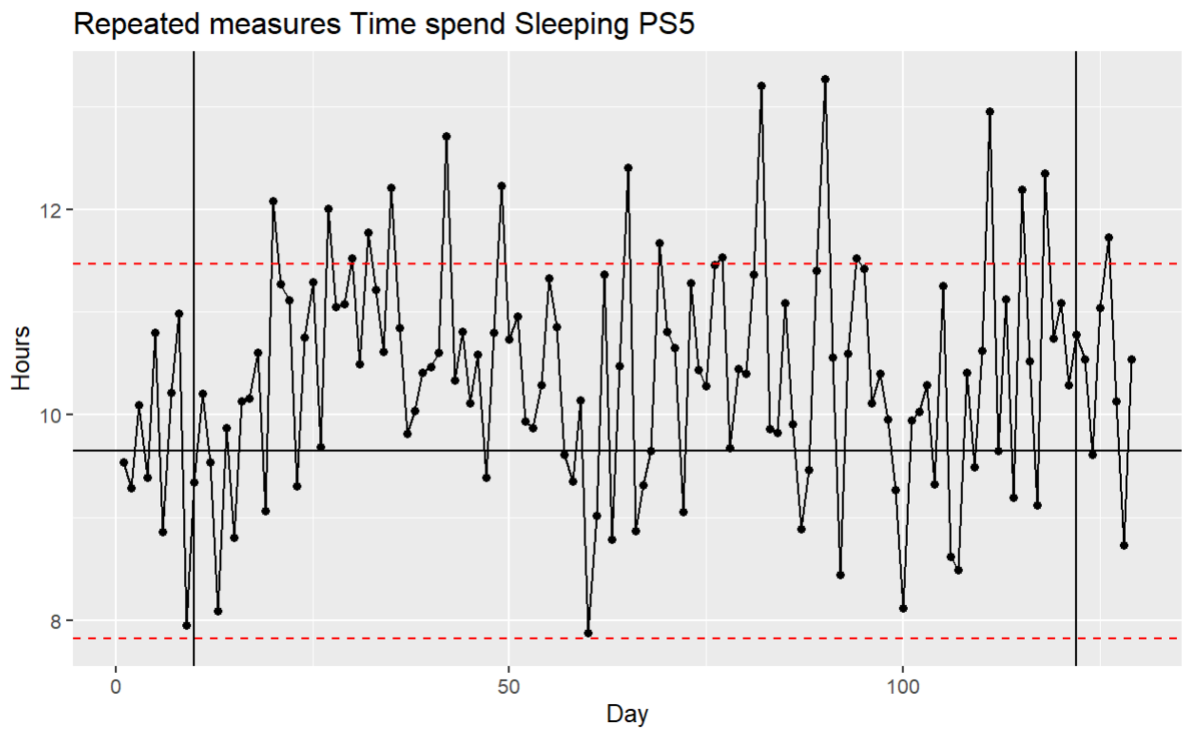
Repeated measures Time spend Sleeping PS5
Hours
12
10
8
0
50
100
Day

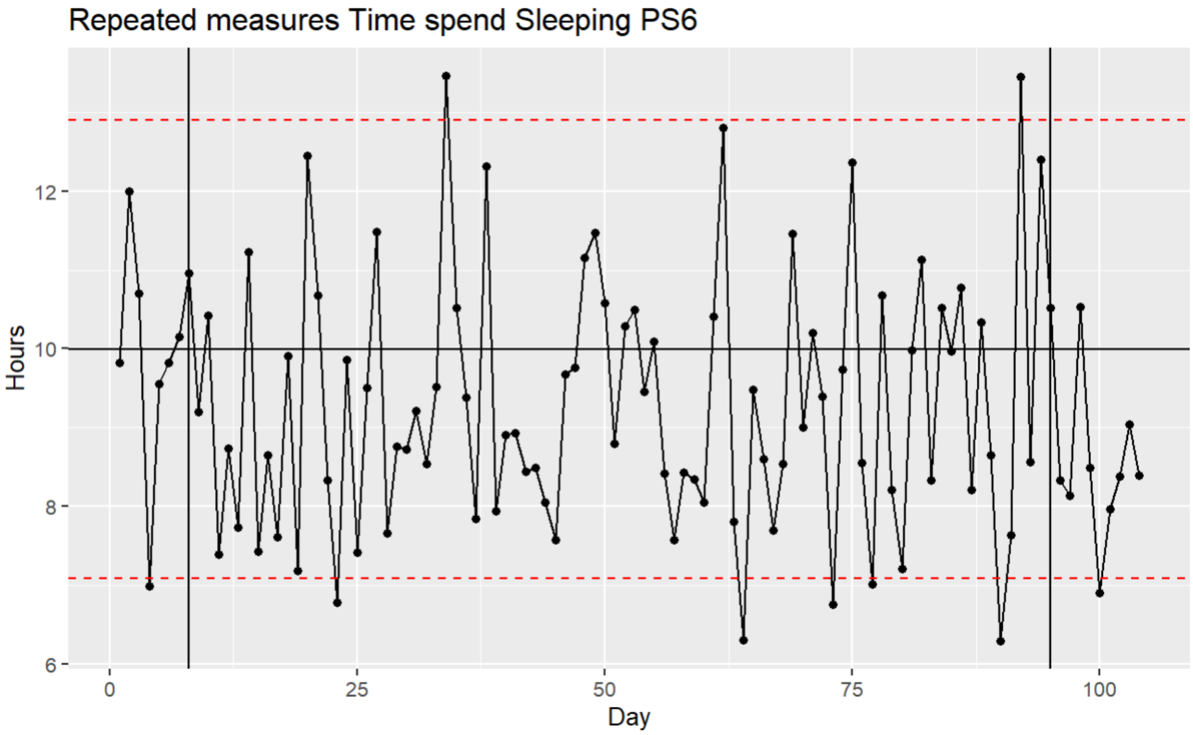
Repeated measures Time spend Sleeping PS6
Hours
12
10
8
6
0
25
50
75
100
Day

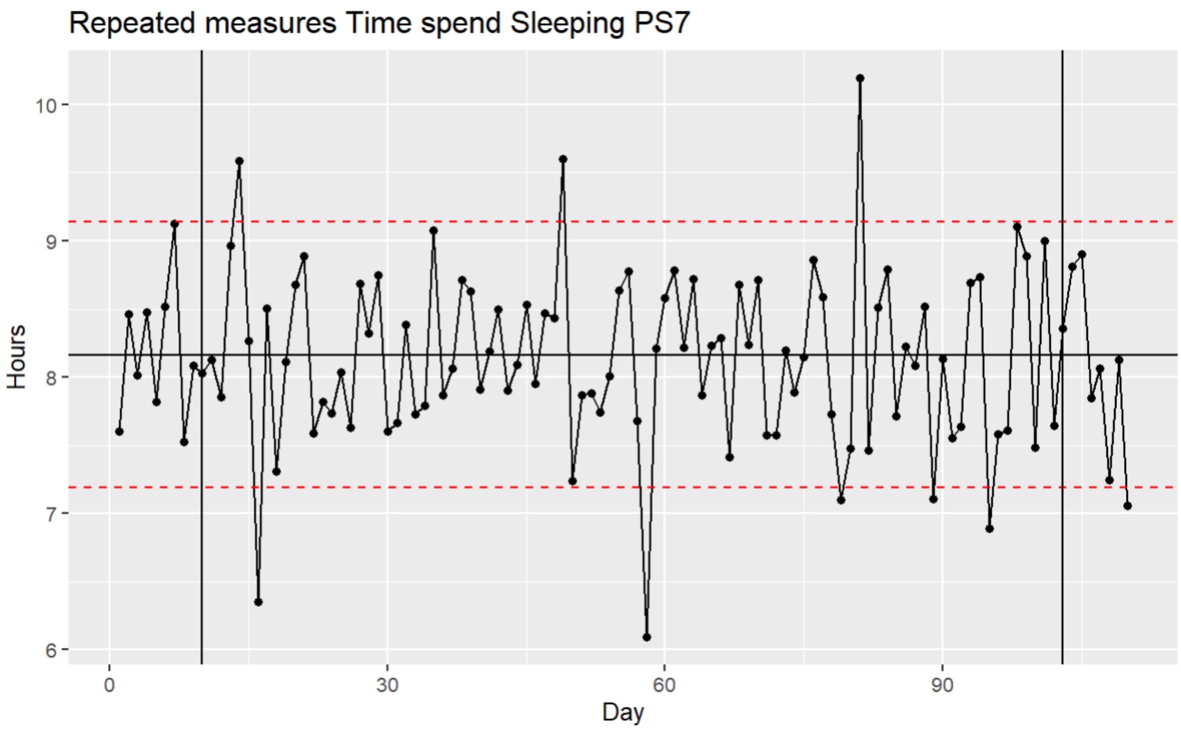

Repeated measures Time spend Sleeping PS7
Hours
10
9
8
7
6
0
30
60
90
Day

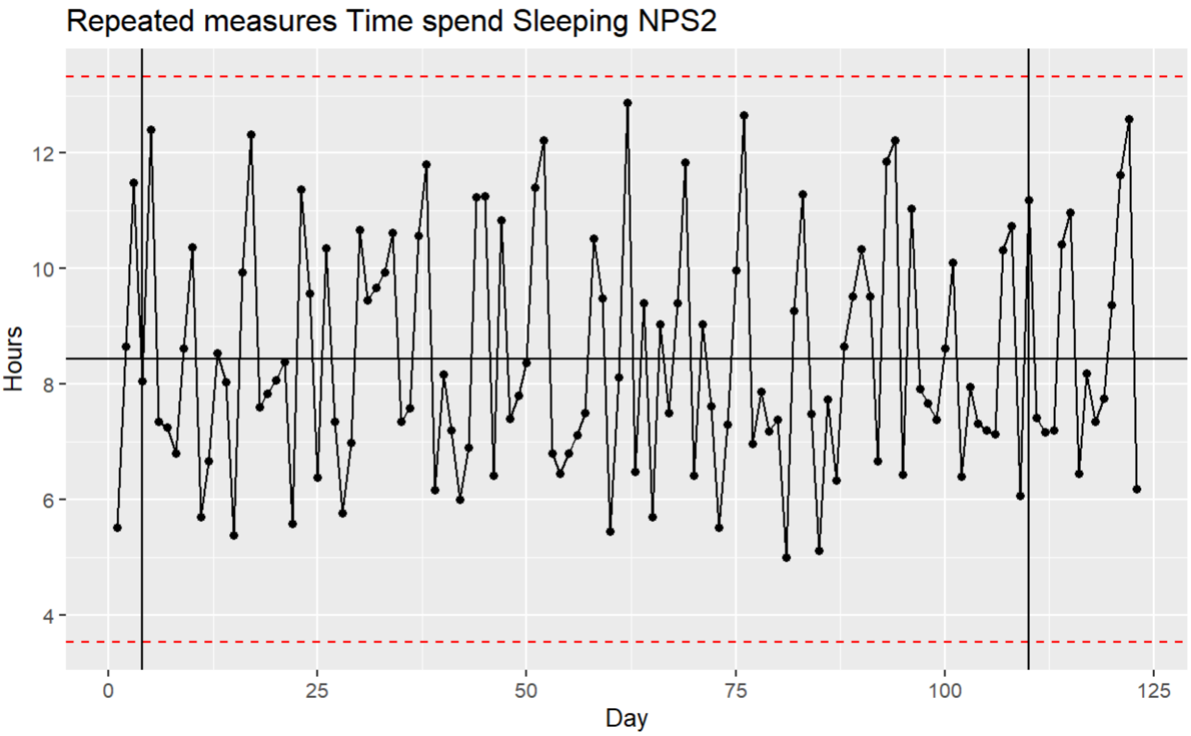
Repeated measures Time spend Sleeping NPS2
Hours
12
10
8
6
4
0
25
50
75
100
125
Day

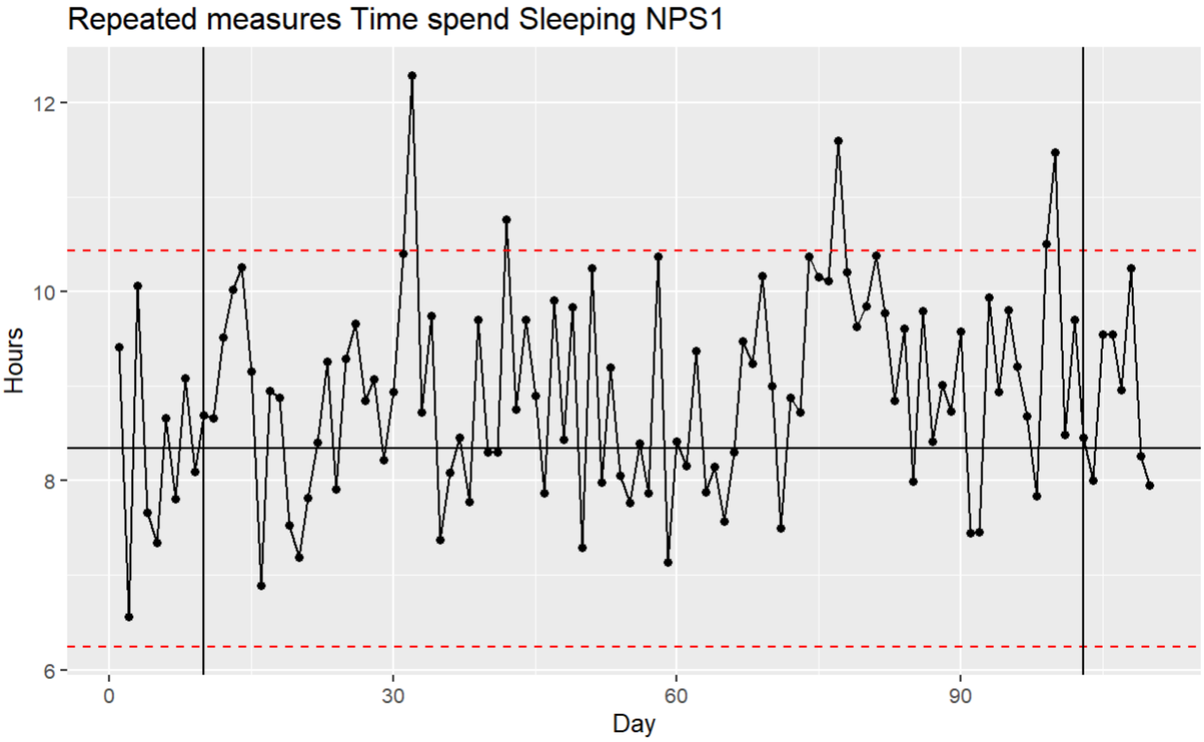
Repeated measures Time spend Sleeping NPS1
Hours
12
10
8
6
0
30
60
90
Day

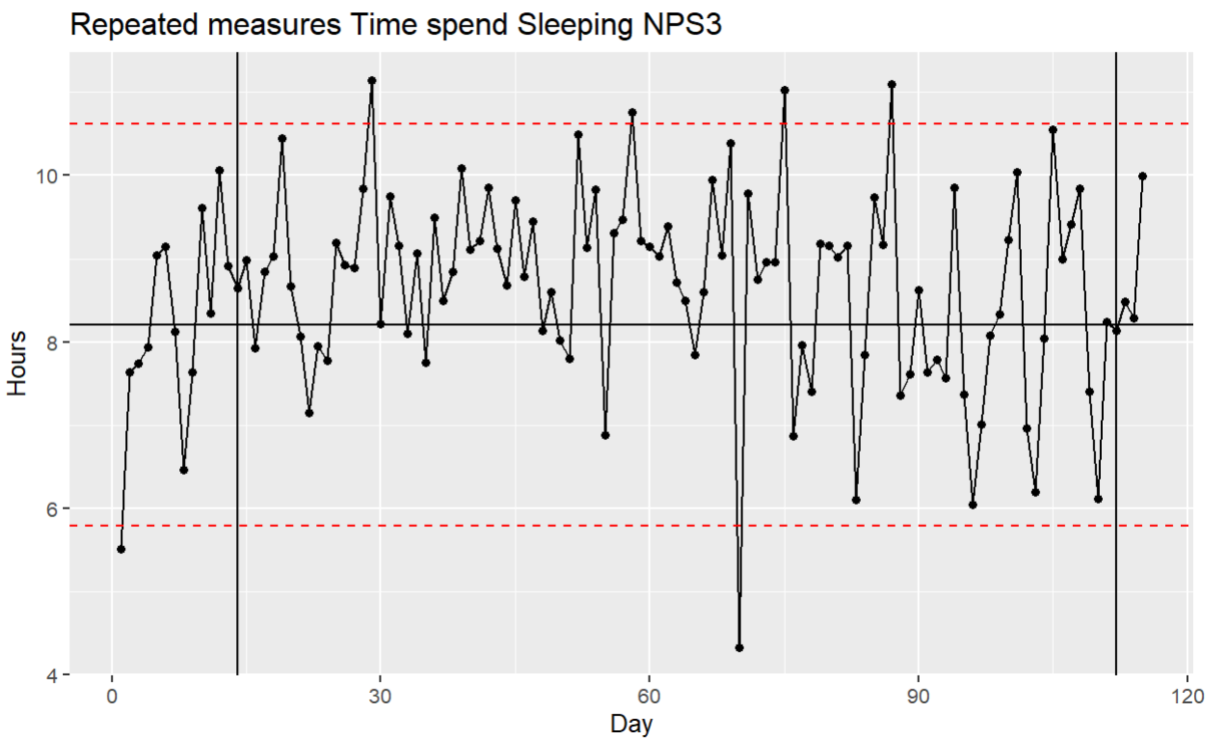

Repeated measures Time spend Sleeping NPS3
Hours
Day

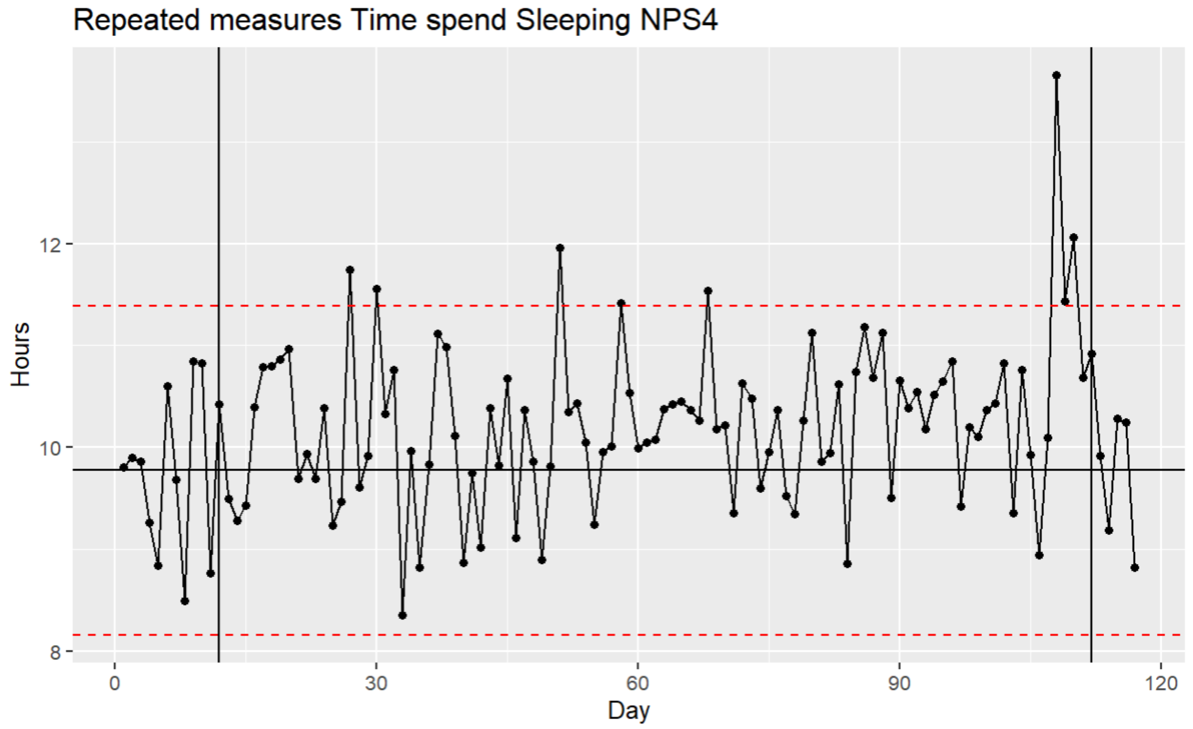

Repeated measures Time spend Sleeping NPS4
Hours
Day

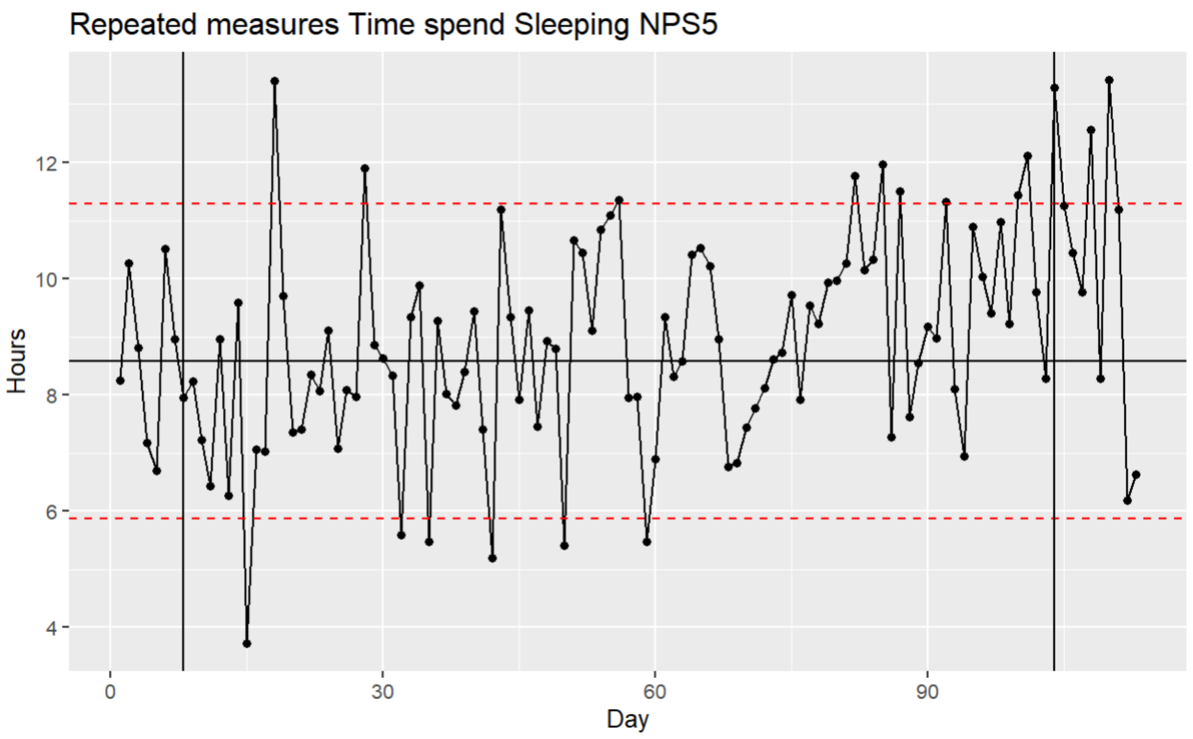

Repeated measures Time spend Sleeping NPS5
Hours
Day
12
10
8
6
4
0
30
60
90

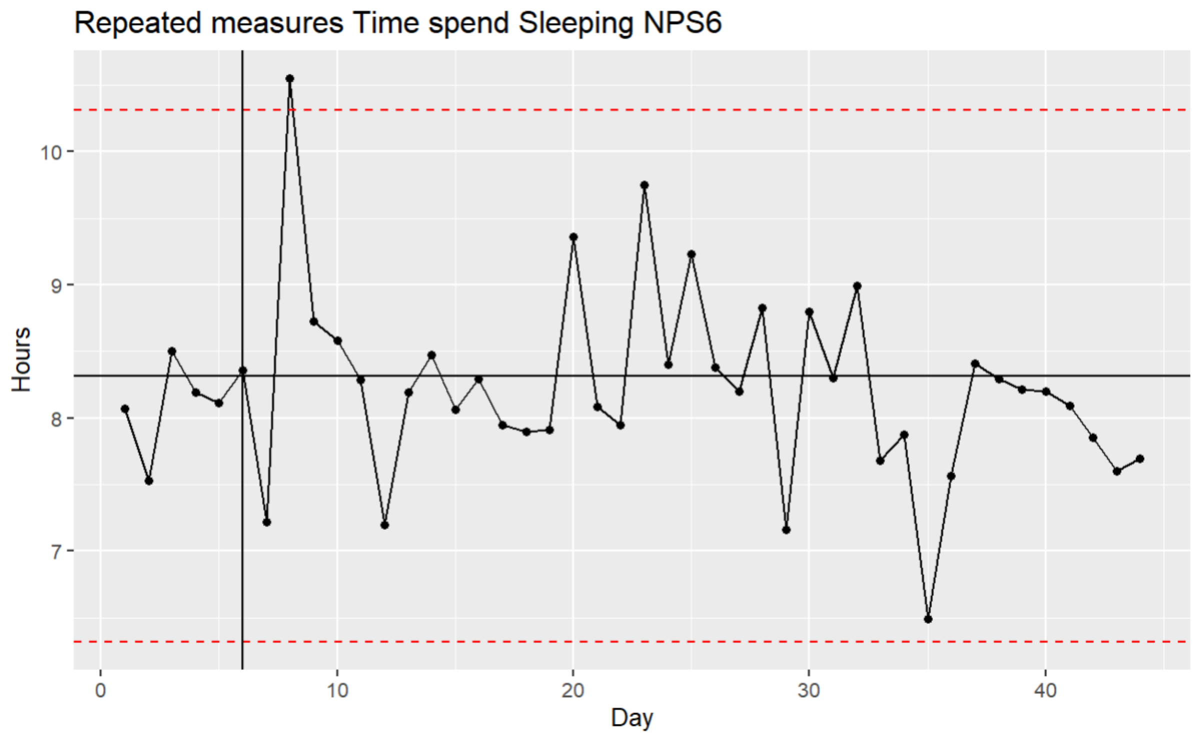

Repeated measures Time spend Sleeping NPS6
Hours
Day
10
9
8
7
0
10
20
30
40

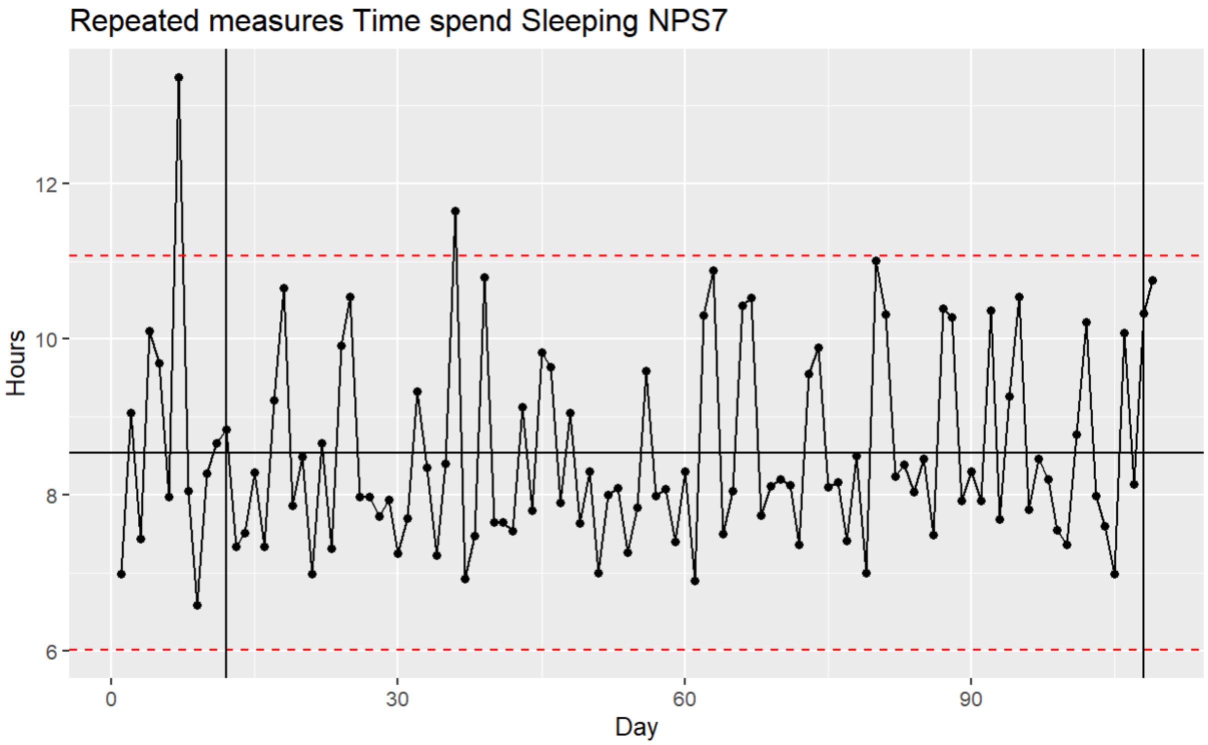
Repeated measures Time spend Sleeping NPS7
Hours
12
10
8
6
0
30
60
90
Day

Table Percentage Exceeding the Mean scores Physical Activity

Table PEM and mean scores (SD) Physical Activity

**With Participatory Support**	**Time in LPA (hr)**			**Time in MVPA (min)**		
Participant	Percentage Exceeding baseline Median (PEM, %)	mean [SD] phase A phase B+A'	Difference phase A phase B+A'	Percentage Exceeding baseline Median (PEM, %)	mean [SD] phase A phase B+A'	Difference phase A phase B+A'
**1**	**73.5%**	3.4 (1.1)<br>4.0 (1.2)	**0.6**	**89.2%**	48.8 (36.6)<br>70.1 (26.1)	**21.3**
**2**	**62.7%**	3.2 (0.8)<br>3.6 (1.0)	**0.4**	**79.7%**	32.4 (10.5)<br>44.4 (17.8)	**12**
**3**	57.7%	3.5 (0.9)<br>3.7 (0.8)	0.2	52.6%	41.1 (10.5)<br>45.1 (13.6)	4
**4**	**100.0%**	2.1 (0.4)<br>6.3 (1.9)	**4.2**	52.2%	15.9 (6.6)<br>15.2 (5.4)	-0.7
**5**	58.8%	2.6 (0.7)<br>3.1 (1.4)	0.5	53.8%	22.8 (5.7)<br>27.4 (16.3)	4.6
**6**	**78.1%**	2.4 (1.5)<br>3.9 (2.0)	**1.5**	**92.7%**	26.4 (21.3)<br>63.6 (31.0)	**37.2**
**7**	**93.0%**	2.5 (0.6)<br>3.7 (1.1)	**1.2**	57.0%	54.1 (15.1)<br>62.3 (29.3)	8.2

**Without Participatory Support**	**Time in LPA (hr)**			**Time in MVPA (min)**		
Participant	Percentage Exceeding baseline Median (PEM, %)	mean [SD] phase A phase B+A'	Difference phase A phase B+A'	Percentage Exceeding baseline Median (PEM, %)	mean [SD] phase A phase B+A'	Difference phase A phase B+A'
**1**	**84.0%**	1.7 (0.5)<br>2.5 (0.8)	**0.8**	**83.0%**	14.7 (3.3)<br>26.1 (12.3)	**11.4**
**2**	**72.3%**	2.6 (0.8)<br>3.6 (1.5)	**1**	**63.9%**	54.2 (16.6)<br>77.3 (39.1)	**23.1**
**3**	33.7%	3.0 (0.9)<br>2.7 (0.9)	-0.3	46.5%	104.4 (38.1)<br>92.4 (37.0)	-12
**4**	39.0%	2.5 (0.7)<br>2.2 (0.5)	-0.3	34.3%	42.3 (17.6)<br>35.7 (17.7)	-6.6
**5**	**72.4%**	4.3 (0.7)<br>4.9 (1.4)	**0.6**	40.0%	63.8 (23.6)<br>52.9 (30.7)	-10.9

**6***	58.3%*	4.1 (1.2)<br>4.0 (0.7)	-0.1	**69.4%***	42.4 (14.9)<br>48.5 (14.6)	**6.1**
**7**	**69.9%**	3.4 (1.0)<br>4.8 (2.0)	**1.4**	26.2%	97.9 (27.3)<br>84.3 (28.8)	-13.6

PEM: Percentage exceeding the median; represents the percentage of days, during and after the intervention, in which there was an improvement compared to the baseline median.

Hr: Hours

Phase A: baseline phase; Phase B: intervention phase; Phase A': post intervention phase

SD: standard deviation

Green: effect of either high (>90) or moderate (70-90); Blue: mild (60-70); Black: questionable effect (50-60) or no effect (<50);

* Pt who dropped out early
