# Supplementary figures and images for: Improving Movement Behavior in People after Stroke with the RISE Intervention: A Randomized Multiple Baseline Study
Source: J Clin Med. 2024 Jul 25;13(15):4341. doi: 10.3390/jcm13154341 (PMC11313465; doi:10.3390/jcm13154341)

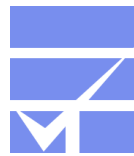

## CONSORT 2010 Flow Diagram

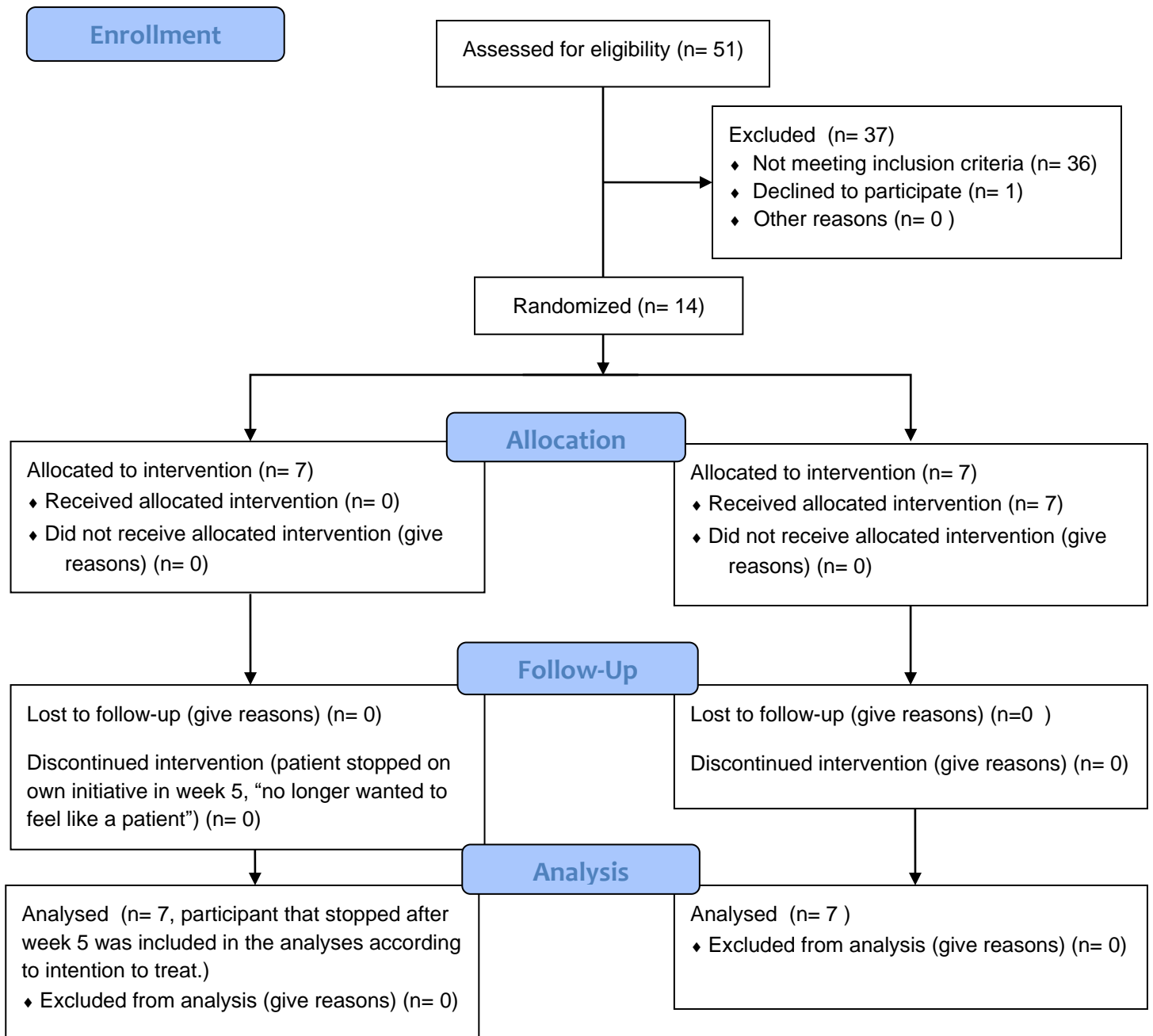

Supplement: Supplementary file 1 [file jcm-13-04341-s001.zip › consort-2010-flow-diagram.pdf]
